# Merging Directed C–H Activations with High-Throughput Experimentation: Development of Iridium-Catalyzed C–H Aminations Applicable to Late-Stage Functionalization

**DOI:** 10.1021/jacsau.2c00039

**Published:** 2022-04-13

**Authors:** Erik Weis, Maria Johansson, Pernilla Korsgren, Belén Martín-Matute, Magnus J. Johansson

**Affiliations:** † Department of Organic Chemistry, Stockholm University, Stockholm, SE 106 91, Sweden; ‡ Medicinal Chemistry, Research and Early Development; Cardiovascular, Renal and Metabolism, Biopharmaceuticals R&D, AstraZeneca, Pepparedsleden 1, Mölndal, 431 50 Gothenburg, Sweden; § Compound Synthesis and Management, Discovery Sciences, BioPharmaceuticals R&D, AstraZeneca, Pepparedsleden 1, Mölndal, 431 50 Gothenburg, Sweden

**Keywords:** catalysis, C−H activation, C−H amination, C−H functionalization, high-throughput experimentation, HTE, iridium, late-stage functionalization, LSF

## Abstract

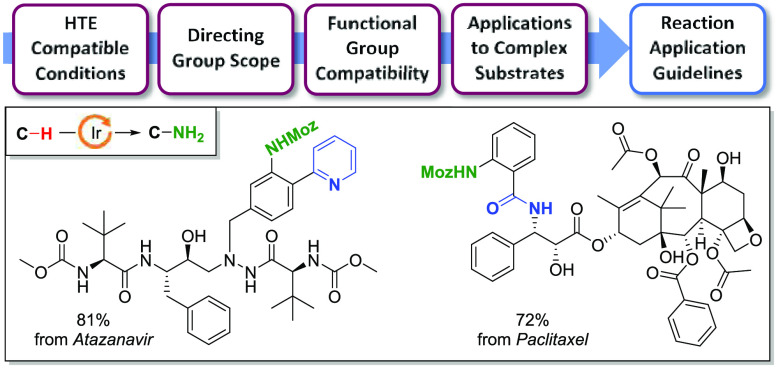

Herein, we report an iridium-catalyzed directed C–H amination methodology developed using a high-throughput experimentation (HTE)-based strategy, applicable for the needs of automated modern drug discovery. The informer library approach for investigating the accessible directing group chemical space, in combination with functional group tolerance screening and substrate scope investigations, allowed for the generation of reaction application guidelines to aid future users. Applicability to late-stage functionalization of complex drugs and natural products, in combination with multiple deprotection protocols leading to the desirable aniline matched pairs, serve to demonstrate the utility of the method for drug discovery. Finally, reaction miniaturization to a nanomolar range highlights the opportunities for more sustainable screening with decreased material consumption.

## Introduction

**Figure 1:**
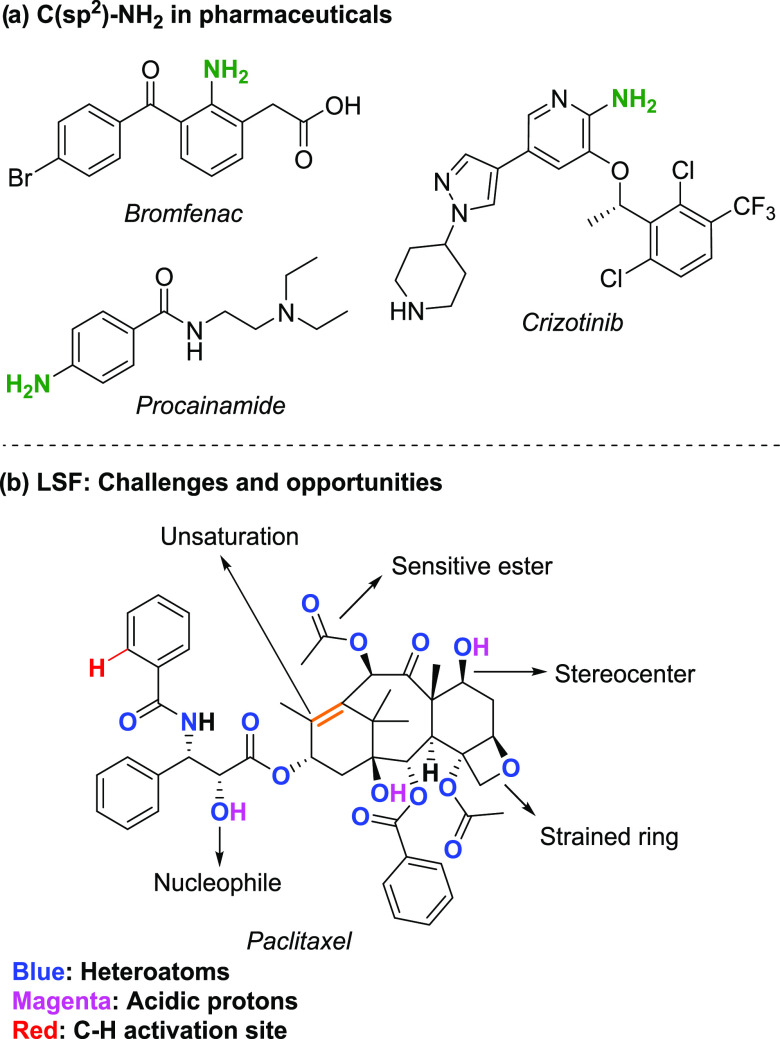
(a) Selected examples of drugs containing an aniline moiety and (b) representative examples of challenging functional groups encountered with LSF applications and opportunities for directed C–H activations.

Innovation in synthetic organic chemistry is of fundamental importance for the improvement of the drug discovery process. While the field has seen tremendous developments over the past century, recent advances in synthetic methods, chemoinformatics, and increasing applicability of automation and miniaturization in synthesis have the potential to further transform and improve modern drug discovery.^1−4^ Two particular technological and synthetic approaches stand at the forefront of our interest and focus in this work: high-throughput experimentation (HTE) and C–H functionalization. HTE techniques attracted significant interest from the pharmaceutical industry and are now increasingly utilized in the drug discovery process.^5^ From the methodology development perspective, the advantages are clear: access to more high-quality and well-rounded results with decreased material and time consumption associated. Adding to this is the importance of large, high-quality datasets for the generation of predictive reactivity models.^6,7^ At the same time, reaction miniaturization allows for more sustainable chemistry by means of decreased material consumption, including reagents, solvents, and especially high-value advanced intermediates and catalysts. Finally, technologies such as automated liquid and solid dispensing allow chemists to avoid repetitive nonintellectual tasks, while providing high reproducibility and evading the risk for human error in setting up large arrays. Given the abundance of C–H bonds in drugs and their building blocks, C–H functionalizations are among the most desirable transformations in drug discovery. Of particular interest are late-stage functionalizations (LSF),^8,9^ where the controlled chemoselective transformation of the desired C–H bonds in complex drug-like molecules has the potential to greatly aid in the hit-to-lead and lead optimization processes.^10^ Bypassing the need for time-, material-, and labor-intensive *de novo* synthesis of analogues would greatly aid in structure–activity relationship (SAR) studies or even the generation of new candidate drugs. In terms of desirable transformations, the introduction of small functional groups such as −CH_3_, −CF_3_, −NH_2_, −OH, and −F is of highest priority and would be widely used in the industry.^1^ Further motivating the development of new amination methodologies, a recent analysis of X-ray structural data identified N–H hydrogen bond donors on aromatic and aliphatic amines as the most common polar functional groups involved in fragment–protein binding (for examples of anilines, see Figure 1a).^2^ Directed C–H activations offer a means of introducing amine moieties in the vicinity of Lewis basic groups commonly present in drug-like molecules with high regioselectivity. Over the past decade, a number of methodologies for C(sp^2^)–H to C(sp^2^)–N bond transformation have been developed,^10−14^ utilizing among others Co,^15,16^ Rh,^17−19^ Ir,^20−24^ and Ru^25−28^ catalysts. However, the applicability of LSF in a drug discovery context remains challenging. We identified several factors that limit the utility of the reported C–H aminations in this respect. First is the inaccessibility of free amines. The majority of the reported directed C–H to C–N bond-forming reactions, while introducing protected amines in the form of amides and sulfonamides, do not include deprotection protocols. Although the introduction of larger substituents can be of utility for fragment-based drug discovery, applications to LSF are of limited use if the free amines cannot be obtained under mild-enough conditions to tolerate a large array of reactive and/or sensitive functional groups present in drug-like molecules. Second is the limited functional group tolerance. A common shortcoming of the reported procedures is a lack of compatibility with polar functional groups commonly present in drug-like molecules, such as heterocycles, alcohols, amines, carboxylic acids, or amides (practical examples in Figure 1b). The third limitation is closely related to this, and it is the lack of reporting of unsuccessful transformations and limited number of reports on applicability to complex substrates. This situation would be largely mitigated by full disclosure of the investigated substrate scope. Aside from this, two distinct approaches have been recently developed to improve the predictability of chemical methods in a more systematic fashion: the intermolecular robustness screening approach developed by the Glorius group^29−31^ and the chemistry informer library approach developed by Krska and co-workers.^7,32^ The intermolecular robustness screening approach evaluates the compatibility of additives bearing a wide variety of functional groups with the transformation of a single substrate. In the informer library approach, the compatibility of a methodology with a large number of complex substrates bearing structural features relevant to pharmaceuticals is evaluated. While the former has been previously used for the reported C–H activation methodologies,^33,34^ including our own,^34^ the latter has so far only been applied to more well-established cross-coupling reactions.^7,32^

Herein, we report the development of an iridium-catalyzed directed *ortho-*C–H amination applicable to a large number of directing groups (DGs) with outstanding functional group tolerance and regioselectivity. The use of the [Cp*Ir(H_2_O)_3_]SO_4_ catalyst allows for regioselective functionalization governed by DGs inherently present in building blocks, drugs, and natural products, without the need for additional ligands. Reaction application guidelines based on a DG informer library, functional group tolerance studies, and LSF informer library aid the potential users in assessing reaction applicability for complex substrates. The obtained Moz-protected amines can be deprotected under three distinct conditions, further increasing the utility of the amination protocol to complex molecules.

## Results and Discussion

**Figure 2:**
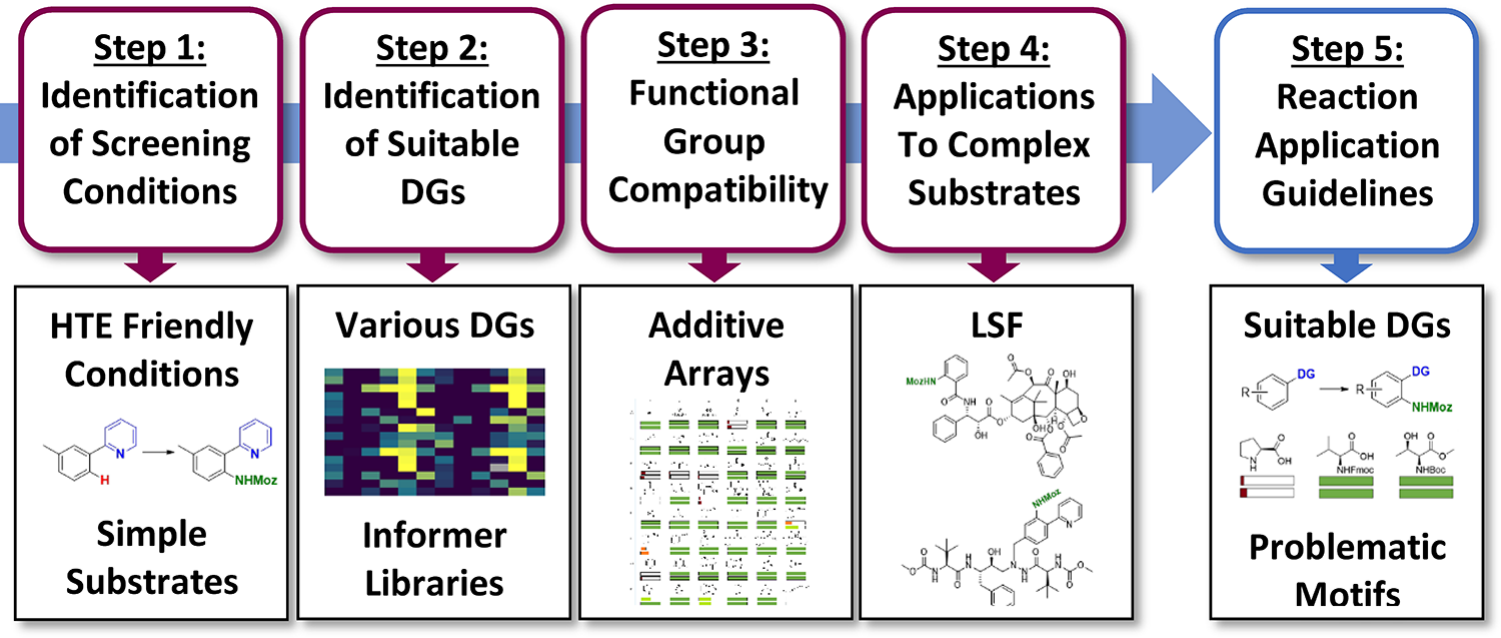
Strategy for HTE-enabled reaction discovery and applicability investigations for methodology development. DG = directing group.

At the beginning of the study, we designed a workflow (Figure 2) which, if successful, would deliver reaction conditions for LSF applications. In the initial stage, an optimization study to find suitable screening conditions was undertaken (Figure 2, step 1). In terms of reaction conditions, we identified several desirable features of “ideal” C–H activation methodologies applicable to HTE.^34−36^ These include (1) use of soluble reagents and liquid dispensing—beyond the ease of setting up complex libraries, this would also allow further decreasing the reaction scale with maintained reproducibility, surpassing the limitations of solid dispensing methods, (2) reactions tolerant toward moisture, allowing for direct use of reagents and large compound libraries without the need of rigorous drying, (3) reactions tolerant toward the air atmosphere, (4) commercially available reagents, (5) compatibility with plastic reaction vessels, and (6) compatibility with nonvolatile reaction solvents. The last two points are crucial for miniaturization and use of 384- and 1536-well reaction plate formats.

The following set of conditions was identified based on these criteria after step 1, initial optimizations (see the Supporting Information), and used for the directing group informer library. [Cp*Ir(H_2_O)_3_SO_4_] was chosen as a catalyst,^34,37^ allowing the reaction to be performed in the absence of silver salts and insoluble additives, thus facilitating the use of liquid-handling systems. Commercially available MozN_3_ (Moz = *p*-methoxybenzyloxycarbonyl) was selected as the nitrogen source, allowing for deprotection of the obtained carbamate under a number of conditions.^38,39^ Although the transformation was performed with a satisfactory outcome in a wide range of solvents (see the Supporting Information), four were chosen for the informer library: 1,2-dichloroethane (DCE), which performed best in the initial study, cyclopentyl methyl ether (CPME) and EtOAc as greener solvent alternatives, and *N*-methyl-2-pyrrolidone (NMP) for its general good solubility of drug-like compounds and high boiling point.

**Figure 3:**
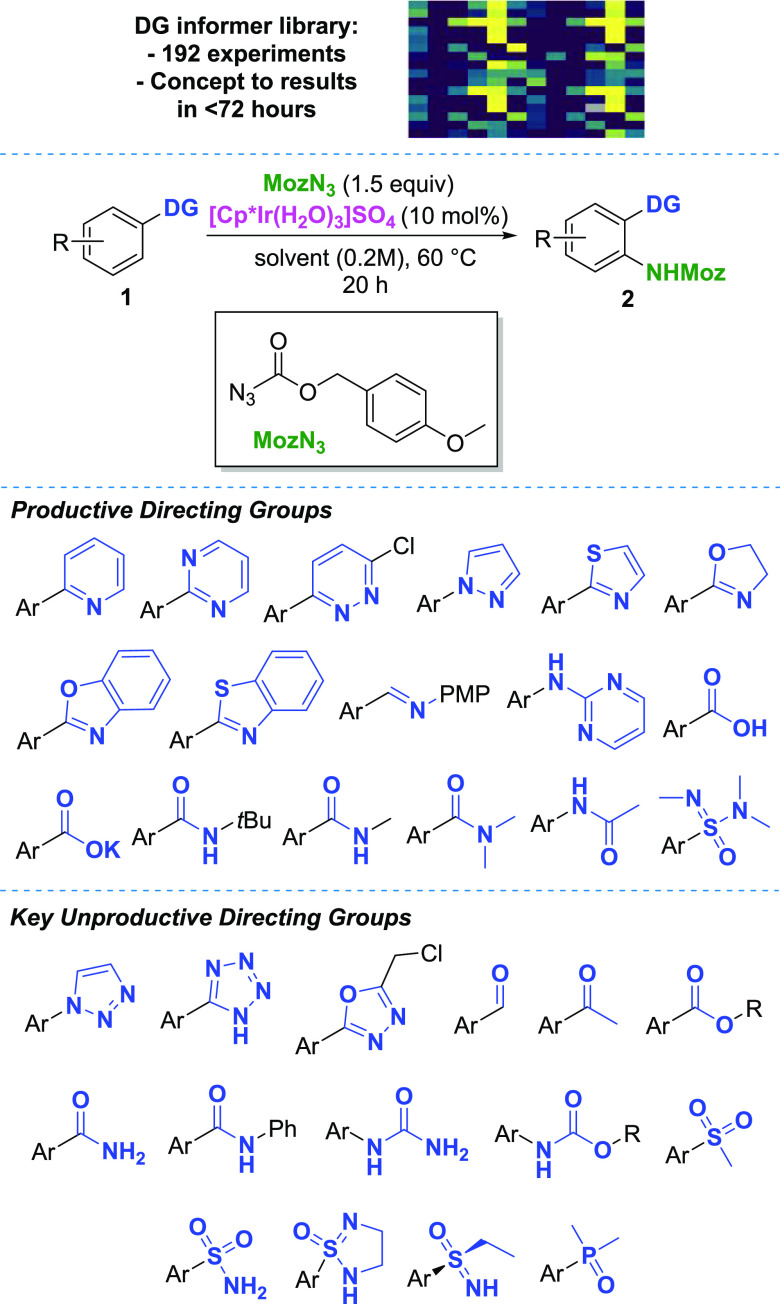
Directing group informer library. A total of 48 substrates tested against four solvents under screening conditions. Productive DGs and key classes of unproductive DGs depicted.

In step 2 (Figure 2), the DG chemical space was probed. Out of the 48 substrates tested under the screening conditions, 16 DGs were shown to be productive for the C–N bond formation, with observed conversions ranging from 10 to >99% (Figure 3, for the complete list including conversions and unproductive substrates, see the Supporting Information). Given the variety of DGs tested, this was an encouraging result. Despite being relatively low at the bottom end, we anticipated that the conversions could be improved by further optimization at a later stage. The following observations were made: DCE showed the best performance throughout the scope. EtOAc and CPME had similar applicability, albeit in some cases with lower conversions. NMP performed well with heterocycles and carboxylic acids; however, it was unproductive with the amide series. While the screening conditions allowed for the functionalization of a variety of substrates under a unified set of conditions, the HTE approach also facilitated rapid substrate-specific reaction optimization. Catalyst and reagent loading and mono/diselectivity were successfully optimized for a number of substrates (for detailed studies, see the Supporting Information).

**Scheme 1:**
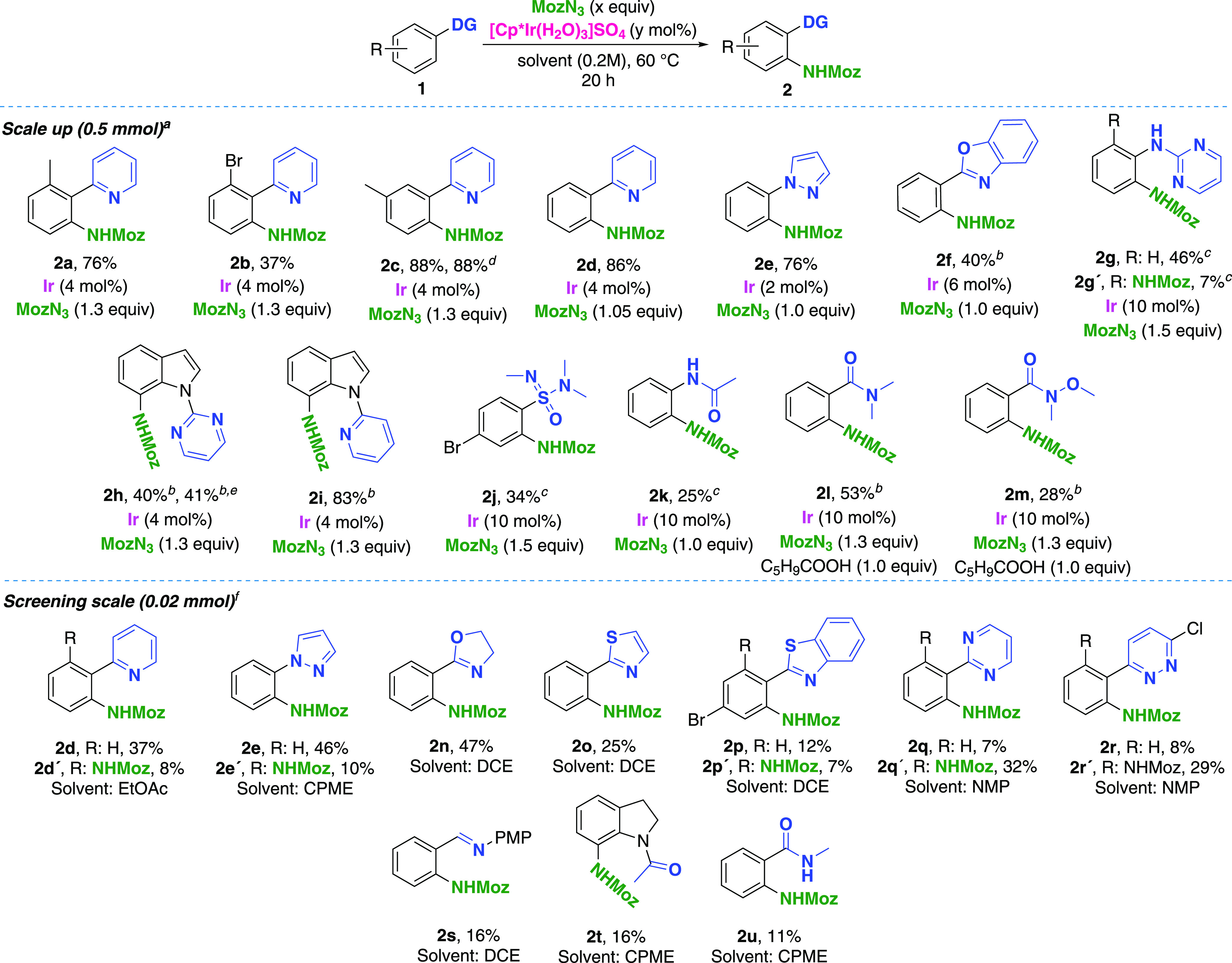
Scope: Building Blocks (Directing Groups Highlighted in Blue; Isolated Yields Shown) DCE used as solvent. Reaction scale 0.2 mmol. Reaction scale: 0.1 mmol. CPME as a solvent. EtOAc as a solvent. Isolated from DG informer library reaction plate. MozN_3_ (1.5 equiv), [Cp*Ir(H_2_O)_3_]SO_4_ (10 mol%).

**Scheme 2:**
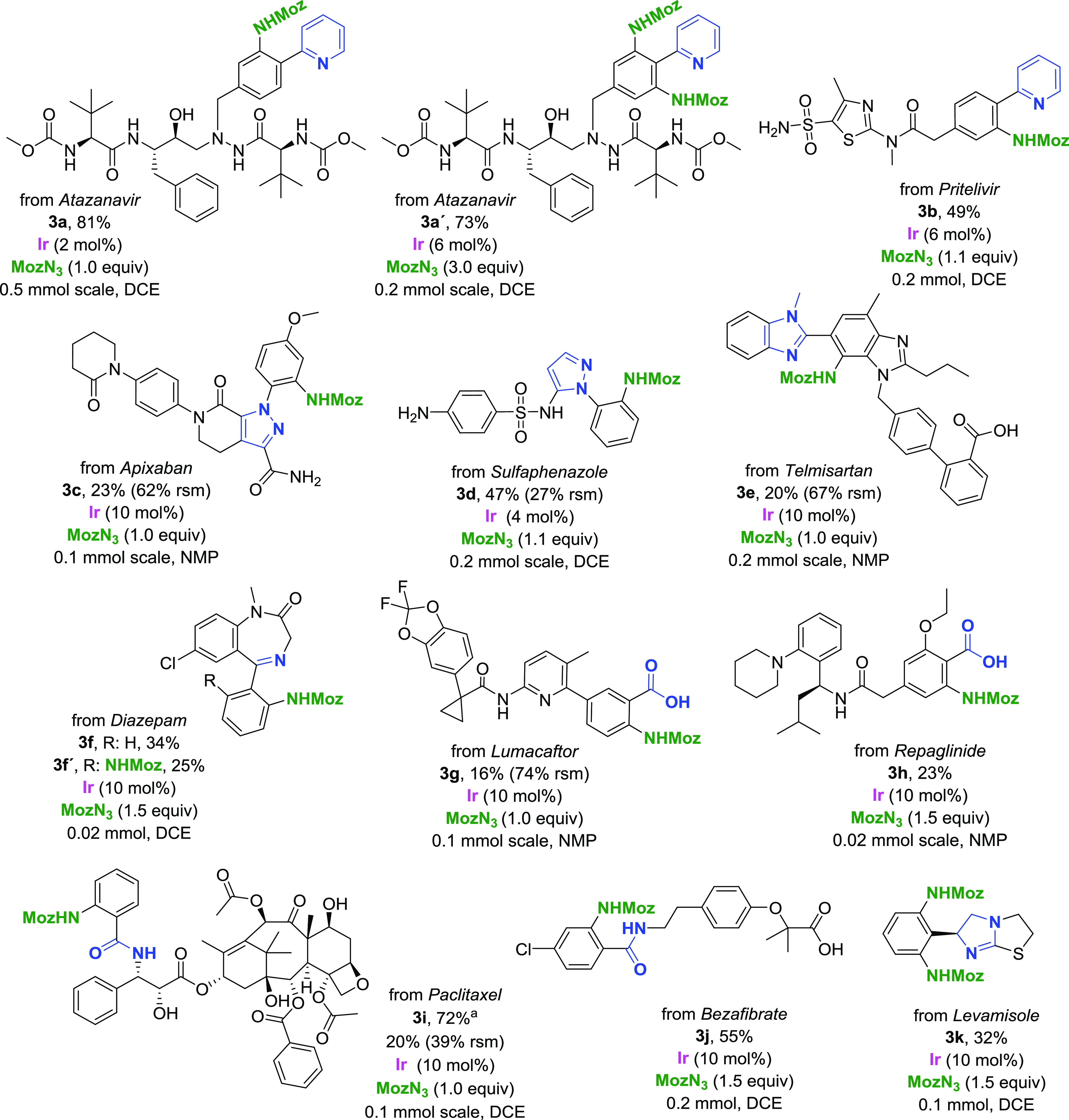
Scope: LSF (Directing Groups Highlighted in Blue; Isolated Yields Shown) C_5_H_9_COOH (1.0 equiv) used as additive. rsm = recovered starting material.

To confirm the performance of the catalytic system on a larger scale, a series of building blocks were functionalized and isolated (Scheme 1). The building block selection was based on positive hits from the directing group informer library (vide supra, Figure 3). For examples with a decreased catalyst and/or reagent loading (compared to screening conditions in Figure 3), the modified conditions were reached by HTE single-substrate optimization (see the Supporting Information). The effect of various substitution patterns on the functionalized system was investigated with the 2-phenylpyridine series. While the *ortho*-methyl substituent was well-tolerated in 1a, in 1b, a significant decrease in yield was observed (2b, 37%). The meta-substituent in 1c was tolerated and yielded the anticipated product (2c) with complete regioselectivity. Importantly, the reaction could also be performed using CPME as a solvent with maintained yield, albeit with an increased catalyst loading (6 mol %). Functionalization of 2-phenylpyridine with high monoselectivity was achieved with a decreased MozN_3_ loading (for optimization, see the Supporting Information), yielding compound 2d in 86% yield. The monoselective functionalization observed with pyrazole 1e after the optimization study (see the Supporting Information) translated well to the 0.5 mmol scale. Benzoxazole 2f was successfully obtained under modified conditions based on single-substrate optimization results (see the Supporting Information). The utility of the presented catalytic system beyond the formation of 5-membered iridacycles was demonstrated with the 2g-2i series, yielding the desired products via 6-membered iridacycle formation. The reaction of N-phenylpyrimidin-2-amine yielded a mixture of monoaminated 2g and diaminated 2g′, favoring the monofunctionalization product. Optimization for mono/diselectivity was not conducted for this substrate. In the indole series, both 2h and 2i were obtained with complete selectivity for the 7-position over the 2-position, favoring 6-membered iridacycle formation. Importantly with 2h, the use of more environmentally benign EtOAc as a solvent had no negative effect on yield. To our delight, oxygen-centered directing groups were also successfully utilized as demonstrated with the **2j** – **2m** series. Sulfonimidamides are an emerging class of compounds within medicinal chemistry,^40^ and to the best of our knowledge, the synthesis of **2j** presents the first application of this moiety within directed C–H activation. Functionalization of acetanilide **2k** extends the accessibility of 6-membered iridacyles to oxygen-centered directing groups. Compound **2l** was obtained with improved yield by adding cyclopentane carboxylic acid as an additive.^41^ Improvement of conversion with amide directing groups in combination with carboxylic acid additives was observed during the LSF scope investigation with bezafibrate 3j (Scheme 2). This observation further extended the scope of accessible directing groups with Weinreb amides, as shown with 2m. This substrate class was unproductive under screening conditions of the directing group informer library (Figure 2). While reaction scale-up was vital for further applications, we were also interested in extending the utility of screening libraries by product isolation from small-scale reactions. Ten functionalized building blocks were isolated directly from the DG informer library plate at the 0.02 mmol scale under standard screening conditions, in quantities sufficient for characterization by NMR spectroscopy. The potential utility of small-scale reaction substance isolation extends beyond compound characterization, as a single milligram of compound is often sufficient for in-depth biological studies.^42^ A common setback of this approach is reduced product yields due to sample handling and purification-associated loses, as demonstrated with the decreased yields of **2d** and of **2e** (Scheme 1, top vs bottom). The applicability of heterocyclic DGs was further demonstrated with dihydrooxazole **1n**, thiazole **1o**, benzothiazole **1p**, pyrimidine **1q**, and pyridazine **1r**. The observed mono/diselectivity under standard screening conditions could be further tuned by single-substrate optimization (**2d**, **2e**, and **2q** in the Supporting Information). The PMP-capped imine **2s** was obtained in a low yield as a result of hydrolysis during purification. Products from oxygen-centered directing groups in *N*-acetyl indoline **2t** and *N*-methyl benzamide **2u** were also isolated, the latter with a lower yield due to problematic separation from the unreacted starting material.

### Functional Group Compatibility

In step 3 (Figure 2), the effect of a series of 46 additives on reaction performance was evaluated (Table 1). The additives were chosen as a means of representing functional groups commonly present in drug-like molecules.^30,33,34^ In terms of the solvent effect, only minimal differences in performance between NMP (top bars) and DCE (bottom bars) were observed. To our delight, out of 47 modified conditions, 35 had no effect on the reaction outcome. Notably, excess water was well-tolerated for the catalytic conditions used here, a feature important for the use of reagents without prior drying. In terms of reagent and functional group tolerance, the following insights were gained: (1) The presence of DMSO has a negative effect on the reaction outcome, while other commonly used polar and/or protic solvents are well-tolerated. This observation is in accordance to similar studies.^33,34^ (2) The majority of polar functional groups commonly present in drug-like molecules were well-tolerated. This includes ether, alcohol, phenol, aldehyde, ketone, carboxylic acid, ester, primary and secondary amides, urea, Weinreb amide, and sulfonamides. The same was observed with functional groups commonly present in cross-coupling reagents, such as aliphatic and aromatic halides and the aryl-Bpin group. (3) The utility of the method is limited by the presence of amines: primary, secondary, and tertiary. The presence of aniline leads to a significant decrease in conversion. (4) While heterocycles are in general well-tolerated, the presence of pyridine is detrimental. The activity is restored by sterically hindering the pyridine nitrogen (pyridine vs 2,6-lutidine). (5) Alkenes are tolerated, but the presence of alkynes leads to complete inhibition of the reaction. While this approach provides valuable information on the limitations of this methodology, we recognize that such a simplified approach has its limitations, as the integrity of the additives after the reaction was not determined. Changes in the electronic properties of the additives by substituent variation can also affect compatibility.

**Table 1:**
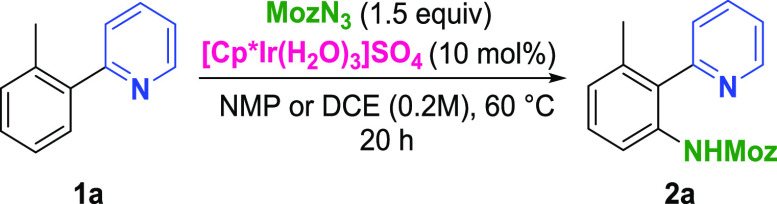

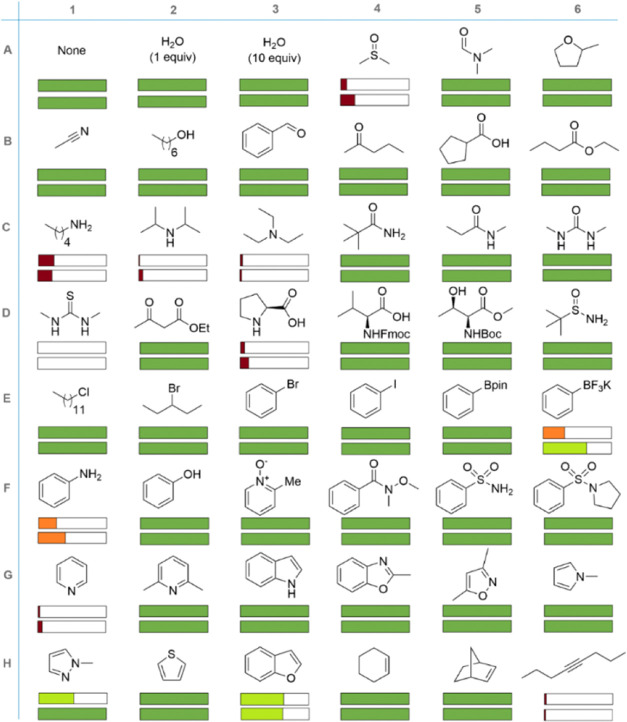
Functional Group Compatibility Study^a^

a Reaction solvents: NMP (top bars), DCE (bottom bars). One equivalent of additive used per reaction. Color coding based on conversion: Green >50%, orange 50–25%, and red < 25%. Analyzed by LCMS (UV trace).

In step 4 (Figure 2), we directed our attention to late-stage amination of a set of complex molecules, consisting of small-molecule drugs and natural products (Scheme 2). The value of the LSF informer library builds on the investigations presented so far, as it introduces further complexity resulting from the interplay of multiple functional groups in a single substrate. A 48-membered LSF informer library was used, this time tested against two solvents: NMP and DCE. Out of these, 11 were considered successful (conversion >10%), with structures confirmed by NMR spectroscopy. Further four compounds were not considered successful due to low conversion and/or product decomposition during purification. The value of performing step 2 (DG informer library) and step 3 (functional group tolerance study) of the envisioned workflow (Figure 2) was shown already at this stage. Out of the 33 remaining compounds, 14 contained unproductive directing groups and 9 contained amines in their structure. The cause behind the failure of the remaining 10 substrates remains unpredicted (for complete library design including unproductive substrates, see the Supporting Information).

Although the reaction conditions for the substrates were mostly based on findings from building block reaction optimizations, single-substrate optimization also proved to be of high utility for LSF examples (see the Supporting Information). In the case of *atazanavir*, single-reaction condition screening allowed us to rapidly identify optimal conditions for accessing the monofunctionalized product **3a** and difunctionalized product **3a′** with a high degree of selectivity in good to very good yields. Further worth noting is the compatibility of the reaction conditions with a number of polar and protic groups, including arrangements of functional groups suitable for bidentate coordination in the peptide backbone. The pyridine moiety also served as a suitable directing group in *pritelivir*. Compound **3b** was successfully obtained, with the primary sulfonamide, thiazole, and tertiary amide groups tolerated. The heavily substituted pyrazole of *apixaban* served as a productive directing group, further demonstrating the utility of this moiety for directed C–H amination. Of important note is the use of NMP as the reaction solvent, as the substrate was insoluble in DCE. While the reaction offered a relatively low isolated yield of **3c**, the majority of the unreacted starting material was successfully isolated. In the case of *sulfaphenazole*, the pyrazole moiety bearing a sulfonamide group in the 5-position served as a suitable directing group, yielding analogue **3d**. Worth noting is the improved tolerance of the aniline compared to the functional group tolerance study (Table 1), presumably due to the different electronic properties of the nitrogen affected by the *para*-sulfonamide group. A case of unexpected selectivity was observed with *telmisartan*, where product **3e**, resulting from the coordination of *N-*methylbenzimidazole, was obtained as a single regioisomer, with no product formation observed from the carboxylate coordination. This is also the only example where we observed the formation of the product with a 1,2,3-substitution pattern. We rationalize the observed selectivity as a result of the steric arrangement of the substrate, with the *ortho*-substituent of the benzoic acid moiety decreasing its reactivity and the fused ring system on the 3-position decreasing steric hindrance and allowing functionalization in a 1,2,3 layout. The imine moiety of *diazepam* facilitated the amination of the phenyl core. Both monofunctionalized **3f** and difunctionalized **3f′** were successfully isolated. It is important to note that the products were isolated directly from the LSF informer library at the 0.02 mmol scale, in quantities sufficient for complete characterization. *Lumacaftor* was successfully functionalized in NMP with complete selectivity for the carboxylate-directed amination. With relatively low conversion, the 2-acylanilido pyridine structural motif was tolerated. A significant amount of unreacted starting material was also recovered. The product of carboxylate-directed C–H amination of *repaglinide***3h** was successfully isolated on the 0.02 mmol screening scale. The tolerance of the tertiary amine moiety is rationalized by its anilinic nature.

A powerful example of the utility of the herein-described amination protocol is demonstrated with the functionalization of *paclitaxel*. This complex natural product contains a number polar and protic functional groups, sensitive ester groups, a strained oxetane ring, and unsaturation, all of which pose a potential challenge for LSF methods. While **3i** could be obtained with 20% isolated yield under standard conditions, the use of one equivalent of acid additive allowed for increasing the isolated yield to 72%. This result, to the best of our knowledge, presents the highest yielding example of *paclitaxel* C–H functionalization reported to date. The positive effect of carboxylic acid additives on conversions with substrates bearing amide directing groups was first observed with the example of *bezafibrate*. The conversion to **3j** was much higher than conversions of the corresponding amides in the DG informer library. This unexpected observation further strengthens the case for LSF informer libraries, as the combinations of structural motifs directly aided in methodology development. Finally, *levamisole*, bearing an unusual sp^2^-sp^3^ linkage between the benzene core and the directing group, was selectively difunctionalized to yield product **3k**. A point worth noting is that even though the isolated yields for a number of presented examples were relatively low, in many cases, these may still be comparable to or exceeding the expected overall yields from *de novo* synthesis of these analogues. The amount of the material obtained from these reactions would suffice the needs of biological studies, allowing for rapid access to SAR data in a fraction of time compared to *de novo* synthesis.

### Deprotection Studies

**Scheme 3:**
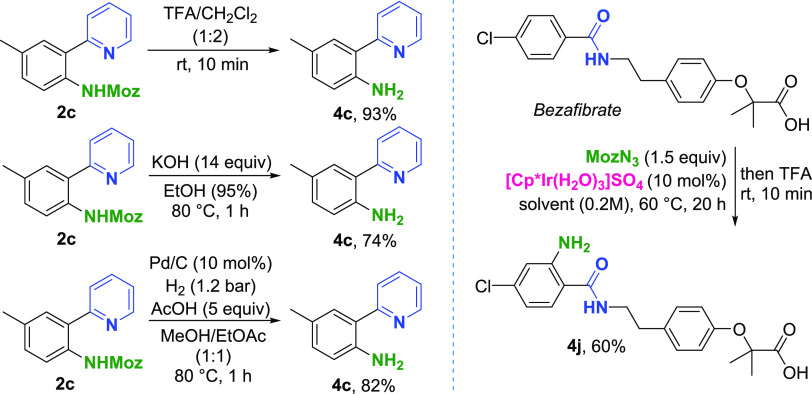
Deprotection Studies^,^ Isolated yields shown. Left: Deprotection of the isolated material. Right: One-pot amination/deprotection. Solvent = DCE. A higher isolated yield of **4j** was obtained compared to the Moz-protected **3j** as a result of better separation by HPLC for the former compound.

The Moz group was successfully deprotected with three distinct deprotection protocols (Scheme 3). This is of particular importance in terms of LSF applications, allowing for deprotection conditions tolerant toward a wide array of functional groups. Deprotection under acidic conditions in the presence of TFA yielded the corresponding aniline in excellent yields. The desired compound was also obtained under basic conditions, using excess KOH in refluxing EtOH. In the third protocol, hydrogenolysis using standard Pd/C hydrogenation yielded the **4c** product in a very good yield. Finally, LSF application of a one-pot amination/deprotection protocol was demonstrated with *bezafibrate*, yielding the free aniline product **4j** in 60% isolated yield.

### Miniaturization Studies

**Figure 4:**
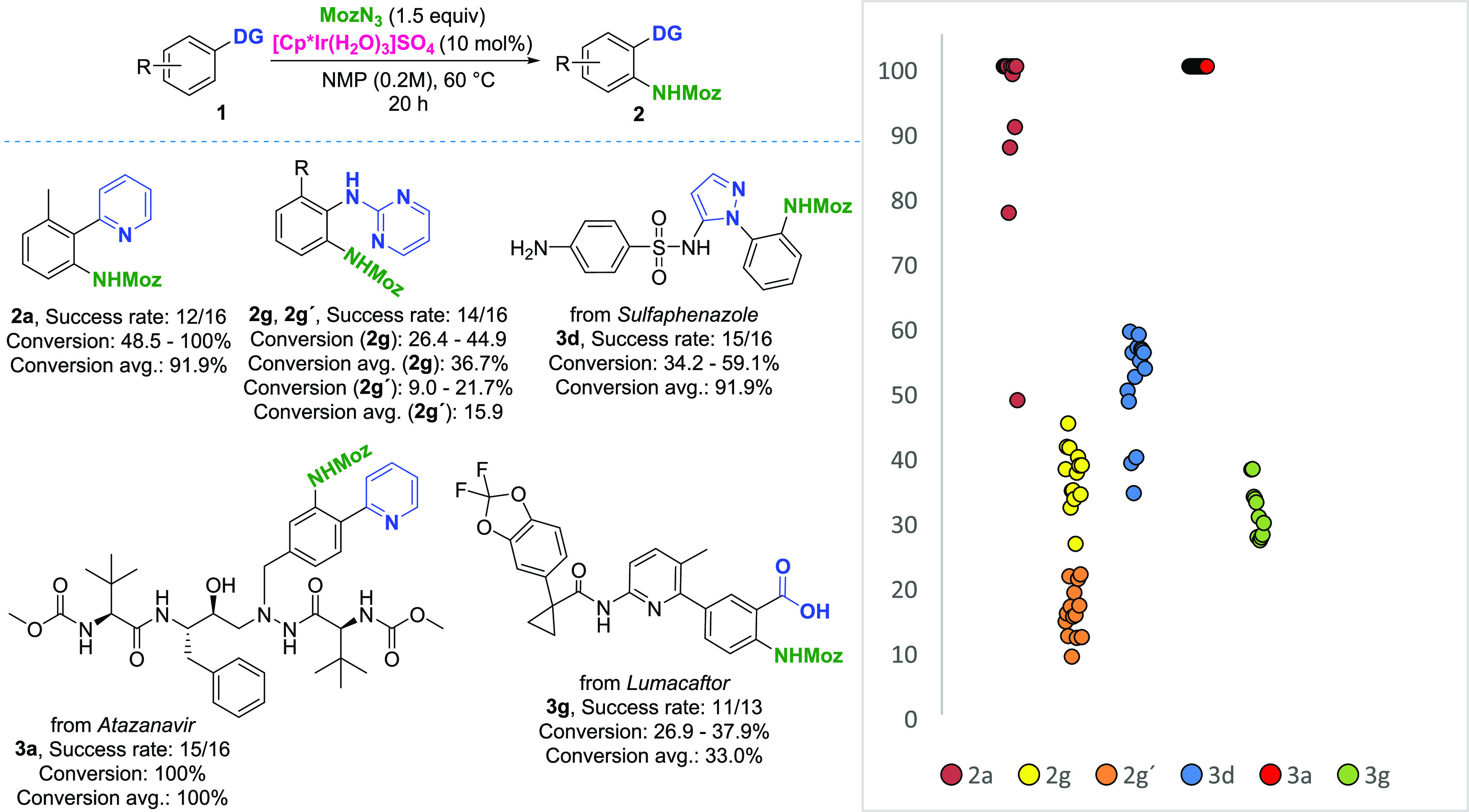
Miniaturization study. Reaction scale: 0.2 μmol, reaction volume: 1.0 μL. Analyzed by LCMS (UV trace). With an unsuccessful reaction, no substrate or product was detected.

In the final experimental study, we further investigated the possibilities in reaction miniaturization enabled by the use of NMP as a nonvolatile solvent. We found that with as little as one microliter of total reaction volume, conversion to the anticipated products with reasonable reproducibility was obtained throughout the selected substrates (Figure 4). This presents an exciting opportunity in terms of improved sustainability of reaction screening by decreased material consumption. An important consideration for such small-scale application is the reaction success rate, with unsuccessful reactions arising from pipetting errors. We were even able to further scale down the reaction using acoustic dispensing for setting up and analyzing the reaction plates.^43,44^ With this technique, we were able to detect product formations in reaction with a total volume as little as 5 nL (1 nmol scale). Taking *lumacaftor* as an example, this means that from one milligram of material, 2210 reactions can be performed.

### Application Guidelines

**Figure 5:**
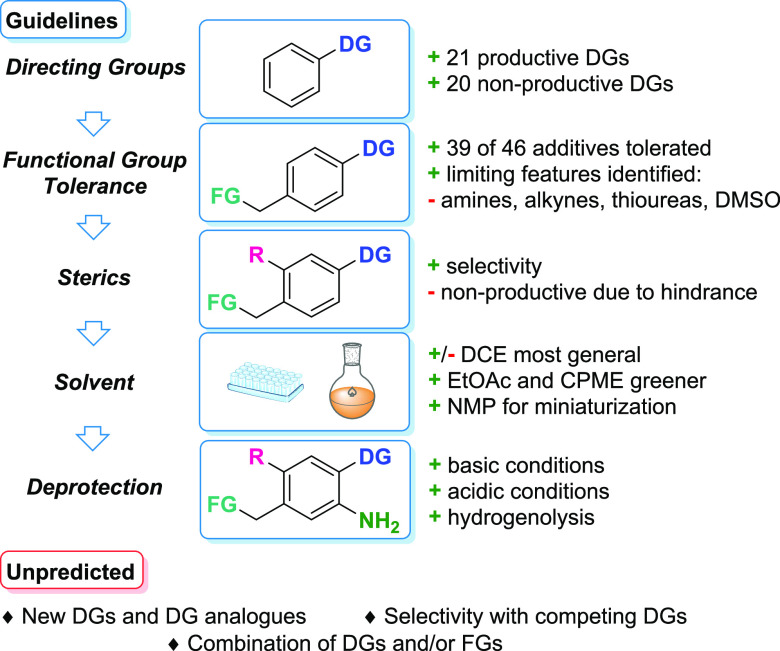
Application guidelines for the C–H amination methodology.

In the final part of this work, step 5 (Figure 2), we present guidelines for reaction outcome prediction (Figure 5).

(1) Directing group selection. In total, 21 productive directing groups were presented in the DG and LSF informer libraries along with 20 nonproductive directing groups from the DG informer library (Scheme 1, for complete substrate structures, see the Supporting Information).
(2) Determination of tolerated functional groups. This is aided by the functional group tolerance study and LSF scope. The major limitation in this respect is the presence of amines, alkynes, thioureas, and residual DMSO. The DMSO sensitivity is important to consider in medicinal chemistry applications, as intermediates are often stored as DMSO solutions and the residual solvent can be present after evaporation.
(3) Steric effects on the substrate should be examined to determine selectivity and/or productivity. Based on the observed results, the reaction proceeds on the less-sterically hindered *ortho* position when two suitable reaction sites are available. *Meta*-substitution with sterically demanding substituents blocks 1,2,3-substitution. Substituents on the directing group and in the *ortho* position of the system to be functionalized can negatively affect the reaction outcome by twisting the directing group out of plane.^45^
(4) The reaction solvent is chosen based on the desired application. While DCE showed the best overall performance, substitution of EtOAc and CPME is possible if the use of a greener solvent is desired. The use of NMP is ideal for applications in miniaturization and for applications with polar drug-like molecules displaying low solubility in DCE.
(5) The final step is the choice of deprotection conditions. The range of the presented protocols should satisfy the needs of potential applications in LSF.

Although we studied the reaction extensively, the reaction application guidelines have their limitations. Given the breadth of the chemical space of Lewis basic DGs, it is likely that some DGs and their analogues remain uninvestigated. Selectivity between DGs, while observed in most cases, was not extensively investigated. Finally, while the functional group tolerance study provides basic guidelines, the combination effect of functional groups or even combination effects with DGs are not possible to predict.

## Conclusions

A directed iridium-catalyzed C–H amination methodology applicable to substrates with a wide range of directing groups and outstanding functional group tolerance was developed. HTE applications not only facilitated rapid optimization of reaction conditions but also allowed for reaction miniaturization to the nanomolar scale and use of automation throughout the campaign. An important aspect of this study was exploring both the opportunities and limitations of the reaction, disclosing both successful and unsuccessful reactions. The directing group and LSF informer libraries, in combination with the functional group tolerance studies, allowed for the generation of guidelines for predicting reaction applicability to complex substrates. In terms of the demonstrated substrate scope, a broad range of building blocks with diverse directing groups were synthesized, and late-stage functionalization of a number of structurally complex drugs and natural products was demonstrated. The utility of the presented method for applications on complex substrates is further increased by access to a range of Moz deprotection protocols. We are confident that the presented reaction and associated methods and techniques will find applications in other laboratories. Finally, it is our sincere hope that this work will inspire others to disclose the limitation of their methodologies, allowing users to save time and effort on unproductive reactions, but also eliminating material consumption for such reactions and ultimately reducing the environmental impact of synthetic chemistry.

## Supporting Information Available

Information on the HTE reaction setup and analytics; experimental procedures; compilation of DG and LSF informer libraries with conversions, including unproductive substrates; ^1^H, ^13^C NMR, and HRMS of final products, 2D NMR in selected cases (PDF)
