# Supplementary material for: Merging Directed C–H Activations with High-Throughput Experimentation: Development of Iridium-Catalyzed C–H Aminations Applicable to Late-Stage Functionalization
Source: JACS Au. 2022 Apr 13;2(4):906–16. doi: 10.1021/jacsau.2c00039 (PMC9088304; doi:10.1021/jacsau.2c00039)
Supplement: Supplementary file 1 — au2c00039_si_001.pdf [file au2c00039_si_001.pdf]

Supporting Information

Merging Directed C–H Activations with High-throughput Experimentation: Development of Iridium-catalyzed C–H Aminations Applicable to Late-stage Functionalization

Erik Weis,[a,b] Maria Johansson,[c] Pernilla Korsgren,[c] Belén Martín-Matute,*[a] Magnus J. Johansson*[a,b]

[a] Department of Organic Chemistry, Stockholm University, Stockholm SE-106 91, Sweden,
[b] Medicinal Chemistry, Research and Early Development; Cardiovascular, Renal and Metabolism, Biopharmaceuticals R&D, AstraZeneca,Gothenburg, Pepparedsleden 1, 431 50 Mölndal, Sweden
[c] Compound Synthesis and Management, Discovery Sciences, BioPharmaceuticals R&D, AstraZeneca, Gothenburg, Pepparedsleden 1, 431 50 Mölndal, Sweden
* Correspondence to: belen.martin.matute@su.se (B.M.-M.) Magnus.J.Johansson2@astrazeneca.com (M.J.J.)

Contents

General Information 3
Reagent information 3
Screening and HTE information: Reaction set-up and analysis (5 µmol - 0.02 mmol scale) 3
Screening and HTE information: Purification (0.02 mmol scale) 4
Purification information: Scale up (0.1 - 0.5 mmol scale) 5
Analytical information 5
Safety considerations 5
Catalyst preparation 5
Reaction optimization 6
Initial optimization 6
Single substrate reoptimizations 10
Single substrate optimization (**2j**) 10
Single substrate optimization (**2f**) 11
Single substrate optimization (**2q**) 11
Single substrate optimization (**2e**) 12
Single substrate optimization (**2d**) 12
Single substrate optimization (**2k** and **2u**) 13
Single substrate optimization (**3a** and **3a´**) 14
Single substrate optimization (**3e**) 14
Functional group tolerance study 15
Directing group informer library 17
LSF informer library 19
General experimental procedures 22
NMR Spectra 44
References 100

General Information

Reagent information

Unless otherwise noted, all reagents and solvents were used as received. The $[Cp^*IrCl_2]_2$ catalyst precursor for the preparation of $[Cp^*Ir(H_2O)_3]SO_4$ was purchased from Strem Chemicals. Et_3N was purchased from Sigma Aldrich. 4-Methoxybenzyloxycarbonyl azide ($MozN_3$) was purchased from Sigma Aldrich. All reactions were performed under air atmosphere with no exclusion of moisture. All reagents were used as received from vendors. The SiliaMetS® Imidazole (Si-IMI) metal scavenger from SIliCycle was used.

Screening and HTE information: Reaction set-up and analysis (1.0 nmol – 8.0 nmol scale)

Reagents were dispensed by acoustic dispensing using the Labcyte Echo® liquid dispensing system. For the reaction set-up Labcyte Echo® qualified 1536-well low dead volume plates were used. As source plates and analytical plates Labcyte Echo® qualified 384-well plates were used. LCMS analysis (MS only, no UV) was carried out on a Waters Acquity UPLC system, on a BEH C18 column (basic method: A: H2O/MeCN/NH_3 = 95/5/0.2, B: MeCN). MS analysis was also performed on an AMI-MS system as previously described.[1]

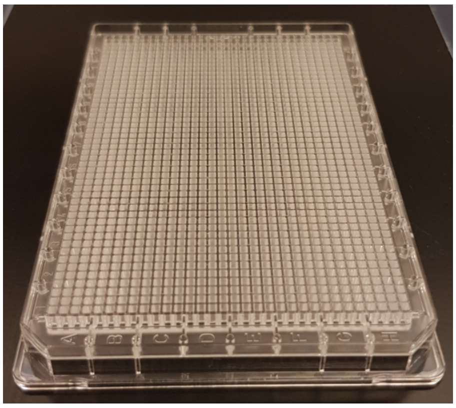

1536-well plate used at 1.0-8.0 nmol scale.

Screening and HTE information: Reaction set-up and analysis (0.2 µmol - 0.8 µmol scale)

For liquid dispensing the Mosquito® LV system from SPT Labtech was used. For reaction set-up 384-well Plate+™ glass-coated microplates from Thermo Fisher Scientific were used. For analysis standard 384-well microplates were used. LCMS analysis was carried out on a Waters Acquity UPLC system, on a BEH C18 column (basic method: A: H2O/MeCN/NH_3 = 95/5/0.2, B: MeCN).

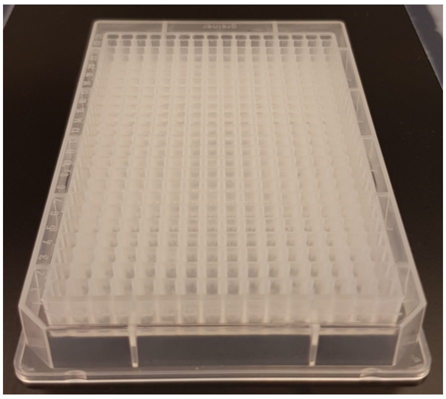

384-well plate used at 0.2-0.8 µmol scale.

Screening and HTE information: Reaction set-up and analysis (5 µmol - 0.02 mmol scale)

Solids were weighed in manually or using the Quantos powder dosing system from Mettler Toledo. For liquid dispensing manual pipettes and the TECAN freedom evo liquid handling platform was used. For solvent removal the SP Scientific Genevac HT-6 evaporation system was used. The reactions were set-up in a Para-Dox® 24- or 96-position parallel synthesis plate using 50 µL or 1 mL vials. LCMS analysis was carried out on a Waters Acquity UPLC system, on a BEH C18 column (basic method: A: H2O/MeCN/NH_3 = 95/5/0.2, B: MeCN).

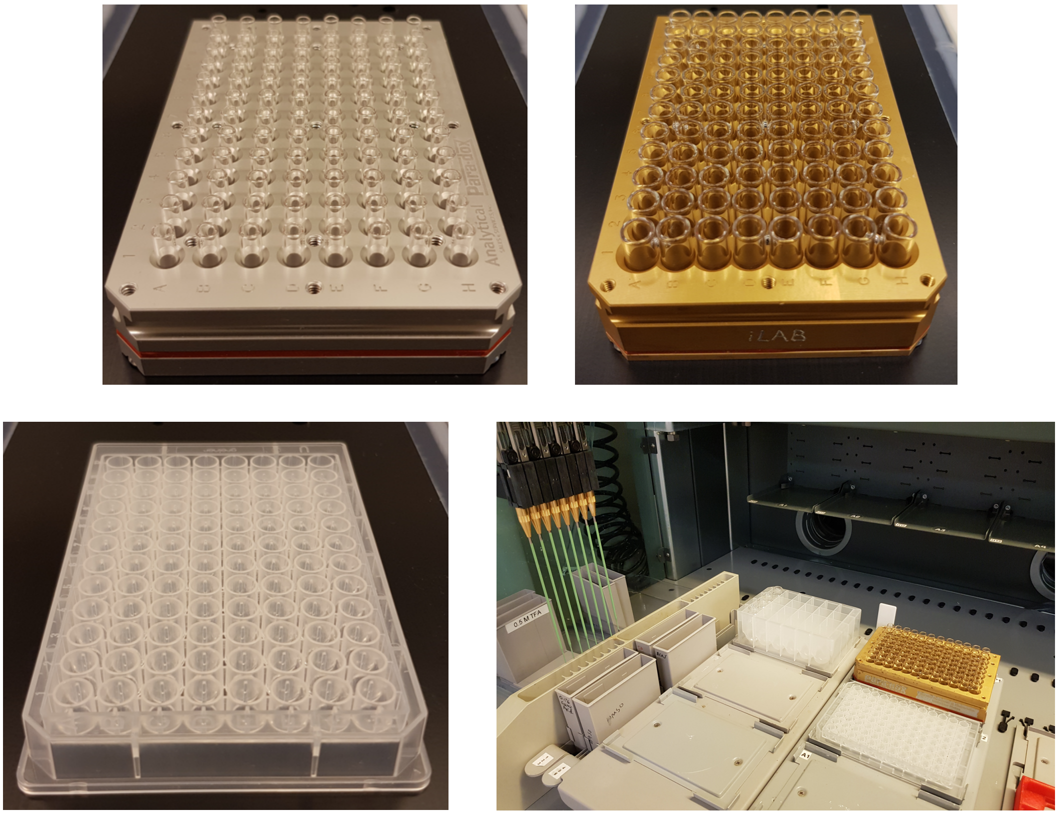

Top: Left: 96-well plate used at 5 µmol scale. Right: 96-well plate used at 0.02 mmol scale.
Bottom: Left: 96-well analytical plate. Right: Analytical plate set-up using the Tecan Freedom EVO® liquid handling system.

Screening and HTE information: Purification (0.02 mmol scale)

Pre-purification analysis of the crude samples was performed on a Waters Acquity System with a Waters QDA Mass Spectrometer using three different columns to evaluate the best method for preparative separation: Waters Acquity UPLC HSS C18 1.8 µm (2.1 x 50 mm) and Waters Acquity UPLC CSH Fluoro-Phenyl 1.7 µm (2.1 x 50 mm) at acidic conditions and Waters Acquity UPLC BEH C18 1.7 µm (2.1 x 50 mm) at basic conditions. A gradient of 2-94% MeCN (10 mM formic acid or 0.2% ammonia) was used with a flow 0.8 mL/min for 2.5 min at 45°C.

Preparative LC was performed on a Waters Fraction Lynx system with a Waters QDA Mass Spectrometer and a 2767 autosampler. The columns used were either Waters SunFire C18 OBD 5 µm (10 x 100 mm) or Waters XSelect CSH Fluoro Phenyl OBD 5 µm (10 x 100 mm) at acidic conditions or Waters XBridge BEH C18 OBD 5 µm (10 x 100 mm) at basic conditions. A focused 50 % MeCN gradient (0.1 M formic acid or 0.2% ammonia) was used with a flow of 8.3 ml/min in 5.2 min at ambient temperature. Fraction collection was triggered using a combination of UV and MS.

The purity of the isolated sample was determined using a Waters Acquity System with a Waters QDA Mass Spectrometer at both acidic and basic conditions, using a Waters Acquity UPLC HSS C18 1.8 µm (2.1 x 50 mm) at low pH and Waters Acquity UPLC BEH C18 1.7 µm (2.1 x 50 mm) at high pH. A

gradient of 5-94% MeCN (10 mM formic acid or 47 mM NH_3/ 6.5 mM NH_4HCO_3) was used with a flow 0.8 mL/min for 1.5 min at 50°C.

Purification information: Scale up (0.1 - 0.5 mmol scale)

Flash column chromatography purifications were performed on Biotage ISOLUTE SI 25 g pre-loaded columns, unless otherwise stated. Biotage® Sfär-samplet® (2.5g) were used for sample preparation for flash column chromatography, unless otherwise stated. For HPLC purification of scale-up compounds (0.1 – 0.5 mmol scale), preparative reverse-phase HPLC on a Kromasil C8 column (10 µm, 250x50 ID mm), using gradient elution (acidic method: A: H2O/MeCN/FA = 95/5/0.2, B: MeCN; or basic method: A: H2O/MeCN/NH_3 = 95/5/0.2, B: MeCN) was used. The reaction mixtures purified by HPLC were treated with a metal scavenger, SiliaMetS® Imidazole from Silicycle prior to purification. Gilson GX-281 liquid handler/autosampler was used. UV detector Gilson UV/VIS-155 was used for UV-triggered collection of fractions at 254 nm wavelength.

Analytical information

LCMS analysis was carried out on a Waters Acquity UPLC system, on a BEH or HSS C18 column. For SFC-MS analysis a Waters Acquity UPC2 SFC-MS system with a BEH column was used. Conversion quantification in the optimization campaign, informer libraries and functional group tolerance studies were based on UV trace as a ratio of SM:P (starting material : product). Nuclear magnetic resonance spectra (^{1}H, ^{13}C, ^{19}F, COSY, HSQC, HMBC) were recorded on Bruker ULTRASHIELD 500 and 600 MHz spectrometer with a Bruker CRYO PLATFORM. ^{1}H NMR spectra were referenced to CD_3OD (3.31 ppm), DMSO-d_6 (2.50) and $CDCl_3$ (7.26). ^{13}C NMR spectra were recorded at 126 and 151 MHz, referenced to in CD_3OD (49.00 ppm), DMSO-d_6 (39.52) and $CDCl_3$ (77.16 ppm). Coupling constant (J) values were measured in Hertz (Hz) and chemical shift (δ) values in parts per million (ppm). HRMS data was recorded on a Waters Acquity System with a XEVO-QTOF MS spectrometer at either acidic or basic conditions, using Leucine Enkephaline (C28H37N5O7, m/z 556.2771) as lock mass. ESI ionization in positive or negative mode. A gradient of 5-90% MeCN (10 mM formic acid or 47 mM NH_3/ 6.5 mM NH_4HCO_3) was run with a flow of 0.8 mL/min for 2.5 min at 45°C, using a Waters Acquity UPLC HSS C18 1.8 µm (2.1 x 50 mm) at low pH and Waters Acquity UPLC BEH C18 1.7 µm (2.1 x 50 mm) at high pH.

Safety considerations

Organic azides are known high energy compounds and special precautions need to be taken when used. As N_2 gas is formed as a side-product in the reaction, measures to mitigate the pressure build-up should be taken. Keeping a 1:3 reaction volume to head space is recommended.

Catalyst preparation

The $[Cp^*Ir(H_2O)_3]SO_4$ catalyst was prepared according to published procedure.[2]
Ag_2SO_4 (2.52 mmol, 786 mg) was added to a suspension of $[Cp^*IrCl_2]_2$ (1.255 mmol, 1.00 g) in deionized H_2O (8.0 mL). The mixture was stirred at room temperature for 16 h. The contents were then transferred into a Corning® 15 mL centrifuge tube and centrifuged at 2000rpm for 1 minute to sediment the silver salts. The supernatant was removed and kept. The cake was washed with water (8 mL), centrifuged and filtrate collected two times. The combined aqueous phases were concentrated in vacuo to yield the product $[Cp^*Ir(H_2O)_3]SO_4$ as a yellow solid (1.15 g, 94%).

Reaction optimization

Initial optimization

Screening 1

The initial reaction conditions were based on previously published method.[3] The commercially available $MozN_3$ was chosen as nitrogen source. Low solubility of the NaOAc and $AgNTf_2$ additives was noted according to the conditions of entry 1. Highest conversion was observed according to conditions of entry 1. Conditions of entry 8 were chosen for further optimization, as lower reaction concentrations were sought after to facilitate the use of stock solutions.

Reaction scale 0.05 mmol. The reactions were set up in a 24-well Para-dox block, vials equipped with stirrer bars. The solid additives and catalysts were weighed in manually. *N*-(*tert*-butyl)benzamide (8.9 mg, 0.05 mmol) and $MozN_3$ (15.5mg, 0.075 mmol) were added, followed by addition of the reaction solvent. The reaction mixtures were stirred at 500 rpm and heated at 60 °C for 20h. After this DMSO (200 µL) and metal scavenger were added to the vials and stirred for additional 2 hours at room temperature. Analyzed by LCMS.

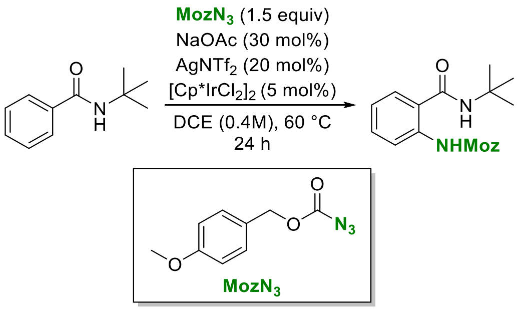

Entry	Deviation	Conversion (%)
1[3]	None	42
2	[Cp*Ir(H2O)3]SO4 (10 mol%), no $AgNTf_2$	65
3	[Cp*Ir(H2O)3]SO4 (5 mol%), no $AgNTf_2$	31
4	[Cp*Ir(H2O)3]SO4 (10 mol%), no $AgNTf_2$, $MozN_3$ (2.5 equiv)	56
5	[Cp*Ir(H2O)3]SO4 (10 mol%), no $AgNTf_2$, room temperature	0
6	[Cp*Ir(H2O)3]SO4 (10 mol%), no $AgNTf_2$, room temperature, KOAc (0.3 equiv.)	0
7	[Cp*Ir(H2O)3]SO4 (10 mol%), no $AgNTf_2$, room temperature, nBu_4OAc (0.3 equiv.)	0
**8**	[Cp*Ir(H2O)3]SO4 (10 mol%), no $AgNTf_2$, 0.2M concentration	**55**

Conversion determined by LCMS (UV trace). Entry chosen for next round of optimization highlighted in green. Entry 1 based on published literature procedure.[3]

Control experiments

A series of control experiments was carried out to prove the observed amination is a result of iridium catalysis. No conversion was observed in absence of $[Cp^*Ir(H_2O)_3]SO_4$ (Entries a-c). similarly, no conversion was observed in presence of Ag_2SO_4.

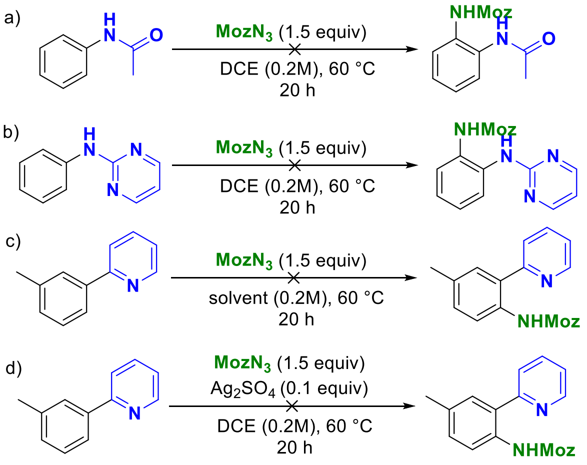

No conversion observed. Determined by LCMS.

The reactions were set up in a 24-well Para-dox gold block equipped with stirrer bars. Reaction scale 0.02 mmol. To each respective vial, the solid reagents, *N*-phenylacetamide (2.7 mg, 0.02 mmol), *N*-phenylpyrimidin-2-amine (3.4 mg, 0.02 mmol), $MozN_3$ (6.2 mg, 0.03 mmol) and Ag_2SO_4 (0.6 mg, 2 µmol) were weighed in manually. DCE (100 µL) was added, followed by addition of 2-(m-tolyl)pyridine (3.4 µL, 0.02 mmol) to respective vials. The reaction mixtures were stirred at 500 rpm and heated at 60 °C for 20h. After this DMSO (200 µL) and metal scavenger were added to the vials and stirred for additional 2 hours at room temperature. Analyzed by LCMS.

Screening 2

Screening against 2 substrates bearing different directing groups. Further miniaturization was also tested, with the reactions on a 5 µmol scale, 25 µL total volume. Better results obtained with $[Cp^*Ir(H_2O)_3]SO_4$. Signifficant decrease in conversion observed with $[Cp^*IrCl_2]_2$ in absence of $AgNTf_2$. Best results obtained with $[Cp^*Ir(H_2O)_3]SO_4$ in absence of any additives.

The reactions were set up in a 96-well Para-dox silver block equipped with stirrer bars. Reaction scale 5 µmol. $[Cp^*Ir(H_2O)_3]SO_4$ (0.05M, 10 µL, 0.5 µmol) , NaOAc (0.05M), LiOAc (0.05M) and KOAc (0.05M) were added as solutions in deionized water. $[Cp^*IrCl_2]_2$ (0.025M, 10 µL) was added as stock solution in MeOH. The solvents were then removed using a Genevac HT6 centrifuge evaporator. The substrates and $MozN_3$ were added as DCE solutions (0.2 M, 25 µL). The reaction mixtures were stirred at 500 rpm and heated at 60 °C for 20h. After this DMSO (20 µL) and metal scavenger were added to the vials and stirred for additional 2 hours at room temperature. Analyzed by LCMS.

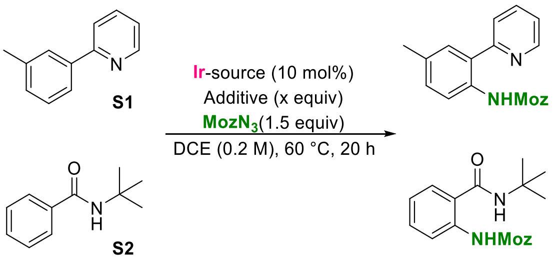

Substrate	$[Cp^*Ir(H_2O)_3]SO_4$		$[Cp^*IrCl_2]_2$	
	S1	S2	S1	S2
No Additive	100	27	3	0
NaOAc (0.3 equiv)	57	21	36	0
LiOAc (0.3 equiv)	93	11	11	0
KOAc (0.3 equiv)	62	4	9	0
NaOAc (1.0 equiv)	99	2	5	0
LiOAc (1.0 equiv)	97	11	15	0
KOAc (1.0 equiv)	94	8	2	0
	Conversion (%)		Conversion (%)	

Conversion determined by LCMS (UV trace). Entry chosen for next round of optimization highlighted in green.

Screening 3

The reactions were set up in a 96-well Para-dox gold block equipped with stirrer bars. Reaction scale 0.02 mmol. $[Cp^*Ir(H_2O)_3]SO_4$ (0.05M, 40 µL, 2 µmol) and NaOAc (0.1M, 60 µL, 6 µmol) were added as solutions in deionized water. The solvent was then removed using a Genevac HT6 centrifuge evaporator. *N*-(*tert*-butyl)benzamide (**S2**, 3.5 mg, 0.02 mmol) was added as solid using the Quantos powder dosing system. $MozN_3$ (6.2 mg, 0.03 mmol) was weighed in manually. The respective solvents (100 µL) were added, followed by the addition of 2-(3-methylphenyl)pyridine (**S1**, 3.4 µL, 0.02 mmol). The reaction mixtures were stirred at 500 rpm and heated at 60 °C for 20h. After this DMSO (200 µL) and metal scavenger were added to the vials and stirred for additional 2 hours at room temperature. Analyzed by LCMS.

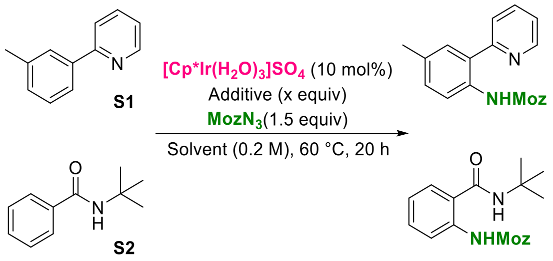

Substrate		NMP	IPA	2MeTHF	EtOAc	Acetone	DCE	Toluene	CPME
S1	No Additive	100	100	100	100	100	100	86	100
	NaOAc (0.3 equiv)	0	7	0	0	0	3	0	0
S2	No Additive	0	12	12	16	11	21	19	21
	NaOAc (0.3 equiv)	0	0	0	0	0	0	0	0
		Conversion (%)							

Conversion determined by LCMS (UV trace). Entry chosen for next round of optimization highlighted in green.

Catalyst loading and solvent effect study

The reactions were set up in a 96-well Para-dox gold block equipped with stirrer bars. Reaction scale 0.02 mmol. $[Cp^*Ir(H_2O)_3]SO_4$ (0.05M) was added as solutions in deionized water. The solvent was then removed using a Genevac HT6 centrifuge evaporator. *N*-(2-Pyrimidyl)indole (3.9 mg, 0.02 mmol) was added as solid using the Quantos powder dosing system. To the same vials $MozN_3$ (0.3M, 100 µL, 0.03 mmol) was added in respective solvents. 2-(3-methylphenyl)pyridine (0.02 mmol) and $MozN_3$ (0.03 mmol) were added together as stock solution (100 µL per vial) in respective solvents. The reaction mixtures were stirred at 500 rpm and heated at 60 °C for 20h. After this DMSO (200 µL) and metal scavenger were added to the vials and stirred for additional 2 hours at room temperature. Analyzed by LCMS.

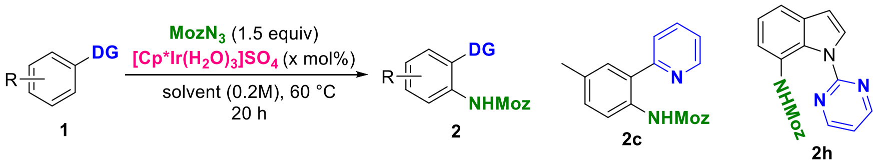

Substrate	2c				2h			
Solvent	NMP	DCE	EtOAc	CPME	NMP	DCE	EtOAc	CPME
10	90.9	99.1	99.5	99.5	100	100	100	93.6
8	92.7	99.2	100	99.4	98.0	100	100	83.9
6	88.1	99.1	64.4	98.6	87.0	100	96.0	66.3
4	57.4	99.5	25.1	65.4	38.9	100	81.6	35.2
2	13.1	83.2	11.1	21.8	6.9	55.0	54.7	7.6
Ir (mol%)								

Conversion determined by LCMS (UV trace). Color gradient: lowest → highest conversion, red → green.

$MozN_3$ and catalyst loading effect study

The reactions were set up in a 96-well Para-dox gold block equipped with stirrer bars. Reaction scale 0.02 mmol. $[Cp^*Ir(H_2O)_3]SO_4$ (0.05M) was added as solutions in deionized water. The solvent was then removed using a Genevac HT6 centrifuge evaporator. The substrates were weighed in manually. *N*-(2-Pyrimidyl)indole (3.9 mg, 0.02 mmol) was added as solid using the Quantos powder dosing system. To the same vials $MozN_3$ (0.3M, 100 µL, 0.03 mmol) was added in respective solvents. 2-(3-methylphenyl)pyridine (0.02 mmol) and $MozN_3$ (0.03 mmol) were added together as stock solution (100 µL per vial) in respective solvents. The reaction mixtures were stirred at 500 rpm and heated at 60 °C for 20h. After this DMSO (200 µL) and metal scavenger were added to the vials and stirred for additional 2 hours at room temperature. Analyzed by LCMS.

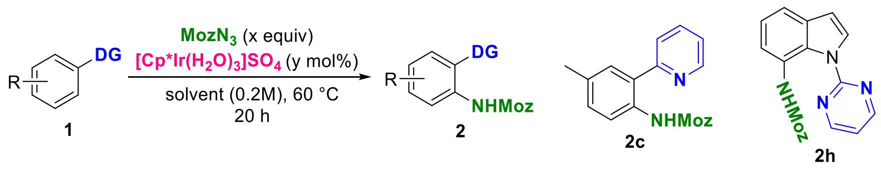

Product	2c						2h					
Solvent	DCE			NMP*			DCE			NMP		
Ir (mol%)	2	4	6	4	6	8	2	4	6	4	6	8
$MozN_3$ (equiv) 1.0	77.9	84.7	83.5	53.1	88.0	89.0	63.6	86.7	85.7	20.1	62.3	94.9
1.1	75.1	91.6	92.3	47.7	94.0	95.1	73.9	93.3	97.1	60.1	90.2	89.8
1.3	80.1	100	100	46.6	88.4	94.1	71.0	100	100	60.7	90.0	90.7
1.5	71.0	100	100	53.8	85.8	90.9	66.3	100	100	42.1	87.7	90.5
2.0	57.2	98.0	100	47.4	70.5	88.0	58.6	100	100	20.7	58.2	87.3
3.0	53.4	86.6	100	44.9	68.6	86.2	42.6	91.4	100	25.0	60.3	82.4

Conversion determined by LCMS (UV trace). Color gradient: lowest → highest conversion, red → green.

*Overall lower yield for the desired product from the functionalization of 2-(3-methylphenyl)pyridine were obtained in NMP due to regioisomer formation, as well as difunctionalization (see below).

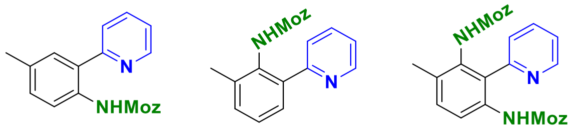

NMP

Ir (mol%)	4			6			8		
	Mono 1	Mono 2	Di	Mono 1	Mono 2	Di	Mono 1	Mono 2	Di
$MozN_3$ (equiv) 1.0	53.1	1.6	2.2	88.0	0.3	4.7	89.0	0.3	3.3
1.1	47.7	2.2	2.0	94.0	0.0	5.3	95.1	0.0	4.3
1.3	46.6	2.3	2.1	88.4	0.0	6.0	94.1	0.2	5.3
1.5	53.8	2.1	2.8	85.8	0.5	6.2	90.9	0.4	8.0
2.0	47.4	2.4	2.5	70.5	1.9	4.9	88.0	0.4	7.3
3.0	44.9	2.6	2.3	68.6	2.1	5.0	86.2	0.4	7.9

Conversion determined by LCMS (UV trace). Color gradient: lowest → highest conversion, red → green.

Single substrate reoptimizations

Single substrate optimization (2j)

For a series of substrates catalyst loading and $MozN_3$ loading were optimized.

The reactions were set up in a 24- or 96-well Para-dox gold block equipped with stirrer bars. Reaction scale 0.02 mmol. $[Cp^*Ir(H_2O)_3]SO_4$ (0.05M) was added as solutions in deionized water. The solvent was then removed using a Genevac HT6 centrifuge evaporator. The substrate (0.02 mmol) and $MozN_3$ (6.2 mg, 0.03 mmol) were together added as stock solutions in DCE (100 µL). The reaction mixtures were stirred at 500 rpm and heated at 60 °C for 20h. After this DMSO (200 µL) and metal scavenger were added to the vials and stirred for additional 2 hours at room temperature. Analyzed by LCMS.

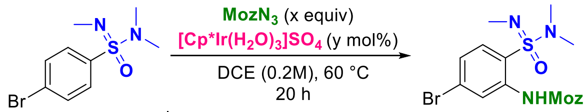

Ir (mol%)		2	4	6
MozN3 (equiv)	1.0	17.7	35.8	51.4
	1.1	20.0	35.7	51.2
	1.3	17.9	35.7	49.6
	1.5	17.6	35.3	48.0
	2.0	16.6	33.6	47.3

Conversion determined by LCMS (UV trace). Color gradient: lowest → highest conversion, red → green.

Single substrate optimization (2f)

The reactions were set up in a 96-well Para-dox gold block equipped with stirrer bars. Reaction scale 0.02 mmol. $[Cp^*Ir(H_2O)_3]SO_4$ (0.05M) was added as solutions in deionized water. The solvent was then removed using a Genevac HT6 centrifuge evaporator. The substrate (0.02 mmol) and $MozN_3$ (6.2 mg, 0.03 mmol) were together added as stock solutions in DCE (100 µL). The reaction mixtures were stirred at 500 rpm and heated at 60 °C for 20h. After this DMSO (200 µL) and metal scavenger were added to the vials and stirred for additional 2 hours at room temperature. Analyzed by LCMS.

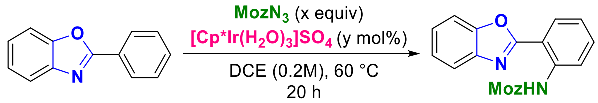

Ir (mol%)		2		4		6	
		Mono	Di	Mono	Di	Mono	Di
MozN3 (equiv)	1.0	21.5	0.0	47.7	0.7	61.5	1.1
	1.1	21.3	0.0	46.4	0.3	62.1	0.6
	1.3	17.2	0.0	41.3	0.3	57.6	0.6
	1.5	18.1	0.0	39.8	0.2	55.3	0.5
	2.0	12.2	0.0	32.0	0.2	48.9	0.4

Conversion determined by LCMS (UV trace). Color gradient: lowest → highest conversion, red → green.

Single substrate optimization (2q)

The reactions were set up in a 96-well Para-dox gold block equipped with stirrer bars. Reaction scale 0.02 mmol. $[Cp^*Ir(H_2O)_3]SO_4$ (0.05M) was added as solutions in deionized water. The solvent was then removed using a Genevac HT6 centrifuge evaporator. The substrate (0.02 mmol) and $MozN_3$ (6.2 mg, 0.03 mmol) were together added as stock solutions in DCE (100 µL). The reaction mixtures were stirred at 500 rpm and heated at 60 °C for 20h. After this DMSO (200 µL) and metal scavenger were added to the vials and stirred for additional 2 hours at room temperature. Analyzed by LCMS.

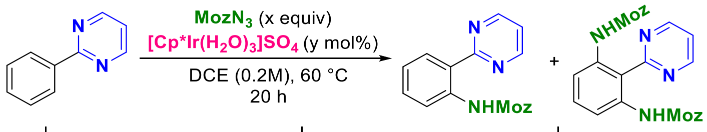

Ir (mol%)		2		4		6	
		Mono	Di	Mono	Di	Mono	Di
$MozN_3$ (equiv)	1.0	22.5	2.5	36.8	10.2	42.4	21.1
	1.1	23.5	3.1	37.7	9.8	40.2	15.6
	1.3	22.2	2.2	33.6	7.9	38.5	13.6
	1.5	23.2	2.4	33.1	7.6	38.0	12.9
	2.0	14.7	0.9	25.5	5.1	35.1	10.7

Conversion determined by LCMS (UV trace). Color gradient: lowest → highest conversion, red → green.

Single substrate optimization (2e)

The reactions were set up in a 96-well Para-dox gold block equipped with stirrer bars. Reaction scale 0.02 mmol. $[Cp^*Ir(H_2O)_3]SO_4$ (0.05M) was added as solutions in deionized water. The solvent was then removed using a Genevac HT6 centrifuge evaporator. The substrate (0.02 mmol) and $MozN_3$ (6.2 mg, 0.03 mmol) were together added as stock solutions in DCE (100 µL). The reaction mixtures were stirred at 500 rpm and heated at 60 °C for 20h. After this DMSO (200 µL) and metal scavenger were added to the vials and stirred for additional 2 hours at room temperature. Analyzed by LCMS.

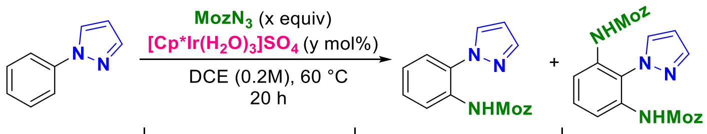

Ir (mol%)		2		4		6	
Product		**2e**	**2e´**	**2e**	**2e´**	**2e**	**2e´**
$MozN_3$ (equiv)	1.0	86.4	9.5	86.9	9.4	86.9	10.2
	1.1	85.9	10.4	86.8	7.7	85.6	13.0
	1.3	78.0	21.2	77.6	22.1	73.7	26.3
	1.5	81.1	17.5	53.9	45.8	49.6	50.1
	2.0	84.3	10.6	53.4	46.0	22.7	76.4

Conversion determined by LCMS (UV trace). Color gradient: lowest → highest conversion, red → green.

Single substrate optimization (2d)

Note: Optimization not conducted in plate format. Scale 0.1 mmol. Experiments set up in 3 consecutive rounds, round 1 (Entries 1-4), round 2 (Entries 5 and 6), Round 3 (Entries 7 and 8). Conditions of entry 8 chosen for scale up. Automatic peak integration and processing used.

$[Cp^*Ir(H_2O)_3]SO_4$ was weighed in a 2 mL glass screw top vial, followed by addition of $MozN_3$. DCE (0.5 mL) was added, followed by addition of 2-phenylpyridine (14.3 µL, 0.1 mmol). The reaction mixtures were stirred at 500 rpm and heated at 60 °C for 20h. Analyzed by LCMS.

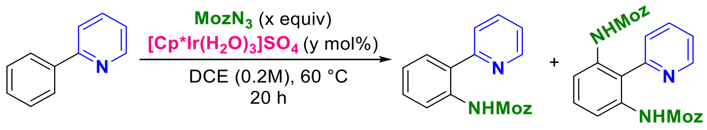

Entry	Ir (mol%)	$MozN_3$ (equiv)	% Mono	% Di
1	2	1.0	75.0	2.0
2	2	1.1	76.4	2.5
3	4	3.0	59.7	4.0
4	6	3.0	52.7	13.5
5	4	1.1	92.7	7.3
6	4	1.3	88.5	11.5
7	4	1.0	82.9	2.9
8	4	1.05	92.4	3.3

Conversion determined by LCMS (UV trace). Color gradient: lowest → highest conversion, red → green.

Single substrate optimization (2k and 2u)

The reactions were set up in a 24- or 96-well Para-dox gold block equipped with stirrer bars. Reaction scale 0.02 mmol, 100 µL total volume. $[Cp^*Ir(H_2O)_3]SO_4$ was added as solutions in deionized water. The solvent was then removed using a Genevac HT6 centrifuge evaporator. The substrate and $MozN_3$ were added as stock solutions in DCE. C_5H_9COOH (2.2 µL) was added with a multichannel pipette. The reaction mixtures were stirred at 500 rpm and heated at 60 °C for 20h. After this DMSO (200 µL) and metal scavenger were added to the vials and stirred for additional 2 hours at room temperature. Analyzed by LCMS.

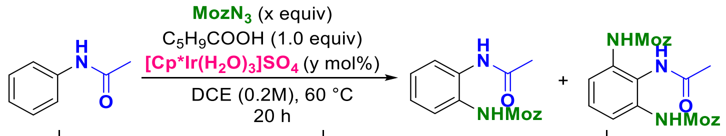

Ir (mol%)		2 mol%		4 mol%		6 mol%	
		Mono (%)	Di (%)	Mono (%)	Di (%)	Mono (%)	Di (%)
$MozN_3$ (equiv)	1.1	47.1	0.9	72.6	4.0	78.4	5.4
	1.3	42.7	0.8	70.8	3.6	80.7	6.5
	1.5	40.3	0.7	66.3	3.0	77.5	5.6

Conversion determined by LCMS (UV trace). Color gradient: lowest → highest conversion, red → green.

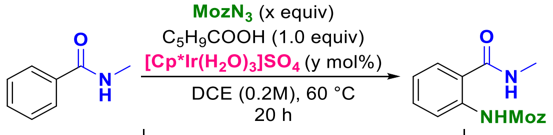

Solvent		DCE			CPME			EtOAc		
Ir (mol%)		2	4	6	2	4	6	2	4	6
$MozN_3$ (equiv)	1.1	34.2	56.3	66.6	15.1	25.7	36.7	5.2	17.5	32.6
	1.3	32.6	50.6	66.5	10.1	25.0	38.7	5.7	15.4	30.7
	1.5	31.9	51.9	63.5	10.3	28.0	39.7	4.5	15.8	29.2

Conversion determined by LCMS (UV trace). Color gradient: lowest → highest conversion, red → green.

Single substrate optimization (3a and 3a´)

The reactions were set up in a 96-well Para-dox gold block equipped with stirrer bars. Reaction scale 0.02 mmol. $[Cp^*Ir(H_2O)_3]SO_4$ (0.05M) was added as solutions in deionized water. The solvent was then removed using a Genevac HT6 centrifuge evaporator. *Atazanavir* (14.1 mg, 0.02 mmol) was added as solid using the Quantos powder dosing system. $MozN_3$ (0.3M, 100 µL, 0.03 mmol) was added as stock solutions in respective solvents. The reaction mixtures were stirred at 500 rpm and heated at 60 °C for 20h. After this DMSO (200 µL) and metal scavenger were added to the vials and stirred for additional 2 hours at room temperature. Analyzed by LCMS.

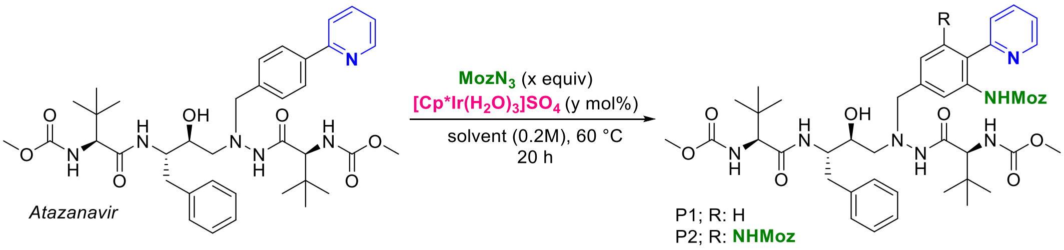

		DCE						NMP					
Ir (mol%)		2		4		6		4		6		8	
		P1	P2	P1	P2	P1	P2	P1	P2	P1	P2	P1	P2
$MozN_3$ (equiv)	1.0	86.5	3.3	90.2	3.7	92.0	4.0	73.4	8.6	77.4	9.3	81.9	11.2
	1.1	89.2	4.5	92.1	7.9	94.2	5.8	76.8	13.6	78.3	19.5	80.6	19.4
	1.3	83.3	16.7	80.0	20.0	80.9	19.1	74.9	13.0	76.2	23.8	67.8	32.2
	1.5	63.0	37.0	63.9	36.1	65.0	35.0	73.8	11.9	64.8	35.2	65.2	34.8
	2.0	43.5	56.5	14.3	85.7	13.0	87.0	72.6	11.6	58.4	32.5	46.9	53.1
	3.0	61.4	38.6	21.9	21.9	0.0	100	74.8	8.9	76.0	24.0	60.0	40.0

Conversion determined by LCMS (UV trace). Color gradient: lowest → highest conversion, red → green.

Single substrate optimization (**3e**)

The reactions were set up in a 96-well Para-dox gold block equipped with stirrer bars. Reaction scale 0.02 mmol. $[Cp^*Ir(H_2O)_3]SO_4$ (0.05M) was added as solutions in deionized water. The solvent was then removed using a Genevac HT6 centrifuge evaporator. *Telmisartan* (10.3 mg, 0.02 mmol) was added as solid using the Quantos powder dosing system. $MozN_3$ (0.3M, 100 µL, 0.03 mmol) was added as stock solution in respective solvent. The reaction mixtures were stirred at 500 rpm and heated at 60 °C for 20h. After this DMSO (200 µL) and metal scavenger were added to the vials and stirred for additional 2 hours at room temperature. Analyzed by LCMS.

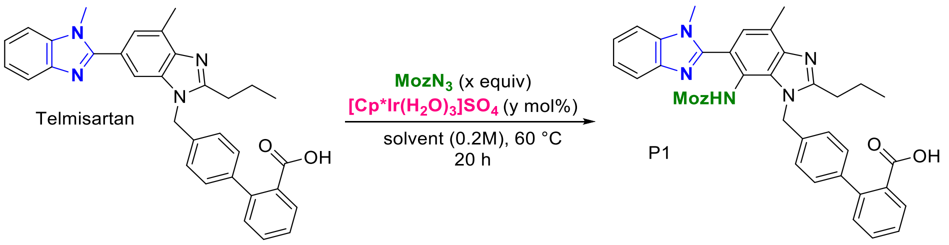

	DCE						NMP					
Ir (mol%)	2		4		6		4		6		8	
$MozN_3$ (equiv)	P1	P2	P1	P2	P1	P2	P1	P2	P1	P2	P1	P2
1.0	2.4	1.3	4.6	1.8	6.7	2.4	4.9	0.3	7.6	0.3	14.2	0.6
1.1	2.7	1.1	4.6	2.7	5.5	3.9	5.7	0.3	10.3	0.4	15.2	0.5
1.3	2.8	0.8	5.0	1.6	6.2	3.4	5.8	0.3	8.3	0.3	15.4	0.4
1.5	2.1	0.9	6.0	1.6	6.8	2.8	5.9	0.3	9.4	0.4	16.7	0.7
2.0	2.5	0.8	5.6	1.7	7.7	1.8	6.2	0.2	11.5	0.4	18.2	0.7
3.0	2.2	0.4	5.8	1.0	8.4	1.7	6.7	0.3	12.8	0.4	16.7	0.6

Conversion determined by LCMS (UV trace). Color gradient: lowest → highest conversion, red → green.

Functional group tolerance study

The reactions were set up in a 96-well Para-dox gold block equipped with stirrer bars. Reaction scale 0.02 mmol. 48 additives screened against 2 solvents (NMP, DCE). $[Cp^*Ir(H_2O)_3]SO_4$ (0.05M, 40 µL, 2 µmol) was added as solution in deionized water. The solvent was then removed using a Genevac HT6 centrifuge evaporator. The solid additives (0.02 mmol) were weighed in manually. 2-(3-methylphenyl)pyridine (0.02 mmol) and $MozN_3$ (0.03 mmol) were added together as stock solution in respective solvents (100 µL per vial). The liquid additives (0.02 mmol) were added manually using a single channel pipette. The reaction mixtures were stirred at 500 rpm and heated at 60 °C for 20h. After this DMSO (200 µL) and metal scavenger were added to the vials and stirred for additional 2 hours at room temperature. Analyzed by LCMS.

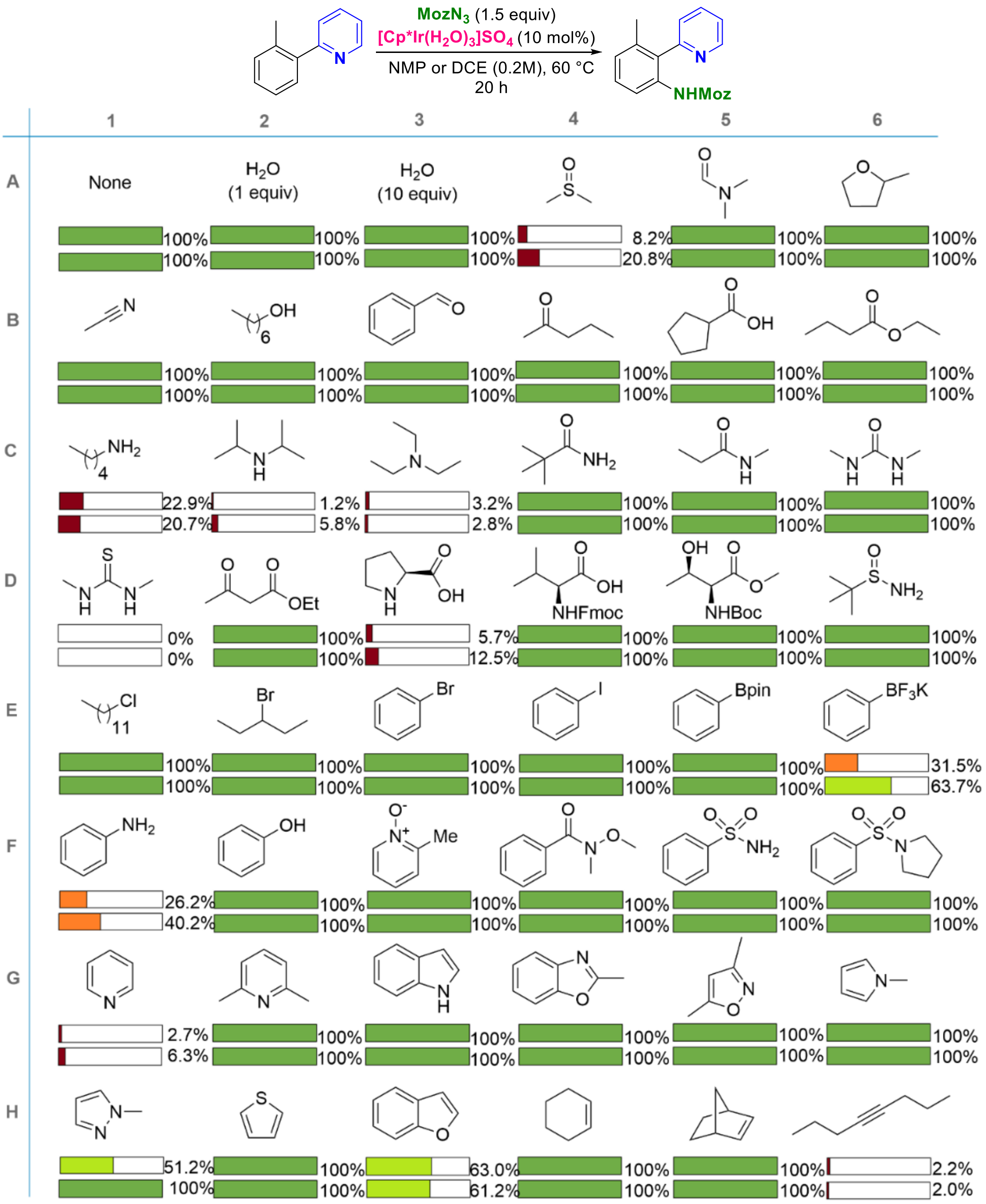

Conversion determined by LCMS (UV trace). Top bars NMP, bottom bars DCE. Color coding: green >50%; orange 25-50%, red <25%.

Directing group informer library

The reactions were set up in a 96-well Para-dox gold block equipped with stirrer bars. Reaction scale 0.02 mmol. [Cp*Ir(H_2O)$_3$]SO_4 (0.05M, 40 µL, 2 µmol) was added as solutions in deionized water. The solvent was then removed using a Genevac HT6 centrifuge evaporator. The solid substrates were weighed in manually. After this, MozN_3 (0.3M, 100 µL, 0.03 mmol) was added as stock solution in respective solvents. Addition of liquid substrates (0.02 mmol) followed. The reaction mixtures were stirred at 500 rpm and heated at 60 °C for 20h. After this DMSO (200 µL) and metal scavenger were added to the vials and stirred for additional 2 hours at room temperature. Analyzed by LCMS.

Below: successful examples

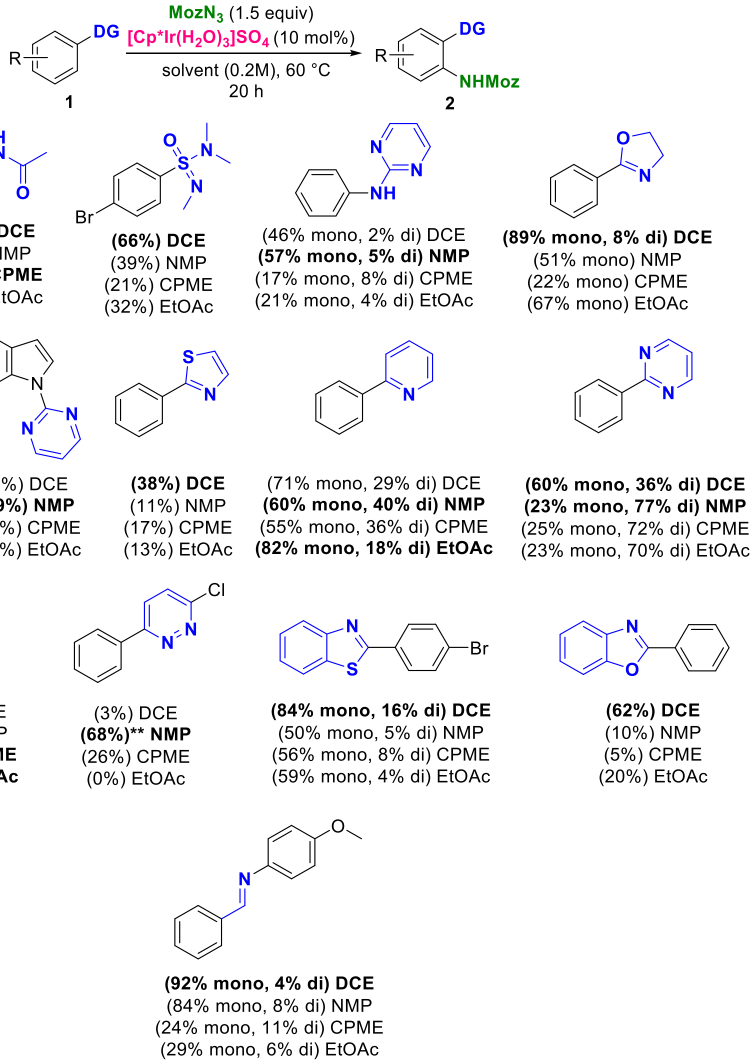

Successful examples, identity confirmed by NMR analysis at screening scale. Conversion determined by LCMS (UV trace).

Below: Purification was not attempted due to low conversion (top row) or insufficient separation in analytical LCMS (bottom row).

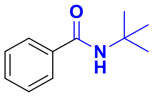

(17%) DCE
(0%) NMP
(16%) CPME
(10%) EtOAc

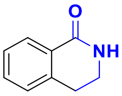

(18%) DCE
(0%) NMP
(26%) CPME
(11%) EtOAc

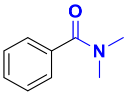

(4%) DCE
(0%) NMP
(6%) CPME
(10%) EtOAc

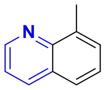

(4%) DCE
(3%) NMP
(1%) CPME
(6%) EtOAc

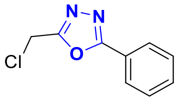

(0%) DCE
(4%) NMP
(0%) CPME
(0%) EtOAc

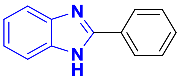

(1%) DCE
(17%) NMP
(0%) CPME
(0%) EtOAc

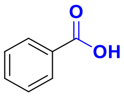

(16% mono, 11% di)[a] DCE
(4% mono, 4% di)[a] NMP
(12% mono, 4% di)[a] CPME
(20% mono, 7% di)[a] EtOAc

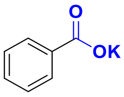

(0% mono, 44% di)[a] DCE
(4% mono, 6% di)[a] NMP
(0% mono, 0% di)[a] CPME
(0% mono, 56% di)[a] EtOAc

Conversion determined by LCMS (UV trace).

Below: Compounds with failed purification. The four examples presented are counted as unsuccessful reactions.

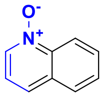

(22%) DCE
(5%) NMP
(34%) CPME
(28%) EtOAc

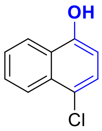

(0%) DCE
(28%) NMP
(0%) CPME
(0%) EtOAc

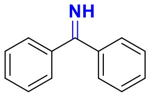

(39%) DCE
(74%) NMP
(35%) CPME
(33%) EtOAc

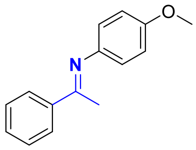

(86% mono) DCE
(54% mono, 31% di) NMP
(24% mono, 22% di) CPME
(2% mono, 20% di) EtOAc

Conversion determined by LCMS (UV trace).Potential reasons for failed purification: Decomposition during sample preparation and/or purification, lack of ionization and detection of predicted product mass during the purification run, or observation of false positives by LCMS.

Below: Compounds with no conversion observed

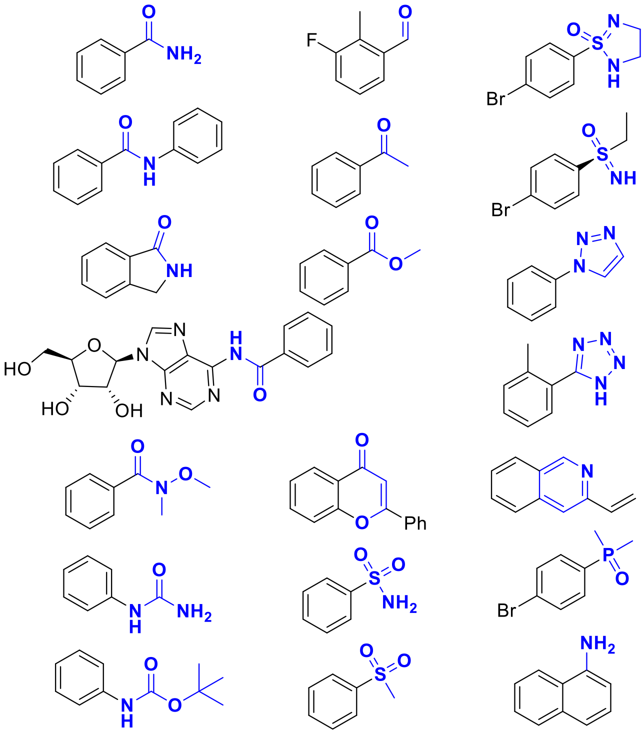

Analyzed by LCMS (UV trace).

LSF informer library

The reactions were set up in a 96-well Para-dox gold block equipped with stirrer bars. Reaction scale 0.02 mmol. [Cp*Ir(H_2O)$_3$]SO_4 (0.05M, 40 µL, 2 µmol) was added as solutions in deionized water. The solvent was then removed using a Genevac HT6 centrifuge evaporator. The substrates (0.02 mmol) were weighed in manually. After this, $MozN_3$ (0.3M, 100 µL, 0.03 mmol) was added as stock solution in respective solvents. The reaction mixtures were stirred at 500 rpm and heated at 60 °C for 20h. After this DMSO (200 µL) and metal scavenger were added to the vials and stirred for additional 2 hours at room temperature. Analyzed by LCMS. Selected examples (highlighted in colored squares) were submitted for purification by HPLC.

Below: Successful isolated examples

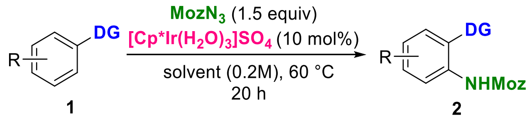

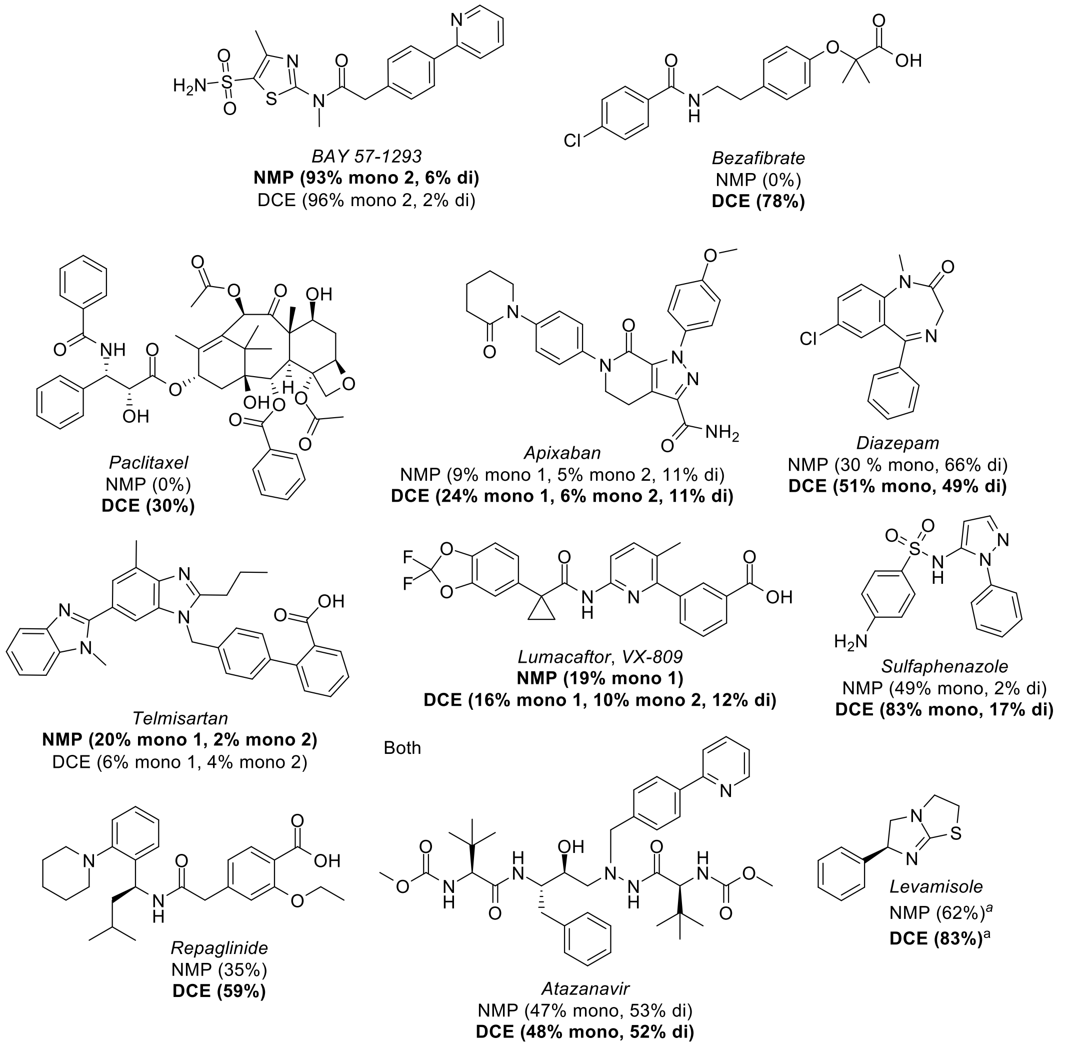

Conversion determined by LCMS (UV trace). [a] Only difunctionalization observed.

Below: Substrate not chosen for purification due to low conversion or product decomposition. Counted as unsuccessful reactions.

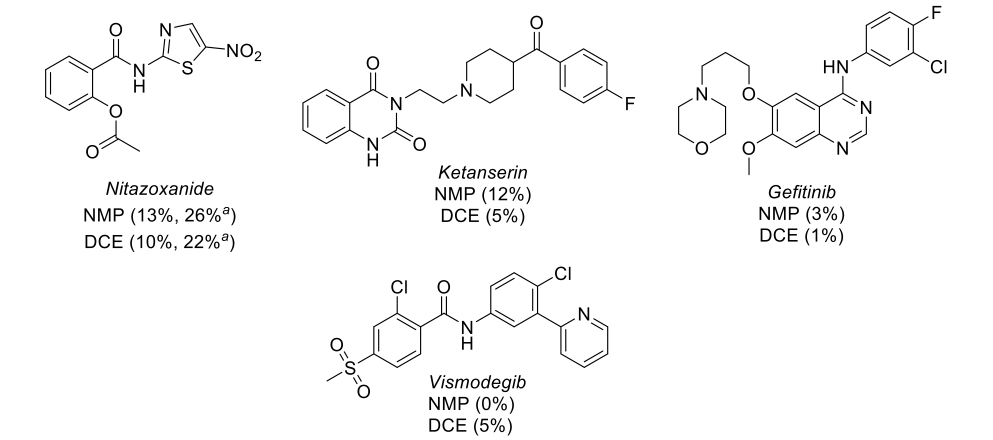

Conversion determined by LCMS (UV trace). [a] Product with O-deacetylation under reaction conditions.

LSF Scope: side reaction

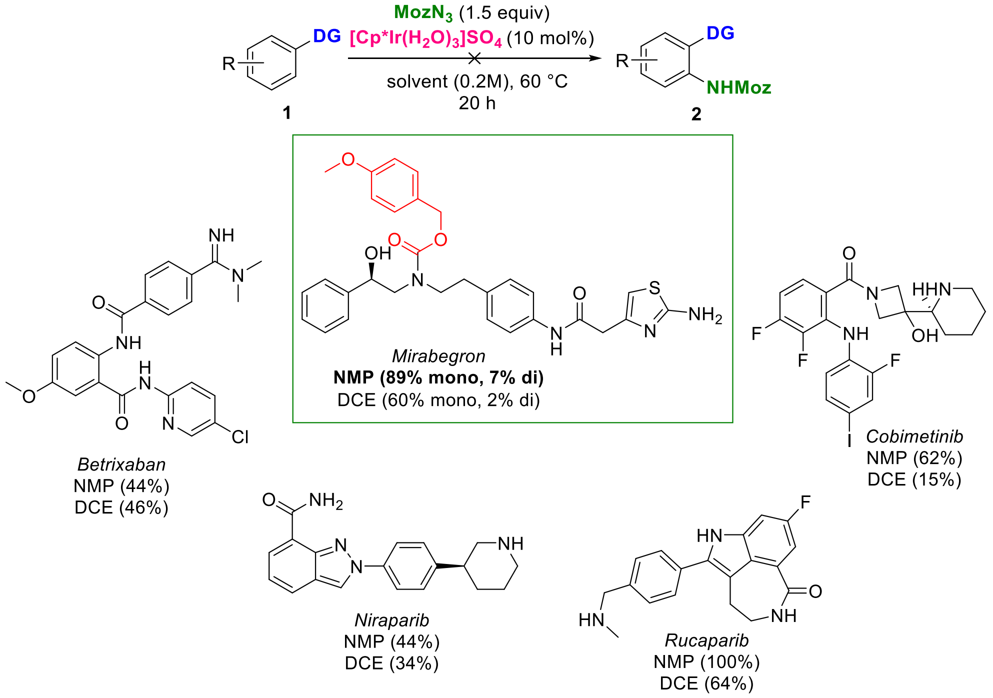

Conversion determined by LCMS (UV trace). Mirabegron analogue isolated and characterized.

In all depicted cases formation of a new species (% conversion in parentheses) was observed, in all cases with a MW lower by 15 Dalton then for the expected products. In the case of Mirabegron the product was isolated and confirmed as the addition product depicted. As the common feature of the compounds with which this mass difference was observed was the presence of highly nucleophilic functional groups, at this point we speculate that the same side reaction occurred in all presented cases.

Below: Compounds with failed purification

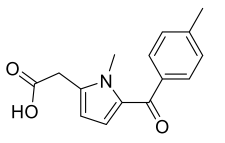

Tolmetin
NMP (0%)
DCE (12%)

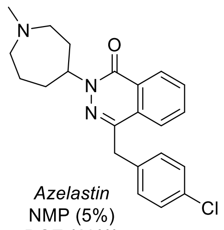

Azelastin
NMP (5%)
DCE (13%)

Conversion determined by LCMS (UV trace).Potential reasons for failed purification: Decomposition during sample preparation and/or purification, lack of ionization and detection of predicted product mass during the purification run, or observation of false positives by LCMS.

Unsuccessful substrates (no conversion)

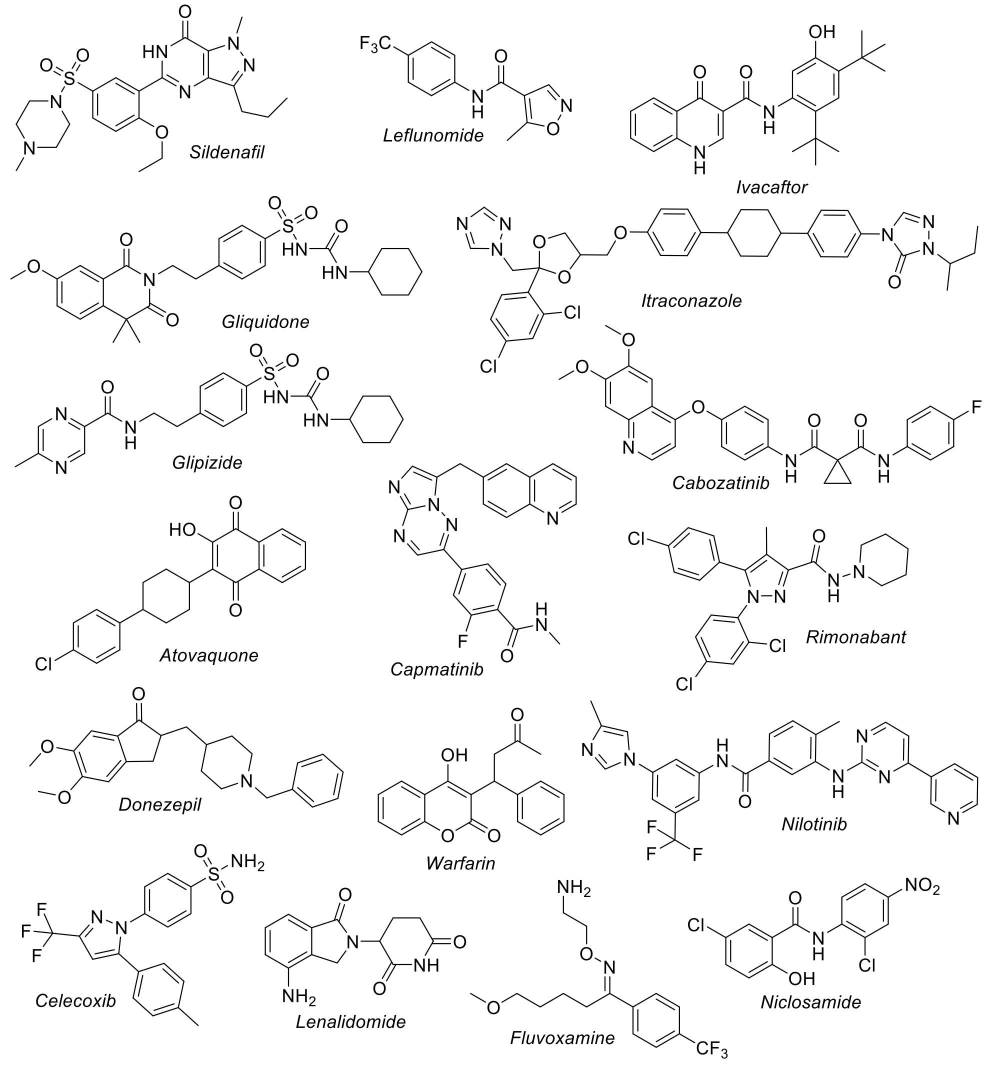

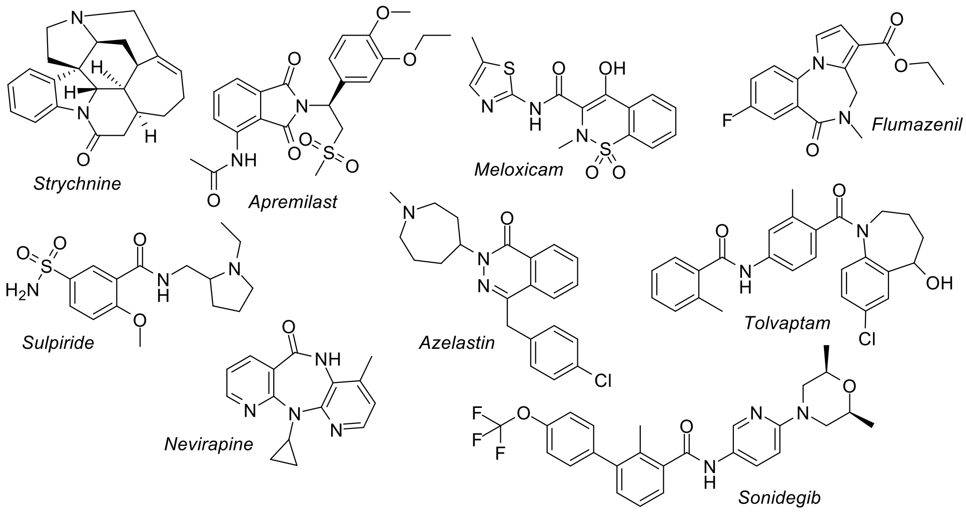

Determined by LCMS.

Miniaturization studies

Reaction scale 0.2 - 0.8 µmol

The reactions were set up in a 384-well Plate+™ glass-coated microplate. Stock solutions S1 were prepared with the respective substrates (0.256 mmol) and $MozN_3$ (0.256 mmol) in 640 µL NMP. Stock solution S2 was prepared from $[Cp^*Ir(H_2O)_3]SO_4$ (0.04 mmol) in 1 mL NMP. Reactions were run at three distinct volumes: 1, 2 and 4 µL. A column control experiments with no catalyst was also set up for each substrate. The stock solutions were dispensed to the reaction plate using the Mosquito liquid dispensing system, with S1 added first, followed by S2. The plate was sealed and heated at 60 °C in a Genevac centrifuge at atmospheric pressure (Note: Using the centrifuge for the reaction minimized solvent loss). The plate was allowed to reach ambient temperature, centrifuged for 5 min at 2500 rpm, seal removed. Metal scavenger (Si-IMI, 2 equiv) was added in NMP. The reactions were diluted with NMP to a total of 40 µL. The plate was sealed and heated at 50 °C on an orbital shaker. An analytical plate was made, with 2 mM concentration, solvent DMSO. Analyzed by LCMS. Reactions were successful throughout the plate, including the lowest reaction volume (1 µL, see manuscript).

Reaction scale 1.0 – 8.0 mmol

The reactions were set up in a 1536-well echo plate. Stock solutions S1 were prepared with the respective substrates (0.1 mmol) and $MozN_3$ (0.1 mmol) in 233 µL NMP. Stock solution S2 was prepared from $[Cp^*Ir(H_2O)_3]SO_4$ (8 µmol) in 200 µL NMP. Reactions were run at four distinct volumes: 5, 10, 20 and 40 nL. The stock solutions were dispensed to the reaction plate using the Echo acoustic liquid handling system, with S1 added first, followed by S2. The plate was sealed and heated at 60 °C in a Genevac centrifuge at atmospheric pressure (Note: Using the centrifuge for the reaction minimized solvent loss). An analytical plate was made, with 2 mM concentration, solvent water. Analyzed by LCMS and AMI-MS. Product detection by MS was observed throughout the plate, even at the lowest reaction volumes (5 nL).

General experimental procedures

General Procedure A

$[Cp^*Ir(H_2O)_3]SO_4$ (9.7 mg, 0.02 mmol, 4 mol%) was weighed in a glass screw-top vial (16 mL). $MozN_3$ (134.7 mg, 0.65 mmol) was added, followed by addition of substrate (0.5 mmol). The vial was

equipped with a stirrer bar, followed by addition of DCE (2.5 mL). The sealed vial was placed in a heating block at 60 °C and stirred for 20 hours.

General Procedure B

[Cp*Ir(H_2O)$_3$]SO_4 (3.9 mg, 8.0 µmol, 4 mol%) was weighed in a glass screw-top vial (4 mL). MozN_3 (41.4 mg, 0.2 mmol) was added, followed by addition of substrate (0.2 mmol). The vial was equipped with a stirrer bar, followed by addition of solvent (1.0 mL). The sealed vial was placed in a heating block at 60 °C and stirred for 20 hours.

General Procedure C

Based on screening conditions. [Cp*Ir(H_2O)$_3$]SO_4 (4.9 mg, 0.01 mmol, 10 mol%) was weighed in a glass screw-top vial (2 mL). MozN_3 (31.1 mg, 0.15 mmol) was added, followed by addition of substrate (0.1 mmol). The vial was equipped with a stirrer bar, followed by addition of solvent (0.5 mL). The sealed vial was placed in a heating block at 60 °C and stirred for 20 hours.

General Procedure D

Reaction set-up according to Directing group and LSF informer library chapters.

General Procedure E

Screening format, selected examples isolated. The reactions were set up in a 96-well Para-dox gold block equipped with stirrer bars. Reaction scale 0.02 mmol, 100 µL total volume. [Cp*Ir(H_2O)$_3$]SO_4 was added as solutions in deionized water (final loading 1.0mg, 2 µmol per vial). The solvent was then removed using a Genevac HT6 centrifuge evaporator. The solid substrates were weighed in manually (0.02 mmol). MozN_3 (per vial 6.2 mg, 0.03 mmol) was added as stock solution in respective solvents. Liquid substrates (0.02 mmol) were added. The reaction mixtures were stirred at 500 rpm and heated at 60 °C for 20h. After this DMSO (200 µL) and metal scavenger were added to the vials and stirred for additional 2 hours at room temperature. Analyzed by LCMS. Purified by preparative reverse phase HPLC.

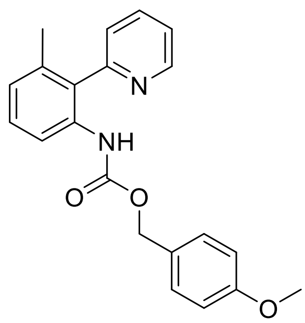

4-methoxybenzyl (3-methyl-2-(pyridin-2-yl)phenyl)carbamate (**2a**) was prepared according to General Procedure A. Purification by automated flash column chromatography (0-80% EtOAc in heptane, 25g SiO_2). Compound **2a** was obtained as a colorless solid (132.7 mg, 76%).

^{1}H NMR (500 MHz, $CDCl_3$) δ 8.72 (ddd, *J* = 4.9, 1.7, 0.9 Hz, 1H), 7.96 (s, 1H), 7.78 (td, *J* = 7.7, 1.8 Hz, 1H), 7.72 (s, 1H), 7.27 – 7.34 (m, 5H), 7.02 (d, *J* = 7.6 Hz, 1H), 6.84 – 6.88 (m, 2H), 5.04 (s, 2H), 3.79 (s, 3H), 2.15 (s, 3H). **^{13}C NMR** (126 MHz, $CDCl_3$) δ 159.7, 156.7, 153.7, 149.8, 137.0, 136.2, 135.7, 130.2, 130.0, 128.9, 128.4, 126.2, 125.8, 122.5, 118.6, 114.0, 66.7, 55.4, 20.9. **HRMS** (ESI) m/z calcd. for $C_{21}H_{20}N_2O_3$ $[M+H]^+$: 349.1552, found 349.1554.

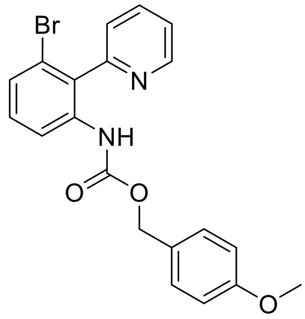

4-methoxybenzyl (3-bromo-2-(pyridin-2-yl)phenyl)carbamate (**2b**) was prepared according to General Procedure A. Purification by automated flash column chromatography (0-80% EtOAc in heptane, 25g SiO_2). Compound **2b** was obtained as a colorless wax (76.1 mg, 37%).

^{1}H NMR (500 MHz, $CDCl_3$) δ 8.71 (ddd, *J* = 4.9, 1.8, 0.9 Hz, 1H), 8.13 (d, *J* = 7.5 Hz, 1H), 7.95 (s, 1H), 7.82 (td, *J* = 7.7, 1.8 Hz, 1H), 7.54 (dt, *J* = 7.9, 1.0 Hz, 1H), 7.40 (dd, *J* = 8.1, 1.1 Hz, 1H), 7.33 (ddd, *J* = 7.6, 4.9, 1.2 Hz, 1H), 7.26 – 7.3 (m, 2H), 7.22 – 7.26 (m, 1H), 6.85 – 6.89 (m, 2H), 5.04 (s, 2H), 3.80 (s, 3H). **^{13}C NMR** (126 MHz, $CDCl_3$) δ 159.8, 156.0, 153.5, 149.5, 137.6, 137.0, 130.4, 130.3, 130.1, 128.2, 127.3 (2C), 123.2, 122.7, 120.1, 114.0, 67.0, 55.4. **HRMS** (ESI) m/z calcd. for $C_{20}H_{17}BrN_2O_3$ $[M+H]^+$: 413.0501, found 413.0513.

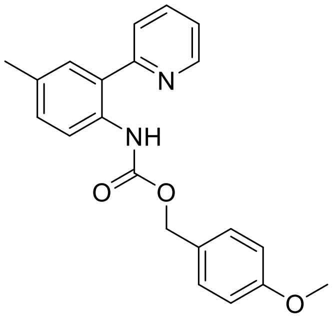

4-methoxybenzyl (3-methyl-2-(pyridin-2-yl)phenyl)carbamate (**2c**) was prepared according to General Procedure A. Purification by automated flash column chromatography (0-80% EtOAc in heptane, 25g SiO_2). Compound **2c** was obtained as a colorless solid (152.5 mg, 88%).

The same compound was also prepared using CPME (2.5 mL) as solvent and increased [Cp*Ir(H_2O)$_3$]SO_4 (14.6 mg, 0.03 mmol, 6 mol%) according to otherwise standard conditions. Compound **2c** was obtained as a colorless solid (153.0 mg, 88%).

^{1}H NMR (500 MHz, $CDCl_3$) δ 11.22 (s, 1H), 8.62 (ddd, *J* = 4.9, 1.8, 0.9 Hz, 1H), 8.20 (d, *J* = 7.8 Hz, 1H), 7.79 (td, *J* = 7.8, 1.9 Hz, 1H), 7.68 (d, *J* = 8.1 Hz, 1H), 7.40 (d, *J* = 1.6 Hz, 1H), 7.34 – 7.38 (m, 2H), 7.2 – 7.24 (m, 2H), 6.88 – 6.91 (m, 2H), 5.12 (s, 2H), 3.81 (s, 3H), 2.36 (s, 3H). **^{13}C NMR** (126 MHz, $CDCl_3$) δ 159.6, 158.4, 154.2, 147.9, 137.6, 135.2, 132.1, 130.8, 130.2, 129.6, 128.9, 125.8, 123.0, 121.8, 120.8, 114.0, 66.4, 55.4, 21.0. **HRMS** (ESI) m/z calcd. for $C_{21}H_{20}N_2O_3$ $[M+H]^+$: 349.1552, found 349.1550.

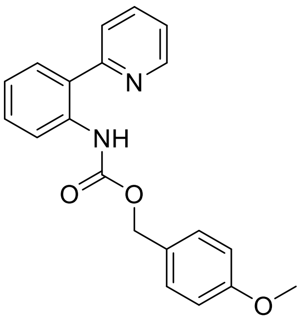

4-methoxybenzyl (2-(pyridin-2-yl)phenyl)carbamate (**2d**) was prepared according to modified General Procedure A, MozN_3 (108.8 mg, 0.525 mmol). Purification by automated flash column chromatography (0-80% EtOAc in heptane, 25g SiO_2). Compound **2d** was obtained as a colorless solid (143.2 mg, 86%).

^{1}H NMR (500 MHz, $CDCl_3$) δ 11.48 (s, 1H), 8.63 (ddd, *J* = 4.9, 1.8, 0.9 Hz, 1H), 8.35 (d, *J* = 8.2 Hz, 1H), 7.78 – 7.82 (m, 1H), 7.69 (d, *J* = 8.1 Hz, 1H), 7.61 (dd, *J* = 7.8, 1.5 Hz, 1H), 7.35 – 7.42 (m, 3H), 7.24 (ddd, *J* = 7.5, 4.9, 1.1 Hz, 1H), 7.12 (td, *J* = 7.8, 1.2 Hz, 1H), 6.88 – 6.92 (m, 2H), 5.14 (s, 2H), 3.81 (s, 3H). **^{13}C NMR** (126 MHz, $CDCl_3$) δ 159.6, 158.3, 154.1, 147.80, 137.83, 137.7, 130.2, 130.1, 129.1, 128.8, 125.6, 123.0, 122.7, 121.9, 120.6, 114.0, 66.5, 55.4. **HRMS** (ESI) m/z calcd. for $C_{20}H_{18}N_2O_3$ $[M+H]^+$: 335.1396, found 335.1401.

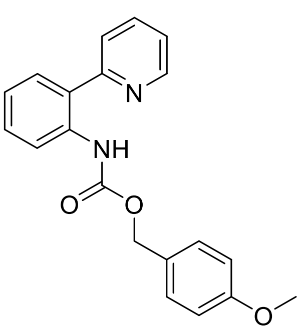

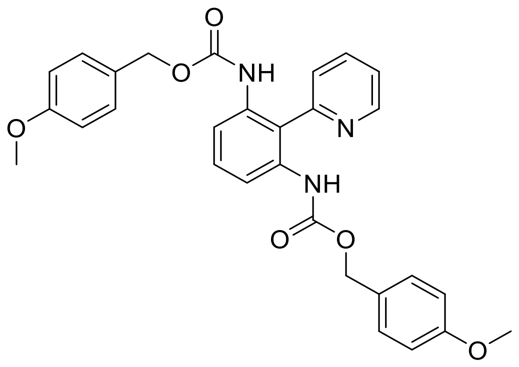

4-methoxybenzyl (2-(pyridin-2-yl)phenyl)carbamate (**2d**) and bis(4-methoxybenzyl) (2-(pyridin-2-yl)-1,3-phenylene)dicarbamate (**2d´**) were prepared according to general conditions E. Purification by preparative LC (pH 10, basic method). Compound **2d** (2.5 mg, 37%, >95% purity) was obtained as a colorless solid. Compound **2d´** (0.8 mg, 8%, >95% purity) was obtained as a colorless solid.

2d´

^{1}H NMR (500 MHz, DMSO) δ 9.18 (s, 2H), 8.67 (ddd, *J* = 4.9, 1.8, 0.9 Hz, 1H), 7.80 (td, *J* = 7.8, 1.8 Hz, 1H), 7.46 (d, *J* = 7.4 Hz, 2H), 7.35 – 7.4 (m, 3H), 7.22 (d, *J* = 8.7 Hz, 4H), 6.88 – 6.92 (m, 4H), 4.92 (s, 4H), 3.74 (s, 6H). **^{13}C NMR** (151 MHz, DMSO) δ 159.1, 154.4, 153.7, 148.8, 137.0, 136.1, 129.8, 129.0, 128.5, 125.8, 122.5, 120.4, 113.8, 65.6, 55.1. **HRMS** (ESI) m/z calcd. For $C_{29}H_{27}N_3O_6$ $[M+H]^+$: 514.1978, found 514.1995.

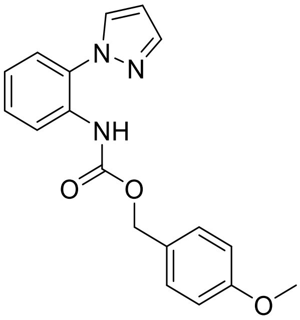

4-methoxybenzyl (2-(1H-pyrazol-1-yl)phenyl)carbamate (**2e**) was prepared according to modified General Procedure A, $[Cp^*Ir(H_2O)_3]SO_4$ (4.9 mg, 0.01 mmol, 2 mol%), $MozN_3$ (103.6 mg, 0.5 mmol). Purification by automated flash column chromatography (0-80% EtOAc in heptane, 25g SiO_2). Compound **2e** was obtained as a colorless solid (116.7 mg, 72%).

^{1}H NMR (500 MHz, DMSO) δ 9.81 (s, 1H), 8.27 (d, *J* = 2.4 Hz, 1H), 8.01 (d, *J* = 7.9 Hz, 1H), 7.8 – 7.91 (m, 1H), 7.57 (dd, *J* = 8.0, 1.4 Hz, 1H), 7.39 (td, *J* = 8.0, 1.5 Hz, 1H), 7.29 – 7.36 (m, 2H), 7.23 (td, *J* = 7.9, 1.4 Hz, 1H), 6.86 – 7 (m, 2H), 6.55 – 6.61 (m, 1H), 5.04 (s, 2H), 3.75 (s, 3H). **^{13}C NMR** (126 MHz, DMSO) δ 159.2, 153.2, 141.1, 131.2, 130.7, 130.1, 129.9, 128.2, 127.8, 124.2, 123.5, 122.5, 113.9, 107.3, 66.1, 55.1. **HRMS** (ESI) m/z calcd. For $C_{18}H_{17}N_3O_3$ $[M+H]^+$: 324.1348, found 324.1343.

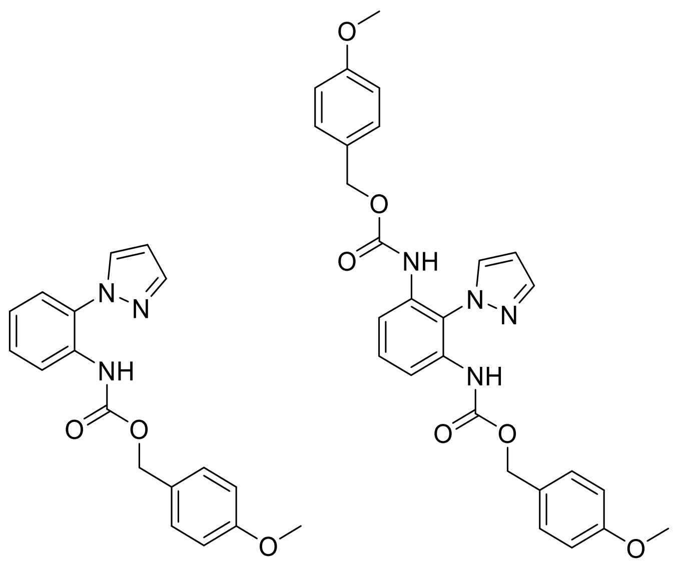

4-methoxybenzyl (2-(1H-pyrazol-1-yl)phenyl)carbamate (**2e**) and bis(4-methoxybenzyl) (2-(1H-pyrazol-1-yl)-1,3-phenylene)dicarbamate (**2e´**) were prepared according to general conditions E. Purification by preparative LC (pH 3, FP column, acidic method). Compound **2e** (3.0 mg, 46%, >95% purity) was obtained as a colorless solid. Compound **2e´** (1.0 mg, 10%, >95% purity) was obtained as a colorless wax.

2e´
^{1}H NMR (500 MHz, DMSO) δ 8.61 (s, 2H), 7.78 – 7.85 (m, 2H), 7.53 (d, *J* = 8.0 Hz, 2H), 7.42 (t, *J* = 8.2 Hz, 1H), 7.24 – 7.27 (m, 4H), 6.89 – 6.93 (m, 4H), 6.48 – 6.51 (m, 1H), 4.96 (s, 4H), 3.74 (s, 6H).
^{13}C NMR (151 MHz, DMSO) δ 159.2, 153.6, 141.2, 133.9, 133.1, 129.9, 128.8, 128.2, 113.8, 106.7, 65.9, 55.1. **HRMS** (ESI) m/z calcd. For $C_{27}H_{26}N_4O_6$ $[M+H]^+$: 503.1931, found 503.1952.

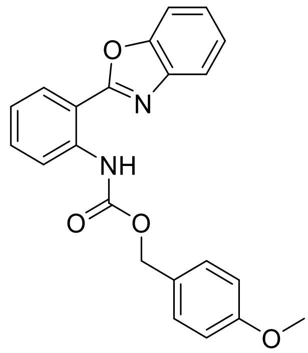

4-methoxybenzyl (2-(benzo[d]oxazol-2-yl)phenyl)carbamate (**2f**) was prepared according to modified General Procedure B. $[Cp^*Ir(H_2O)_3]SO_4$ (5.8 mg, 12.0 µmol, 6 mol%), solvent DCE (1.0 mL). Purification by automated flash column chromatography (0-40% EtOAc in heptane, 25g SiO_2). Compound **2f** was obtained as a colorless solid (30.2 mg, 40%).

^{1}H NMR (500 MHz, DMSO) δ 11.12 (s, 1H), 8.41 (d, *J* = 8.1 Hz, 1H), 8.19 (dd, *J* = 8.0, 1.4 Hz, 1H), 7.8 – 7.86 (m, 2H), 7.63 (td, *J* = 8.7, 8.1, 1.5 Hz, 1H), 7.48 (td, *J* = 7.8, 1.4 Hz, 1H), 7.4 – 7.45 (m, 3H), 7.25 – 7.29 (m, 1H), 6.95 – 6.98 (m, 2H), 5.19 (s, 2H), 3.76 (s, 3H). **^{13}C NMR** (126 MHz, DMSO) δ 161.3, 159.3, 153.1, 148.7, 140.2, 138.5, 133.0, 130.2, 128.6, 128.2, 126.1, 125.2, 122.7, 119.6, 118.7, 113.9, 112.4, 111.0, 66.3, 55.1. **HRMS** (ESI) m/z calcd. For $C_{22}H_{18}N_2O_4$ $[M+H]^+$: 375.1345, found 375.1342.

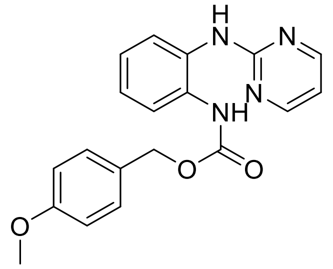

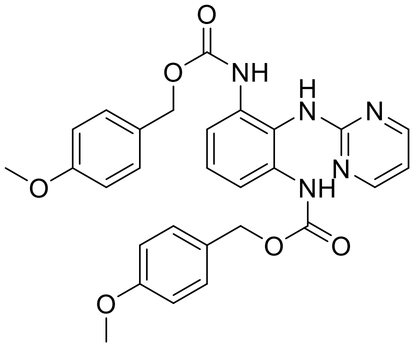

4-methoxybenzyl (2-(pyrimidin-2-ylamino)phenyl)carbamate (**2g**) and bis(4-methoxybenzyl) (2-(pyrimidin-2-ylamino)-1,3-phenylene)dicarbamate (**2g´**) were prepared according to General Procedure C. Purification by preparative reverse phase HPLC (36-76% MeCN in NH_3 buffer) to give **2g** (16.2 mg, 46%) as a colorless solid, and **2g´** (3.5 mg, 7%) as a colorless solid.

2g

^{1}H NMR (500 MHz, DMSO) δ 8.98 (d, *J* = 23.1 Hz, 1H), 8.66 (s, 1H), 8.40 (d, *J* = 4.8 Hz, 2H), 7.71 (dd, *J* = 7.8, 1.7 Hz, 1H), 7.49 (d, *J* = 7.3 Hz, 1H), 7.35 (d, *J* = 8.5 Hz, 2H), 7.07 – 7.14 (m, 2H), 6.91 – 6.94 (m, 2H), 6.81 (t, *J* = 4.8 Hz, 1H), 5.06 (s, 2H), 3.75 (s, 3H). **^{13}C NMR** (151 MHz, DMSO) δ 160.4, 159.2, 158.2, 154.3, 131.8, 130.2, 130.0, 128.4, 124.4, 123.9, 113.8, 112.6, 65.9, 55.1. **HRMS** (ESI) m/z calcd. For $C_{19}H_{18}N_4O_3$ $[M+H]^+$: 351.1457, found 351.1443.

2g´

^{1}H NMR (500 MHz, $CDCl_3$) δ 8.28 (d, *J* = 4.8 Hz, 2H), 7.62 (s, 2H), 7.27 – 7.32 (m, 5H), 6.97 (s, 2H), 6.87 (d, *J* = 8.6 Hz, 4H), 6.84 (s, 1H), 6.67 (t, *J* = 4.8 Hz, 1H), 5.08 (s, 4H), 3.80 (s, 6H). **^{13}C NMR** (151 MHz, $CDCl_3$) δ 161.3, 159.8, 158.7, 153.8, 135.4, 130.3, 128.1, 128.0, 114.0, 113.1, 77.2, 77.0, 76.8, 67.1, 55.3. **HRMS** (ESI) m/z calcd. For $C_{28}H_{27}N_5O_6$ $[M+H]^+$: 530.2039, found 530.2052.

4-methoxybenzyl (2-(pyrimidin-2-ylamino)phenyl)carbamate (**2g**) and bis(4-methoxybenzyl) (2-(pyrimidin-2-ylamino)-1,3-phenylene)dicarbamate (**2g´**) were prepared according to general conditions E. Purification by preparative LC (pH 10, basic method). Compound **2g** (2.3 mg, 33%, >95% purity) was obtained as a colorless solid. Compound **2e´** (0.5 mg, 5%, >95% purity) was obtained as a colorless wax.

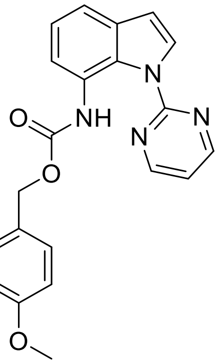

4-methoxybenzyl (1-(pyrimidin-2-yl)-1H-indol-7-yl)carbamate (**2h**) was prepared according to General Procedure B. Solvent DCE. Purification by automated flash column chromatography (0-80% EtOAc in heptane, 25g SiO_2). Compound **2h** was obtained as a colorless solid (30.2 mg, 40%).

The same compound was also prepared using EtOAc (1.0 mL) as solvent and increased $[Cp^*Ir(H_2O)_3]SO_4$ (5.9 mg, 12.0 µmol, 6 mol%) according to otherwise standard conditions. Compound **2h** (30.7 mg, 41%) was obtained as a colorless solid.

^{1}H NMR (500 MHz, $CDCl_3$) δ 11.23 (s, 1H), 8.72 (d, *J* = 4.8 Hz, 2H), 8.59 – 8.65 (m, 1H), 7.48 – 7.51 (m, 1H), 7.39 – 7.43 (m, 2H), 7.17 – 7.23 (m, 2H), 7.09 (t, *J* = 4.8 Hz, 1H), 6.97 (s, 1H), 6.91 – 6.94 (m, 2H), 5.21 (s, 2H), 3.82 (s, 3H). **^{13}C NMR** (126 MHz, $CDCl_3$) δ 159.9, 158.8, 157.8, 152.8, 135.6, 132.9, 130.6, 130.0, 128.3, 123.1, 122.1, 119.6, 116.3, 116.1, 114.1, 93.8, 67.2, 55.4. **HRMS** (ESI) m/z calcd. For $C_{21}H_{18}N_4O_3$ $[M+H]^+$: 375.1457, found 375.1469.

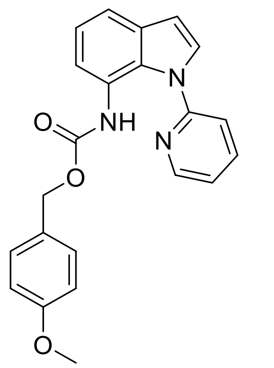

4-methoxybenzyl (1-(pyridin-2-yl)-1H-indol-7-yl)carbamate (**2i**) was prepared according to General Procedure B. Solvent DCE. Purification by automated flash column chromatography (0-70% EtOAc in heptane, 25g SiO_2). Compound **2i** (61.9 mg, 83%)was obtained as a light yellow wax.

^{1}H NMR (500 MHz, $CDCl_3$) δ 9.75 (s, 1H), 8.56 (ddd, *J* = 5.0, 1.9, 0.7 Hz, 1H), 7.89 (ddd, *J* = 8.2, 7.5, 2.0 Hz, 1H), 7.67 (d, *J* = 8.2 Hz, 1H), 7.55 – 7.59 (m, 1H), 7.5 – 7.52 (m, 1H), 7.36 – 7.39 (m, 2H), 7.23 (ddd, *J* = 7.4, 5.0, 0.9 Hz, 1H), 7.12 – 7.2 (m, 2H), 6.71 – 6.99 (m, 3H), 5.18 (s, 2H), 3.81 (s, 3H). **^{13}C NMR** (126 MHz, $CDCl_3$) δ 159.9, 152.9, 151.5, 148.9, 139.2, 134.8, 132.3, 130.5, 129.4, 128.2, 121.9, 121.4, 121.0, 120.3, 118.4, 114.1, 110.3, 92.0, 67.3, 55.4. **HRMS** (ESI) m/z calcd. For $C_{22}H_{19}N_3O_3$ $[M+H]^+$: 374.1504, found 374.1499.

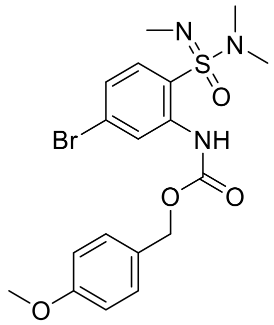

4-methoxybenzyl (5-bromo-2-(N,N,N'-trimethylsulfamidimidoyl)phenyl)carbamate (**2j**) was prepared according to General Procedure C. Solvent DCE, 0.2 mmol scale. Purification by automated flash column chromatography (0-80% EtOAc in heptane, 25g SiO_2). Compound **2j** (31.4 mg, 34%) was obtained as a colorless oil.

^{1}H NMR (500 MHz, $CDCl_3$) δ 11.65 (s, 1H), 8.64 (d, *J* = 1.8 Hz, 1H), 7.61 (d, *J* = 8.6 Hz, 1H), 7.32 – 7.36 (m, 2H), 7.23 (dd, *J* = 8.6, 1.9 Hz, 1H), 6.87 – 6.92 (m, 2H), 5.14 (d, *J* = 2.1 Hz, 2H), 3.81 (s, 3H), 2.87 (s, 3H), 2.61 (s, 6H). **^{13}C NMR** (126 MHz, $CDCl_3$) δ 159.8, 153.3, 138.9, 130.1, 129.7, 128.4, 128.3, 125.0, 123.0, 119.5, 114.0, 66.9, 55.4, 38.4, 27.7. **HRMS** (ESI) m/z calcd. For $C_{18}H_{22}BrN_3O_4S$ $[M+H]^+$: 456.0593, found 456.0598.

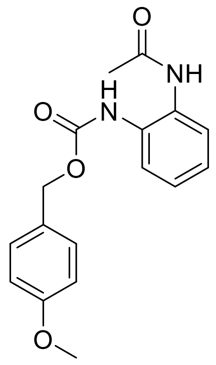

4-methoxybenzyl (2-acetamidophenyl)carbamate (**2k**) was prepared according to modified General Procedure C. Solvent DCE, 0.2 mmol scale, $MozN_3$ (41.4 mg, 0.2 mmol). Purification by automated flash column chromatography (0-80% EtOAc in heptane, 25g SiO_2). Compound **2k** (15.5 mg, 25%) was obtained as a colorless solid.

^{1}H NMR (500 MHz, $CDCl_3$) δ 8.12 (s, 1H), 7.39 – 7.53 (m, 2H), 7.34 – 7.37 (m, 2H), 7.26 (d, *J* = 7.5 Hz, 1H), 7.15 (td, *J* = 7.9, 1.4 Hz, 1H), 7.05 – 7.09 (m, 1H), 6.89 – 6.94 (m, 2H), 5.14 (s, 2H), 3.82 (s, 3H), 2.07 (s, 3H). **^{13}C NMR** (126 MHz, $CDCl_3$) δ 169.9, 159.8, 154.9, 130.9, 130.4, 129.6, 128.2, 126.6, 125.5, 125.3, 124.5, 114.0, 67.2, 55.4, 23.8. **HRMS** (ESI) m/z calcd. For $C_{17}H_{18}N_2O_4$ $[M+H]^+$: 315.1345, found 315.1360.

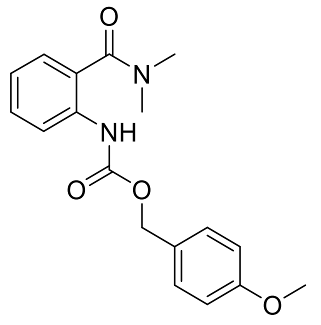

4-methoxybenzyl (2-(dimethylcarbamoyl)phenyl)carbamate (**2l**) was prepared according to General Procedure D. Purification by automated flash column chromatography (10-100% EtOAc in heptane, 25g SiO_2). Compound **2l** (35.1 mg, 53%) was obtained as a colorless oil.
^{1}H NMR (500 MHz, $CDCl_3$) δ 8.30 (s, 1H), 8.15 (d, *J* = 8.1 Hz, 1H), 7.33 – 7.4 (m, 3H), 7.18 – 7.21 (m, 1H), 7.03 (td, *J* = 7.5, 1.1 Hz, 1H), 6.87 – 6.92 (m, 2H), 5.11 (s, 2H), 3.81 (s, 3H), 3.08 (s, 3H), 2.99 (s, 3H). **^{13}C NMR** (126 MHz, $CDCl_3$) δ 170.2, 159.7, 153.7, 137.1, 130.8, 130.4, 128.3, 127.7, 123.7, 122.2, 121.0, 114.0, 66.8, 55.4, 40.0, 35.49. **HRMS** (ESI) m/z calcd. For $C_{18}H_{20}N_2O_4$ $[M+H]^+$: 329.1501, found 329.1508.

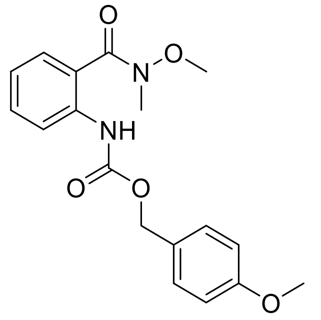

4-methoxybenzyl (2-(methoxy(methyl)carbamoyl)phenyl)carbamate (**2m**) was prepared according to General Procedure D. Purification by automated flash column chromatography (0-80% EtOAc in heptane, 25g SiO_2). Compound **2m** (19.5 mg, 28%) was obtained as a colorless wax.

^{1}H NMR (500 MHz, $CDCl_3$) δ 8.63 (s, 1H), 8.18 (d, *J* = 8.1 Hz, 1H), 7.47 – 7.51 (m, 1H), 7.39 – 7.43 (m, 1H), 7.35 (d, *J* = 8.7 Hz, 2H), 7.03 (td, *J* = 7.8, 1.0 Hz, 1H), 6.88 – 6.91 (m, 2H), 5.12 (s, 2H), 3.81 (s, 3H), 3.52 (s, 3H), 3.34 (s, 3H). **^{13}C NMR** (126 MHz, $CDCl_3$) δ 168.9, 159.8, 153.7, 137.7, 131.6, 130.4, 128.9, 128.3, 122.1, 121.4, 120.6, 114.0, 66.9, 61.5, 55.4, 34.1. **HRMS** (ESI) m/z calcd. For $C_{18}H_{20}N_2O_5$ $[M+H]^+$: 345.1450, found 345.1460.

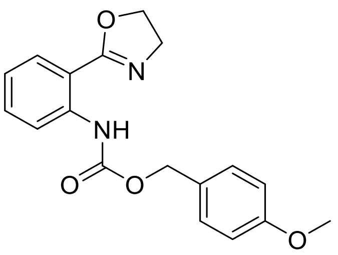

4-methoxybenzyl (2-(4,5-dihydrooxazol-2-yl)phenyl)carbamate (**2n**) was prepared according to general conditions E. Purification by preparative LC (pH 10, basic method). Compound **2n** (3.1 mg, 47%, 90% purity) was obtained as a colorless solid.

^{1}H NMR (500 MHz, DMSO) δ 11.76 (s, 1H), 8.34 – 8.38 (m, 1H), 7.81 (dd, *J* = 7.9, 1.5 Hz, 1H), 7.52 – 7.55 (m, 1H), 7.36 – 7.39 (m, 2H), 7.11 (td, *J* = 7.9, 1.1 Hz, 1H), 6.93 – 6.96 (m, 2H), 5.11 (s, 2H), 4.38 (t, *J* = 9.6 Hz, 2H), 4.07 (t, *J* = 9.6 Hz, 2H), 3.75 (s, 3H). **^{13}C NMR** (151 MHz, DMSO) δ 163.6, 159.3, 153.0, 139.4, 132.7, 130.4, 129.1, 128.2, 121.7, 117.4, 113.9, 112.2, 66.3, 66.1, 55.2, 54.3. **HRMS** (ESI) m/z calcd. For $C_{18}H_{18}N_2O_4$ $[M+H]^+$: 327.1345, found 327.1348.

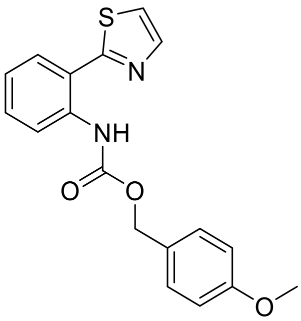

4-methoxybenzyl (2-(thiazol-2-yl)phenyl)carbamate (**2o**) was prepared according to general conditions E. Purification by preparative LC (pH 10, basic method). Compound **2o** (1.7 mg, 25%, >95% purity) was obtained as a colorless solid.

^{1}H NMR (500 MHz, DMSO) δ 11.51 (s, 1H), 8.30 (d, *J* = 8.3 Hz, 1H), 8.02 (d, *J* = 3.4 Hz, 1H), 7.90 (dd, *J* = 7.9, 1.4 Hz, 1H), 7.86 (d, *J* = 3.4 Hz, 1H), 7.48 (ddd, *J* = 8.6, 7.4, 1.5 Hz, 1H), 7.36 – 7.39 (m, 2H), 7.16 – 7.19 (m, 1H), 6.93 – 6.96 (m, 2H), 5.12 (s, 2H), 3.75 (s, 3H). **^{13}C NMR** (151 MHz, DMSO) δ 167.6, 159.3, 153.3, 142.8, 136.3, 131.0, 130.3, 129.1, 128.3, 123.2, 120.4, 119.5, 119.4, 113.9, 66.1, 55.2. **HRMS** (ESI) m/z calcd. For $C_{18}H_{16}N_2O_3S$ $[M+H]^+$: 341.0960, found 341.0966.

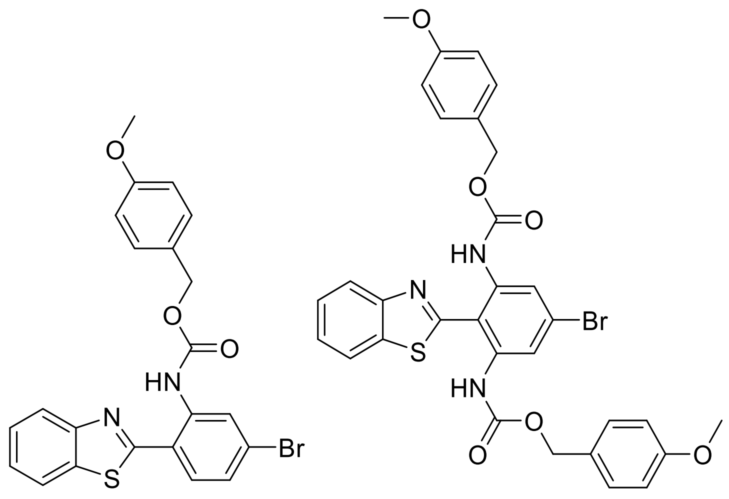

4-methoxybenzyl (2-(benzo[d]thiazol-2-yl)-5-bromophenyl)carbamate (**2p**) and bis(4-methoxybenzyl) (2-(benzo[d]thiazol-2-yl)-5-bromo-1,3-phenylene)dicarbamate (**2p´**) were prepared according to general conditions E. Purification by preparative LC (pH 10, basic method). Compound **2p** (1.1 mg, 12%, >95% purity) was obtained as a colorless solid. Compound **2p´** (0.9 mg, 7%, >95% purity) was obtained as a colorless solid.

2p

^{1}H NMR (500 MHz, DMSO) δ 11.66 (s, 1H), 8.53 (d, *J* = 1.9 Hz, 1H), 8.18 (d, *J* = 7.7 Hz, 1H), 7.99 (d, *J* = 7.8 Hz, 1H), 7.93 (d, *J* = 8.5 Hz, 1H), 7.56 – 7.6 (m, 1H), 7.52 (t, *J* = 7.5 Hz, 1H), 7.38 – 7.45 (m, 3H), 6.94 – 6.97 (m, 2H), 5.17 (s, 2H), 3.76 (s, 3H). **^{13}C NMR** (151 MHz, DMSO) δ 167.0, 159.3, 152.0, 133.0, 131.8, 130.0, 128.2, 127.1, 126.2, 125.2, 122.5, 122.3, 122.1, 113.9, 66.4, 55.1. **HRMS** (ESI) m/z calcd. For $C_{22}H_{17}BrN_2O_3S$ $[M+H]^+$: 469.0221, found 469.0221.

2p´

^{1}H NMR (500 MHz, DMSO) δ 10.57 (s, 2H), 8.12 (d, *J* = 7.4 Hz, 1H), 8.01 (d, *J* = 8.0 Hz, 1H), 7.87 (s, 2H), 7.58 (td, *J* = 8.2, 7.7, 1.2 Hz, 1H), 7.5 – 7.53 (m, 1H), 7.29 (d, *J* = 8.5 Hz, 4H), 6.88 (d, *J* = 8.6 Hz, 4H), 5.05 (s, 4H), 3.73 (s, 6H). **^{13}C NMR** (151 MHz, DMSO) δ 159.1, 153.8, 150.4, 134.3, 129.9, 128.2, 126.7, 126.0, 123.7, 122.3, 122.0, 113.8, 66.2, 55.1. **HRMS** (ESI) m/z calcd. For $C_{31}H_{26}BrN_3O_6S$ $[M+H]^+$: 648.0804, found 648.0828.

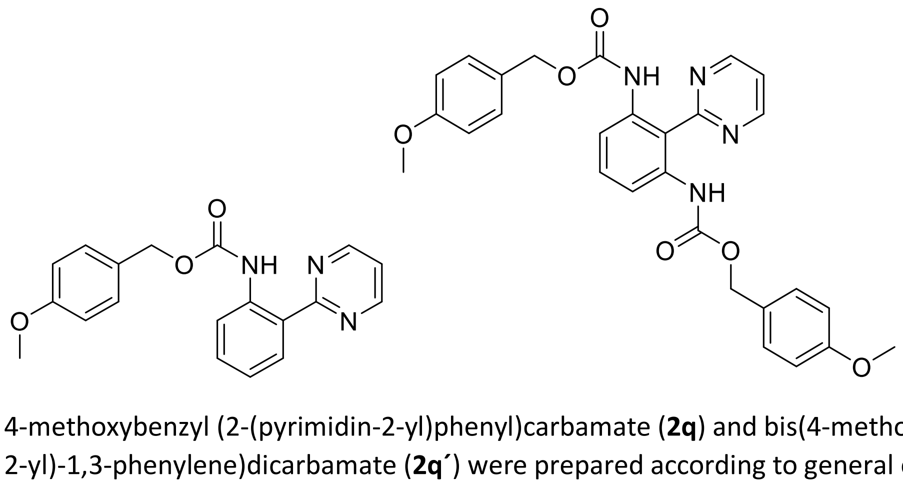

4-methoxybenzyl (2-(pyrimidin-2-yl)phenyl)carbamate (**2q**) and bis(4-methoxybenzyl) (2-(pyrimidin-2-yl)-1,3-phenylene)dicarbamate (**2q´**) were prepared according to general conditions E. Purification by preparative LC (pH 10, basic method). Compound **2q** (0.5 mg, 7%, 90% purity in pH10, >95%

purity at pH 3) was obtained as a yellow wax. Compound **2q´** (3.3 mg, 32%, >95% purity) was obtained as a yellow solid.

2q

^{1}H NMR (500 MHz, DMSO) δ 12.00 (s, 1H), 8.98 (d, *J* = 4.9 Hz, 2H), 8.50 (dd, *J* = 8.1, 1.5 Hz, 1H), 8.32 (dd, *J* = 8.4, 0.9 Hz, 1H), 7.48 – 7.54 (m, 2H), 7.36 – 7.4 (m, 2H), 7.18 (ddd, *J* = 8.1, 7.2, 1.2 Hz, 1H), 6.93 – 6.96 (m, 2H), 5.12 (s, 2H), 3.75 (s, 3H). **^{13}C NMR** (151 MHz, DMSO) δ 163.7, 159.2, 157.3, 153.1, 139.1, 131.9, 130.4, 130.1, 128.4, 122.3, 119.5, 119.1, 113.9, 65.9, 55.1. **HRMS** (ESI) m/z calcd. For $C_{19}H_{17}N_3O_3$ [M+H]$^+$: 336.1348, found 336.1352.

2q´

^{1}H NMR (500 MHz, DMSO) δ 10.00 (s, 2H), 8.93 (d, *J* = 5.0 Hz, 2H), 7.61 (d, *J* = 8.2 Hz, 2H), 7.46 (t, *J* = 5.0 Hz, 1H), 7.43 (t, *J* = 8.2 Hz, 1H), 7.26 – 7.3 (m, 4H), 6.89 – 6.93 (m, 4H), 4.97 (s, 4H), 3.74 (s, 6H). **^{13}C NMR** (151 MHz, DMSO) δ 163.11, 159.14, 157.19, 153.33, 137.45, 130.15, 129.92, 128.41, 119.51, 119.02, 117.49, 113.81, 65.71, 55.12. **HRMS** (ESI) m/z calcd. For $C_{28}H_{26}N_4O_6$ [M+H]$^+$: 515.1931, found 515.1944.

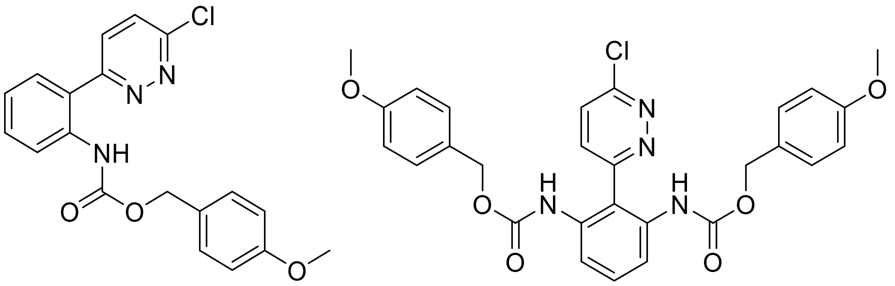

4-methoxybenzyl (2-(6-chloropyridazin-3-yl)phenyl)carbamate (**2r**) and bis(4-methoxybenzyl) (2-(6-chloropyridazin-3-yl)-1,3-phenylene)dicarbamate (**2r´**) were prepared according to general conditions E. Purification by preparative LC (pH 3, FP column, acidic method).Compound **2r** (0.6 mg, 8%, 82% purity at pH 10, 87% purity at pH 3) was obtained as a light yellow wax. Compound **2r´** (3.2 mg, 29%, >95% purity) was obtained as a yellow solid.

2r

^{1}H NMR (500 MHz, DMSO) δ 10.10 (s, 1H), 8.07 (d, *J* = 9.0 Hz, 1H), 7.99 (d, *J* = 9.0 Hz, 1H), 7.85 (d, *J* = 8.8 Hz, 1H), 7.71 (dd, *J* = 7.8, 1.5 Hz, 1H), 7.5 – 7.54 (m, 1H), 7.31 (dd, *J* = 7.6, 1.1 Hz, 1H), 7.26 – 7.29 (m, 2H), 6.91 – 6.93 (m, 2H), 4.99 (s, 2H), 3.75 (s, 3H). **^{13}C NMR** (151 MHz, DMSO) δ 159.8, 159.2, 154.8, 153.6, 136.5, 130.7, 130.36, 130.35, 130.0, 129.50, 129.47, 129.4, 128.4, 124.4, 113.8, 65.8, 55.1. **HRMS** (ESI) m/z calcd. For $C_{19}H_{16}ClN_3O_3$ [M+H]$^+$: 370.0958, found 370.0965.

2r´

^{1}H NMR (500 MHz, DMSO) δ 9.06 (s, 2H), 7.85 (d, *J* = 8.9 Hz, 1H), 7.61 (d, *J* = 8.9 Hz, 1H), 7.44 – 7.48 (m, 1H), 7.37 – 7.43 (m, 2H), 7.16 – 7.2 (m, 4H), 6.89 – 6.92 (m, 4H), 4.91 (s, 4H), 3.75 (s, 6H). **^{13}C NMR** (151 MHz, DMSO) δ 159.1, 157.5, 154.6, 154.0, 136.6, 132.8, 130.4, 129.7, 129.4, 128.5, 125.5, 121.9, 113.8, 65.6, 55.1. **HRMS** (ESI) m/z calcd. For $C_{28}H_{25}ClN_4O_6$ [M+H]$^+$: 547.1384, found 547.1373.

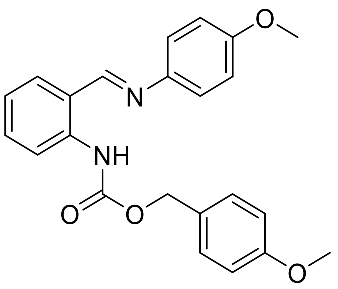

4-methoxybenzyl (*E*)-(2-(((4-methoxyphenyl)imino)methyl)phenyl)carbamate (**2s**) was prepared according to general conditions E. Purification by preparative LC (pH 10, basic method). Compound **2s** (1.2 mg, 16%, 90% purity at pH 10, 88% purity at pH 3) was obtained as yellow wax.

^{1}H NMR (500 MHz, DMSO) δ 12.27 (s, 1H), 8.82 (s, 1H), 8.26 (d, *J* = 8.3 Hz, 1H), 7.74 (dd, *J* = 7.7, 1.6 Hz, 1H), 7.47 – 7.52 (m, 1H), 7.36 – 7.4 (m, 2H), 7.3 – 7.33 (m, 2H), 7.18 (td, *J* = 7.5, 1.1 Hz, 1H), 7.01 – 7.04 (m, 2H), 6.93 – 6.96 (m, 2H), 5.14 (s, 2H), 3.79 (s, 3H), 3.75 (s, 3H). **^{13}C NMR** (126 MHz, DMSO) δ 161.0, 159.2, 158.5, 153.3, 142.1, 139.4, 134.1, 131.9, 129.9, 128.4, 122.5, 122.2, 121.2, 117.9, 114.7, 113.9, 65.9, 55.4, 55.1. **HRMS** (ESI) m/z calcd. For $C_{23}H_{22}N_2O_4$ $[M+H]^+$: 391.1658, found 391.1659.

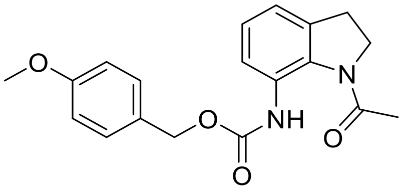

4-methoxybenzyl (1-acetylindolin-7-yl)carbamate (**2t**) was prepared according to general conditions E. Purification by preparative LC (pH 10, basic method). Compound **2t** (1.2 mg, 16%, >95% purity) was obtained as colorless solid.

^{1}H NMR (500 MHz, DMSO) δ 9.86 (s, 1H), 7.64 (d, *J* = 7.9 Hz, 1H), 7.32 – 7.36 (m, 2H), 7.11 – 7.15 (m, 1H), 7.01 (dd, *J* = 7.3, 1.1 Hz, 1H), 6.92 – 6.95 (m, 2H), 5.03 (s, 2H), 4.10 (t, *J* = 7.9 Hz, 2H), 3.75 (s, 3H), 3.04 (t, *J* = 7.9 Hz, 2H), 2.27 (s, 3H). **^{13}C NMR** (151 MHz, DMSO) δ 170.0, 159.2, 153.0, 135.9, 132.6, 132.0, 130.2, 128.5, 127.5, 125.8, 119.8, 113.9, 65.7, 55.1, 51.2, 28.5, 24.3. **HRMS** (ESI) m/z calcd. For $C_{19}H_{20}N_2O_4$ $[M+H]^+$: 339.1345, found 339.1349.

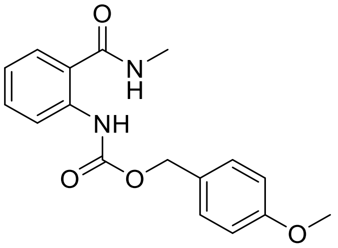

4-methoxybenzyl (2-(methylcarbamoyl)phenyl)carbamate (**2u**) was prepared according to general conditions E. Purification by preparative LC (pH 10, basic method). Compound **2u** (0.7 mg, 11%, >95% purity) was obtained as colorless solid.

^{1}H NMR (500 MHz, DMSO) δ 11.05 (s, 1H), 8.73 (s, 1H), 8.20 (d, *J* = 7.7 Hz, 1H), 7.70 (dd, *J* = 7.9, 1.4 Hz, 1H), 7.47 – 7.51 (m, 1H), 7.34 – 7.37 (m, 2H), 7.09 (td, *J* = 7.8, 1.2 Hz, 1H), 6.93 – 6.96 (m, 2H), 5.07 (s, 2H), 3.75 (s, 3H), 2.76 (d, *J* = 4.5 Hz, 3H). **^{13}C NMR** (151 MHz, DMSO) δ 169.12, 159.71, 153.26, 139.59, 135.29, 132.57, 130.64, 128.70, 128.43, 120.03, 119.05, 114.34, 66.40, 55.59, 26.67. **HRMS** (ESI) m/z calcd. For $C_{17}H_{18}N_2O_4$ $[M+H]^+$: 315.1345, found 315.1351.

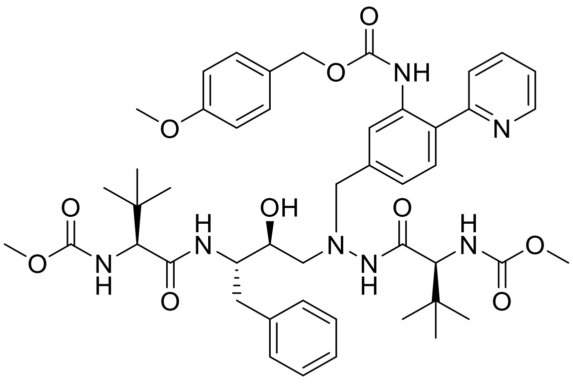

Methyl ((5S,10S,11S,14S)-11-benzyl-5-(tert-butyl)-10-hydroxy-8-(3-((((4-methoxybenzyl)oxy)carbonyl)amino)-4-(pyridin-2-yl)benzyl)-15,15-dimethyl-3,6,13-trioxo-2-oxa-4,7,8,12-tetraazahexadecan-14-yl)carbamate (**3a**) was prepared based on modified General Procedure A. [Cp*Ir(H_2O)$_3$]SO_4 (4.9 mg, 0.01 mmol, 2 mol%), MozN_3 (103.6 mg, 0.5 mmol). Purification by preparative reverse phase HPLC (44-84% MeCN in NH_3 buffer) to give compound **3a** (356.6 mg, 81%) as a yellow solid.

^{1}H NMR (500 MHz, DMSO) δ 11.61 (s, 1H), 9.14 (s, 1H), 8.64 (ddd, *J* = 4.9, 1.8, 0.9 Hz, 1H), 8.10 (s, 1H), 7.96 (td, *J* = 7.8, 1.8 Hz, 1H), 7.87 (d, *J* = 8.3 Hz, 1H), 7.70 (d, *J* = 8.2 Hz, 1H), 7.55 (d, *J* = 9.1 Hz, 1H), 7.39 (ddd, *J* = 7.5, 5.0, 1.0 Hz, 1H), 7.32 – 7.36 (m, 2H), 7.16 – 7.24 (m, 6H), 7.1 – 7.15 (m, 1H), 7.00 (d, *J* = 9.5 Hz, 1H), 6.91 – 6.95 (m, 2H), 6.86 (d, *J* = 9.4 Hz, 1H), 5.06 (s, 2H), 5.02 (s, 1H), 3.9 – 3.99 (m, 2H), 3.84 (d, *J* = 9.4 Hz, 1H), 3.74 (d, *J* = 1.3 Hz, 3H), 3.64 (d, *J* = 9.4 Hz, 1H), 3.49 – 3.55 (m, 6H), 2.69 – 2.84 (m, 3H), 0.74 (s, 9H), 0.62 (s, 9H). **^{13}C NMR** (126 MHz, DMSO) δ 170.2, 170.0, 159.2, 157.1, 156.5, 153.0, 147.7, 139.6, 139.0, 138.3, 136.9, 130.0, 129.8, 129.1, 129.0, 128.6, 128.0, 125.8, 124.1, 123.2, 122.9, 122.3, 120.3, 113.9, 113.8, 68.0, 65.6, 63.1, 61.3, 61.1, 60.7, 55.1, 51.6, 51.4, 40.4, 37.7, 33.6, 33.4, 26.7, 26.2. **HRMS** (ESI) m/z calcd. For $C_{47}H_{61}N_7O_{10}$ $[M+H]^+$: 884.4558, found 884.4525.

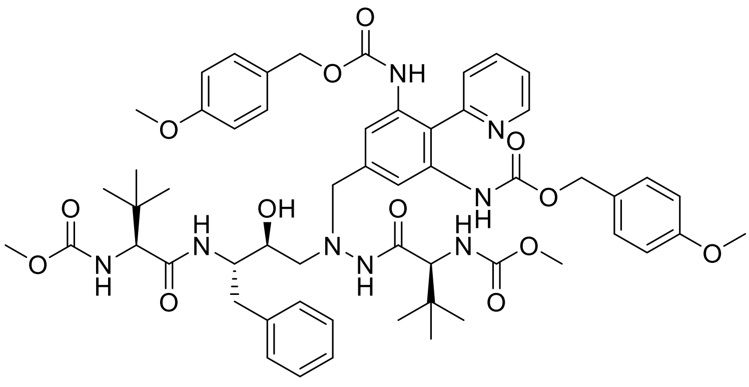

Bis(4-methoxybenzyl) (5-((5S,8S,9S)-8-benzyl-5-(tert-butyl)-9-hydroxy-11-((S)-2-((methoxycarbonyl)amino)-3,3-dimethylbutanamido)-3,6-dioxo-2-oxa-4,7,11-triazadodecan-12-yl)-2-(pyridin-2-yl)-1,3-phenylene)dicarbamate (**3a´**) was prepared according to modified General Procedure B. [Cp*Ir(H_2O)$_3$]SO_4 (5.9 mg, 12.0 µmol, 6 mol%), MozN_3 (124.3 mg, 0.6 mmol), DCE solvent. Purification by preparative reverse phase HPLC (55-95% MeCN in NH_3 buffer) to give compound **3a´** (154.3 mg, 73%) as a yellow solid.

^{1}H NMR (500 MHz, DMSO) δ 9.30 (s, 2H), 9.19 (s, 1H), 8.67 (d, *J* = 4.6 Hz, 1H), 7.78 (td, *J* = 7.8, 1.7 Hz, 1H), 7.57 (d, *J* = 8.9 Hz, 1H), 7.47 (s, 2H), 7.32 – 7.38 (m, 2H), 7.18 – 7.24 (m, 8H), 7.12 – 7.15 (m, 1H), 7.02 (d, *J* = 9.3 Hz, 1H), 6.85 – 6.92 (m, 5H), 5.05 (s, 1H), 4.91 (s, 4H), 3.96 – 4.01 (m, 1H), 3.89 – 3.96 (m, 2H), 3.86 (d, *J* = 9.4 Hz, 1H), 3.74 (s, 6H), 3.66 (d, *J* = 9.3 Hz, 1H), 3.57 – 3.62 (m, 1H), 2.71 – 2.85 (m, 3H), 2.61 (d, *J* = 9.9 Hz, 1H), 0.76 (s, 9H), 0.64 (s, 9H). **^{13}C NMR** (126 MHz, DMSO) δ 170.3, 170.1, 159.1, 156.6, 154.7, 153.5, 148.7, 139.0, 138.7, 137.0, 135.9, 129.8, 129.51, 129.49, 129.1, 128.5, 128.0, 125.9, 125.6, 122.3, 120.6, 119.9, 114.8, 113.82, 113.78, 68.8, 67.8, 65.5, 63.1, 61.3, 61.1, 60.7, 55.1, 51.8, 51.5, 51.5, 37.7, 33.7, 33.4, 26.6, 26.3. **HRMS** (ESI) m/z calcd. For $C_{56}H_{70}N_8O_{13}$ $[M+H]^+$: 1063.5140, found 1063.5129.

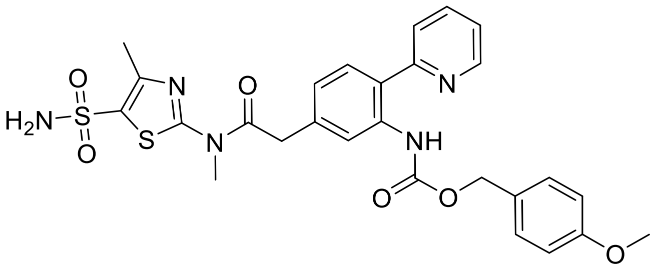

4-methoxybenzyl (5-(2-(methyl(4-methyl-5-sulfamoylthiazol-2-yl)amino)-2-oxoethyl)-2-(pyridin-2-yl)phenyl)carbamate (**3b**) was prepared based on General Procedure B. $[Cp^*Ir(H_2O)_3]SO_4$ (5.9 mg, 12.0 µmol, 6 mol%), $MozN_3$ (45.6 mg, 0.22 mmol), solvent DCE. Purification by preparative reverse phase HPLC (36-76% MeCN in HCOOH buffer) to give compound **3b** (56.9 mg, 49%) as a colorless solid.

^{1}H NMR (500 MHz, DMSO) δ 11.62 (s, 1H), 8.67 (ddd, *J* = 4.9, 1.8, 0.9 Hz, 1H), 8.10 (s, 1H), 7.98 (td, *J* = 7.8, 1.8 Hz, 1H), 7.93 (d, *J* = 8.2 Hz, 1H), 7.79 (d, *J* = 8.1 Hz, 1H), 7.66 (s, 2H), 7.41 (ddd, *J* = 7.4, 4.9, 1.2 Hz, 1H), 7.31 – 7.36 (m, 2H), 7.09 (dd, *J* = 8.1, 1.7 Hz, 1H), 6.91 – 6.95 (m, 2H), 5.06 (s, 2H), 4.23 (s, 2H), 3.74 (s, 3H), 3.71 (s, 3H), 2.48 (s, 3H). **^{13}C NMR** (151 MHz, DMSO) δ 171.6, 159.2, 158.5, 156.9, 153.1, 148.2, 147.8, 138.3, 137.1, 136.1, 130.0, 129.4, 128.5, 128.3, 124.6, 124.1, 123.0, 122.5, 121.4, 113.9, 65.8, 55.1, 40.5, 34.3, 16.2. **HRMS** (ESI) m/z calcd. For $C_{27}H_{27}N_5O_6S_2$ $[M+H]^+$: 582.1481, found 582.1475.

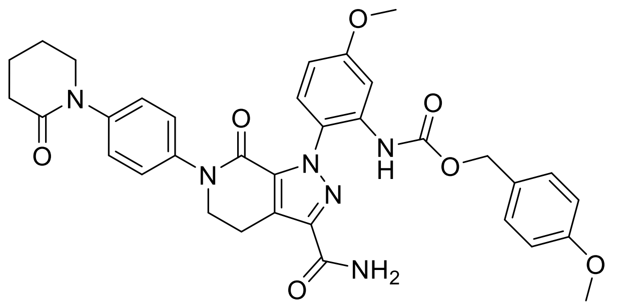

4-methoxybenzyl (2-(3-carbamoyl-7-oxo-6-(4-(2-oxopiperidin-1-yl)phenyl)-4,5,6,7-tetrahydro-1H-pyrazolo[3,4-c]pyridin-1-yl)-5-methoxyphenyl)carbamate (**3c**) was prepared based on General Procedure C. $MozN_3$ (20.7 mg, 0.1 mmol), solvent NMP. Purification by preparative reverse phase HPLC (34-74% MeCN in NH_3 buffer) to give compound **3c** (14.6 mg, 23%) as a yellow solid, as well as recovered starting material (28.4 mg, 62%).

^{1}H NMR (500 MHz, DMSO) δ 8.91 (s, 1H), 7.77 (s, 1H), 7.40 (s, 1H), 7.35 (s, 1H), 7.23 – 7.3 (m, 7H), 6.89 – 6.93 (m, 2H), 6.73 (dd, *J* = 8.8, 2.8 Hz, 1H), 5.00 (s, 2H), 3.96 (t, *J* = 6.5 Hz, 2H), 3.77 (s, 3H), 3.74 (s, 3H), 3.57 (t, *J* = 5.6 Hz, 2H), 3.17 (t, *J* = 6.6 Hz, 2H), 2.37 (t, *J* = 6.4 Hz, 2H), 1.84 (qt, *J* = 10.8, 5.3 Hz,

4H). **^{13}C NMR** (126 MHz, DMSO) δ 168.8, 163.3, 159.5, 159.2, 156.6, 153.7, 141.9, 141.2, 139.7, 135.2, 134.7, 130.0, 129.2, 128.3, 126.3, 125.7, 124.7, 124.5, 113.8, 108.3, 65.8, 55.5, 55.1, 50.9, 50.8, 32.6, 23.0, 21.1, 20.9. **HRMS** (ESI) m/z calcd. For $C_{34}H_{34}N_6O_7$ $[M-H]^-$: 637.2411, found 637.2383.

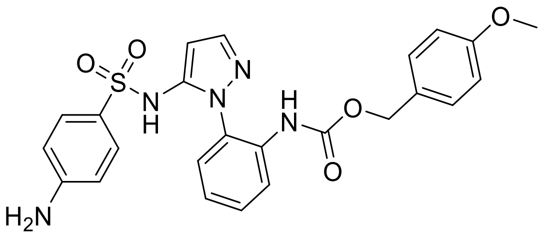

4-methoxybenzyl (2-(5-((4-aminophenyl)sulfonamido)-1H-pyrazol-1-yl)phenyl)carbamate (**3d**) was prepared based on General Procedure B. $MozN_3$ (45.6 mg, 0.22 mmol), solvent DCE. Purification by preparative reverse phase HPLC (38-78% MeCN in HCOOH buffer) to give compound **3d** (46.6 mg, 47%) as a colorless solid, as well as recovered starting material (16.8 mg, 27%).

^{1}H NMR (500 MHz, DMSO) δ 9.88 (s, 1H), 8.02 (s, 1H), 7.98 (d, *J* = 8.2 Hz, 1H), 7.65 (d, *J* = 2.0 Hz, 1H), 7.44 – 7.48 (m, 1H), 7.29 – 7.34 (m, 4H), 7.17 (td, *J* = 7.7, 1.3 Hz, 1H), 7.11 (dd, *J* = 7.9, 1.5 Hz, 1H), 6.9 – 6.94 (m, 2H), 6.56 – 6.6 (m, 2H), 6.10 (s, 2H), 5.88 (d, *J* = 1.9 Hz, 1H), 5.03 (s, 2H), 3.74 (s, 3H). **^{13}C NMR** (126 MHz, DMSO) δ 159.2, 153.3, 152.8, 140.7, 137.0, 133.9, 130.1, 129.2, 128.9, 128.1, 127.9, 127.4, 124.0, 123.4, 121.5, 113.9, 112.6, 101.6, 66.1, 55.1. **HRMS** (ESI) m/z calcd. For $C_{24}H_{23}N_5O_5S$ $[M+H]^+$: 494.1498, found 494.1497.

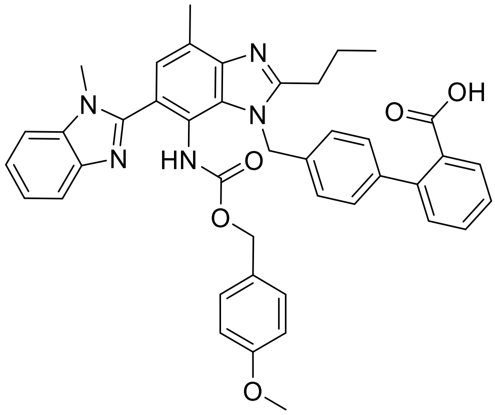

4'-((4'-((((4-methoxybenzyl)oxy)carbonyl)amino)-1,7'-dimethyl-2'-propyl-1H,3'H-[2,5'-bibenzo[d]imidazol]-3'-yl)methyl)-[1,1'-biphenyl]-2-carboxylic acid (**3e**) was prepared based on General Procedure C. Scale 0.2 mmol, $MozN_3$ (41.4 mg, 0.2 mmol), NMP solvent. Purification by preparative reverse phase HPLC (28-68% MeCN in NH_3 buffer) to give compound **3e** (27.5 mg, 20%) as a colorless solid, as well as recovered starting material (69.0 mg, 67%).

^{1}H NMR (500 MHz, MeOD) δ 7.72 (dd, *J* = 7.7, 1.1 Hz, 1H), 7.66 (d, *J* = 7.6 Hz, 1H), 7.45 – 7.49 (m, 2H), 7.32 – 7.4 (m, 4H), 7.27 – 7.31 (m, 3H), 7.11 (d, *J* = 8.2 Hz, 2H), 6.88 – 6.94 (m, 2H), 6.61 (d, *J* = 8.2 Hz, 2H), 5.55 (s, 2H), 4.83 (s, 2H), 3.75 (s, 3H), 3.45 (s, 3H), 2.99 – 3.03 (m, 2H), 2.60 (s, 3H), 1.83 (h, *J* = 7.4 Hz, 2H), 1.04 (t, *J* = 7.4 Hz, 3H). **^{13}C NMR** (126 MHz, MeOD) δ 173.4, 160.8, 159.5, 157.3, 153.5, 144.5, 142.7, 142.1, 141.0, 136.3, 135.0, 134.3, 131.6, 131.4, 130.7, 130.5, 130.0, 128.3, 127.4, 124.6, 124.3, 118.9, 114.7, 112.0, 111.8, 67.3, 64.9, 55.7, 48.1, 30.3, 22.8, 14.2, 12.6. **HRMS** (ESI) m/z calcd. For $C_{42}H_{39}N_5O_5$ $[M-H]^-$: 692.2873, found 692.2854.

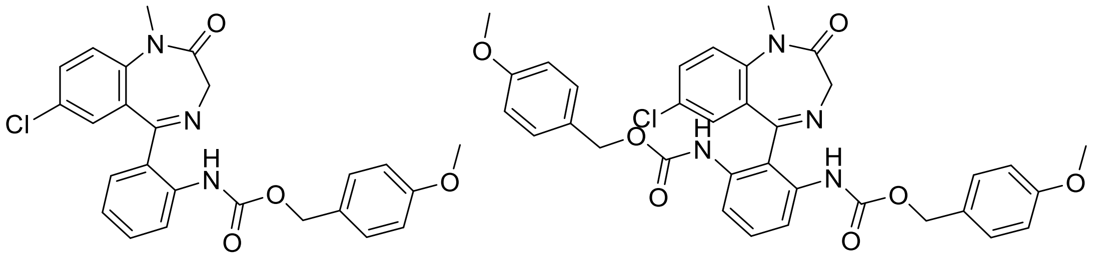

4-methoxybenzyl (2-(7-chloro-1-methyl-2-oxo-2,3-dihydro-1H-benzo[e][1,4]diazepin-5-yl)phenyl)carbamate (**3f**) and bis(4-methoxybenzyl) (2-(7-chloro-1-methyl-2-oxo-2,3-dihydro-1H-benzo[e][1,4]diazepin-5-yl)-1,3-phenylene)dicarbamate (**3f´**) were prepared based on General Procedure E. Solvent DCE. Purification by preparative LC (pH 3, HSS column, acidic method). Compound **3f** (3.2 mg, 34%, 90% purity) was obtained as a colorless solid, and compound **3f´** (3.2 mg, 25%, >95% purity) was obtained as a colorless solid.

3f

^{1}H NMR (500 MHz, DMSO) δ 10.28 (s, 1H), 7.86 (d, *J* = 8.1 Hz, 1H), 7.66 (dd, *J* = 8.9, 2.5 Hz, 1H), 7.56 (d, *J* = 8.9 Hz, 1H), 7.48 (ddd, *J* = 8.4, 7.2, 1.8 Hz, 1H), 7.29 (d, *J* = 8.7 Hz, 2H), 7.18 (dd, *J* = 7.8, 1.6 Hz, 1H), 7.13 – 7.16 (m, 1H), 7.11 (d, *J* = 2.5 Hz, 1H), 6.9 – 6.95 (m, 2H), 4.97 (s, 2H), 4.53 (d, *J* = 11.1 Hz, 1H), 3.81 (d, *J* = 11.0 Hz, 1H), 3.75 (s, 3H), 3.29 (s, 3H). **^{13}C NMR** (151 MHz, DMSO) δ 169.2, 168.8, 159.2, 153.4, 142.4, 137.8, 132.2, 131.4, 131.0, 130.2, 129.9, 128.8, 128.3, 127.9, 127.8, 123.8, 123.2, 122.1, 113.8, 65.7, 56.3, 55.1, 34.4. **HRMS** (ESI) m/z calcd. For $C_{25}H_{22}ClN_3O_4$ $[M+H]^+$: 464.1377, found 464.1385.

3f´

^{1}H NMR (500 MHz, DMSO) δ 9.36 (s, 1H), 8.52 (s, 1H), 7.73 (s, 1H), 7.50 (d, *J* = 7.4 Hz, 1H), 7.37 – 7.45 (m, 2H), 7.31 (d, *J* = 6.9 Hz, 2H), 7.12 (s, 2H), 6.95 (s, 1H), 6.90 (d, *J* = 8.3 Hz, 4H), 6.85 (d, *J* = 2.3 Hz, 1H), 5.03 (s, 2H), 4.62 (s, 2H), 4.48 (d, *J* = 10.4 Hz, 1H), 3.80 (d, *J* = 10.5 Hz, 1H), 3.74 (s, 6H), 3.17 (s, 3H). **^{13}C NMR** (151 MHz, DMSO) δ 168.4, 166.1, 159.1, 153.8, 153.7, 141.7, 137.1, 131.4, 130.6, 130.1, 130.0, 128.4, 128.3, 127.5, 123.3, 119.8, 113.8, 65.8, 65.4, 56.4, 55.1, 34.6. **HRMS** (ESI) m/z calcd. For $C_{34}H_{31}ClN_4O_7$ $[M+H]^+$: 643.1959, found 643.1971.

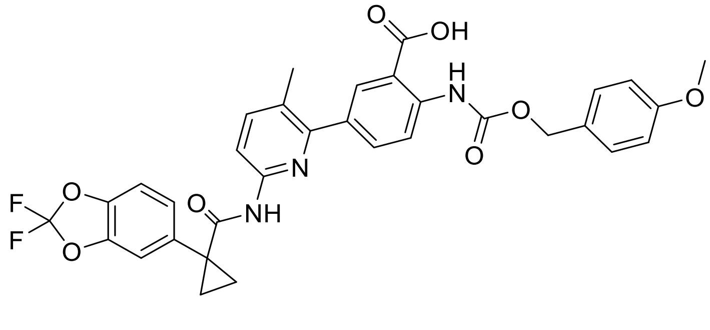

5-(6-(1-(2,2-difluorobenzo[d][1,3]dioxol-5-yl)cyclopropane-1-carboxamido)-3-methylpyridin-2-yl)-2-((((4-methoxybenzyl)oxy)carbonyl)amino)benzoic acid (**3g**) was prepared according to General Procedure C. Scale 0.2 mmol, $MozN_3$ (41.4 mg, 0.2 mmol), NMP solvent. Purification by preparative reverse phase HPLC (28-68% MeCN in NH_3 buffer) to give compound **3g** (20.0 mg, 16%) as a light brown solid, as well as recovered starting material (66.6 mg, 74%).

^{1}H NMR (500 MHz, MeOD) δ 8 – 8.03 (m, 2H), 7.88 (d, *J* = 8.6 Hz, 1H), 7.81 (d, *J* = 2.0 Hz, 1H), 7.70 (d, *J* = 8.7 Hz, 1H), 7.29 (d, *J* = 1.6 Hz, 1H), 7.2 – 7.25 (m, 3H), 7.13 (d, *J* = 8.2 Hz, 1H), 6.87 – 6.89 (m, 2H), 5.00 (s, 2H), 3.79 (s, 3H), 2.07 (s, 3H), 1.64 (q, *J* = 3.9 Hz, 2H), 1.20 (q, *J* = 4.0 Hz, 2H). **^{13}C NMR** (126 MHz, MeOD) δ 174.0, 170.2, 161.2, 155.6, 154.6, 150.2, 145.3, 144.8, 142.7, 140.7, 136.5, 132.7, 131.5, 131.1, 130.0, 129.7, 129.0, 128.2, 123.1, 114.9, 113.5, 111.2, 67.8, 55.7, 40.4, 32.3, 18.3, 17.6. **^{19}F NMR** (471 MHz, MeOD) δ -51.83. **HRMS** (ESI) m/z calcd. For $C_{33}H_{27}F_2N_3O_8$ $[M+H]^+$: 632.1844, found 632.1871.

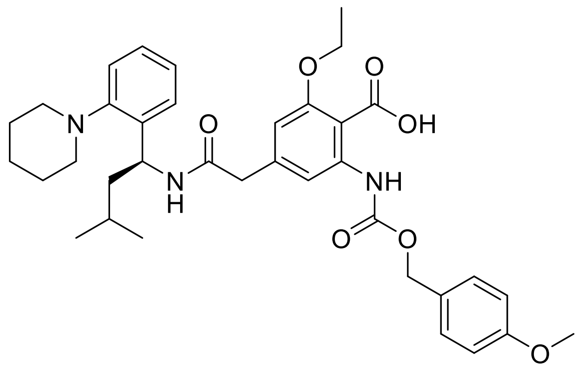

(S)-2-ethoxy-6-((((4-methoxybenzyl)oxy)carbonyl)amino)-4-(2-((3-methyl-1-(2-(piperidin-1-yl)phenyl)butyl)amino)-2-oxoethyl)benzoic acid (**3h**) was prepared according to General Procedure E. Solvent NMP. Purification by preparative LC (pH 10, basic method). Compound **3h** (2.9 mg, 23%, >95% purity) was obtained as a light brown solid.

^{1}H NMR (500 MHz, DMSO) δ 9.38 (s, 1H), 8.45 (d, *J* = 8.5 Hz, 1H), 7.32 – 7.35 (m, 2H), 7.31 (dd, *J* = 7.7, 1.5 Hz, 1H), 7.27 (s, 1H), 7.13 – 7.16 (m, 1H), 7.08 (dd, *J* = 7.9, 1.1 Hz, 1H), 6.99 – 7.03 (m, 1H), 6.91 – 6.95 (m, 2H), 6.69 (s, 1H), 5.35 (td, *J* = 9.4, 4.8 Hz, 1H), 5.04 (s, 2H), 3.97 (q, *J* = 6.9 Hz, 2H), 3.75 (s, 3H), 3.41 (s, 2H), 3.08 (s, 2H), 2.54 (s, 2H), 1.62 – 1.72 (m, 2H), 1.44 – 1.62 (m, 6H), 1.26 – 1.34 (m, 4H), 0.88 – 0.91 (m, 6H). **^{13}C NMR** (151 MHz, DMSO) δ 168.7, 167.7, 159.2, 156.7, 153.3, 151.5, 140.5, 140.1, 137.2, 129.9, 128.4, 127.2, 126.0, 124.0, 120.5, 114.5, 113.8, 109.0, 65.8, 64.2, 55.1, 46.5, 46.0, 42.8, 26.3, 24.9, 23.9, 23.2, 21.8, 14.6. **HRMS** (ESI) m/z calcd. For $C_{36}H_{45}N_3O_7$ $[M+H]^+$: 632.3336, found 632.3359.

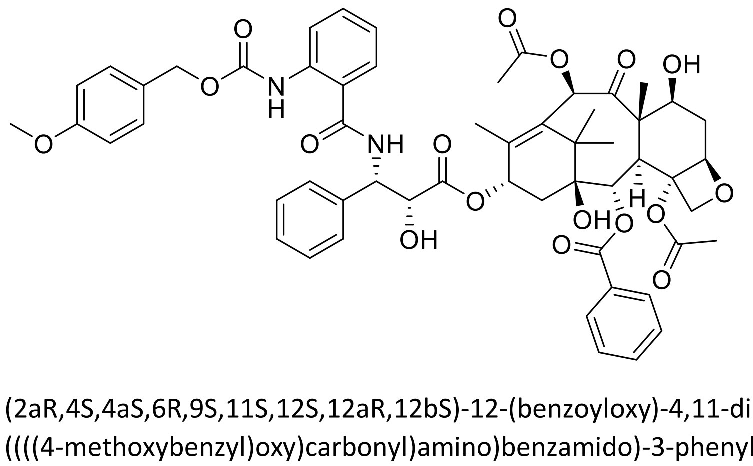

(2aR,4S,4aS,6R,9S,11S,12S,12aR,12bS)-12-(benzoyloxy)-4,11-dihydroxy-9-(((2R,3S)-2-hydroxy-3-(2-((((4-methoxybenzyl)oxy)carbonyl)amino)benzamido)-3-phenylpropanoyl)oxy)-4a,8,13,13-tetramethyl-5-oxo-3,4,4a,5,6,9,10,11,12,12a-decahydro-1H-7,11-methanocyclodeca[3,4]benzo[1,2-b]oxete-6,12b(2aH)-diyl diacetate (**3i**) was prepared according to General Procedure C. $MozN_3$ (20.7

mg, 0.1 mmol), solvent DCE. Purification by preparative reverse phase HPLC (43-83% MeCN in HCOOH buffer) to give compound **3i** (20.7 mg, 20%) as a colorless solid, as well as recovered starting material (33.3 mg, 39%).

Compound **3i** was also prepared according to General Procedure D, 0.1 mmol scale. Purification by preparative reverse phase HPLC (43-83% MeCN in HCOOH buffer) to give compound **3i** (74.8 mg, 72%) as a colorless solid.

^{1}H NMR (500 MHz, DMSO) δ 10.58 (s, 1H), 9.30 (d, *J* = 8.4 Hz, 1H), 8.19 (d, *J* = 8.4 Hz, 1H), 7.92 – 7.97 (m, 2H), 7.87 (dd, *J* = 7.9, 1.3 Hz, 1H), 7.69 – 7.74 (m, 1H), 7.63 (t, *J* = 7.6 Hz, 2H), 7.51 – 7.57 (m, 1H), 7.37 – 7.41 (m, 4H), 7.32 – 7.36 (m, 2H), 7.16 – 7.25 (m, 2H), 6.9 – 6.94 (m, 2H), 6.34 (d, *J* = 7.3 Hz, 1H), 6.28 (s, 1H), 5.86 (t, *J* = 8.7 Hz, 1H), 5.40 (d, *J* = 7.2 Hz, 1H), 5.30 (t, *J* = 8.6 Hz, 1H), 5 – 5.09 (m, 2H), 4.93 (d, *J* = 7.1 Hz, 1H), 4.90 (d, *J* = 10.9 Hz, 1H), 4.69 (s, 1H), 4.51 – 4.58 (m, 1H), 4.09 (dt, *J* = 11.0, 7.0 Hz, 1H), 3.96 – 4.05 (m, 2H), 3.73 (s, 3H), 3.59 (d, *J* = 7.2 Hz, 1H), 2.31 (tt, *J* = 9.0, 4.6 Hz, 1H), 2.16 (s, 3H), 2.11 (s, 3H), 1.75 – 1.83 (m, 4H), 1.63 (dd, *J* = 15.0, 9.3 Hz, 2H), 1.50 (s, 3H), 1.02 (s, 3H), 1.00 (s, 3H). **^{13}C NMR** (126 MHz, DMSO) δ 202.4, 172.7, 169.9, 168.8, 168.1, 165.2, 159.3, 152.8, 139.1, 138.9, 138.5, 133.5, 133.4, 132.4, 130.2, 130.0, 129.5, 128.7, 128.5, 128.4, 128.2, 127.7, 127.6, 121.9, 120.0, 118.7, 113.9, 83.6, 80.2, 76.7, 75.4, 74.8, 74.5, 73.5, 70.5, 69.5, 66.0, 57.4, 56.6, 55.1, 46.1, 43.0, 36.6, 34.6, 26.3, 22.6, 21.3, 20.7, 13.8, 9.8. **HRMS** (ESI) m/z calcd. For $C_{56}H_{60}N_2O_{17}$ $[M+H]^+$: 1033.3970, found 1033.3960.

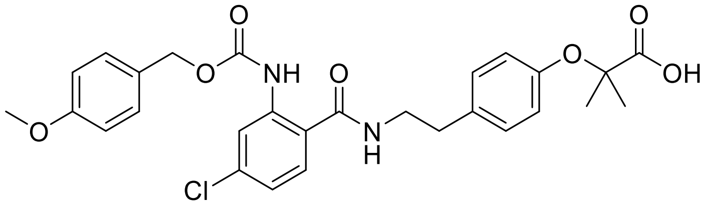

2-(4-(2-(4-chloro-2-((((4-methoxybenzyl)oxy)carbonyl)amino)benzamido)ethyl)phenoxy)-2-methylpropanoic acid (**3j**) was prepared according to General Procedure C, 0.2 mmol scale. Purification by preparative reverse phase HPLC (53-93% MeCN in HCOOH buffer) to give compound **3j** (59.9 mg, 55%) as a colorless solid.

^{1}H NMR (500 MHz, DMSO) δ 13.01 (s, 1H), 11.18 (s, 1H), 8.94 (t, *J* = 5.5 Hz, 1H), 8.28 (d, *J* = 2.1 Hz, 1H), 7.71 (d, *J* = 8.6 Hz, 1H), 7.35 – 7.39 (m, 2H), 7.18 (dd, *J* = 8.5, 2.2 Hz, 1H), 7.11 (d, *J* = 8.6 Hz, 2H), 6.92 – 6.97 (m, 2H), 6.72 – 6.77 (m, 2H), 5.09 (s, 2H), 3.75 (s, 3H), 3.38 – 3.44 (m, 2H), 2.75 (t, *J* = 7.5 Hz, 2H), 1.47 (s, 6H). **^{13}C NMR** (126 MHz, DMSO) δ 175.1, 167.4, 159.3, 153.7, 152.7, 140.7, 136.7, 132.3, 130.3, 129.8, 129.4, 128.0, 121.6, 118.5, 117.9, 117.9, 113.9, 78.3, 66.3, 55.1, 41.0, 33.8, 25.1. **HRMS** (ESI) m/z calcd. For $C_{28}H_{29}ClN_2O_7$ $[M+H]^+$: 541.1741, found 541.1760.

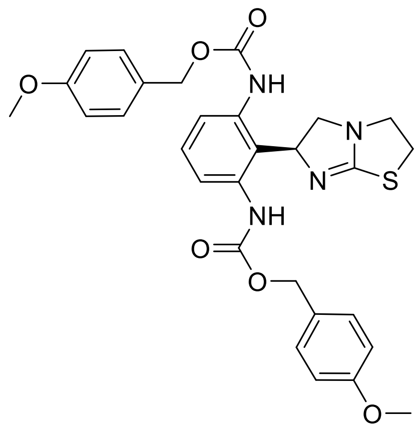

Bis(4-methoxybenzyl) (2-(2,3,5,6-tetrahydroimidazo[2,1-b]thiazol-6-yl)-1,3-phenylene)(S)-dicarbamate (**3k**) was prepared according to General Procedure C. Purification by preparative reverse phase HPLC (44-84% MeCN in NH_3 buffer) to give compound **3k** (18.1 mg, 32%) as a yellow solid.

^{1}H NMR (500 MHz, DMSO) δ 8.37 (s, 1H), 7.26 – 7.36 (m, 6H), 7.07 – 7.11 (m, 2H), 6.88 – 6.92 (m, 2H), 6.81 – 6.85 (m, 2H), 5.16 (dd, *J* = 9.4, 3.4 Hz, 1H), 4.95 – 5.01 (m, 4H), 3.85 – 3.93 (m, 1H), 3.73 (s, 6H), 3.39 – 3.47 (m, 1H), 3.3 – 3.38 (m, 3H), 3.23 (dd, *J* = 9.3, 3.3 Hz, 1H), 2.76 (t, *J* = 7.1 Hz, 2H). **^{13}C NMR** (151 MHz, DMSO) δ 159.2, 159.0, 157.6, 153.0, 151.0, 141.9, 129.9, 129.3, 128.7, 128.3, 127.7, 127.7, 125.7, 113.8, 113.6, 66.7, 66.5, 55.1, 54.9, 49.9, 41.5, 36.3.

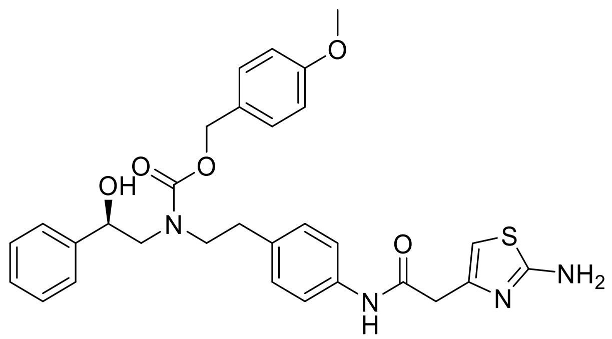

4-methoxybenzyl (R)-(4-(2-(2-aminothiazol-4-yl)acetamido)phenethyl)(2-hydroxy-2-phenylethyl)carbamate (**3l**) was prepared according to General Procedure E. Solvent NMP. Purification by preparative LC (pH 10, basic method). Compound **3l** (6.8 mg, 61%, >95% purity) was obtained as a colorless solid.

^{1}H NMR (500 MHz, DMSO) δ 10.01 (d, *J* = 3.5 Hz, 1H), 7.50 (d, *J* = 8.4 Hz, 1H), 7.47 (d, *J* = 8.4 Hz, 1H), 7.26 – 7.32 (m, 5H), 7.18 – 7.25 (m, 2H), 7.07 (d, *J* = 8.3 Hz, 1H), 6.98 (d, *J* = 8.4 Hz, 1H), 6.93 – 6.96 (m, 2H), 6.90 (s, 2H), 6.29 (s, 1H), 5.46 (dd, *J* = 8.9, 4.6 Hz, 1H), 4.93 – 4.98 (m, 2H), 4.70 (ddt, *J* = 36.2, 8.5, 4.5 Hz, 1H), 3.76 (s, 3H), 3.14 – 3.31 (m, 3H), 2.58 – 2.73 (m, 2H). **^{13}C NMR** (151 MHz, DMSO) δ 168.3, 167.9, 159.0, 155.6, 155.2, 145.9, 143.7, 143.6, 137.4, 133.9, 133.8, 129.6, 128.9, 128.08, 128.05, 127.1, 126.0, 125.9, 119.0, 113.8, 102.6, 71.6, 71.0, 66.01, 65.97, 55.4, 55.1, 54.9, 50.1, 49.8, 33.7, 32.9. **HRMS** (ESI) m/z calcd. For $C_{30}H_{32}N_4O_5S$ $[M+H]^+$: 561.2172, found 561.2181.

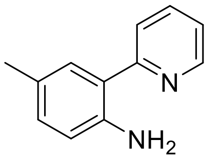

4-methyl-2-(pyridin-2-yl)aniline (**4a**) was prepared with 3 distinct protocols.

Conditions A: Acidic deprotection
4-methoxybenzyl (4-methyl-2-(pyridin-2-yl)phenyl)carbamate (**2c**, 70.0 mg, 0.2 mmol) was added into a 4 mL screw top vial, followed by addition of CH_2Cl_2 (1 mL) and TFA (0.5 mL). The resulting mixture was stirred at room temperature for 10 minutes, followed by removal of volatiles *in vacuo*. The residue was redissolved in CH_2Cl_2 (2 mL) and washed with saturated aq. $NaHCO_3$ (2mL). The aqueous phase was extracted with CH_2Cl_2 (2 x 2 mL). The combined organic phases were concentrated *in vacuo*. Purification by automated flash column chromatography (0-60% EtOAc in heptane, 25g SiO_2). Compound **4a** was obtained as a yellow solid (35.1 mg, 93%).

Conditions B: Basic deprotection
4-methoxybenzyl (4-methyl-2-(pyridin-2-yl)phenyl)carbamate (**2c**, 70.0 mg, 0.2 mmol) was added into a 4 mL screw top vial, followed by addition of powdered KOH (157.0 mg, 2.8 mmol) EtOH (95%, 1 mL). The resulting mixture was heated at 80 °C for 1 hour, followed by removal of volatiles *in vacuo*. The residue was redissolved in CH_2Cl_2 (4 mL) and washed with saturated aq. NH_4Cl (8 mL). The aqueous phase was extracted with CH_2Cl_2 (2 x 4 mL).Purification by automated flash column chromatography (0-60% EtOAc in heptane, 25g SiO_2). Compound **4a** was obtained as a yellow solid (27.9 mg, 74%).

Conditions C: Hydrogenolysis
4-methoxybenzyl (4-methyl-2-(pyridin-2-yl)phenyl)carbamate (**2c**, 70.0 mg, 0.2 mmol) was added into a 4 mL screw top vial, followed by addition of Pd/C (22.7 mg, 10 mol%, Pd 10% wt.), EtOAc (0.5 mL), MeOH (0.5 mL) and AcOH (50 µL). The resulting mixture was stirred under an H_2 atmosphere (1.2 bar) at room temperature for 5 hours. Solids were removed by filtration, and the filtrate concentrated *in vacuo*. The residue was redissolved in CH_2Cl_2 (2 mL) and washed with saturated aq. $NaHCO_3$ (2mL). The aqueous phase was extracted with CH_2Cl_2 (2 x 2 mL). The combined organic phases were concentrated *in vacuo*. Purification by automated flash column chromatography (0-60% EtOAc in heptane, 25g SiO_2). Compound **4a** was obtained as a yellow solid (30.8 mg, 82%).

^{1}H NMR (500 MHz, DMSO) δ 8.55 – 8.63 (m, 1H), 7.85 (td, *J* = 8.0, 1.9 Hz, 1H), 7.76 (d, *J* = 8.2 Hz, 1H), 7.34 (s, 1H), 7.26 (ddd, *J* = 7.3, 4.9, 0.9 Hz, 1H), 6.92 (dd, *J* = 8.1, 1.6 Hz, 1H), 6.68 (d, *J* = 8.2 Hz, 1H), 6.32 (s, 2H), 2.21 (s, 3H). **^{13}C NMR** (126 MHz, DMSO) δ 158.98, 147.74, 145.25, 137.13, 130.43, 129.24, 124.17, 121.75, 120.89, 120.31, 116.73, 20.18. **HRMS** (ESI) m/z calcd. For $C_{12}H_{12}N_2$ $[M+H]^+$: 185.1078, found 185.1071.

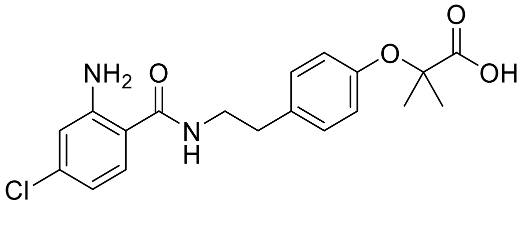

2-(4-(2-(2-amino-4-chlorobenzamido)ethyl)phenoxy)-2-methylpropanoic acid (**4b**) was prepared from commercially available *Bezofibrate* (72.4 mg, 0.2 mmol) utilizing a sequential one pot protocol. The

substrate was weighed in a 4 mL screw top glass vial, followed by addition of $MozN_3$ (62.2 mg, 0.3 mmol), $[Cp^*Ir(H_2O)_3]SO_4$ (9.7 mg, 0.02 mmol, 10 mol%) and DCE (1.0 mL). The sealed vial was placed in a heating block at 60 °C and stirred for 20 hours. After this, the reaction mixture was let to cool to room temperature, followed by addition of TFA (0.5 mL). The Mixture was let to stir at room temperature for 10 minutes, followed by removal of volatiles *in vacuo*. Purification by preparative reverse phase HPLC (36-76% MeCN in HCOOH buffer) to give compound **4b** (44.9 mg, 60%) as a light brown solid.

^{1}H NMR (500 MHz, DMSO) δ 8.36 (t, *J* = 5.5 Hz, 1H), 7.42 (d, *J* = 8.5 Hz, 1H), 7.09 – 7.12 (m, 2H), 6.73 – 6.76 (m, 3H), 6.66 (s, 2H), 6.51 (dd, *J* = 8.5, 2.2 Hz, 1H), 3.36 – 3.4 (m, 2H), 2.7 – 2.76 (m, 2H), 1.47 (s, 6H). **^{13}C NMR** (126 MHz, DMSO) δ 175.2, 168.0, 153.7, 151.0, 136.0, 132.7, 129.9, 129.4, 118.5, 115.0, 114.2, 113.5, 78.3, 40.7, 34.2, 25.1. **HRMS** (ESI) m/z calcd. For $C_{19}H_{21}ClN_2O_4$ $[M+H]^+$: 377.1268, found 377.1264.

NMR Spectra

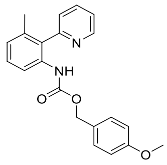

4-methoxybenzyl (3-methyl-2-(pyridin-2-yl)phenyl)carbamate (**2a**)

Frequency: 500.13MHz
Experiment Name: Proton_day
#Scans: 16
Solvent: CDCl3

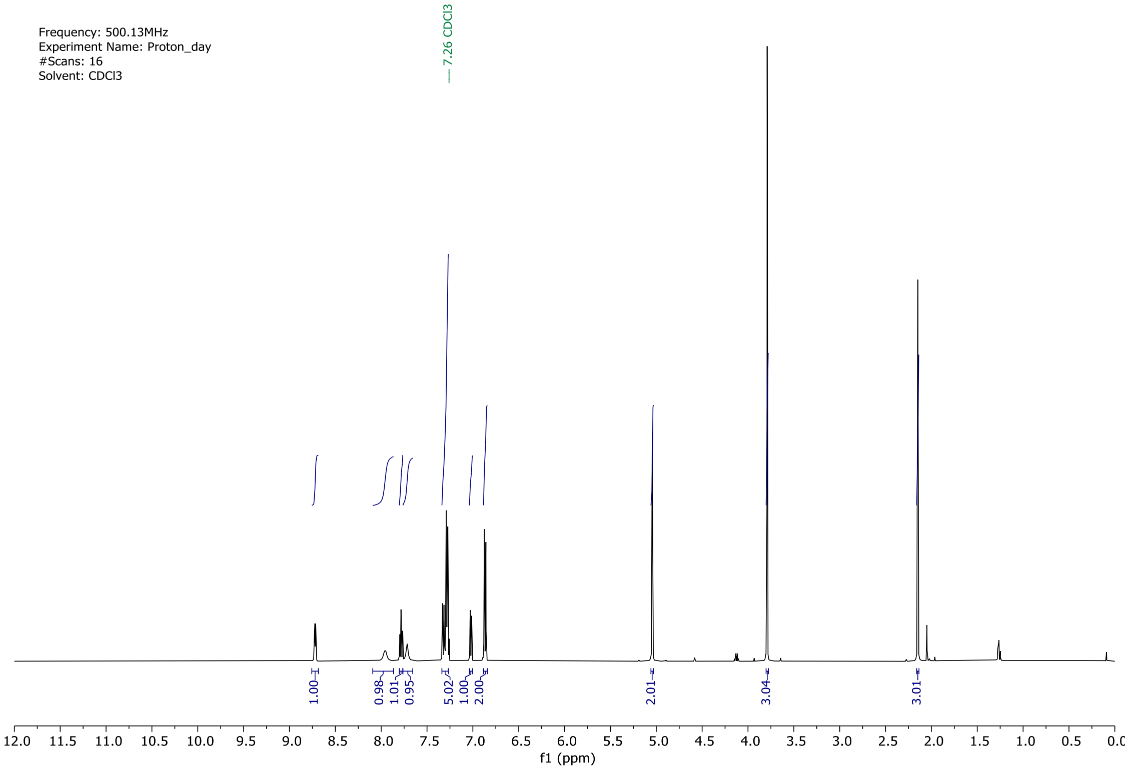

Frequency: 125.76MHz
Experiment Name: Carbon_day
#Scans: 296
Solvent: CDCl3

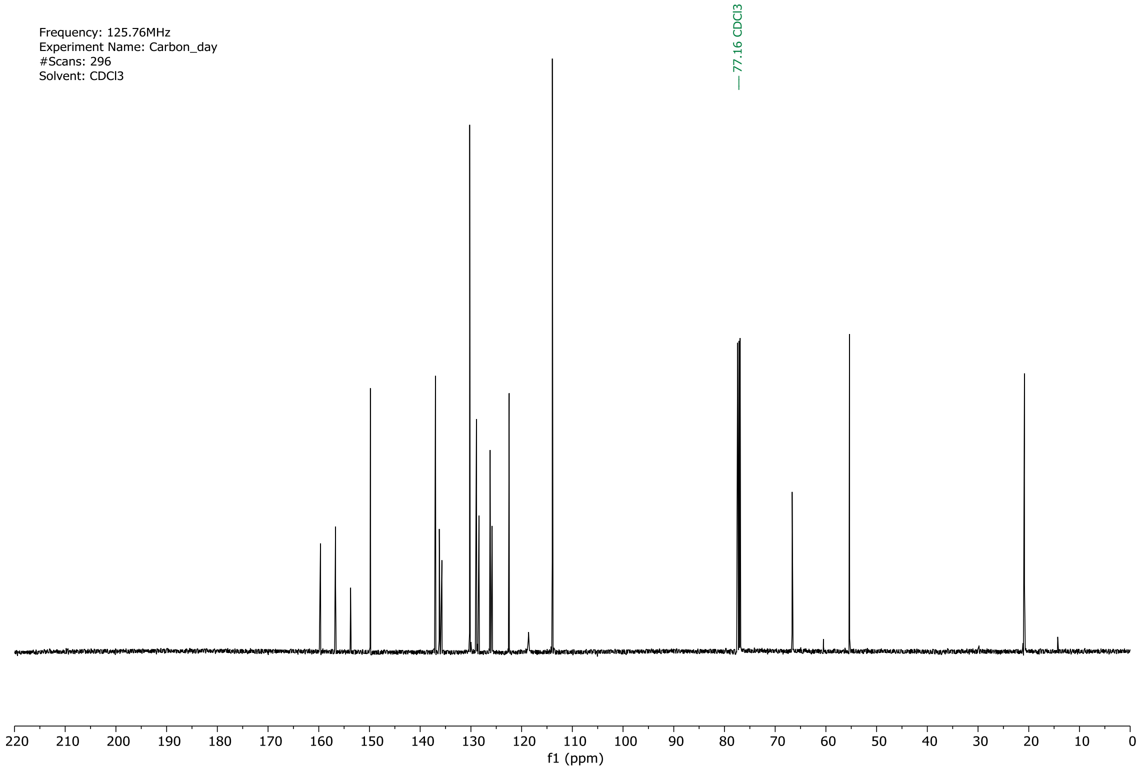

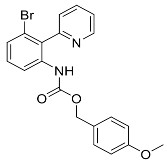

4-methoxybenzyl (3-bromo-2-(pyridin-2-yl)phenyl)carbamate (**2b**)

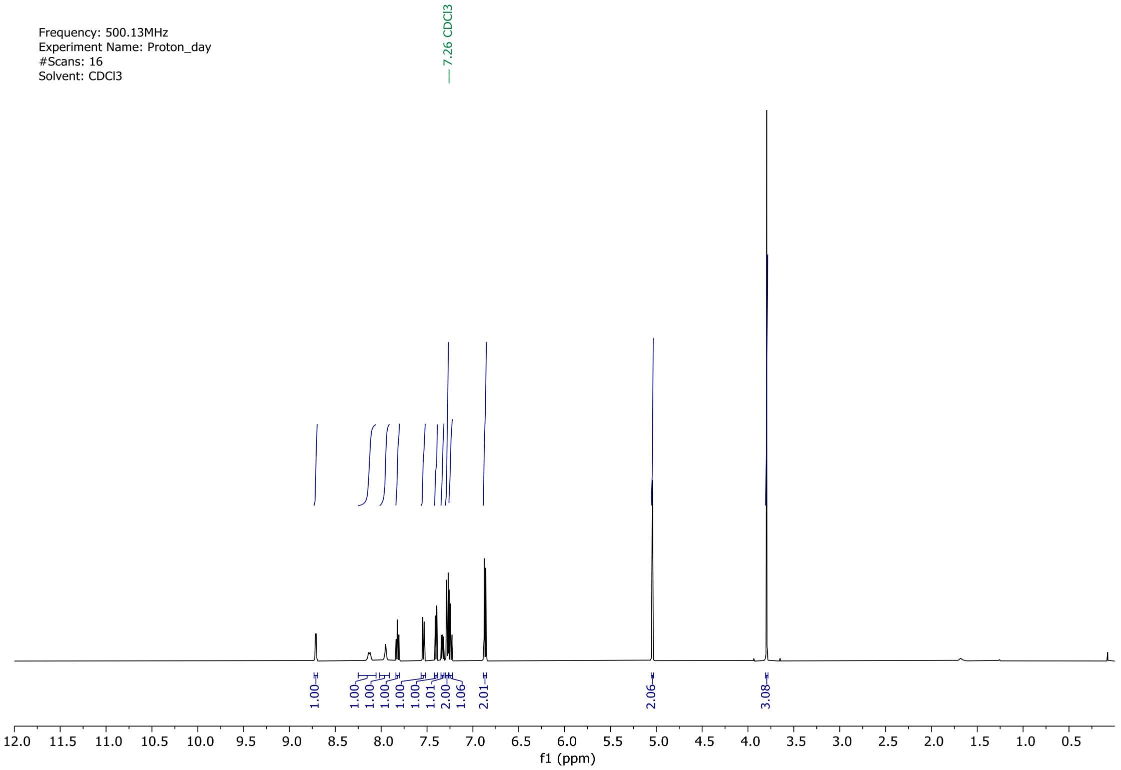

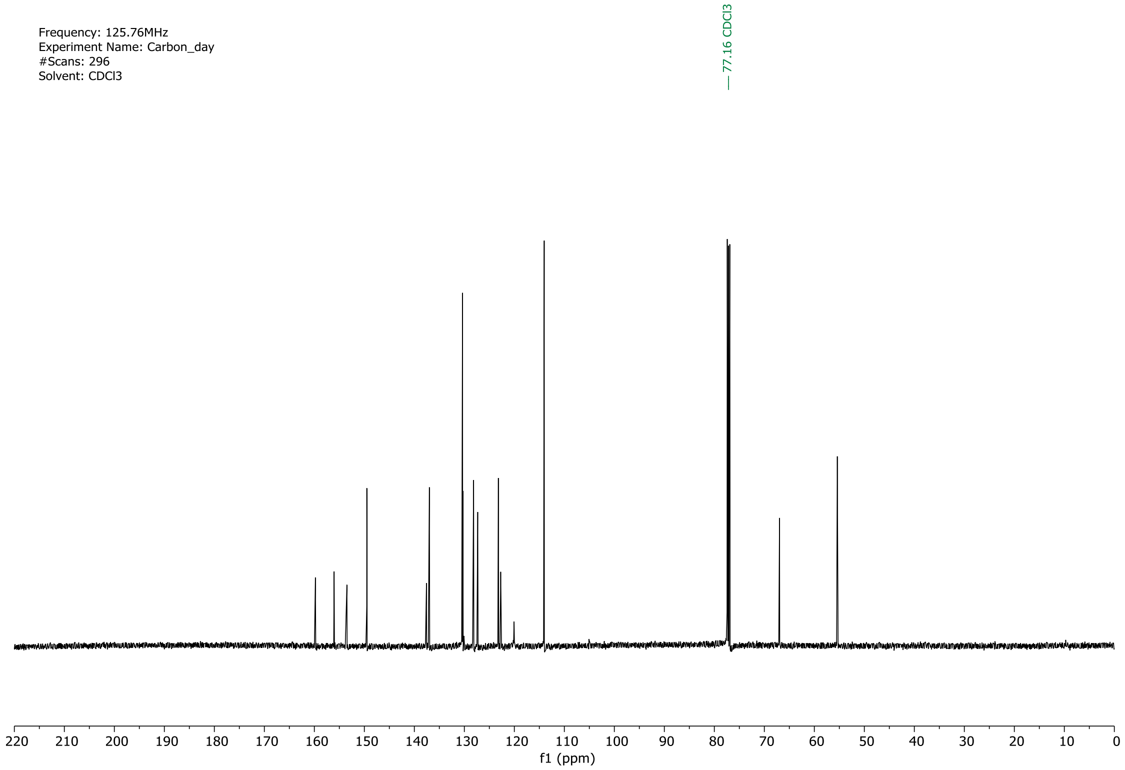

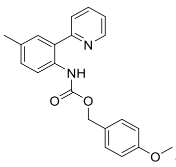

4-methoxybenzyl (3-methyl-2-(pyridin-2-yl)phenyl)carbamate (**2c**)

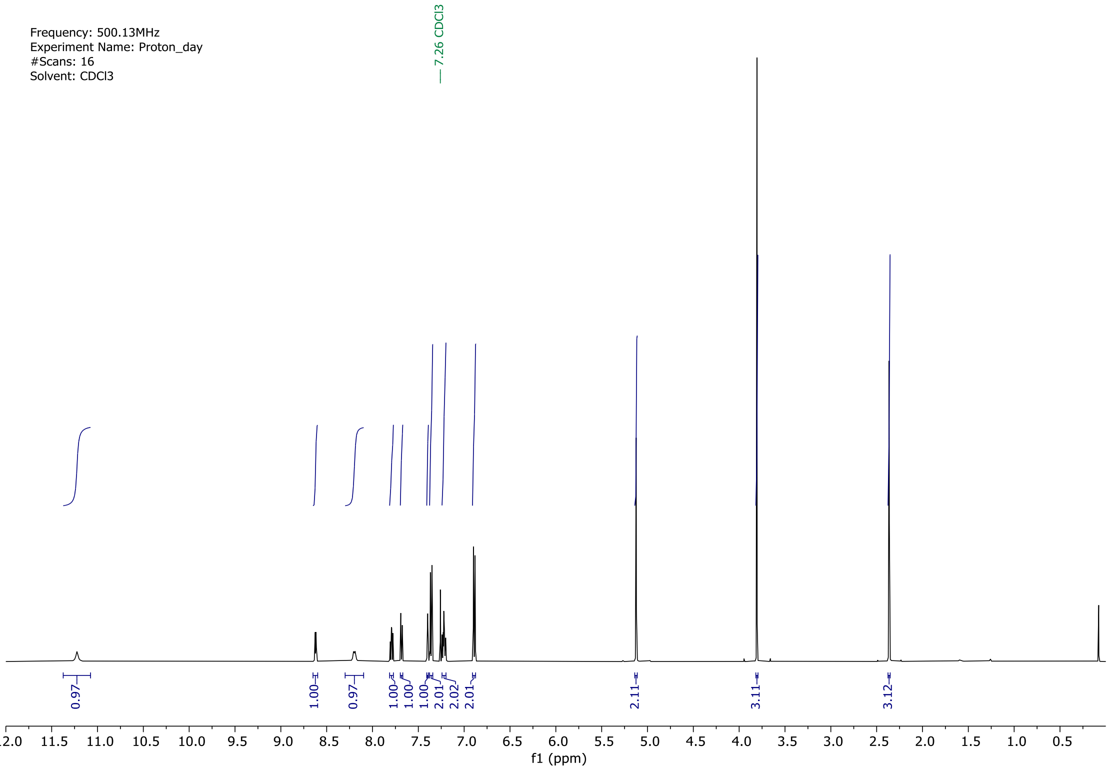

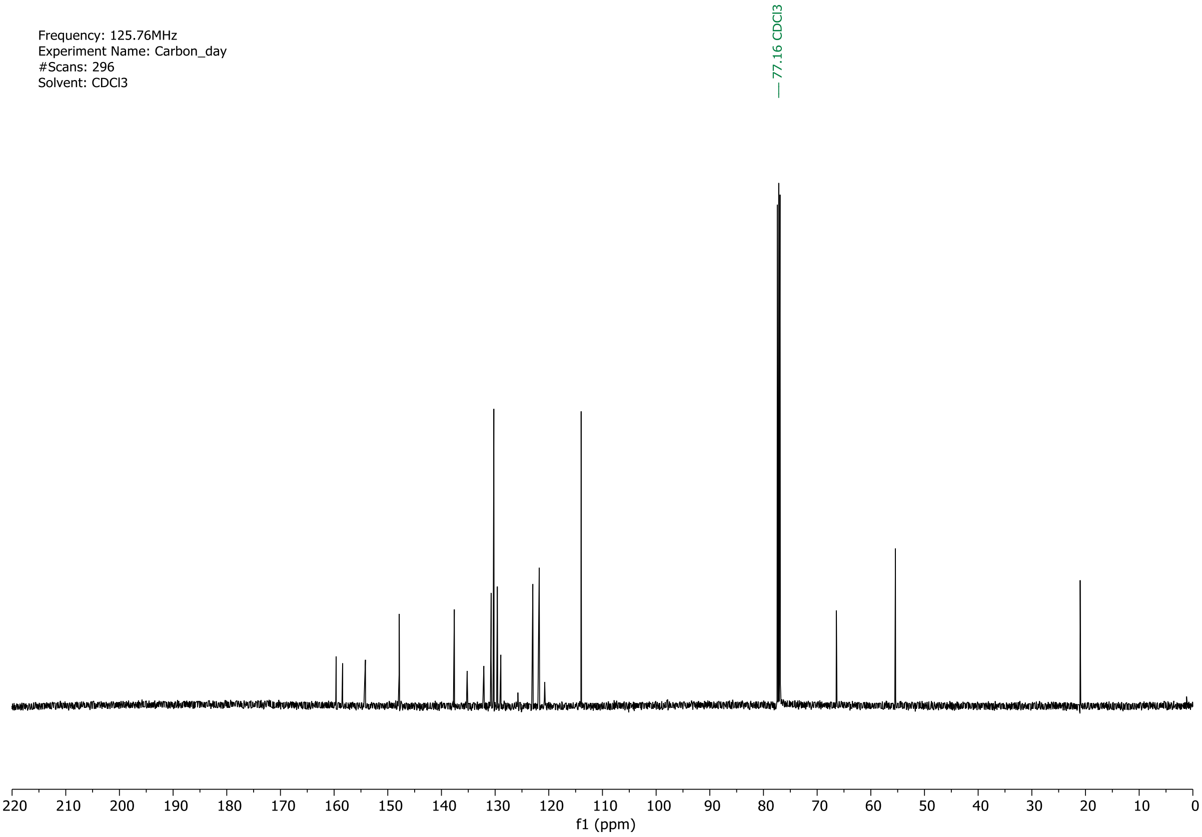

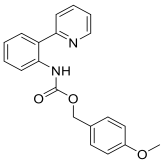

4-methoxybenzyl (2-(pyridin-2-yl)phenyl)carbamate (**2d**)

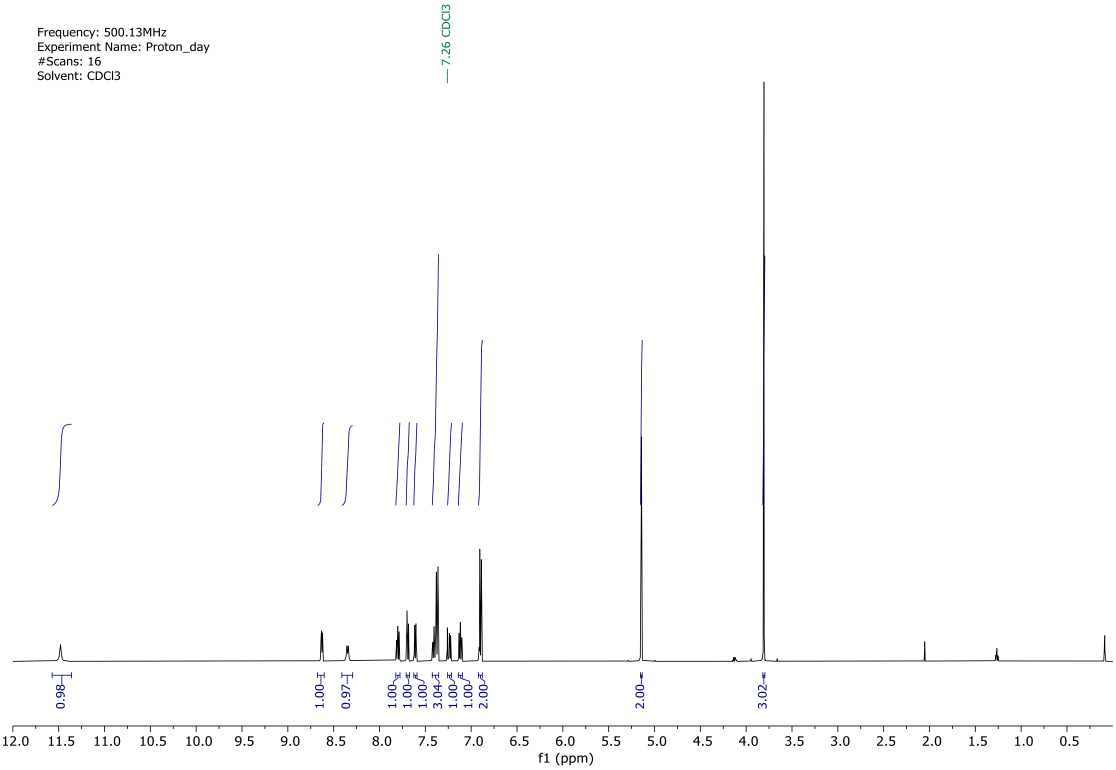

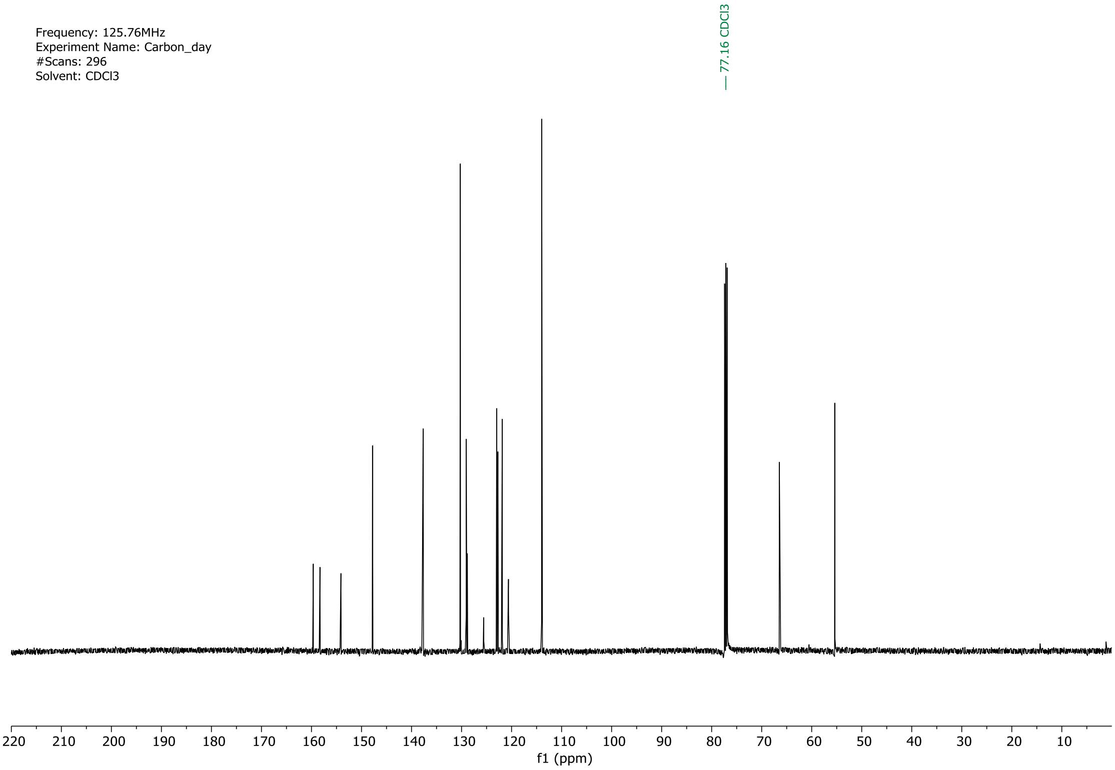

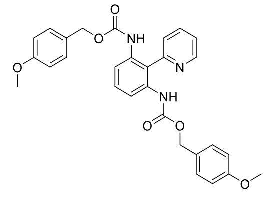

bis(4-methoxybenzyl) (2-(pyridin-2-yl)-1,3-phenylene)dicarbamate (**2d´**)

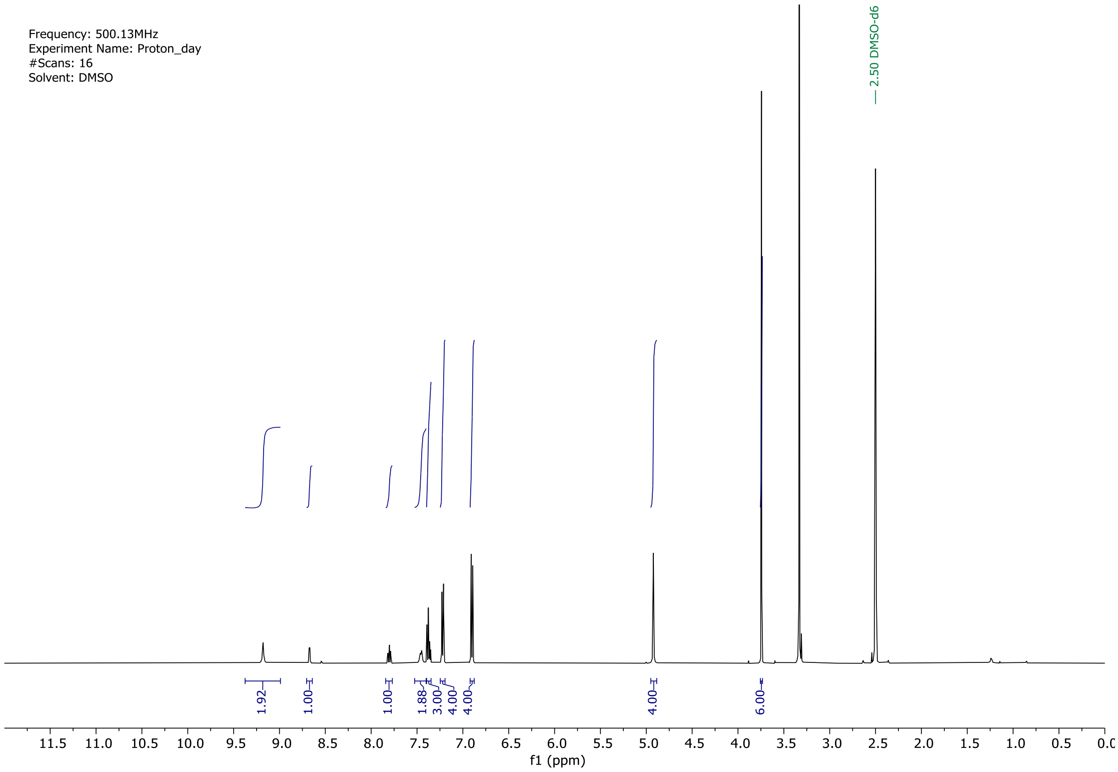

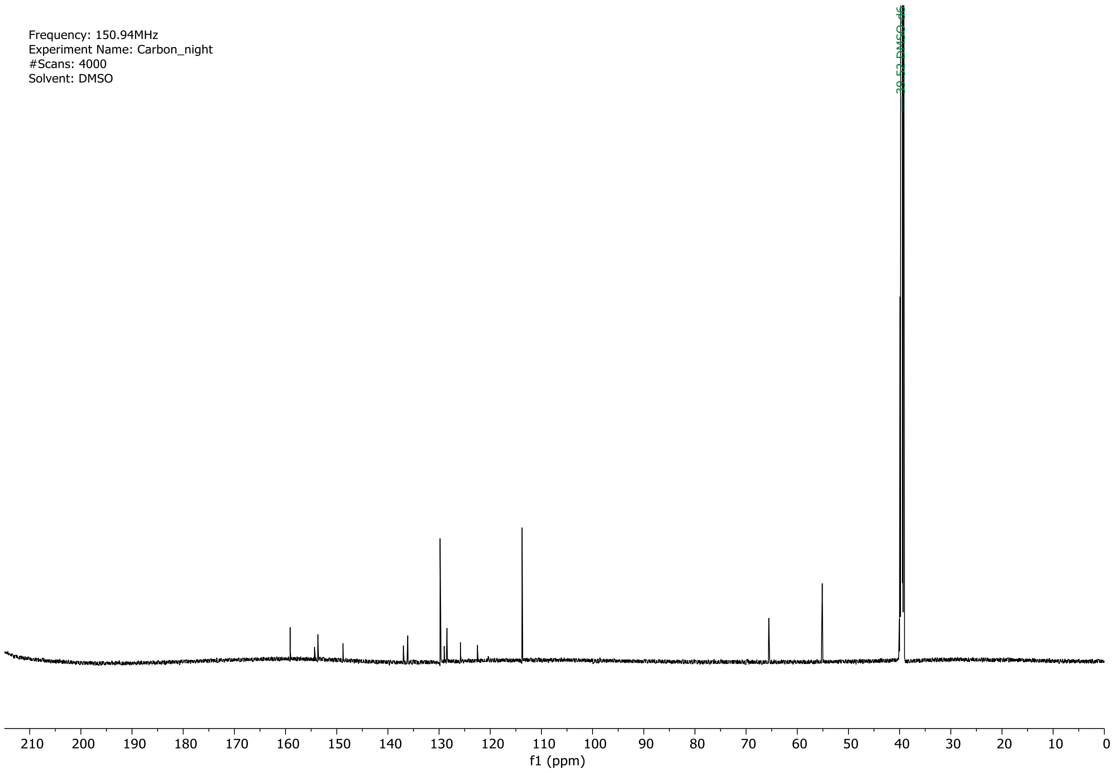

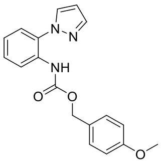

4-methoxybenzyl (2-(1H-pyrazol-1-yl)phenyl)carbamate (**2e**)

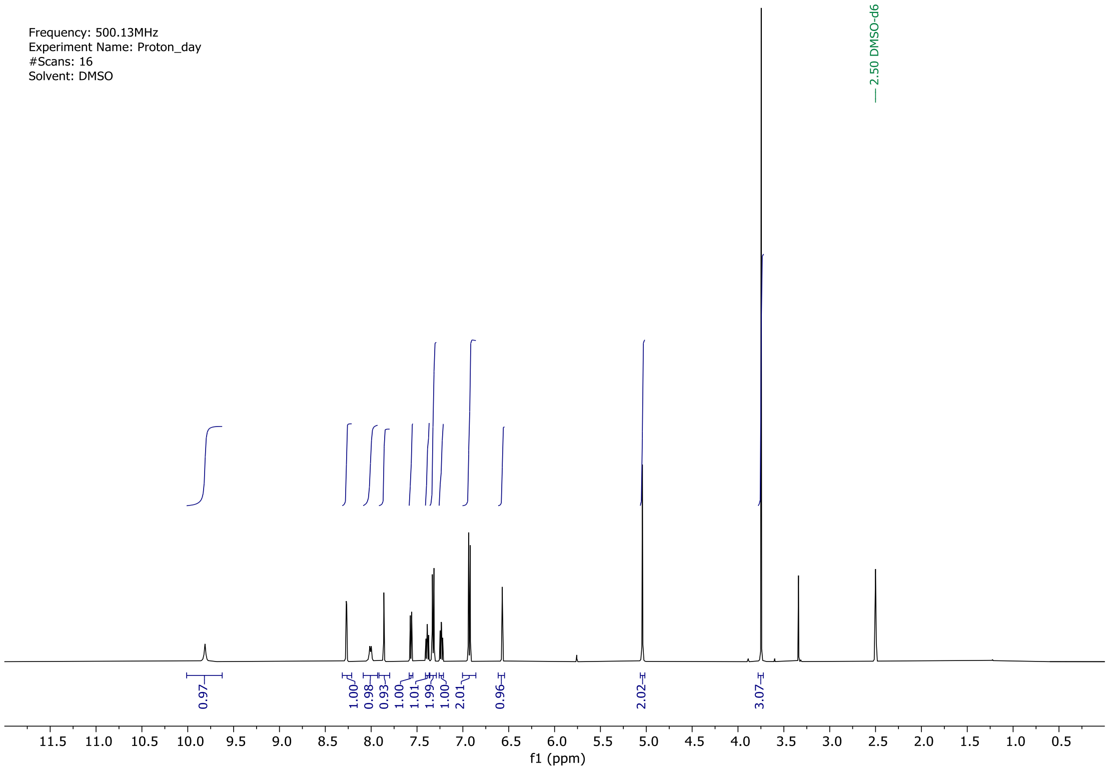

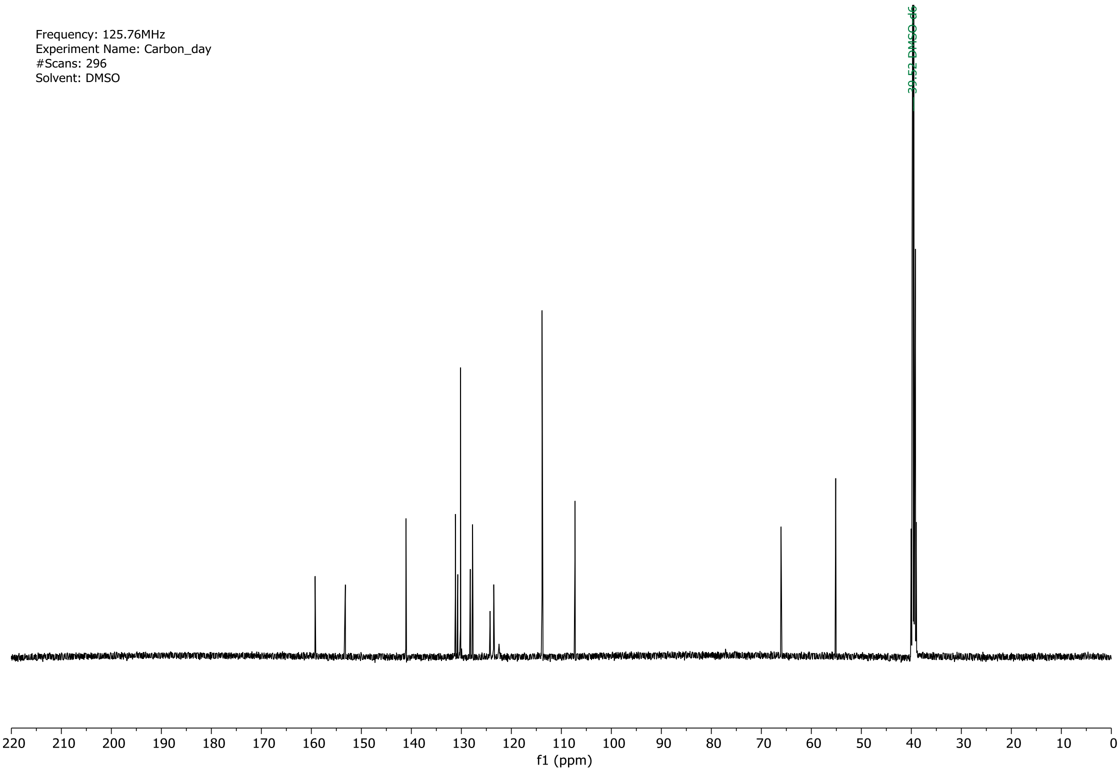

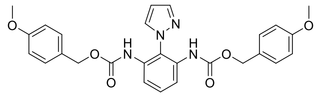

bis(4-methoxybenzyl) (2-(1H-pyrazol-1-yl)-1,3-phenylene)dicarbamate (**2e´**)

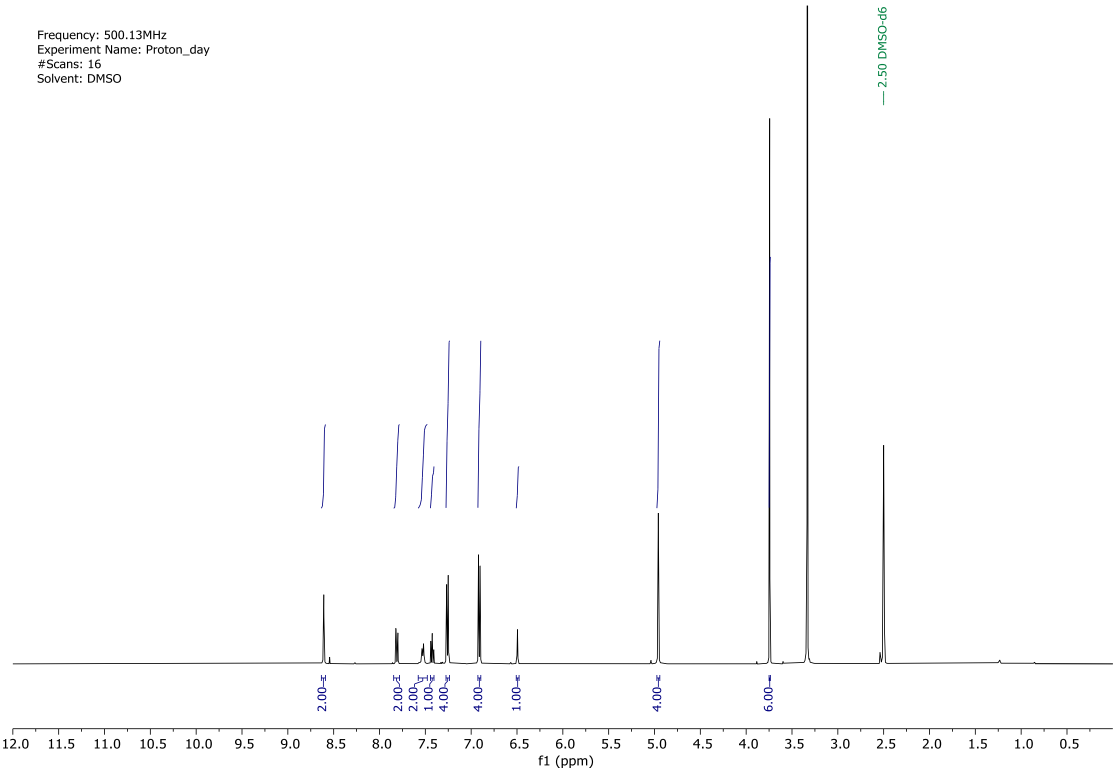

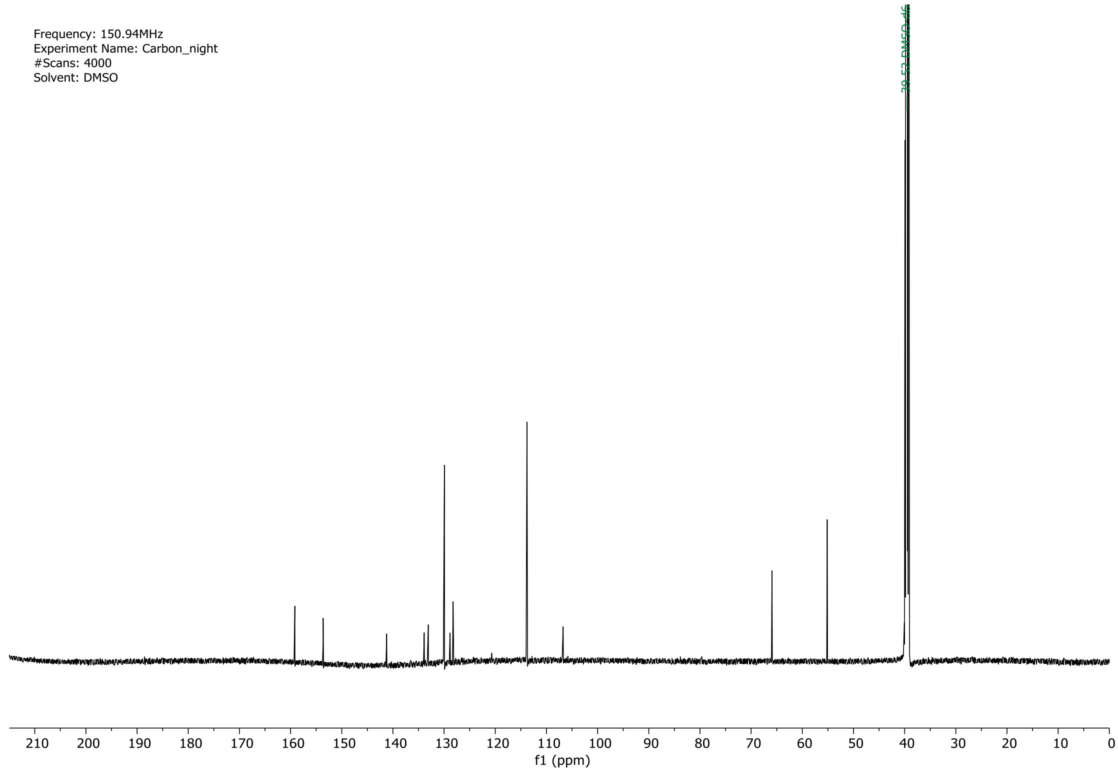

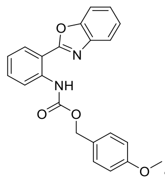

4-methoxybenzyl (2-(benzo[d]oxazol-2-yl)phenyl)carbamate (**2f**)

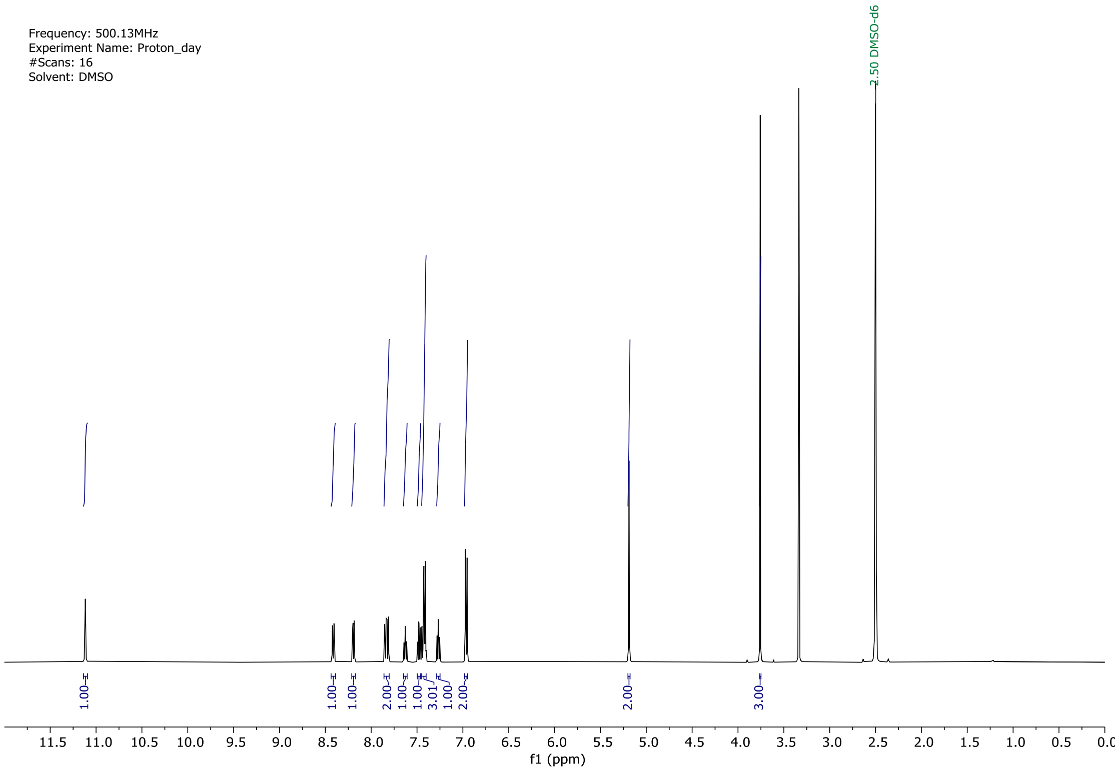

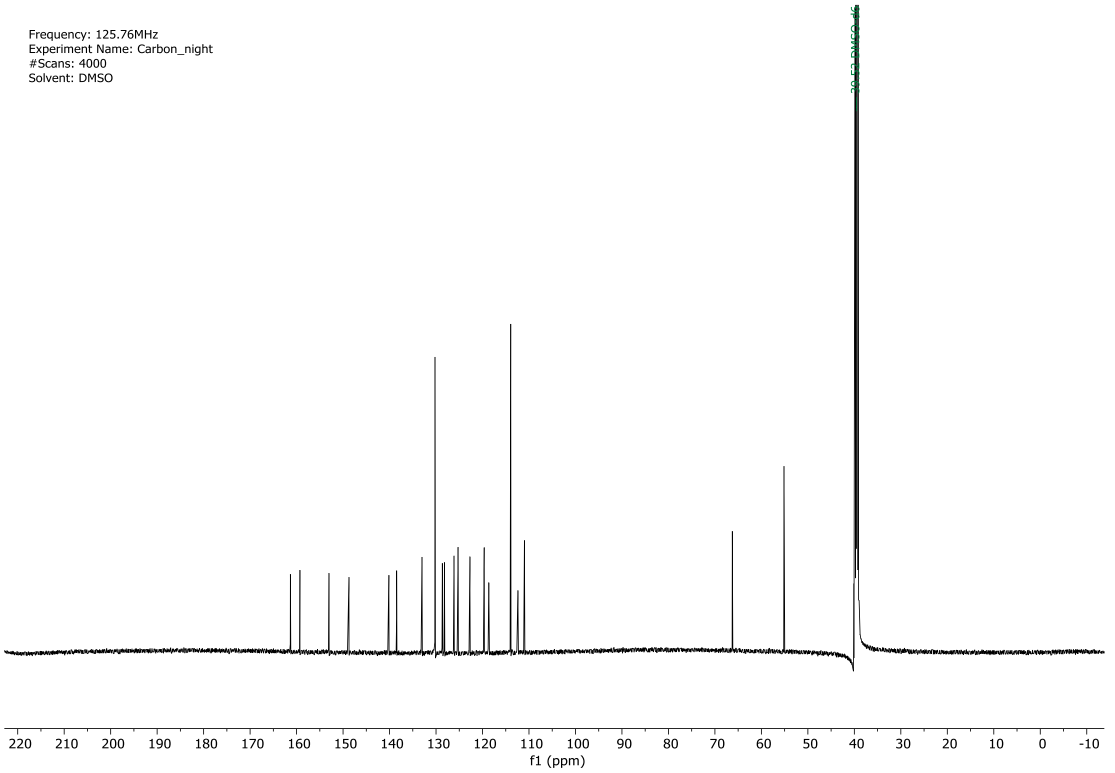

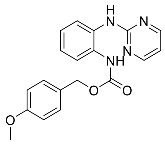

4-methoxybenzyl (2-(pyrimidin-2-ylamino)phenyl)carbamate (**2g**)

Frequency: 500.13MHz
Experiment Name: Proton_day
#Scans: 16
Solvent: DMSO

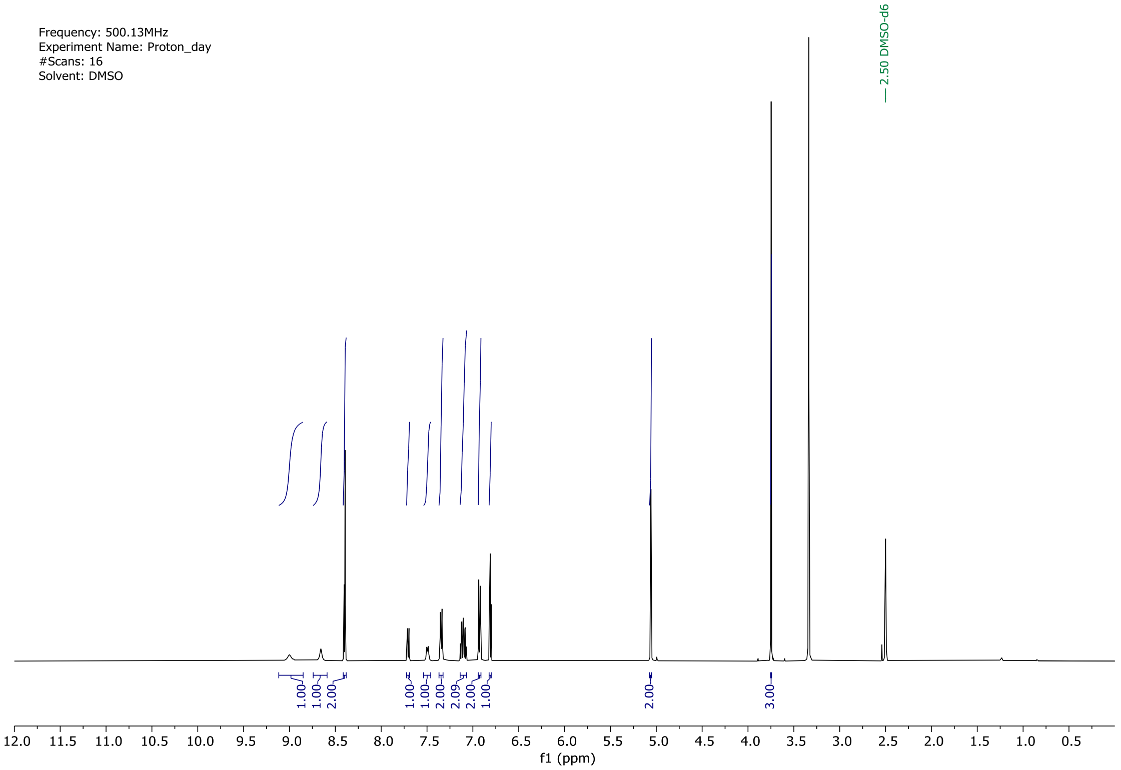

Frequency: 150.94MHz
Experiment Name: Carbon_night
#Scans: 4000
Solvent: DMSO

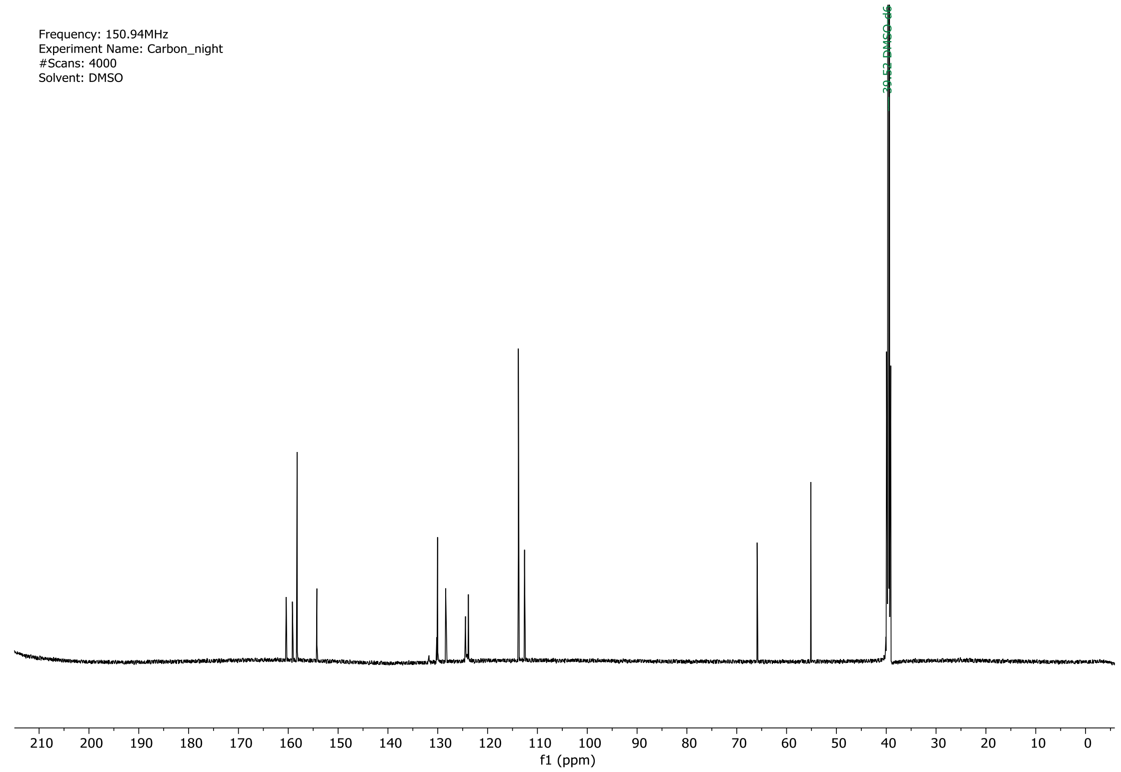

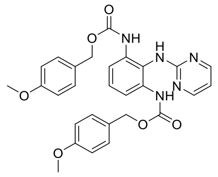

bis(4-methoxybenzyl) (2-(pyrimidin-2-ylamino)-1,3-phenylene)dicarbamate (**2g´**)

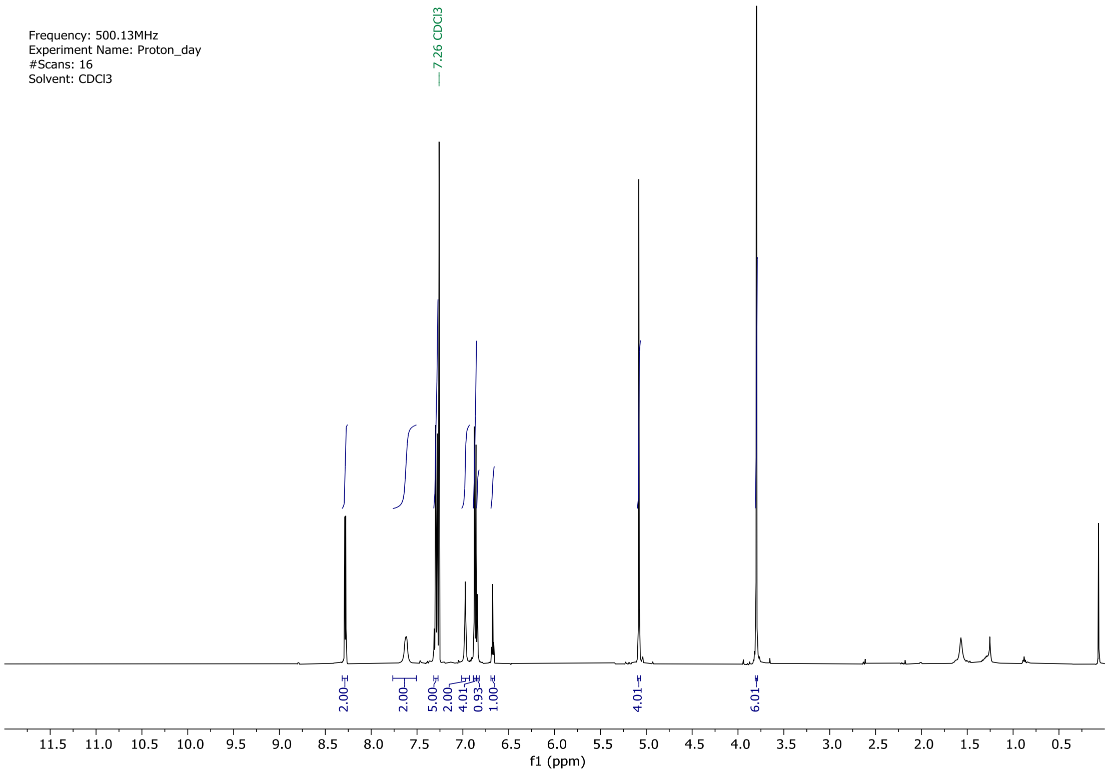

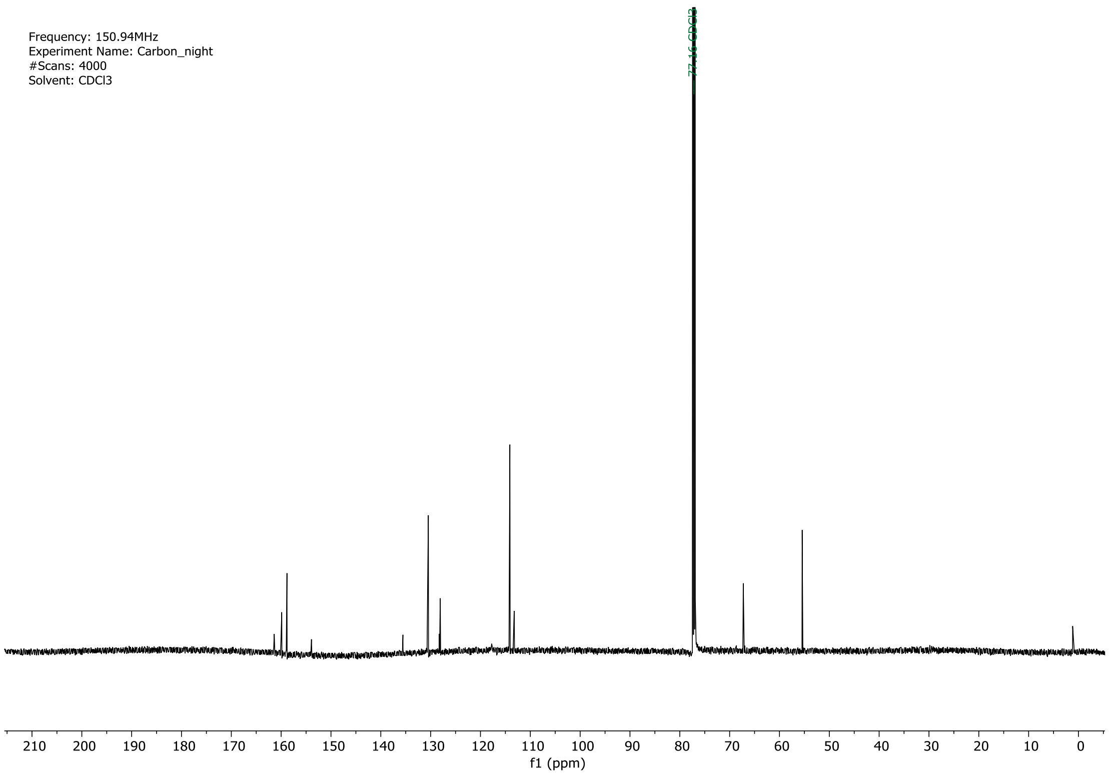

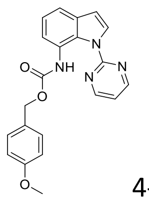

4-methoxybenzyl (1-(pyrimidin-2-yl)-1H-indol-7-yl)carbamate (**2h**)

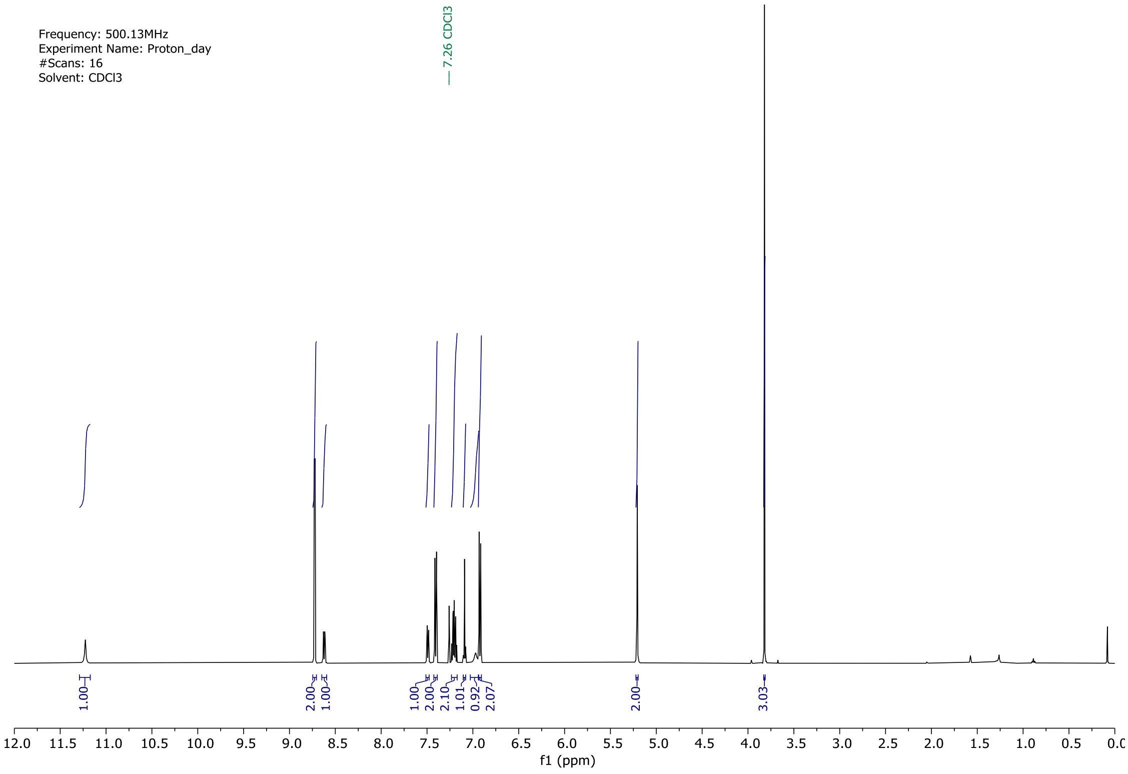

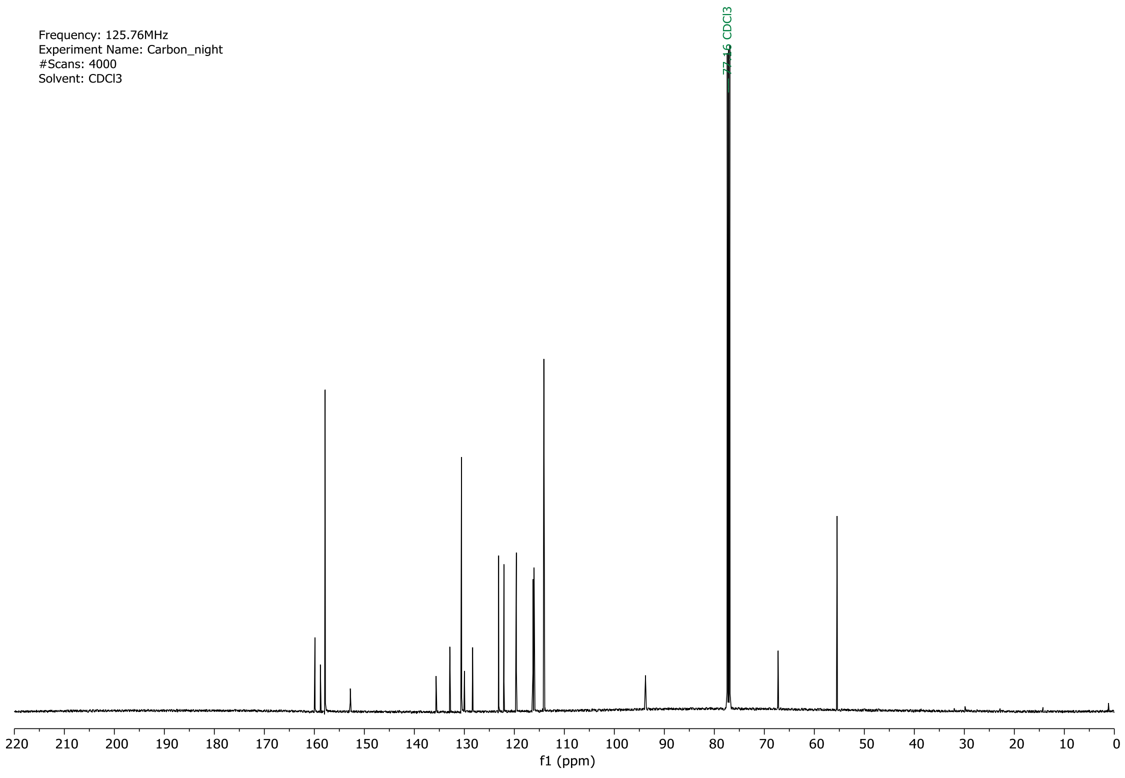

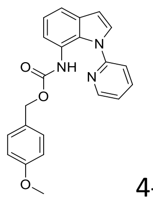

4-methoxybenzyl (1-(pyridin-2-yl)-1H-indol-7-yl)carbamate (**2i**)

Frequency: 500.13MHz
Experiment Name: Proton_day
#Scans: 16
Solvent: CDCl3

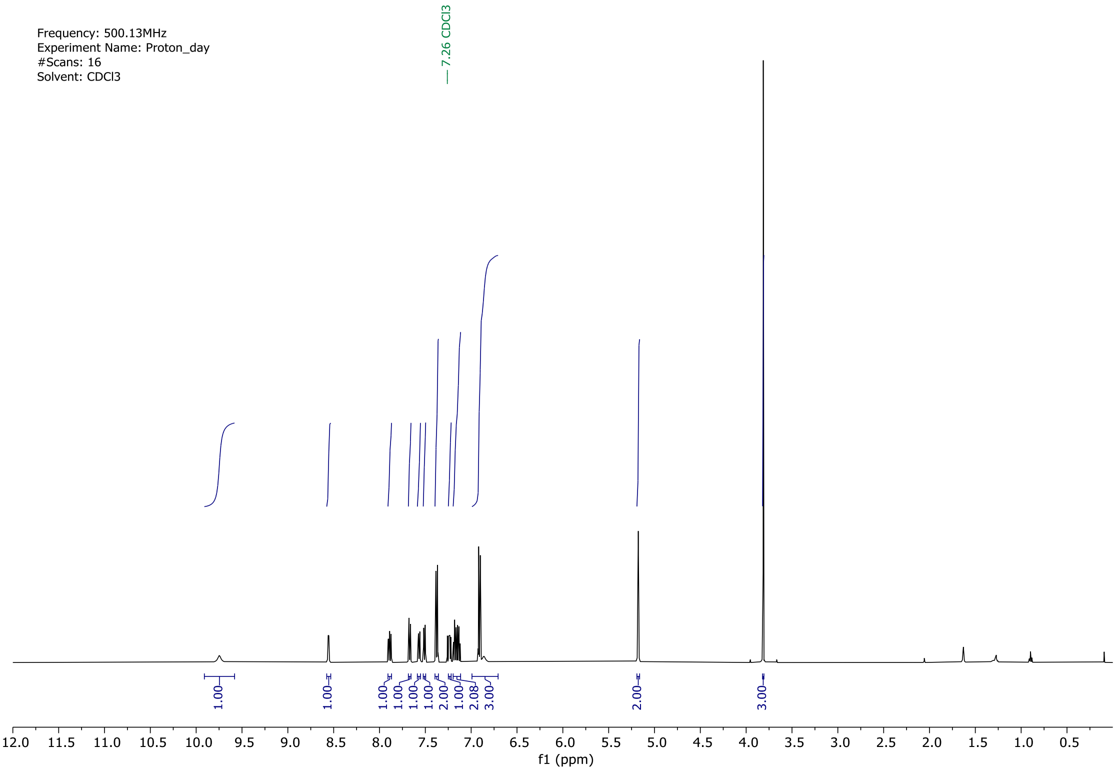

Frequency: 125.76MHz
Experiment Name: Carbon_night
#Scans: 4000
Solvent: CDCl3

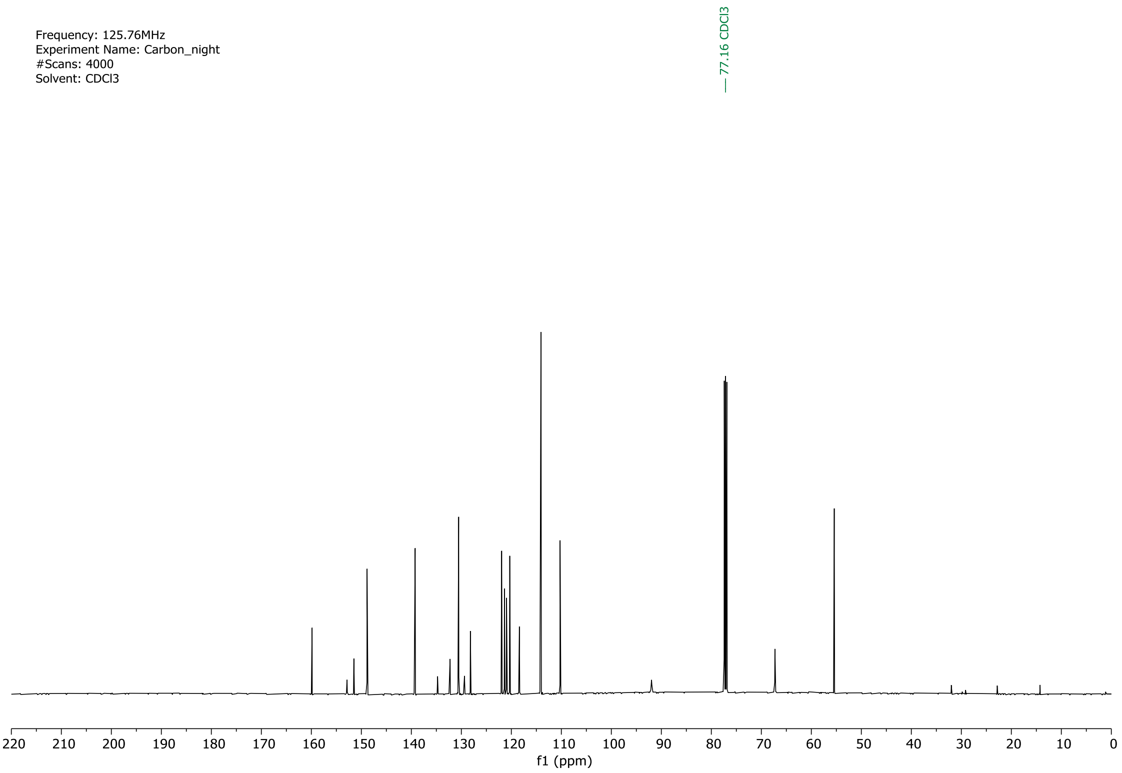

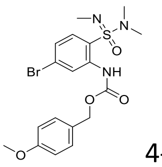

4-methoxybenzyl (5-bromo-2-(N,N,N'-trimethylsulfamidimidoyl)phenyl)carbamate (**2j**)

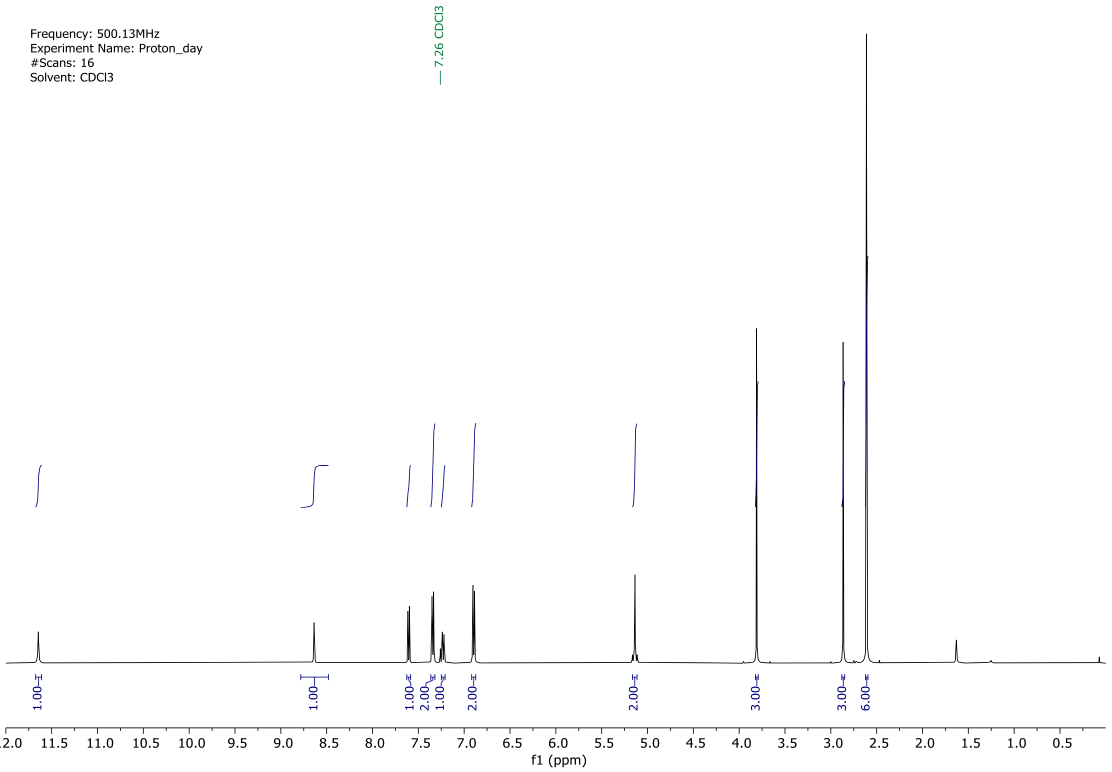

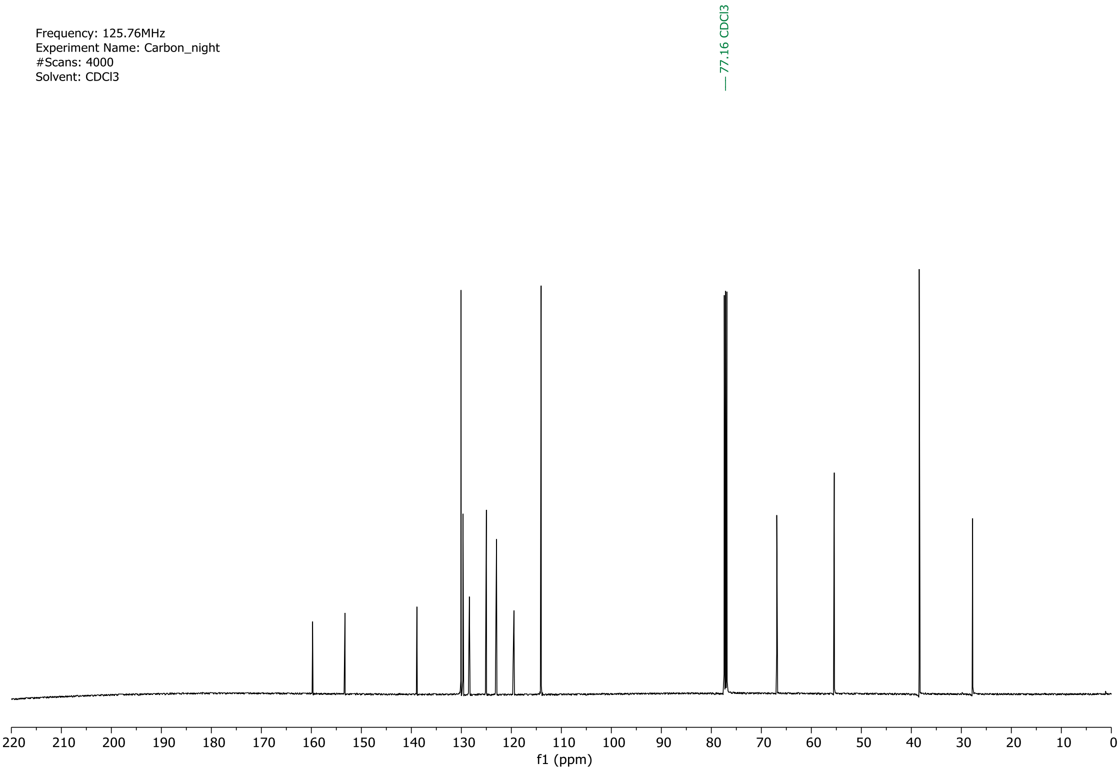

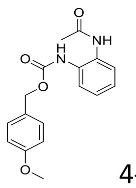

4-methoxybenzyl (2-acetamidophenyl)carbamate (**2k**)

Frequency: 500.13MHz
Experiment Name: Proton_day
#Scans: 16
Solvent: CDCl3

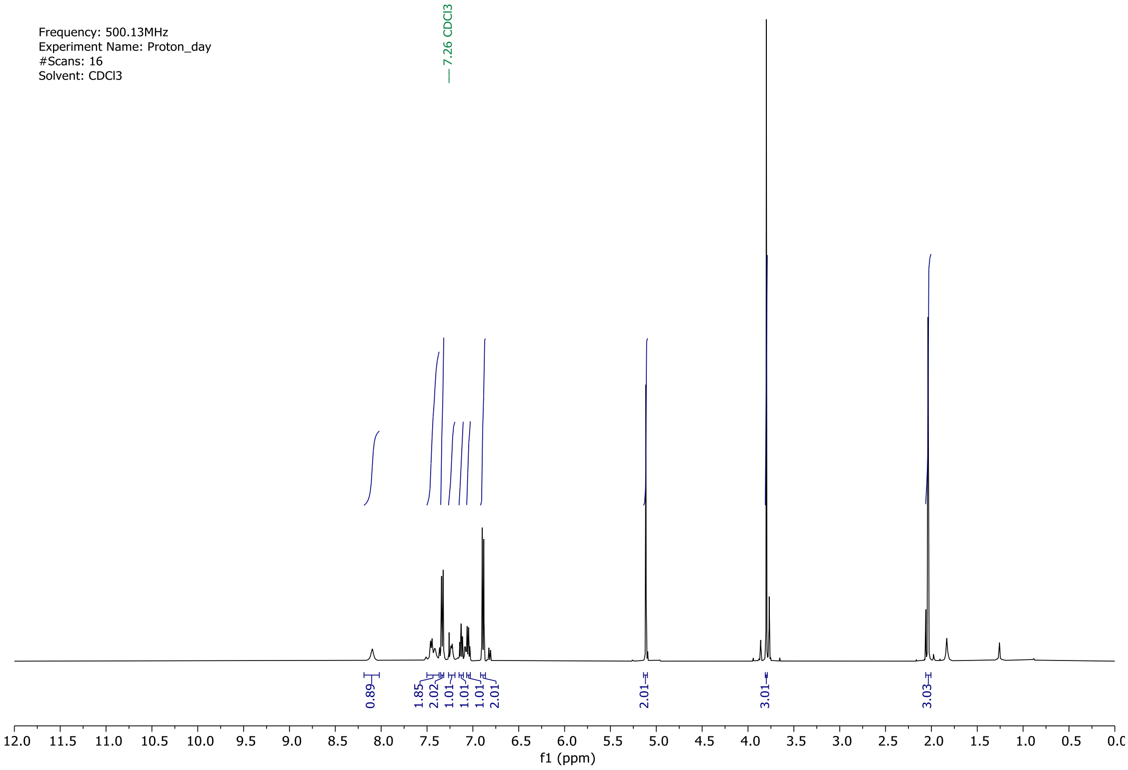

Frequency: 125.76MHz
Experiment Name: Carbon_night
#Scans: 4000
Solvent: CDCl3

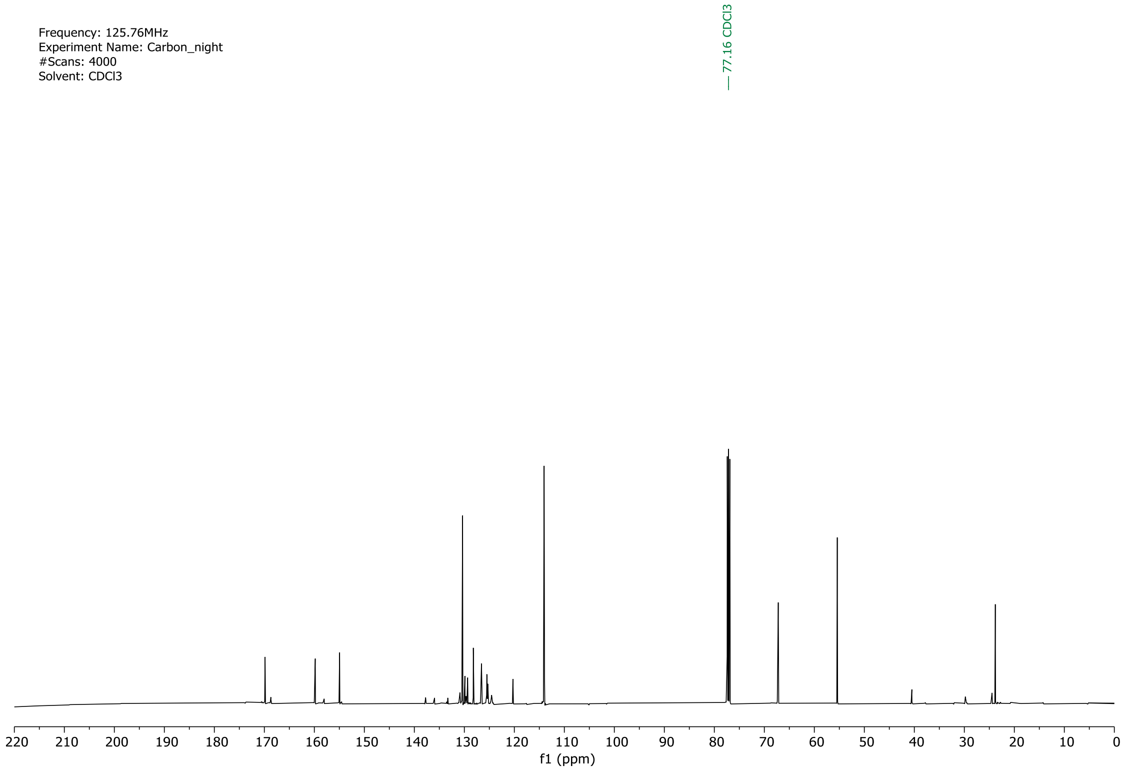

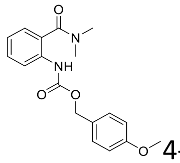

4-methoxybenzyl (2-(dimethylcarbamoyl)phenyl)carbamate (**2l**)

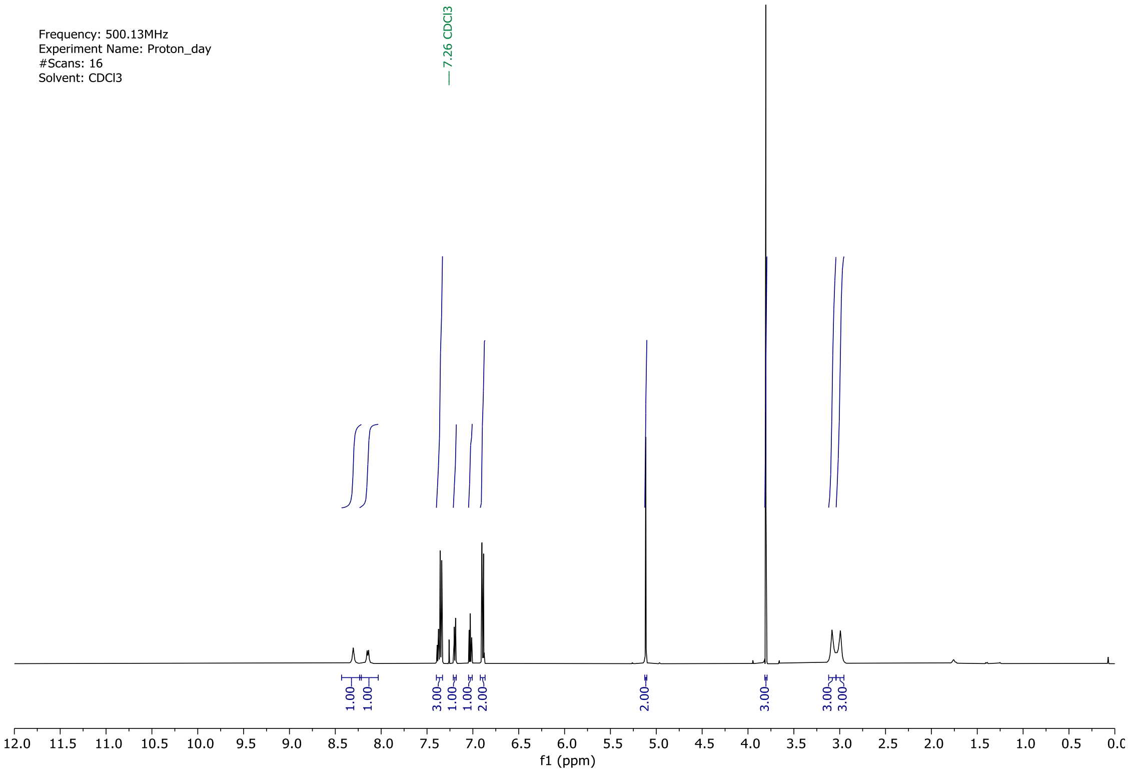

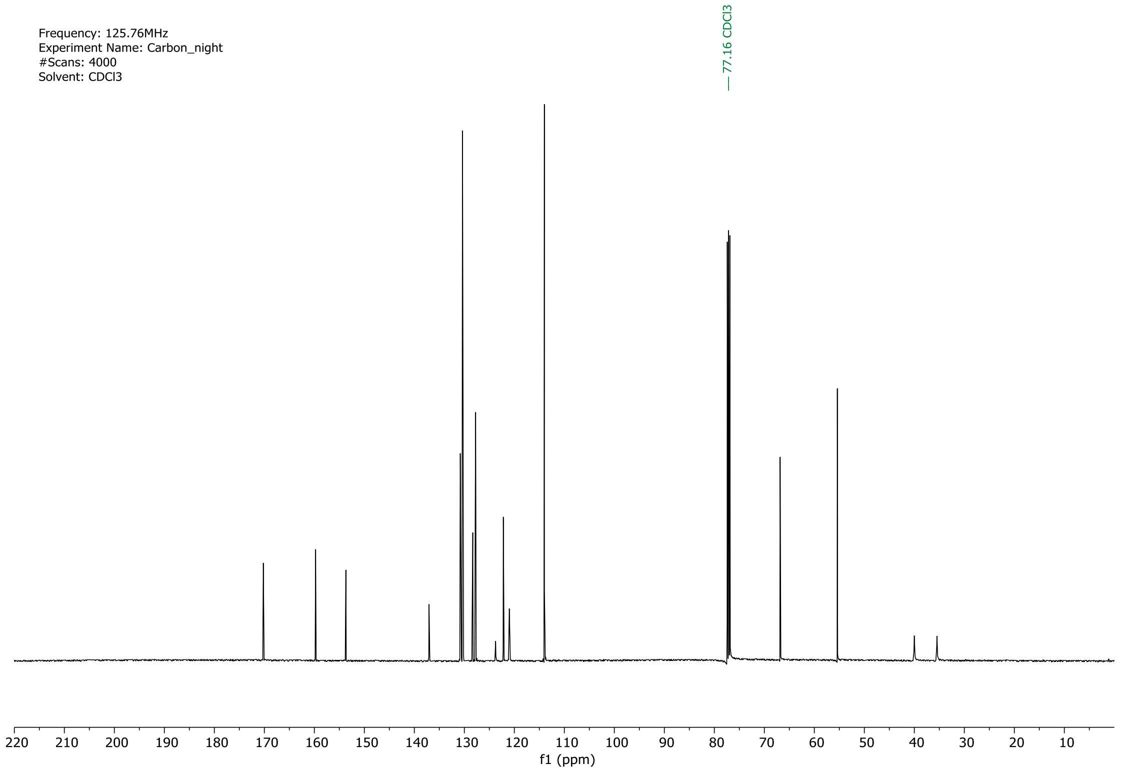

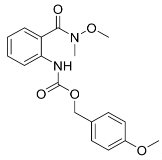

4-methoxybenzyl (2-(methoxy(methyl)carbamoyl)phenyl)carbamate (**2m**)

Frequency: 500.13MHz
Experiment Name: Proton_day
#Scans: 16
Solvent: CDCl3

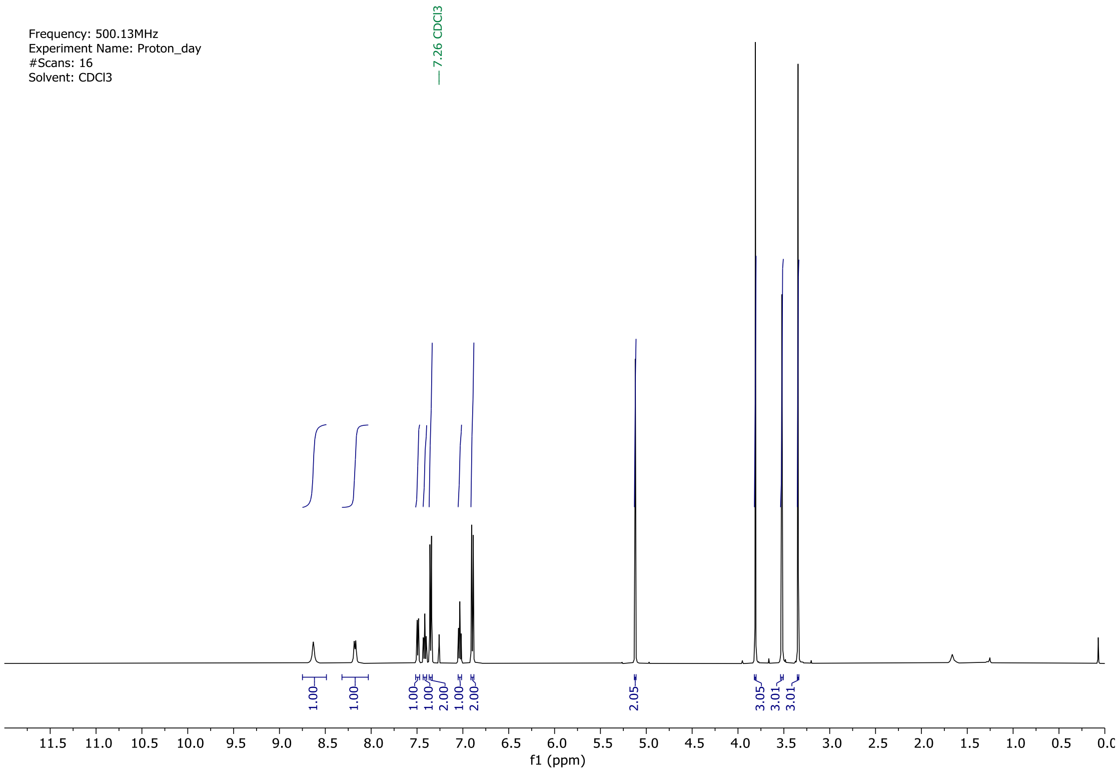

Frequency: 125.76MHz
Experiment Name: Carbon_night
#Scans: 4000
Solvent: CDCl3

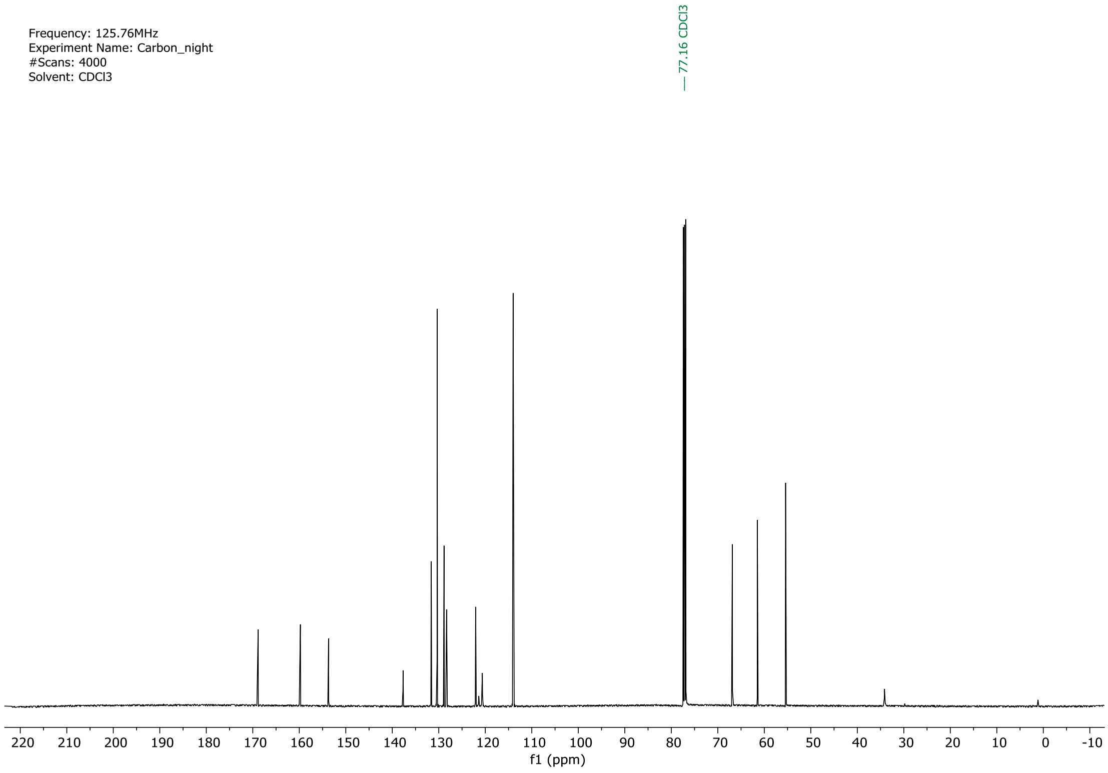

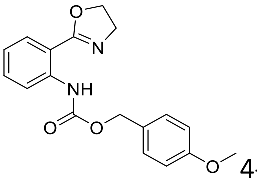

4-methoxybenzyl (2-(4,5-dihydrooxazol-2-yl)phenyl)carbamate (**2n**)

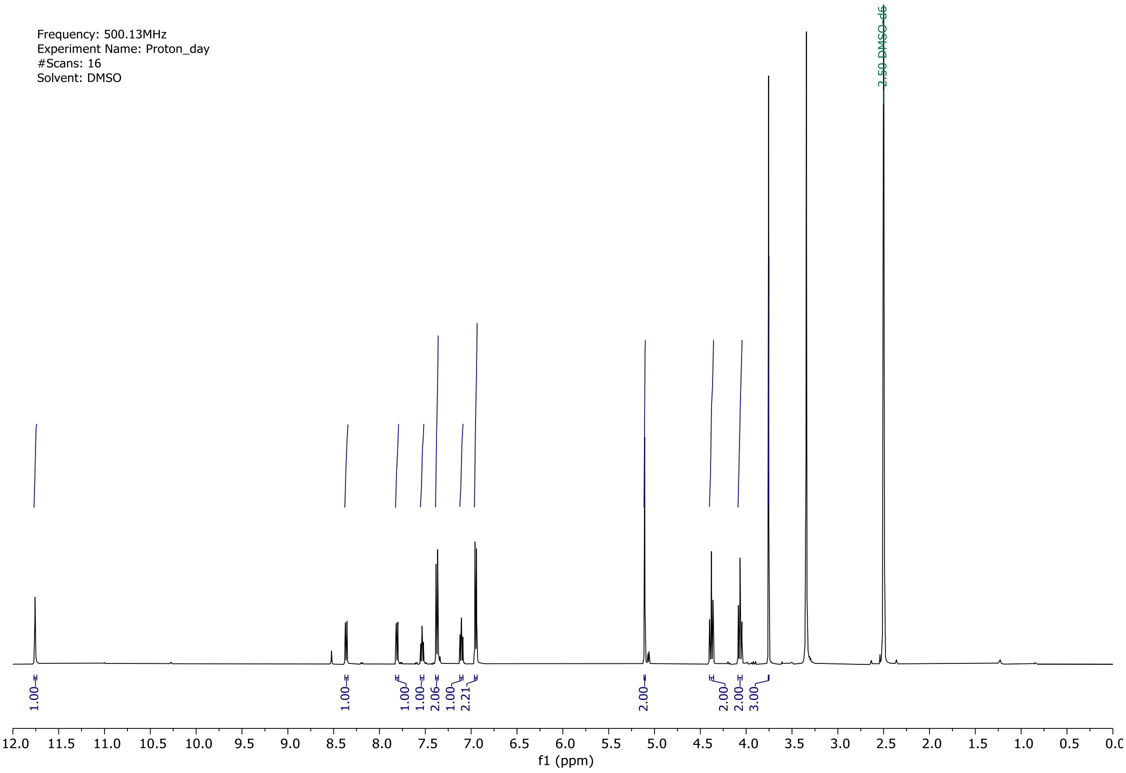

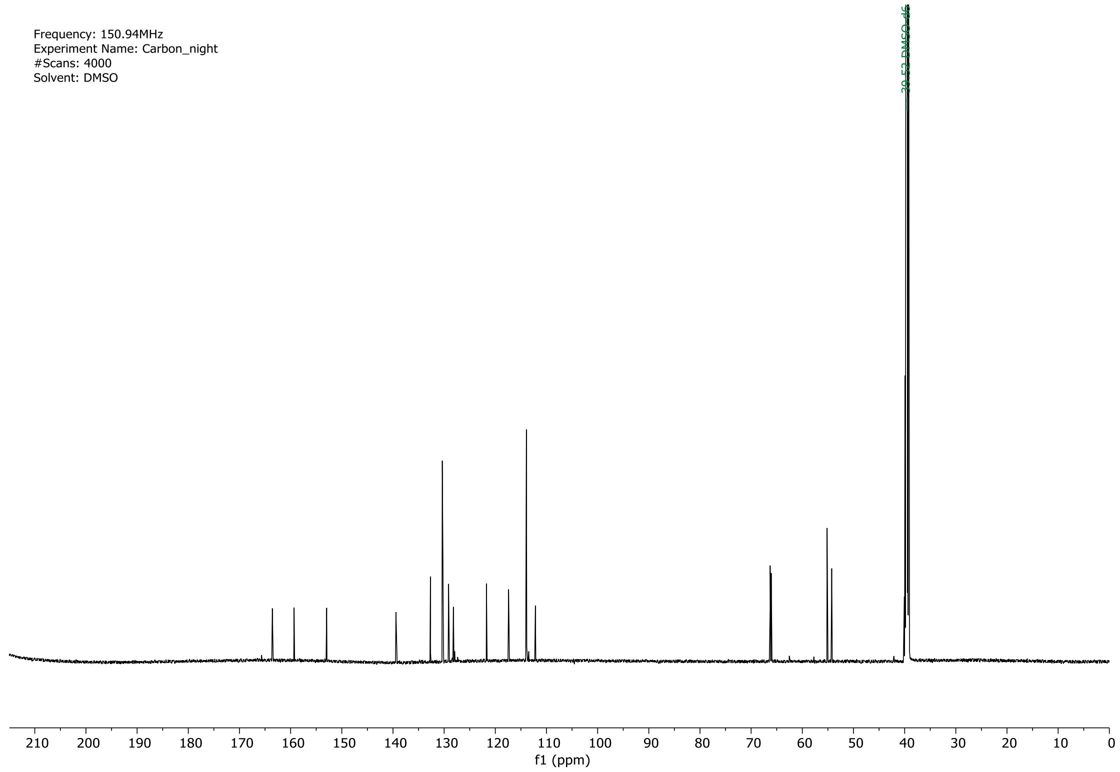

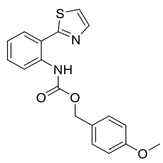

4-methoxybenzyl (2-(thiazol-2-yl)phenyl)carbamate (**2o**)

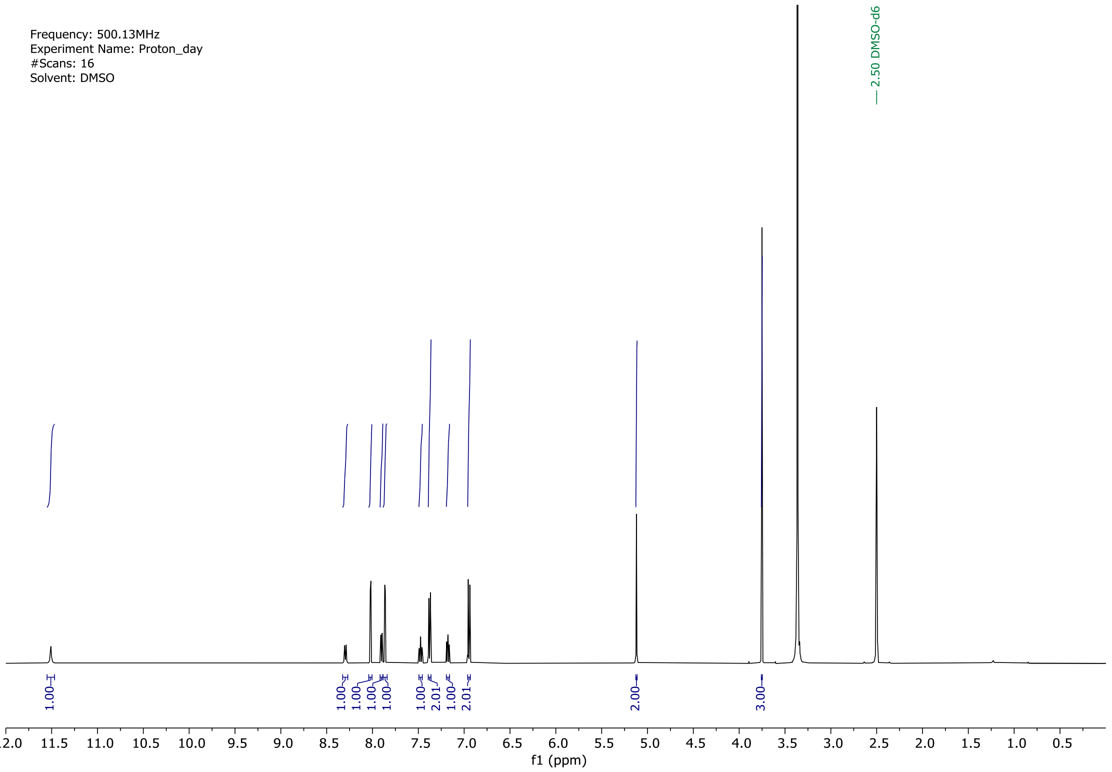

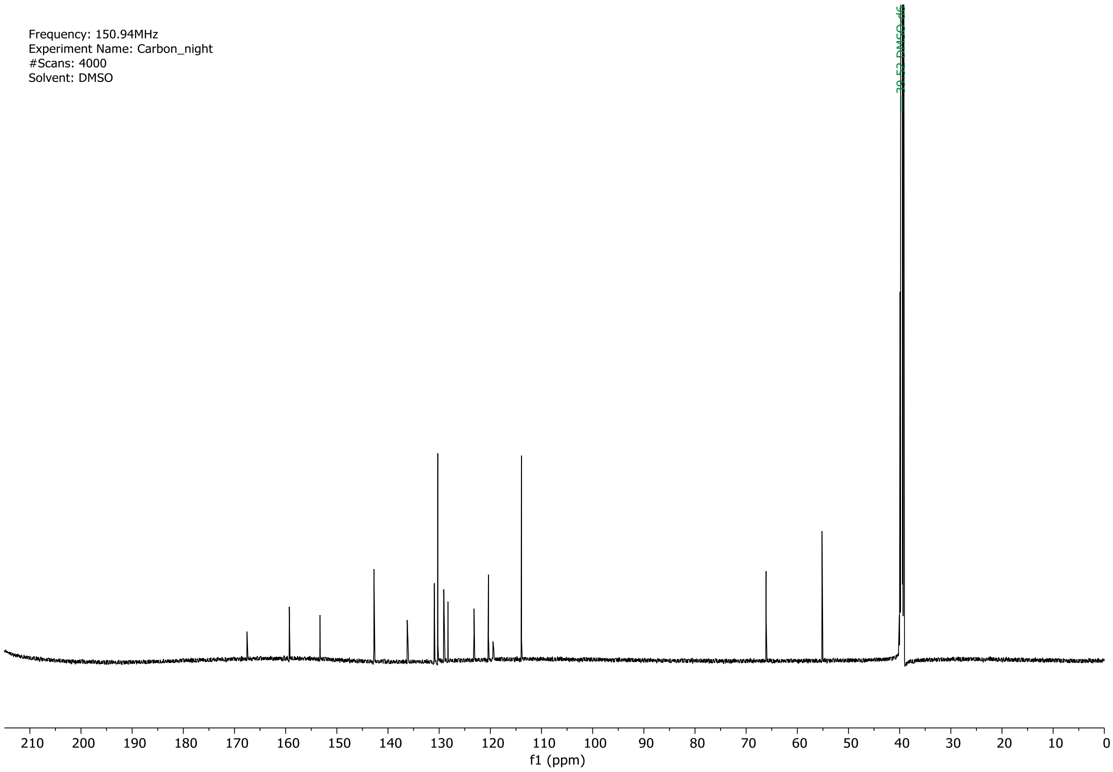

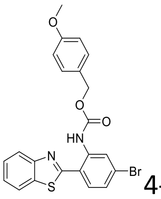

4-methoxybenzyl (2-(benzo[d]thiazol-2-yl)-5-bromophenyl)carbamate (**2p**)

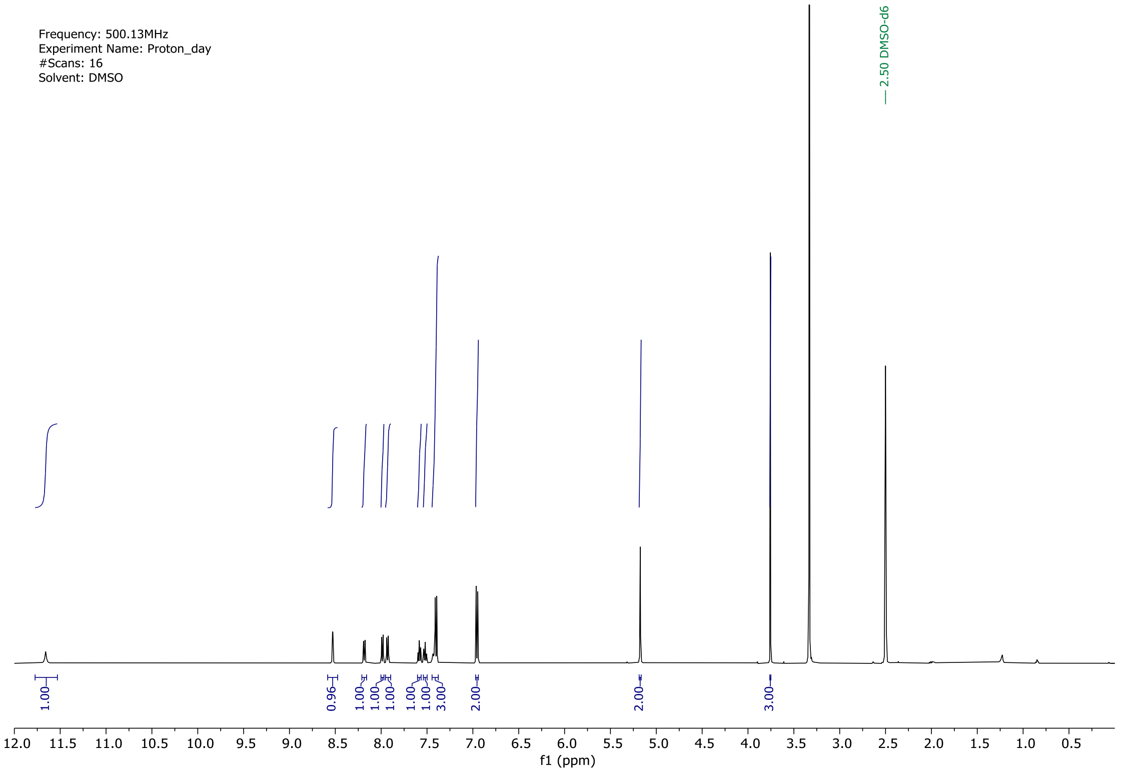

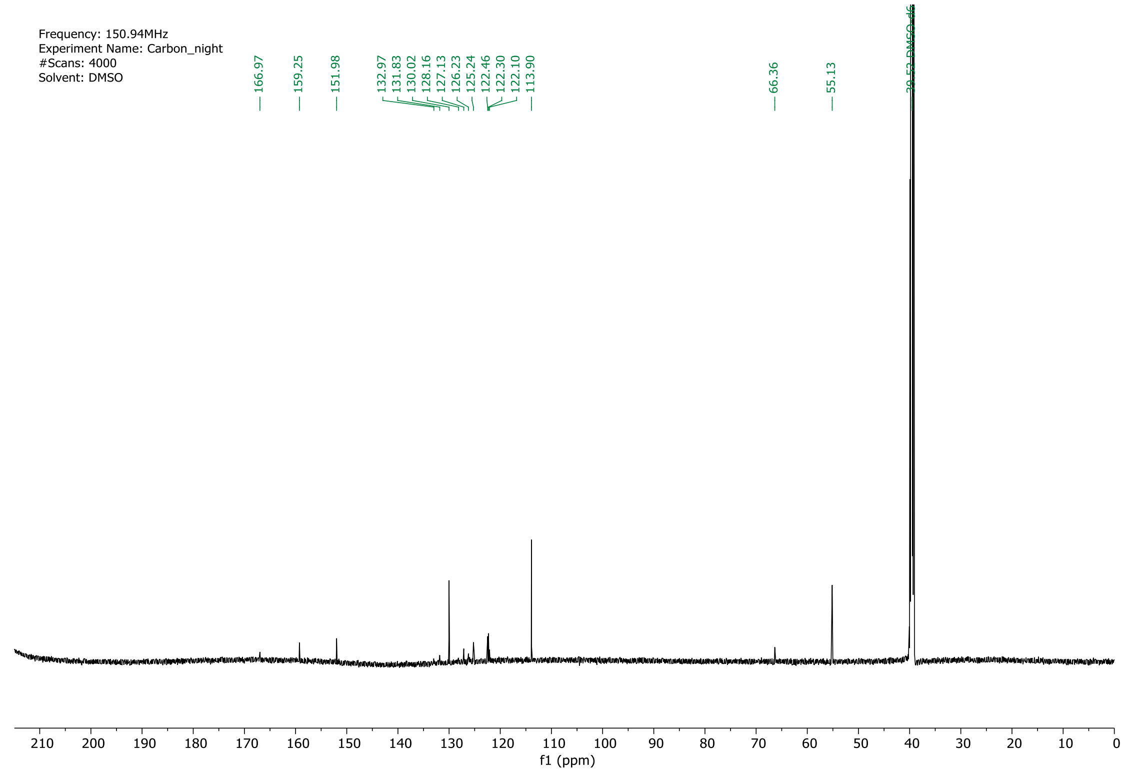

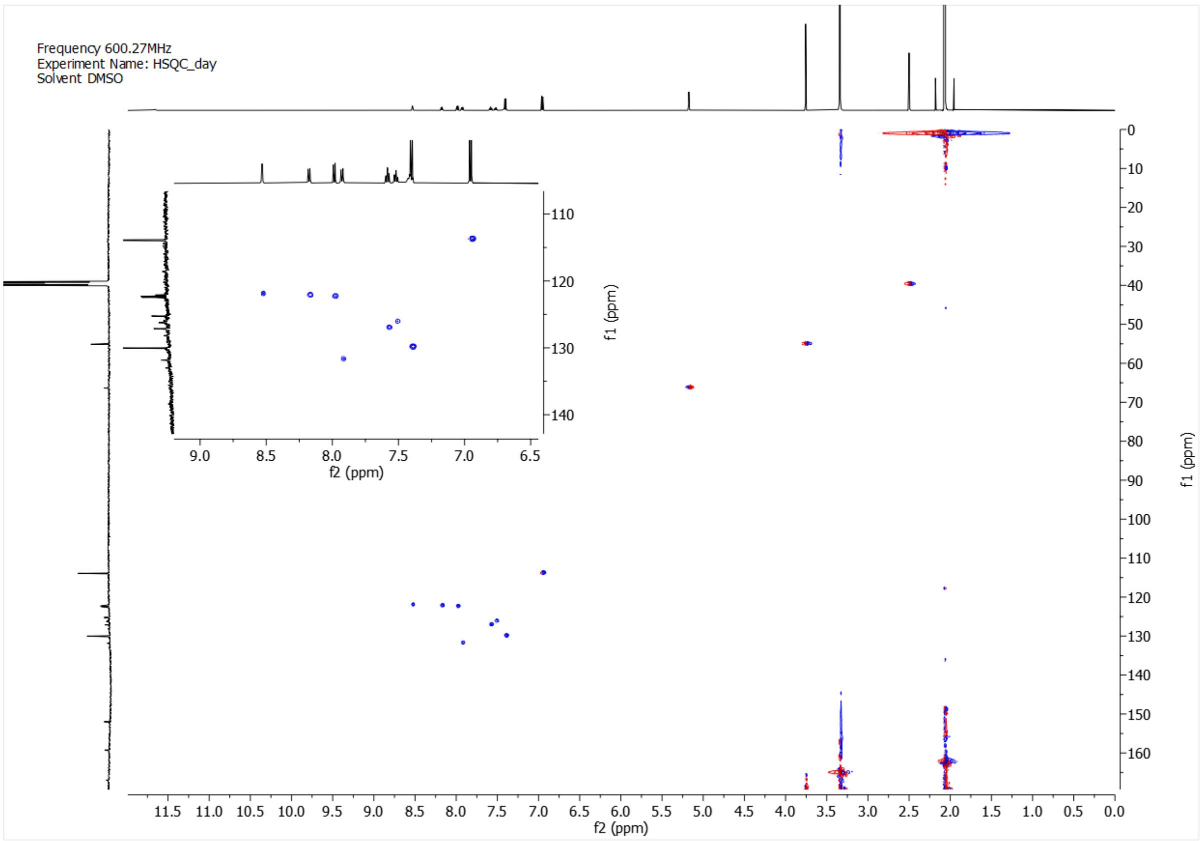
Frequency 600.27MHz
Experiment Name: HSQC_day
Solvent DMSO
f2 (ppm)
f1 (ppm)

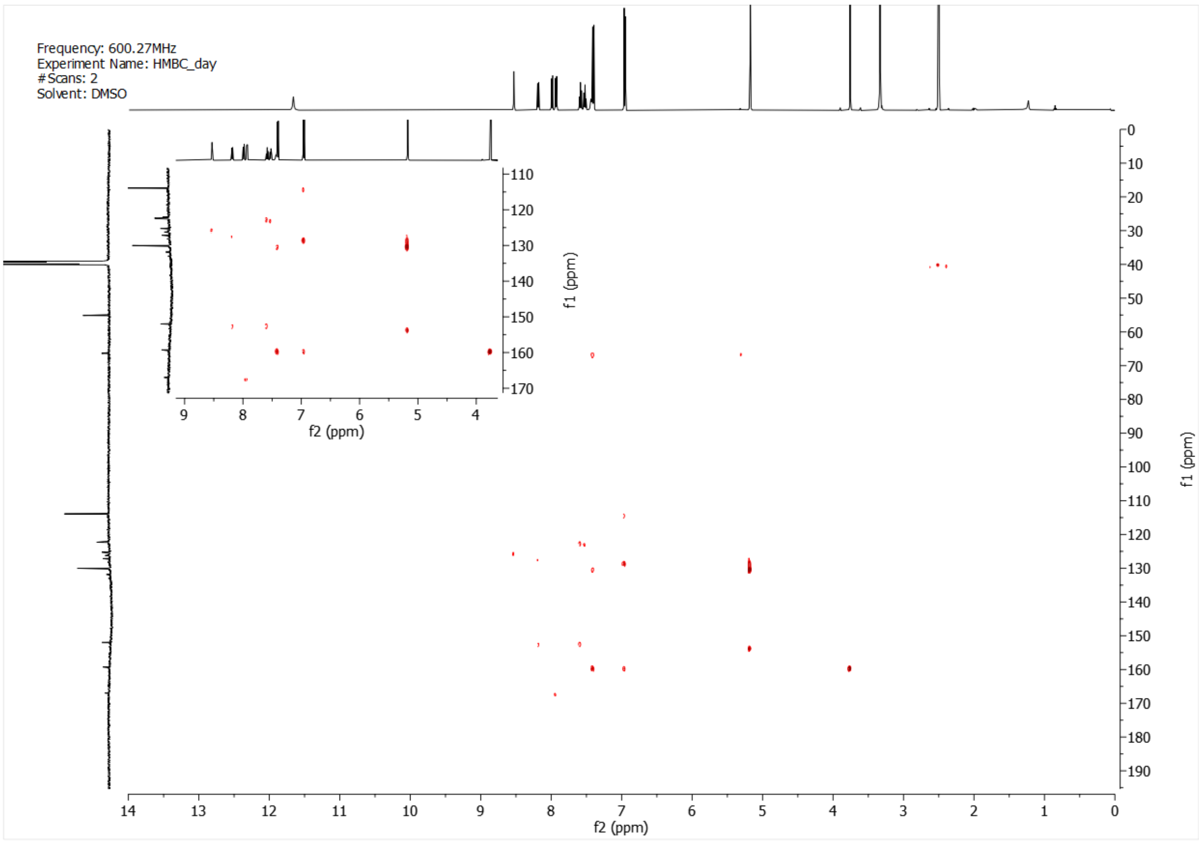
Frequency: 600.27MHz
Experiment Name: HMBC_day
#Scans: 2
Solvent: DMSO
f2 (ppm)
f1 (ppm)

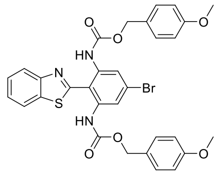

bis(4-methoxybenzyl) (2-(benzo[d]thiazol-2-yl)-5-bromo-1,3-phenylene)dicarbamate (**2p´**)

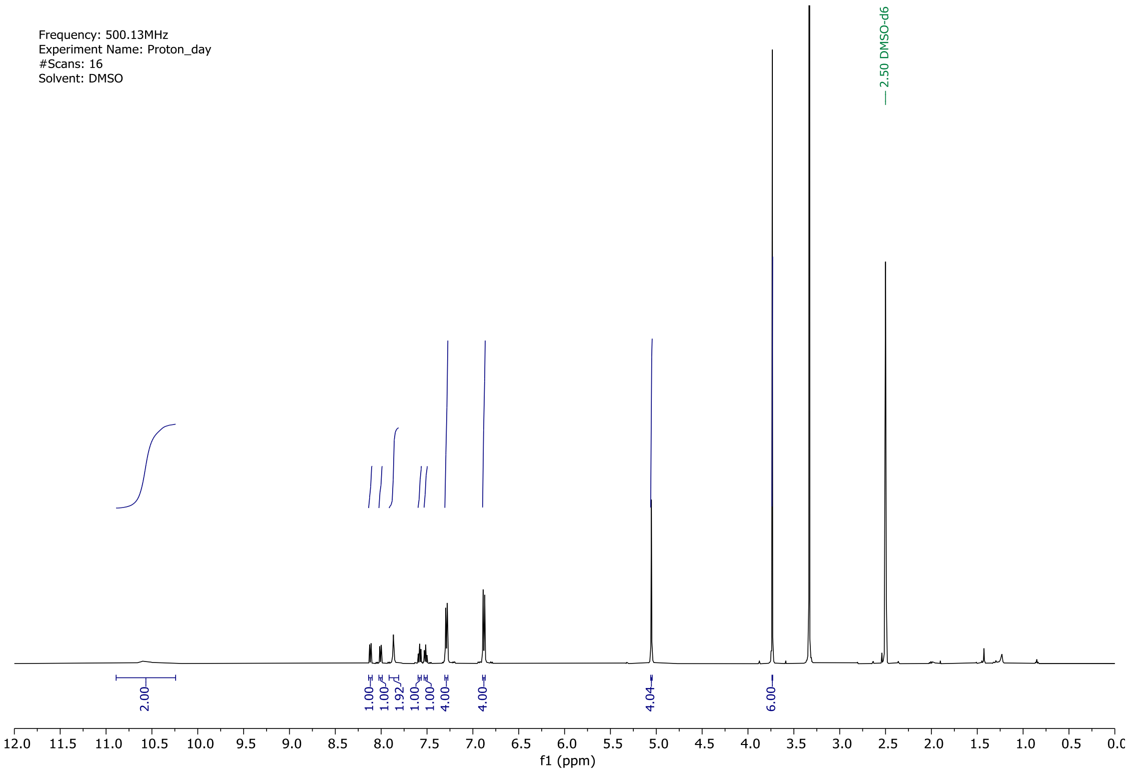

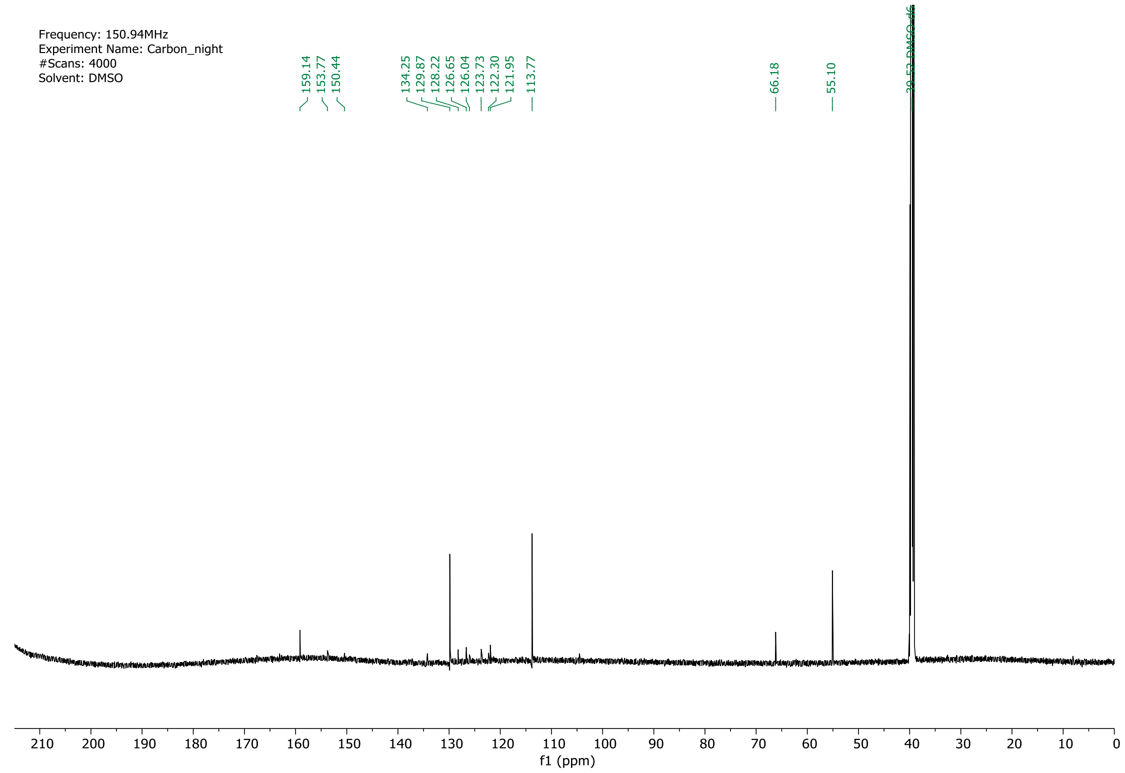

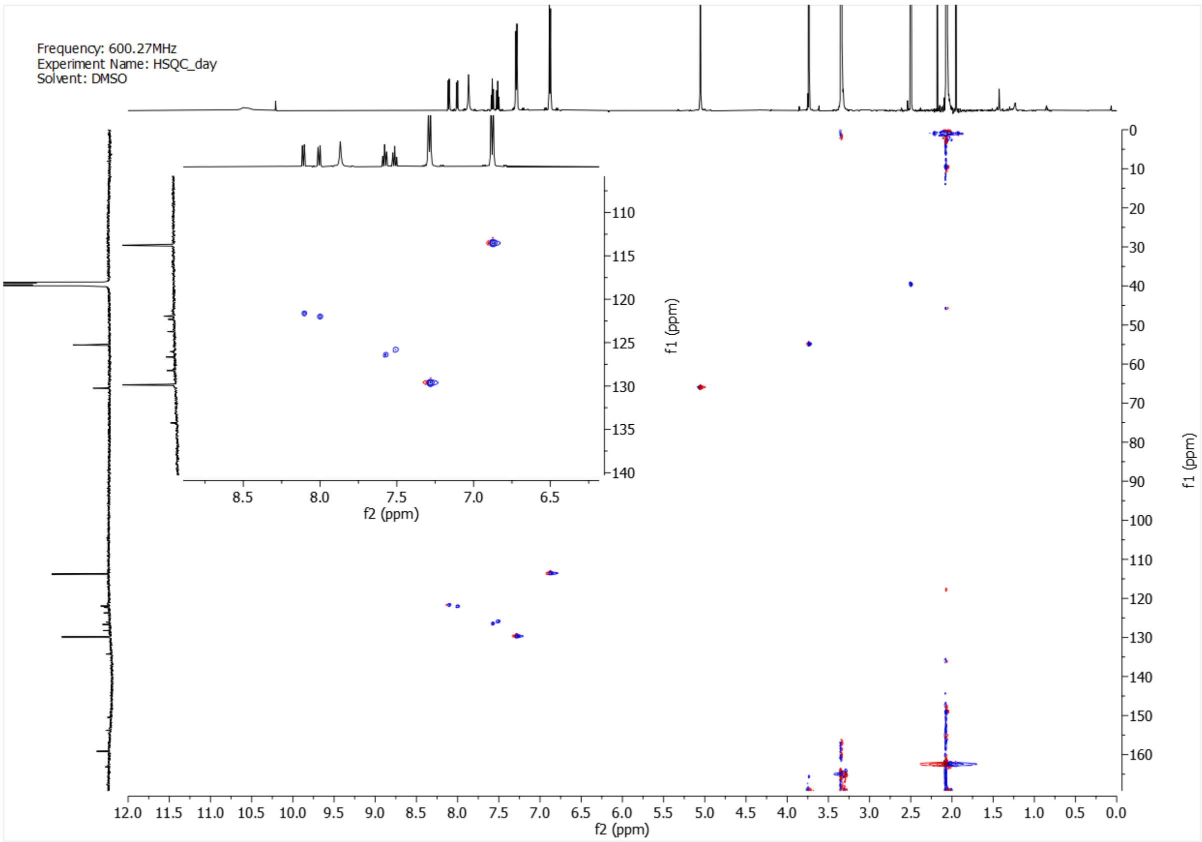
Frequency: 600.27MHz
Experiment Name: HSQC_day
Solvent: DMSO
f1 (ppm)
f2 (ppm)

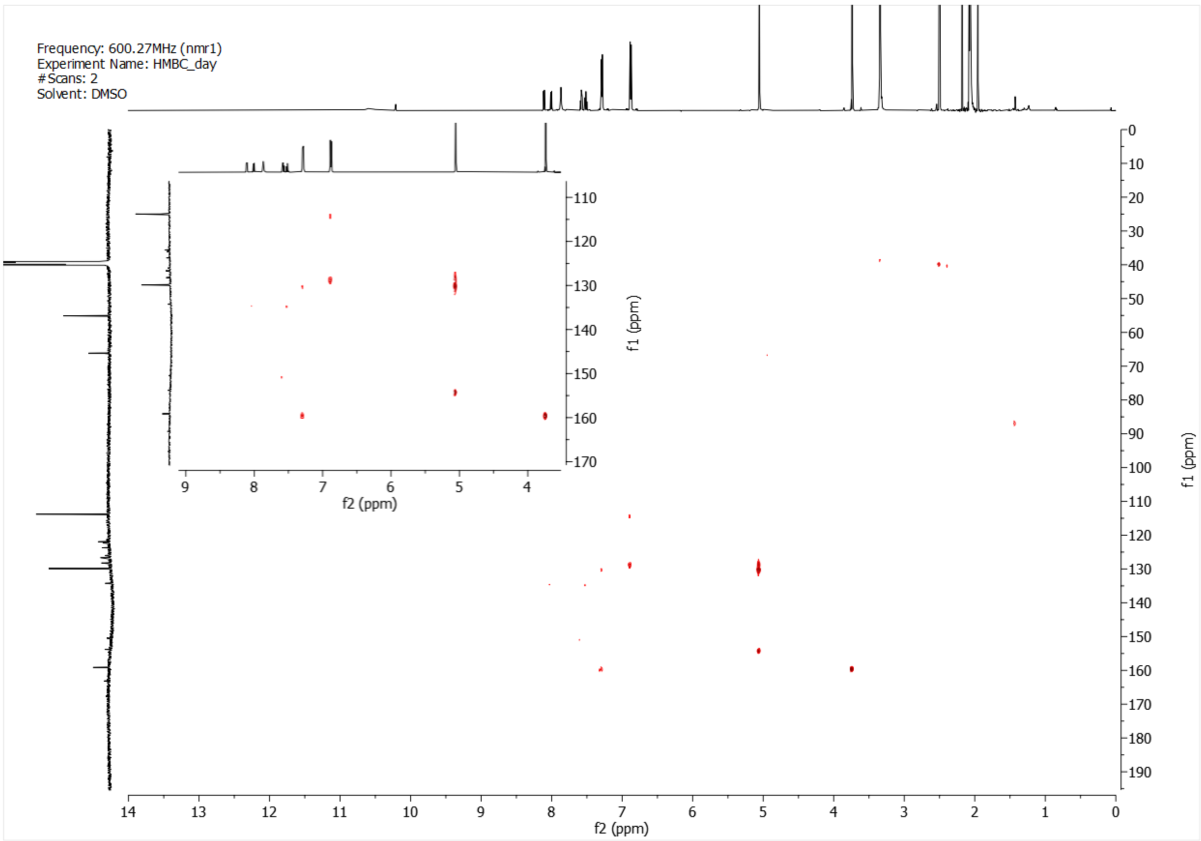
Frequency: 600.27MHz (nmr1)
Experiment Name: HMBC_day
#Scans: 2
Solvent: DMSO
f1 (ppm)
f2 (ppm)

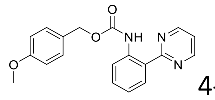

4-methoxybenzyl (2-(pyrimidin-2-yl)phenyl)carbamate (**2q**)

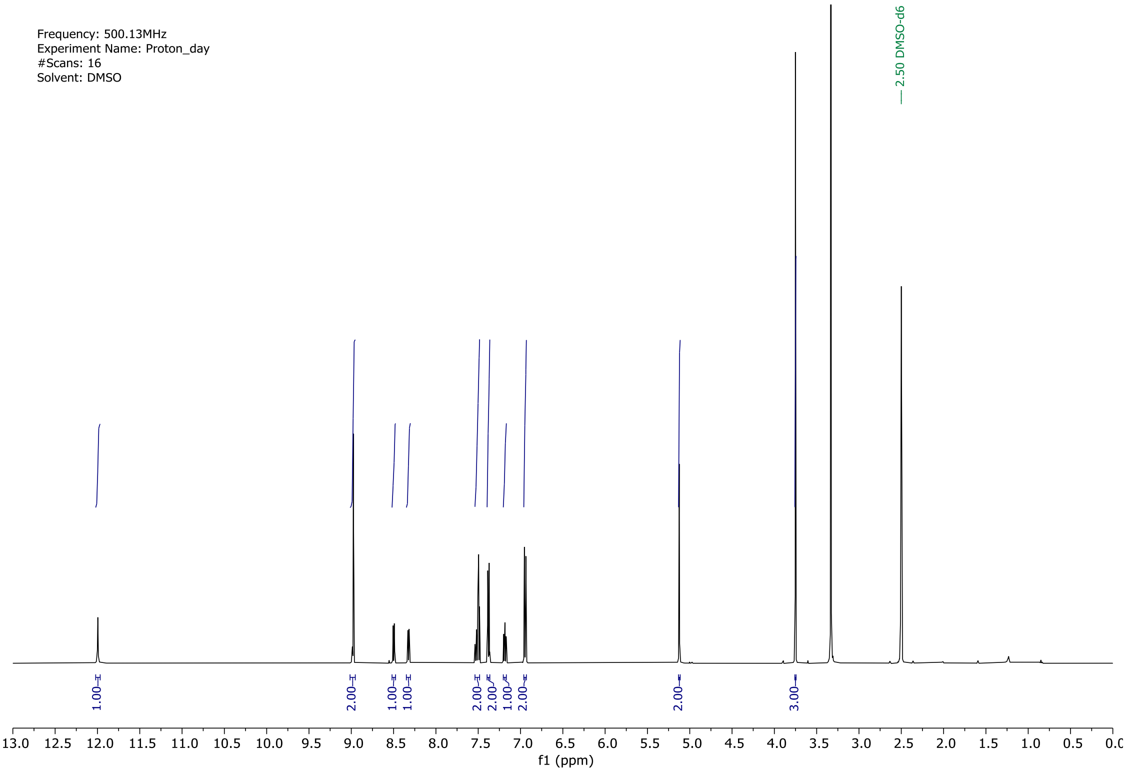

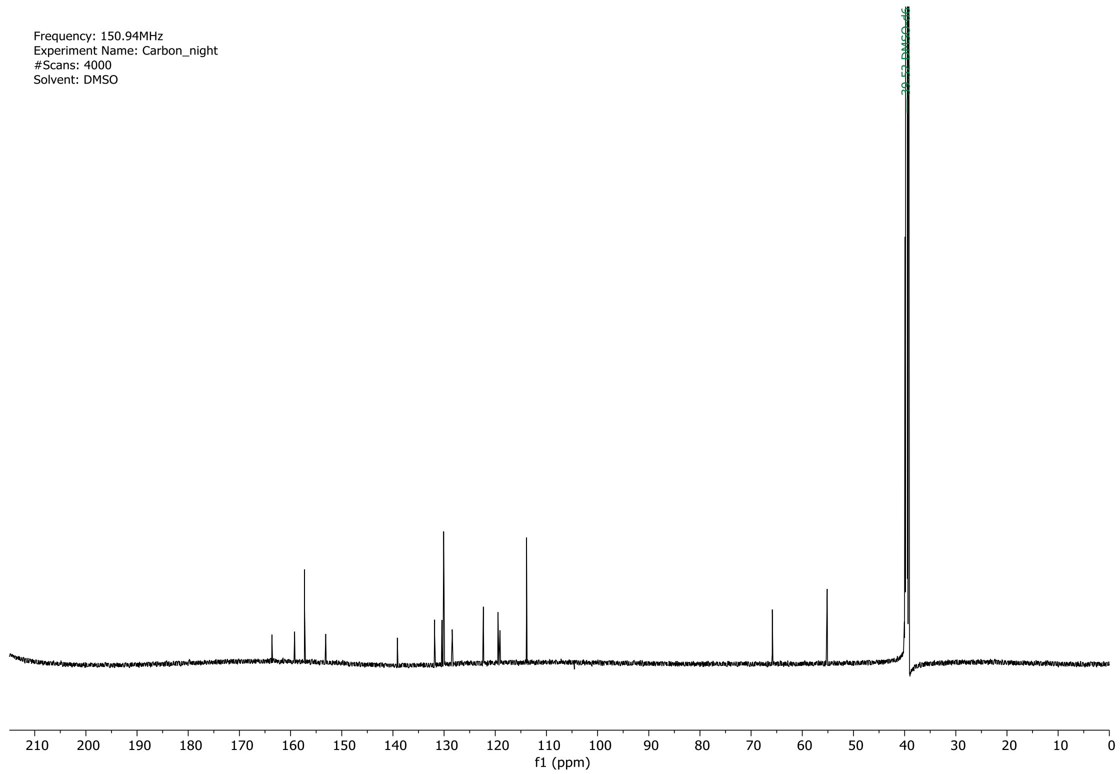

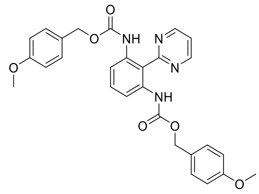

bis(4-methoxybenzyl) (2-(pyrimidin-2-yl)-1,3-phenylene)dicarbamate (**2q´**)

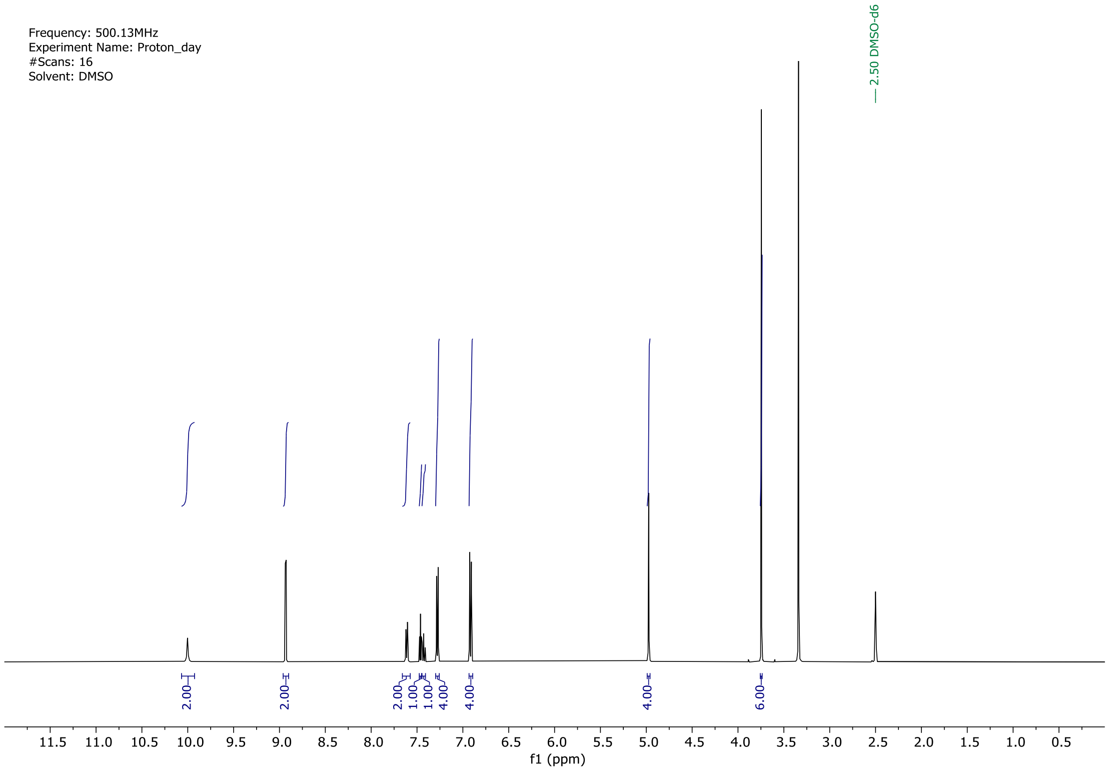

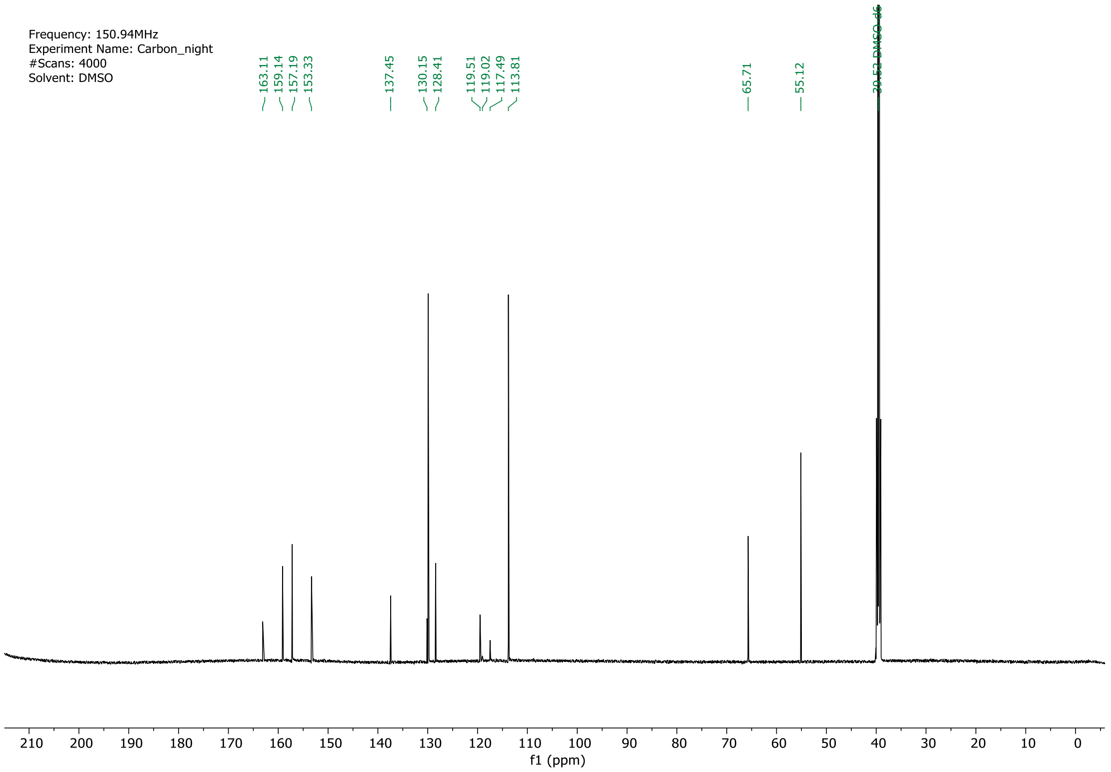

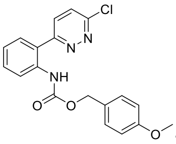

4-methoxybenzyl (2-(6-chloropyridazin-3-yl)phenyl)carbamate (**2r**)

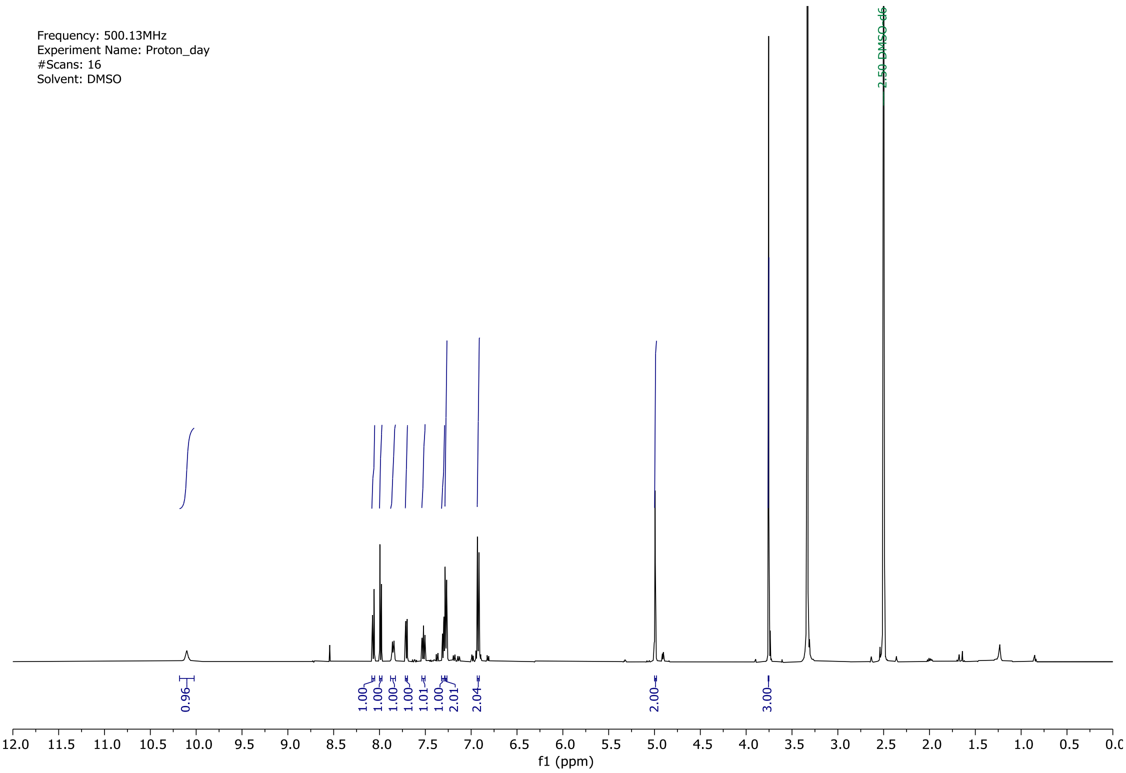

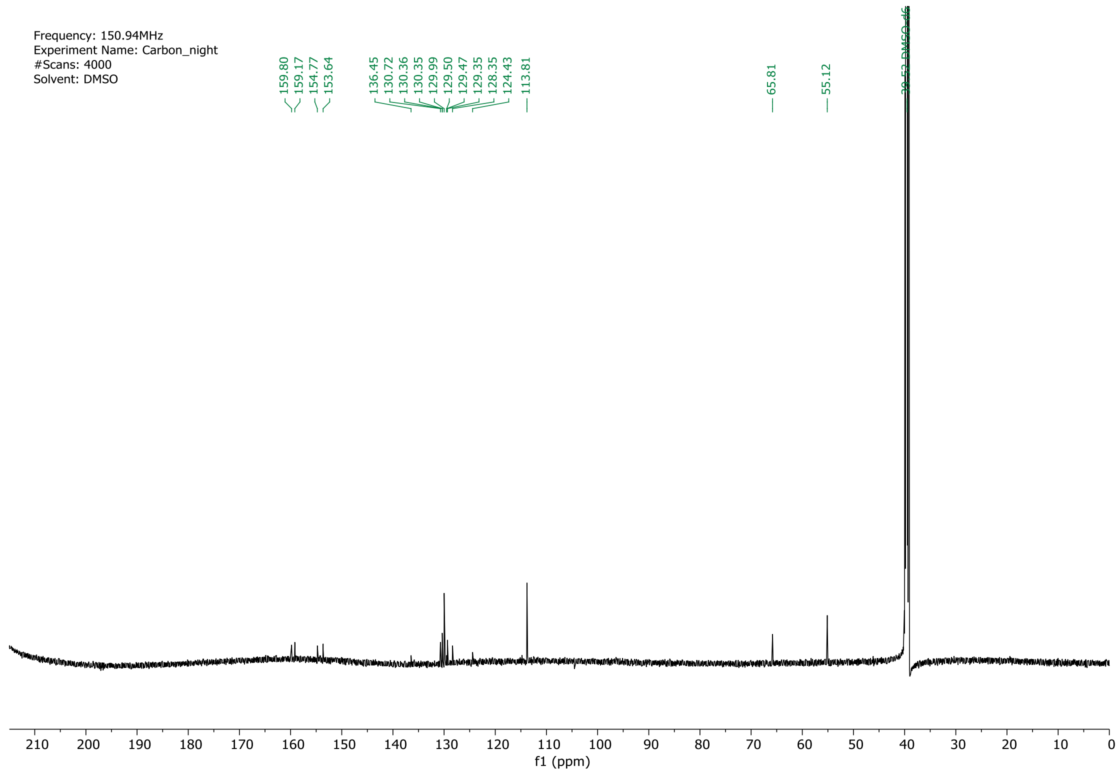

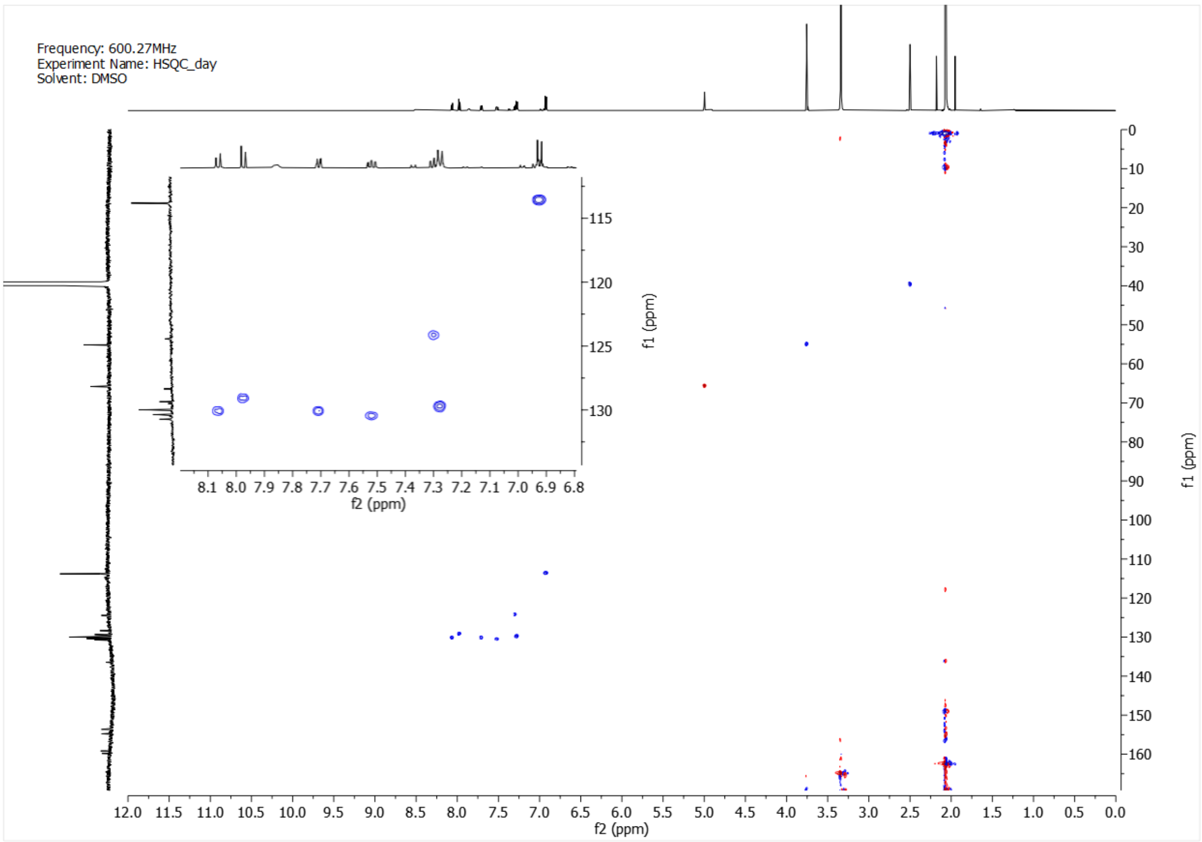
Frequency: 600.27MHz
Experiment Name: HSQC_day
Solvent: DMSO
f2 (ppm)
f1 (ppm)

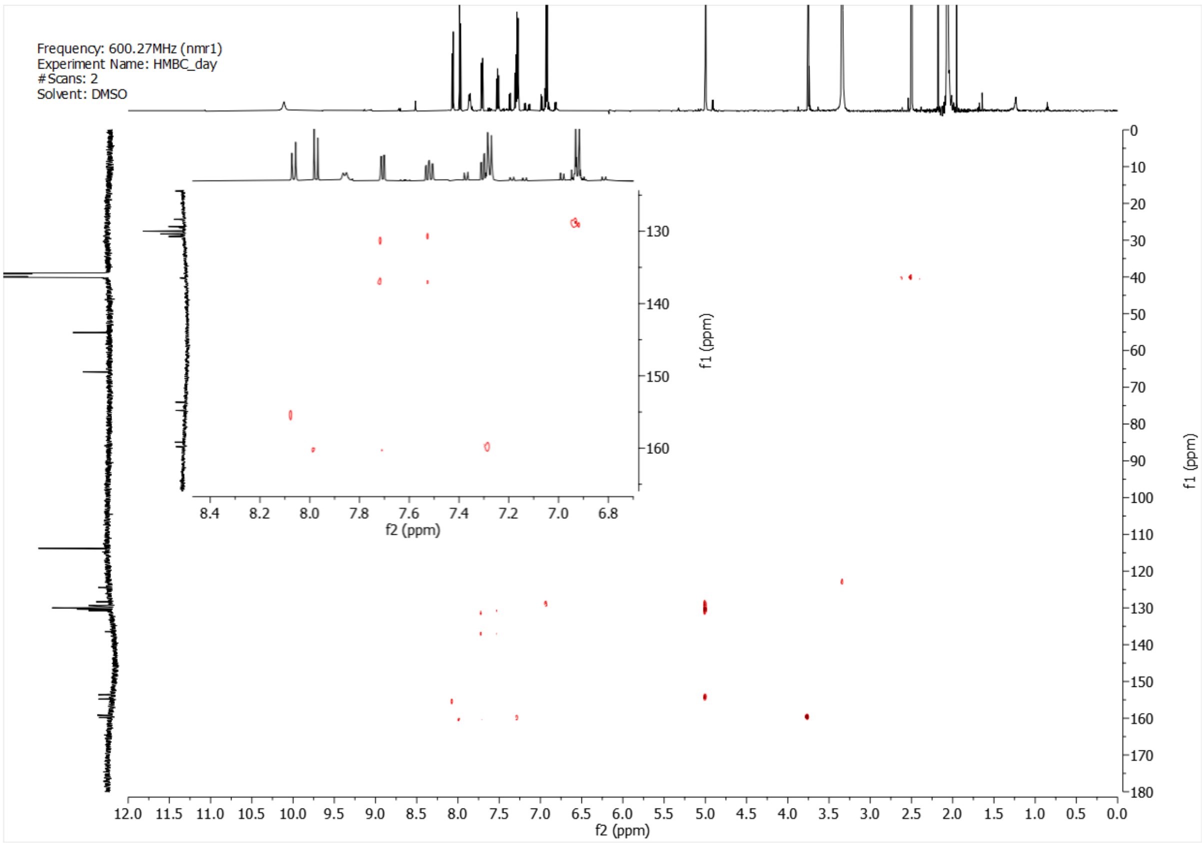
Frequency: 600.27MHz (nmr1)
Experiment Name: HMBC_day
#Scans: 2
Solvent: DMSO
f2 (ppm)
f1 (ppm)

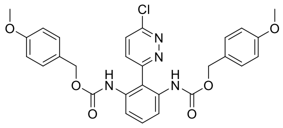

bis(4-methoxybenzyl) (2-(6-chloropyridazin-3-yl)-1,3-phenylene)dicarbamate (**2r´**)

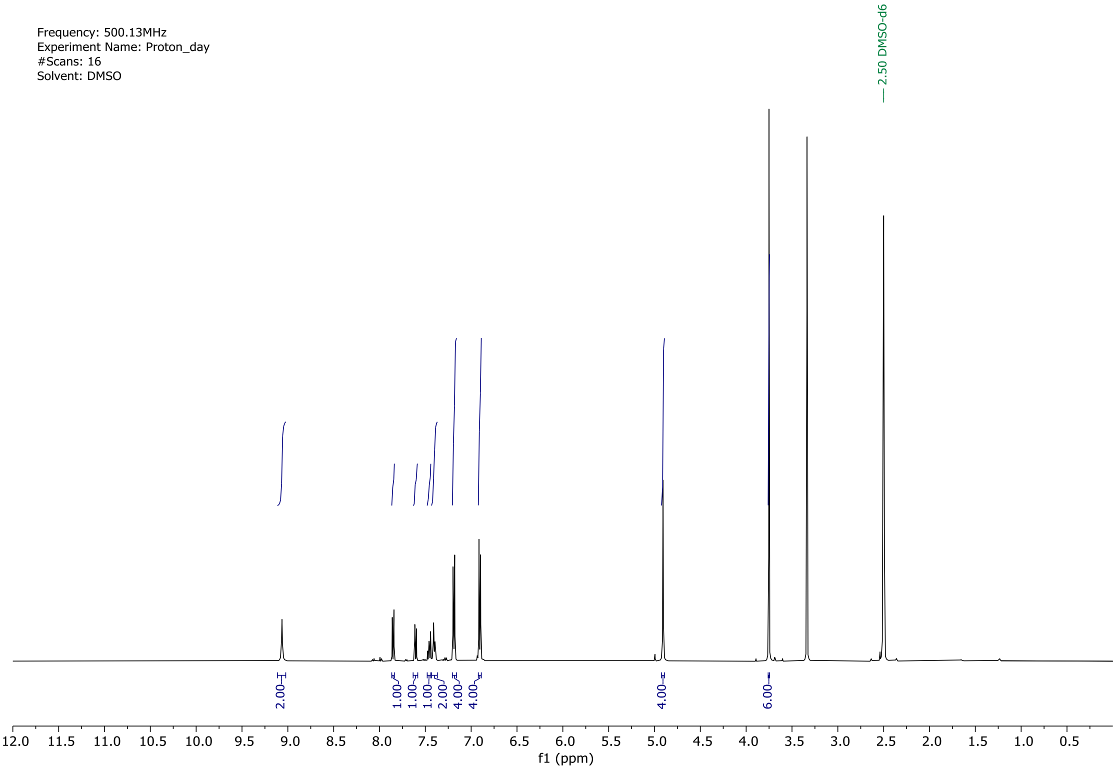

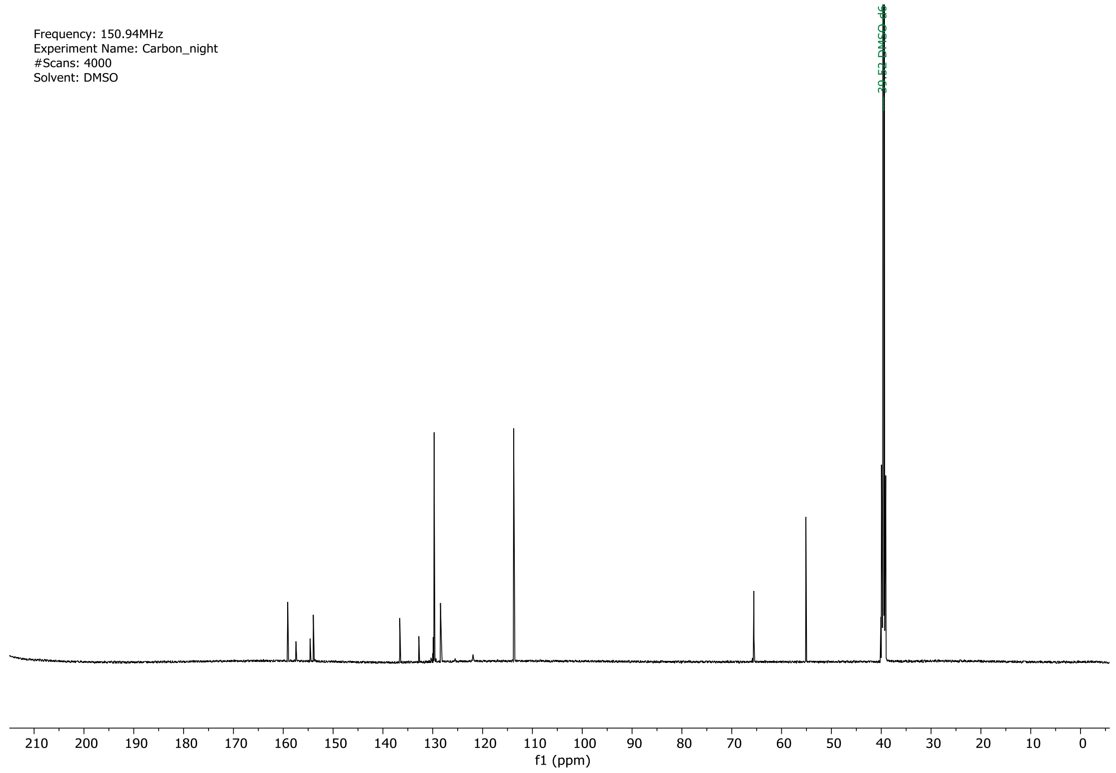

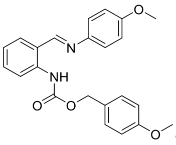

4-methoxybenzyl (*E*)-(2-(((4-methoxyphenyl)imino)methyl)phenyl)carbamate (**2s**)

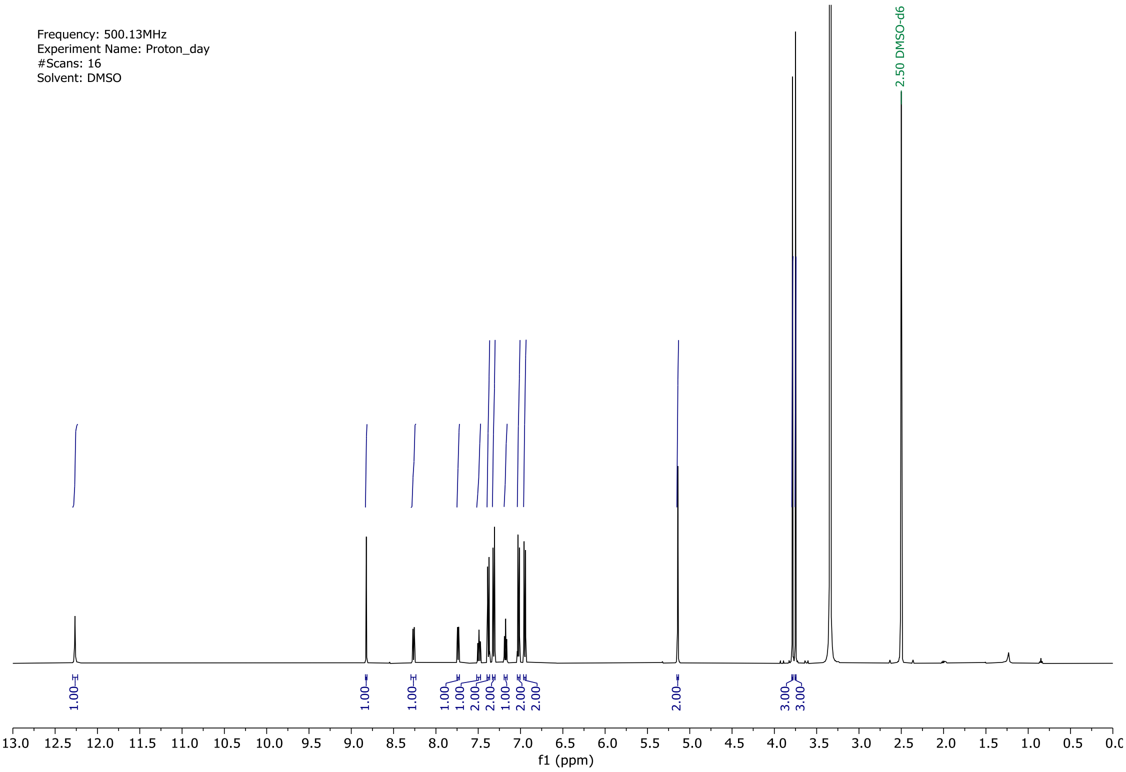

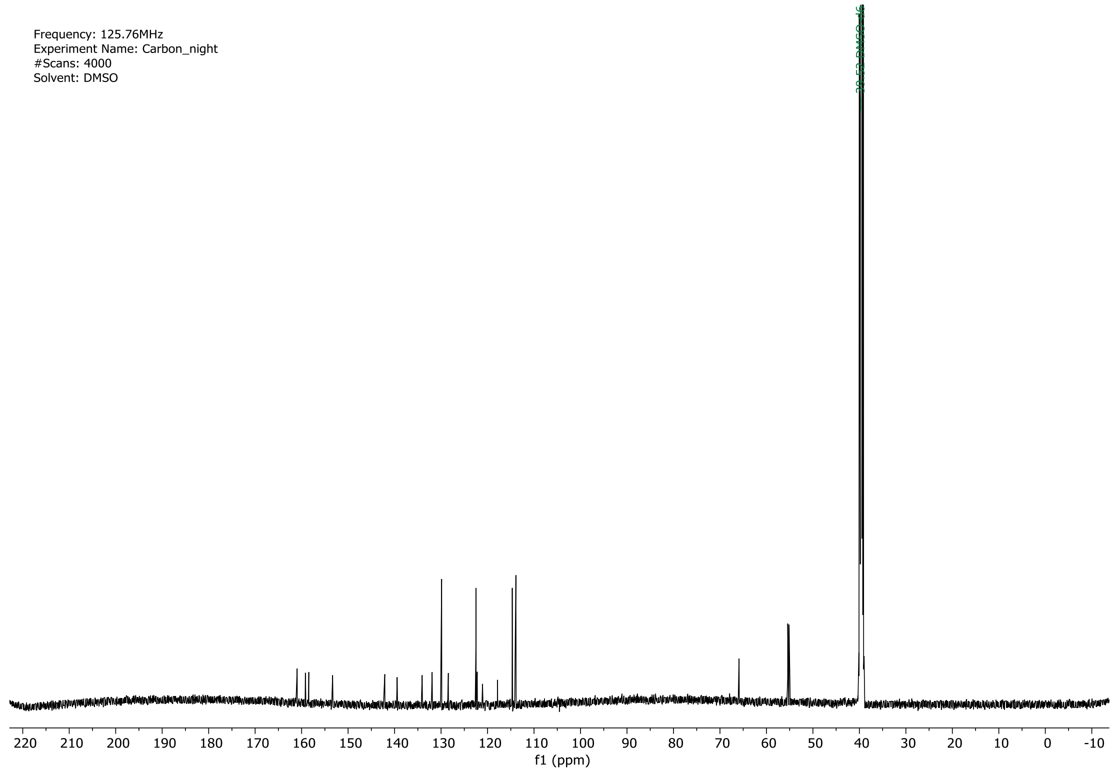

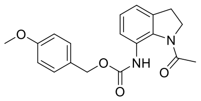

4-methoxybenzyl (1-acetylindolin-7-yl)carbamate (**2t**)

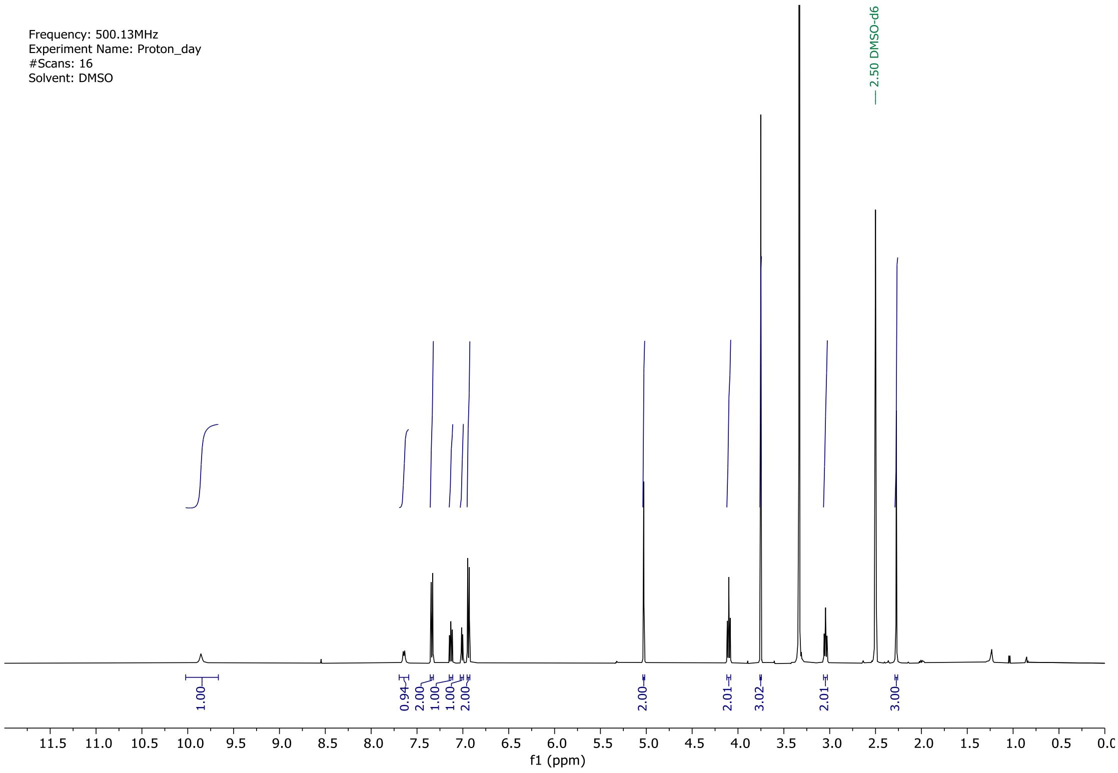

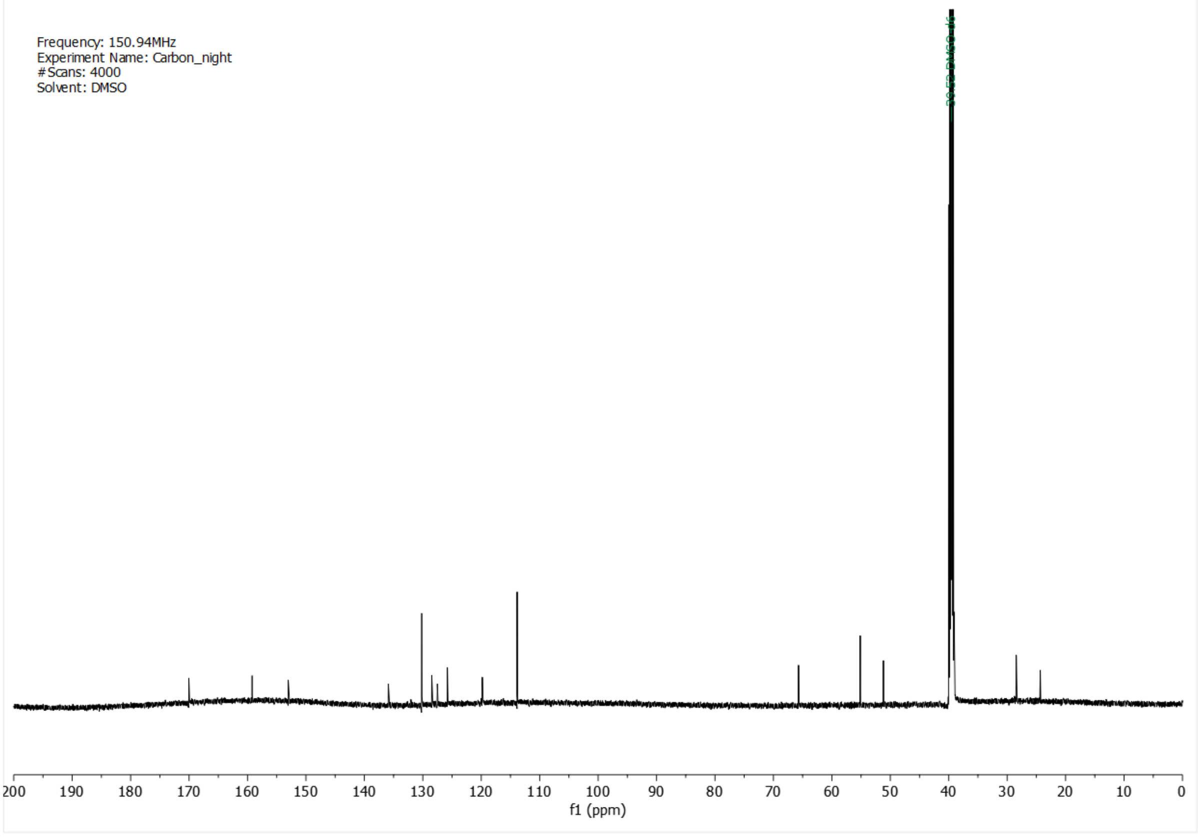

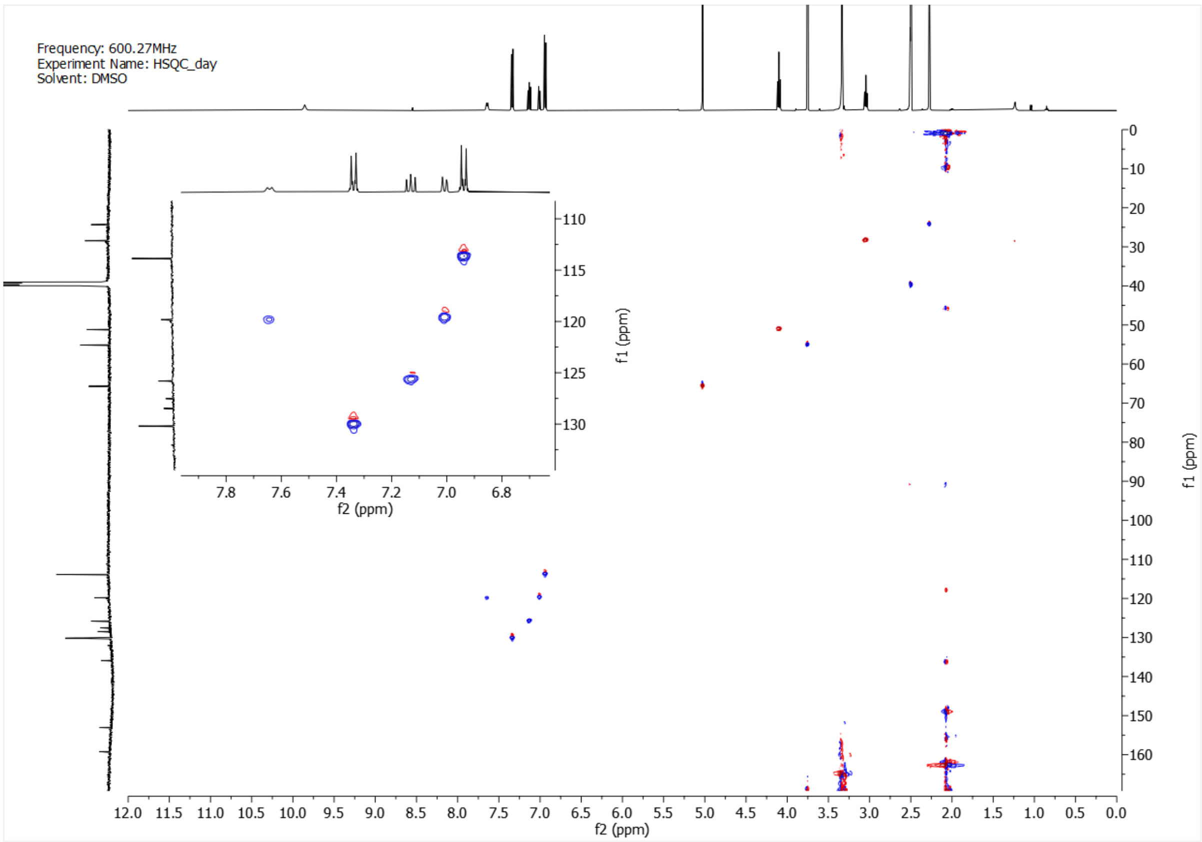
Frequency: 600.27MHz
Experiment Name: HSQC_day
Solvent: DMSO
f1 (ppm)
f2 (ppm)

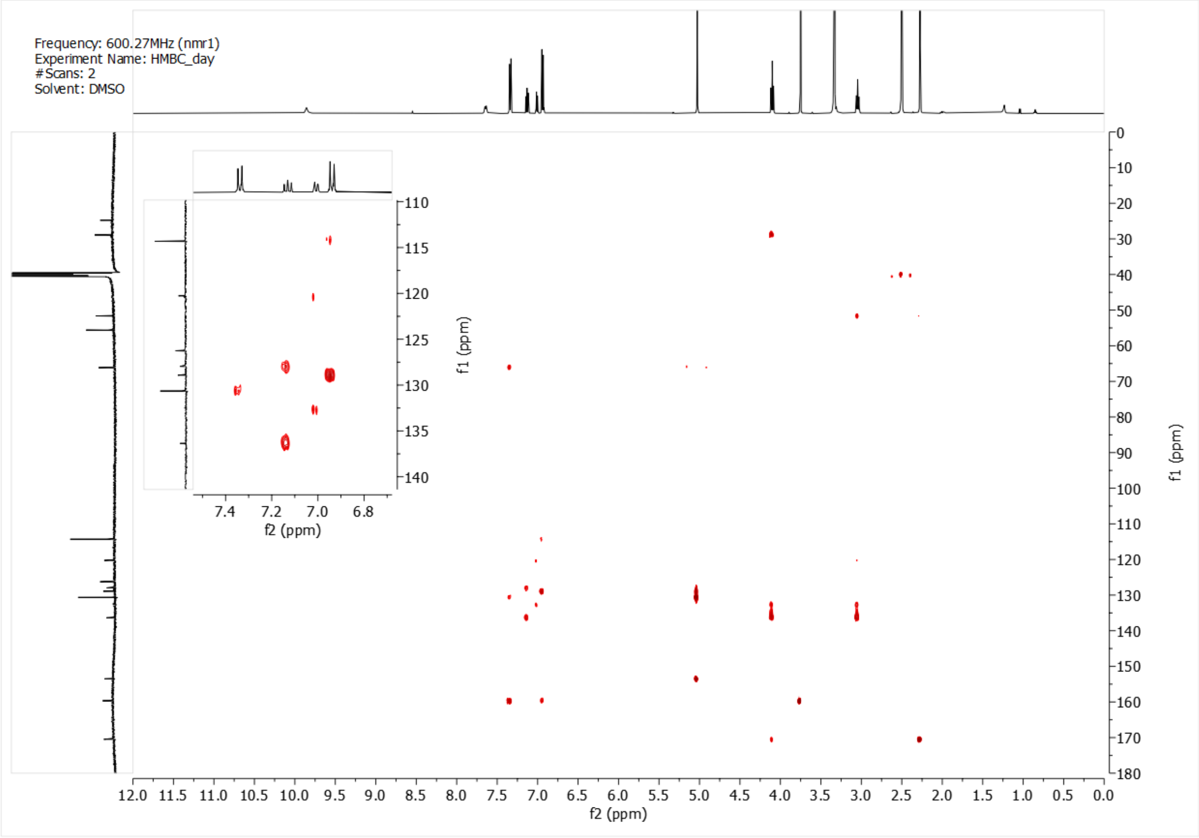
Frequency: 600.27MHz (nmr1)
Experiment Name: HMBC_day
#Scans: 2
Solvent: DMSO
f1 (ppm)
f2 (ppm)

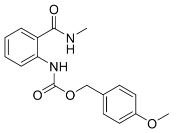

4-methoxybenzyl (2-(methylcarbamoyl)phenyl)carbamate (**2u**)

Frequency: 500.13MHz
Experiment Name: Proton_day
#Scans: 16
Solvent: DMSO

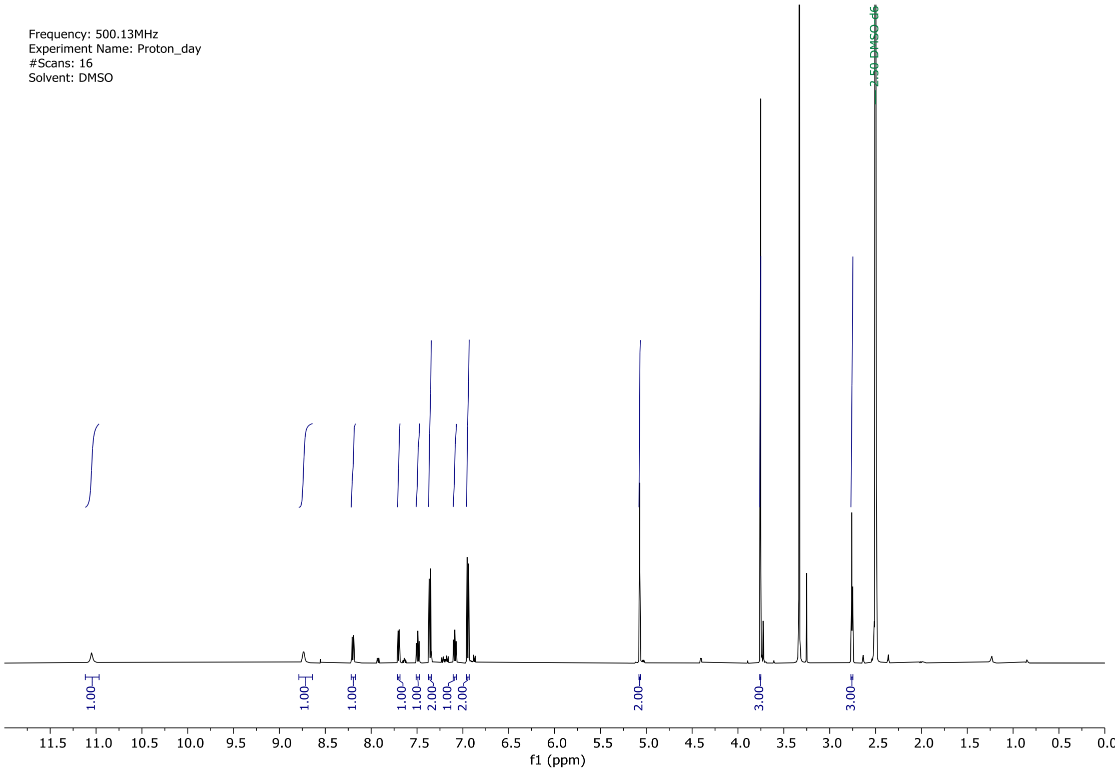

Frequency: 150.94MHz
Experiment Name: Carbon_night
#Scans: 4000
Solvent: DMSO

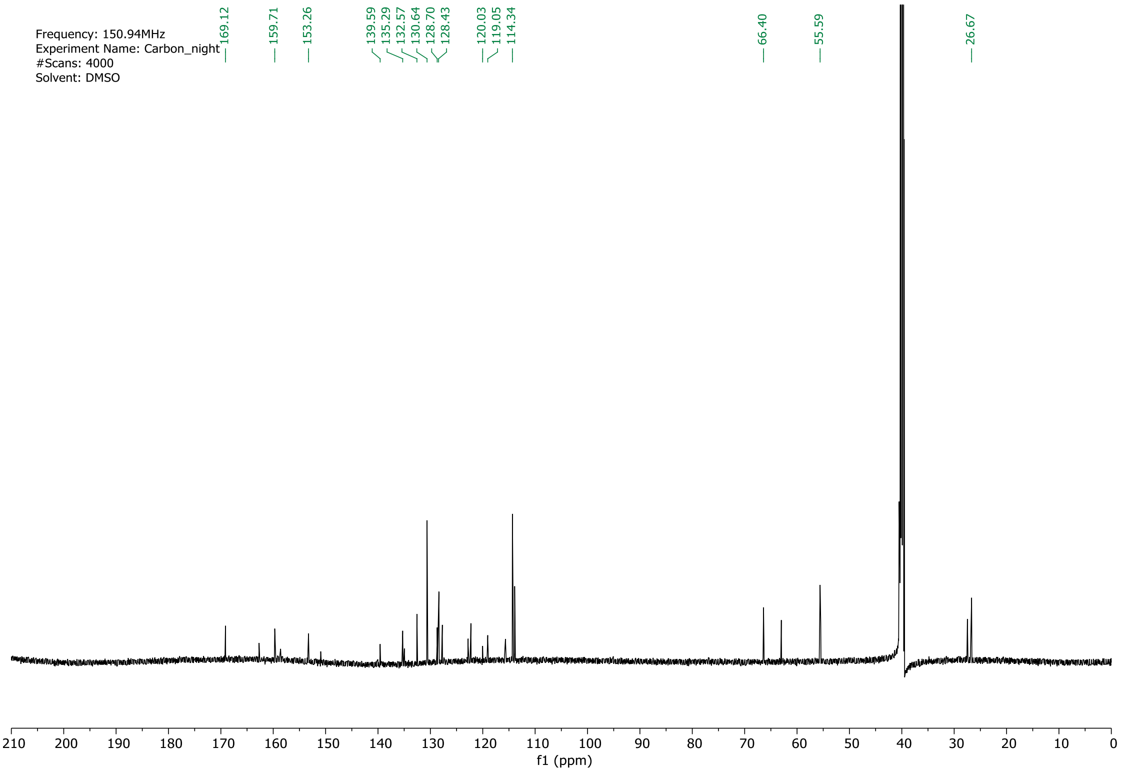

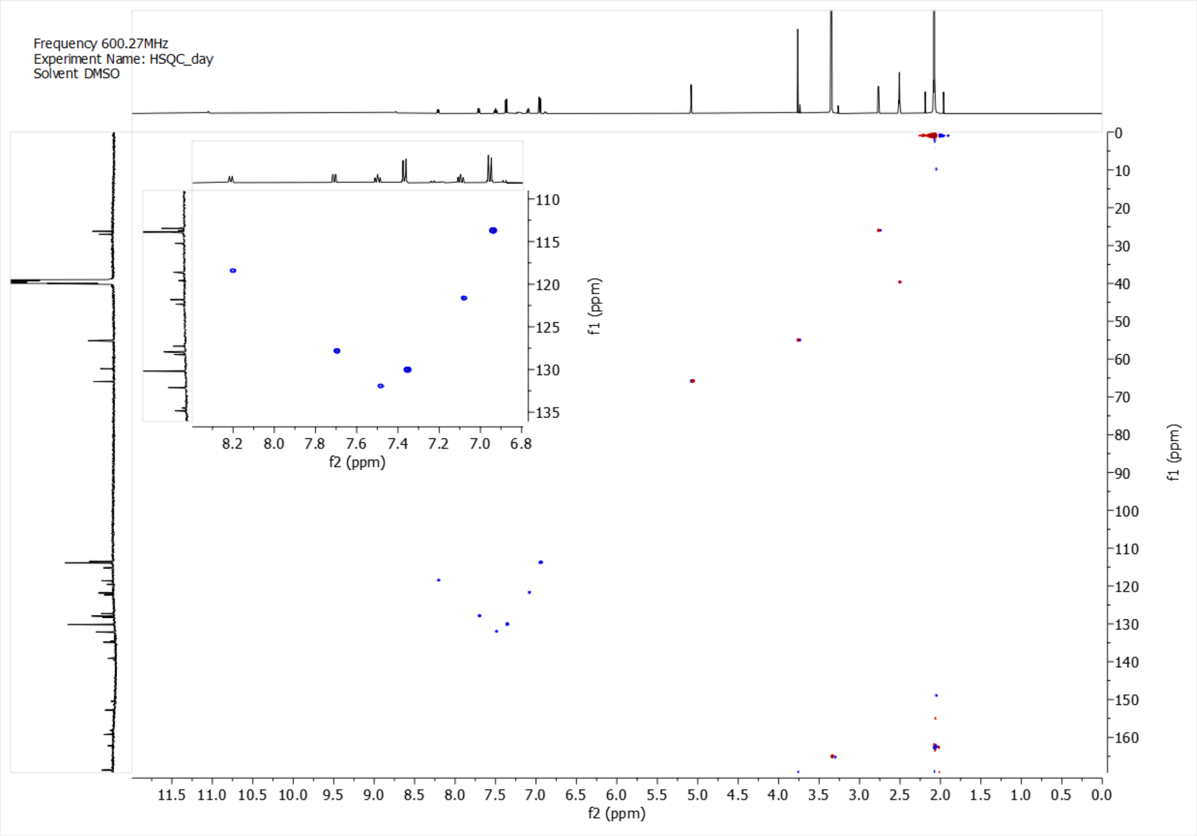
Frequency 600.27MHz
Experiment Name: HSQC_day
Solvent DMSO
f1 (ppm)
f2 (ppm)

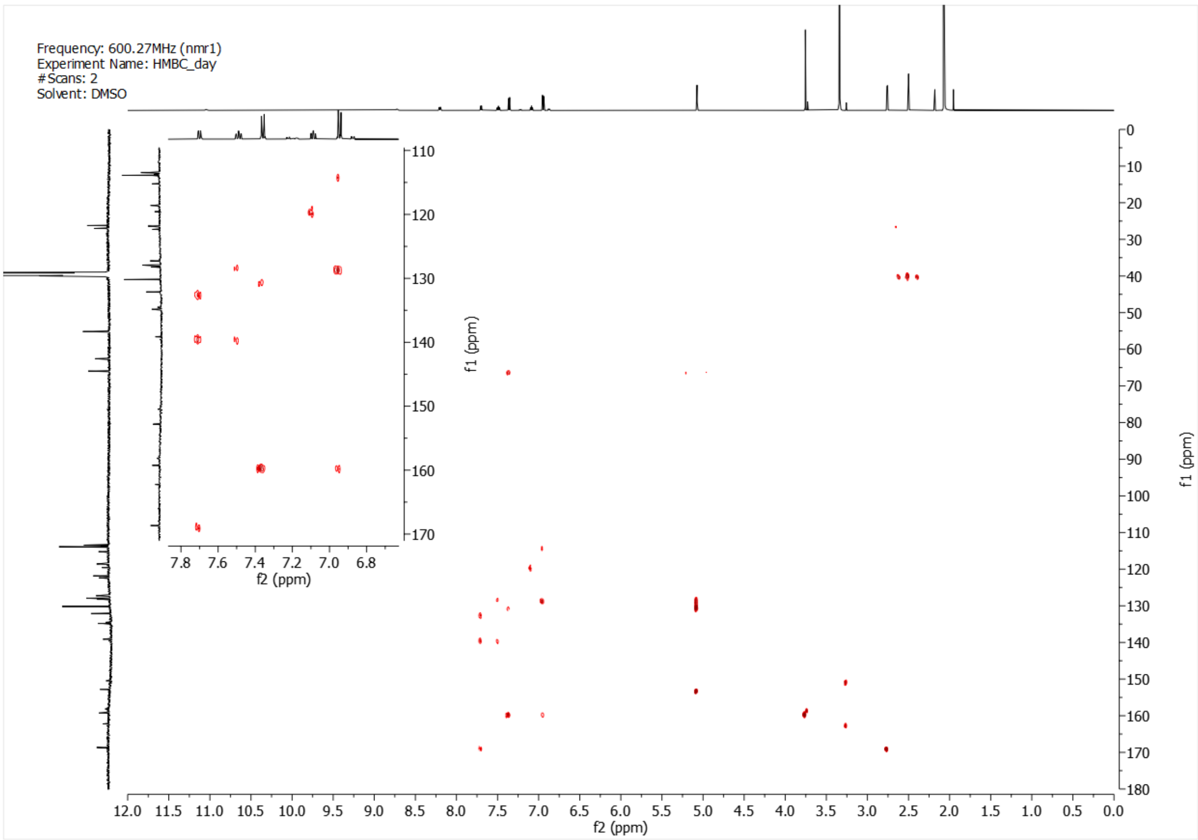
Frequency: 600.27MHz (nmr1)
Experiment Name: HMBC_day
#Scans: 2
Solvent: DMSO
f1 (ppm)
f2 (ppm)

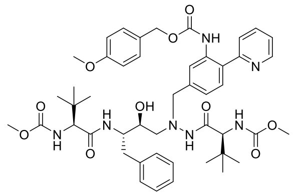

Methyl ((5S,10S,11S,14S)-11-benzyl-5-(tert-butyl)-10-hydroxy-8-(3-((((4-methoxybenzyl)oxy)carbonyl)amino)-4-(pyridin-2-yl)benzyl)-15,15-dimethyl-3,6,13-trioxo-2-oxa-4,7,8,12-tetraazahexadecan-14-yl)carbamate (**3a**)

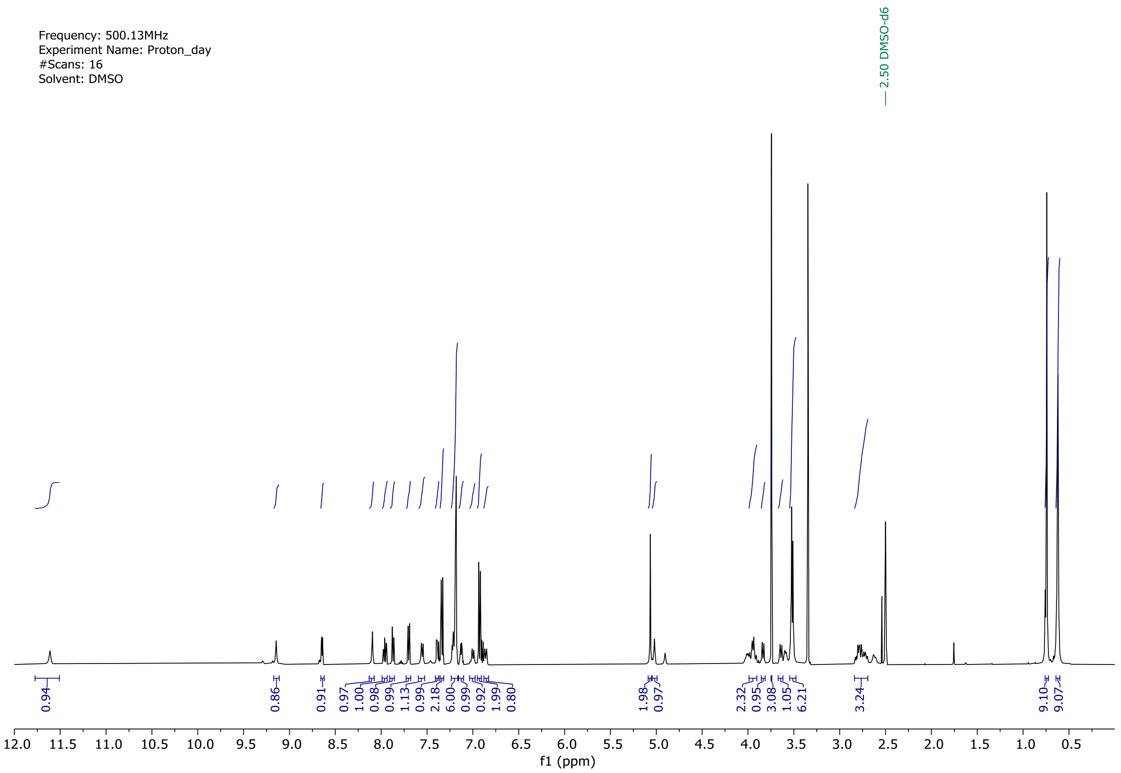

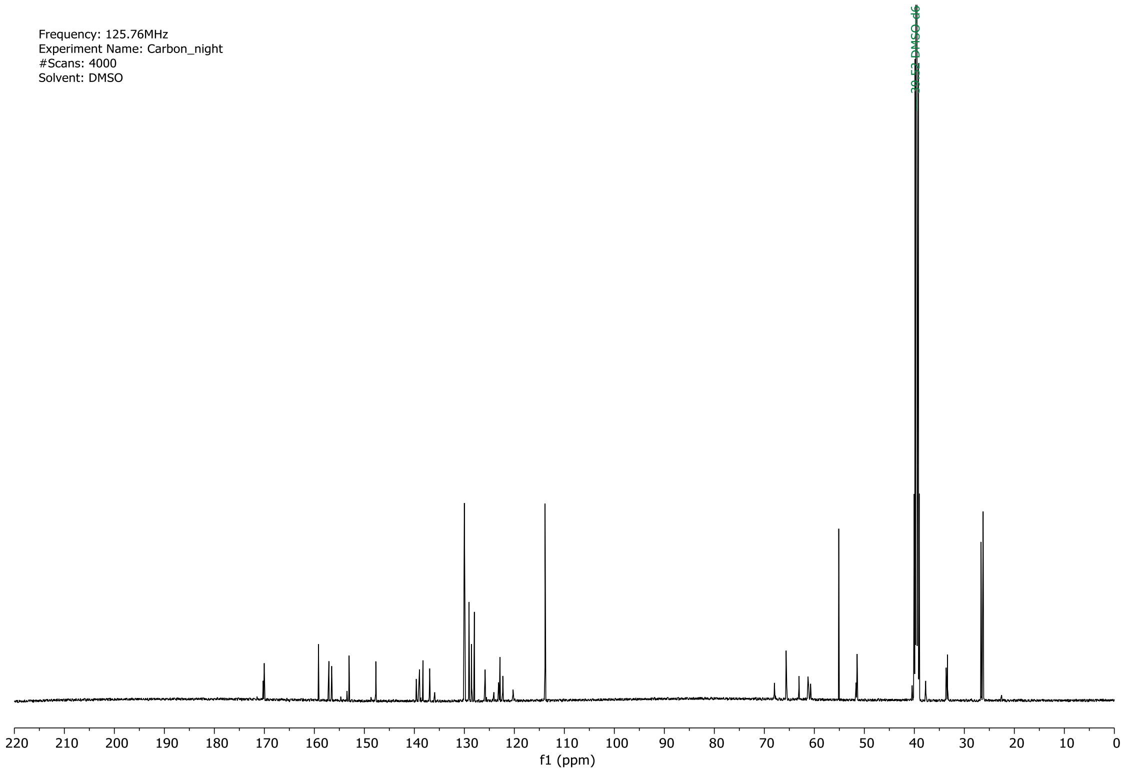

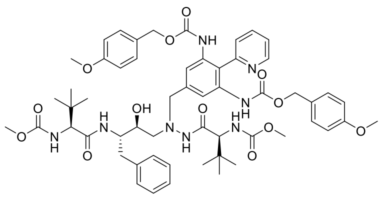

Bis(4-methoxybenzyl) (5-((5S,8S,9S)-8-benzyl-5-(tert-butyl)-9-hydroxy-11-((S)-2-((methoxycarbonyl)amino)-3,3-dimethylbutanamido)-3,6-dioxo-2-oxa-4,7,11-triazadodecan-12-yl)-2-(pyridin-2-yl)-1,3-phenylene)dicarbamate (**3a´**)

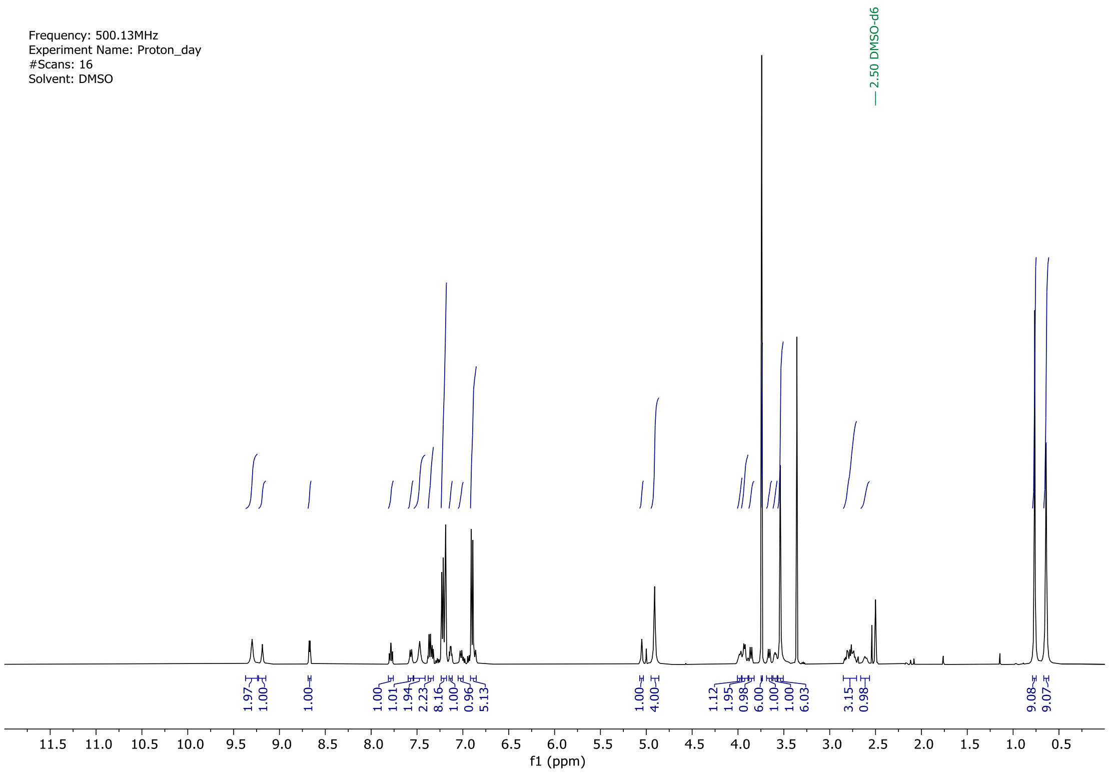

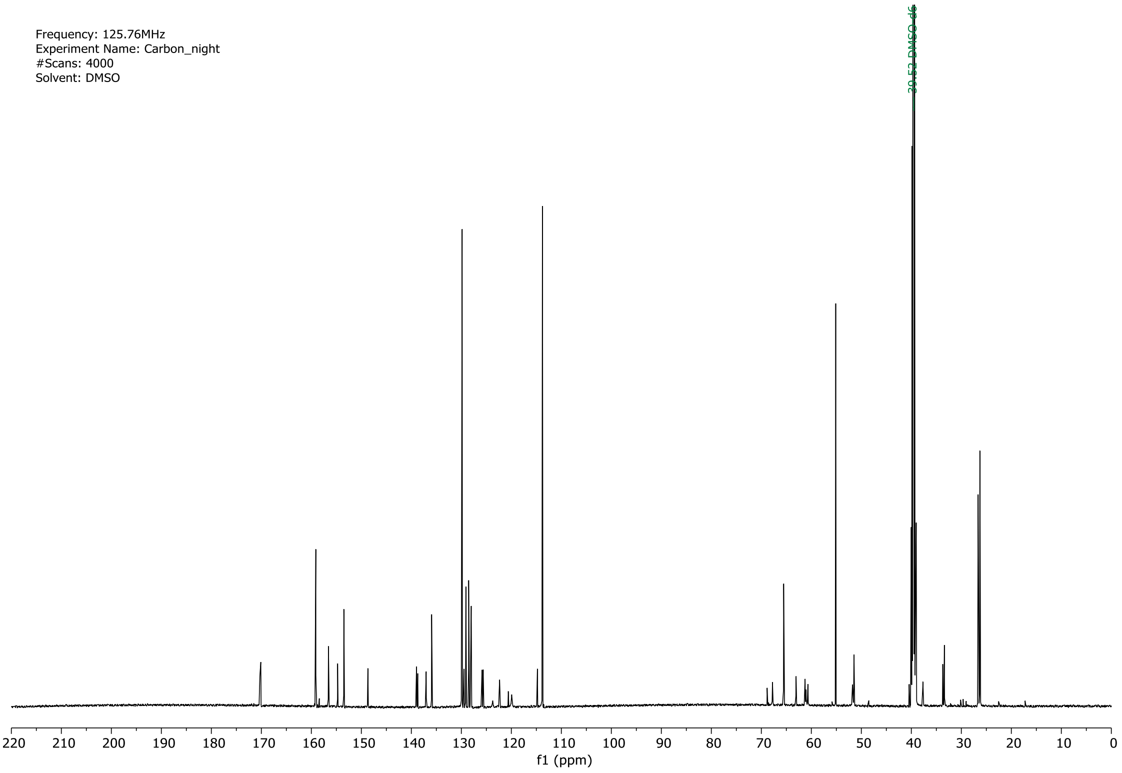

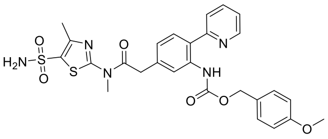

4-methoxybenzyl (5-(2-(methyl(4-methyl-5-sulfamoylthiazol-2-yl)amino)-2-oxoethyl)-2-(pyridin-2-yl)phenyl)carbamate (**3b**)

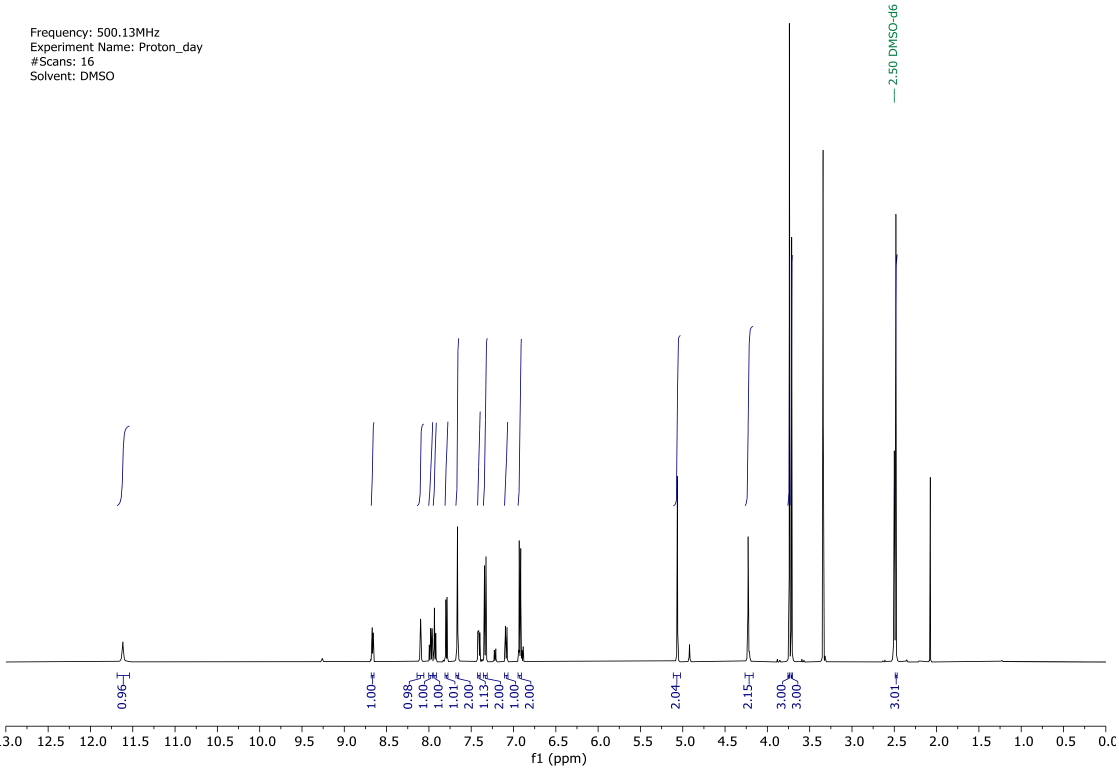

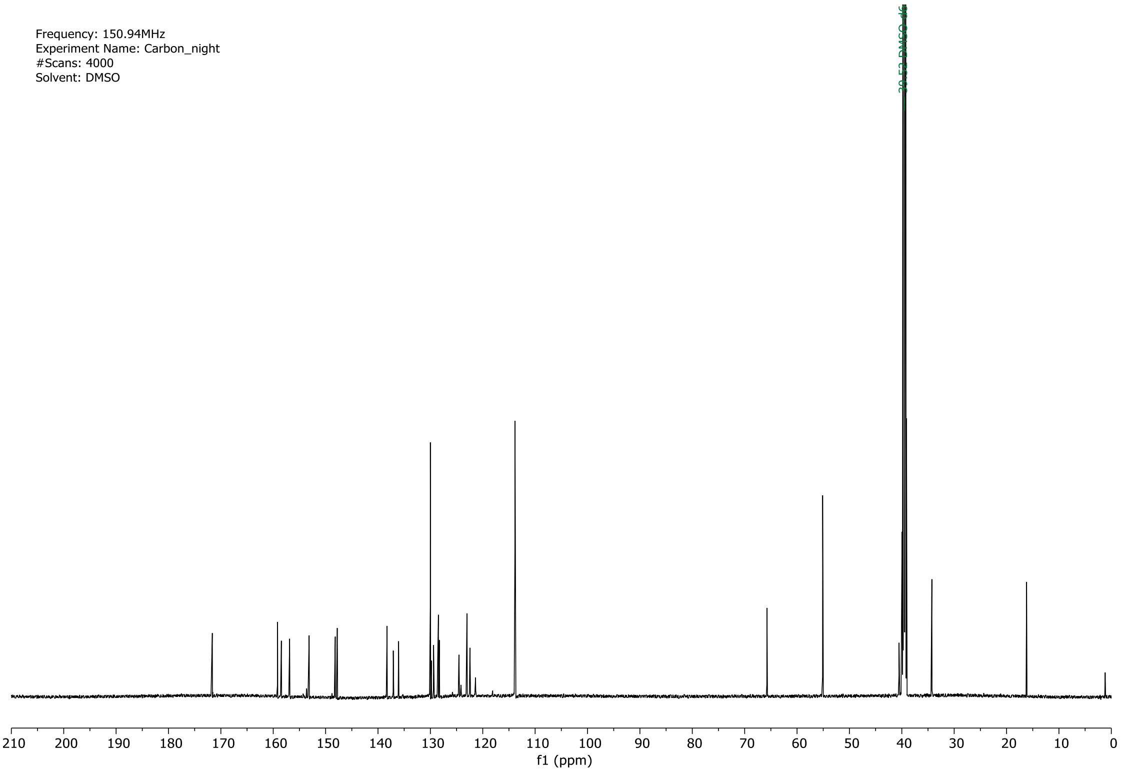

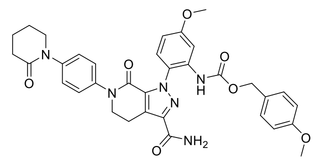

4-methoxybenzyl (2-(3-carbamoyl-7-oxo-6-(4-(2-oxopiperidin-1-yl)phenyl)-4,5,6,7-tetrahydro-1H-pyrazolo[3,4-c]pyridin-1-yl)-5-methoxyphenyl)carbamate (**3c**)

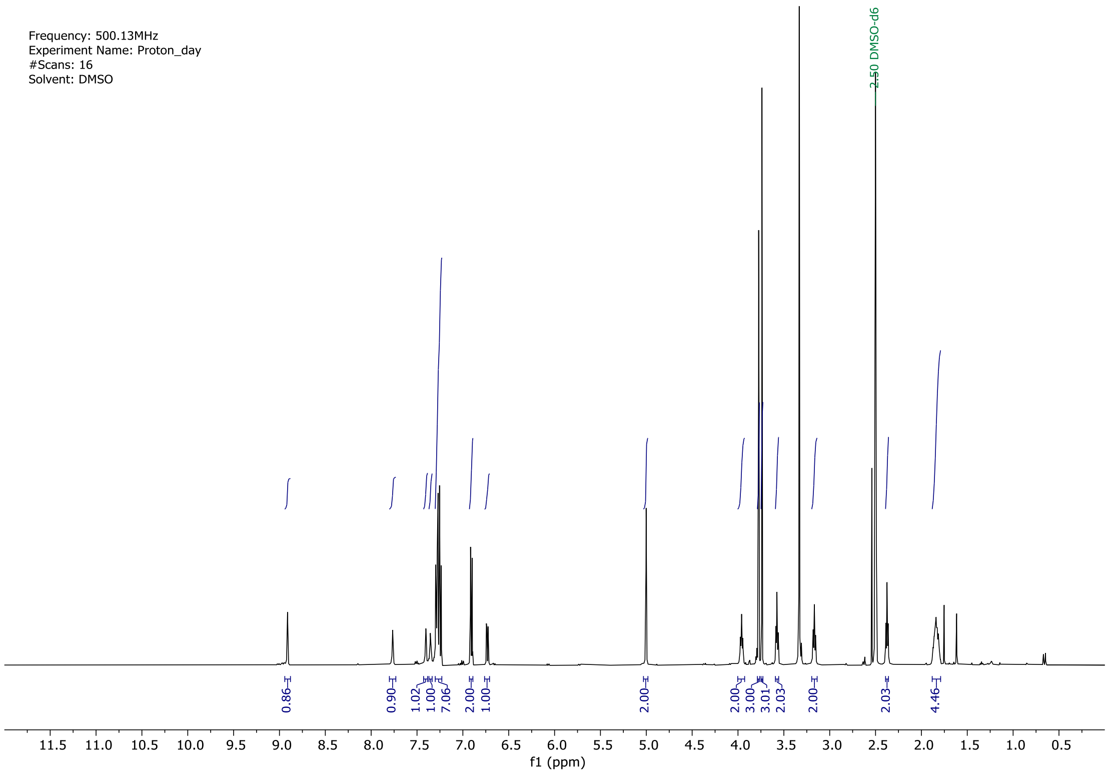

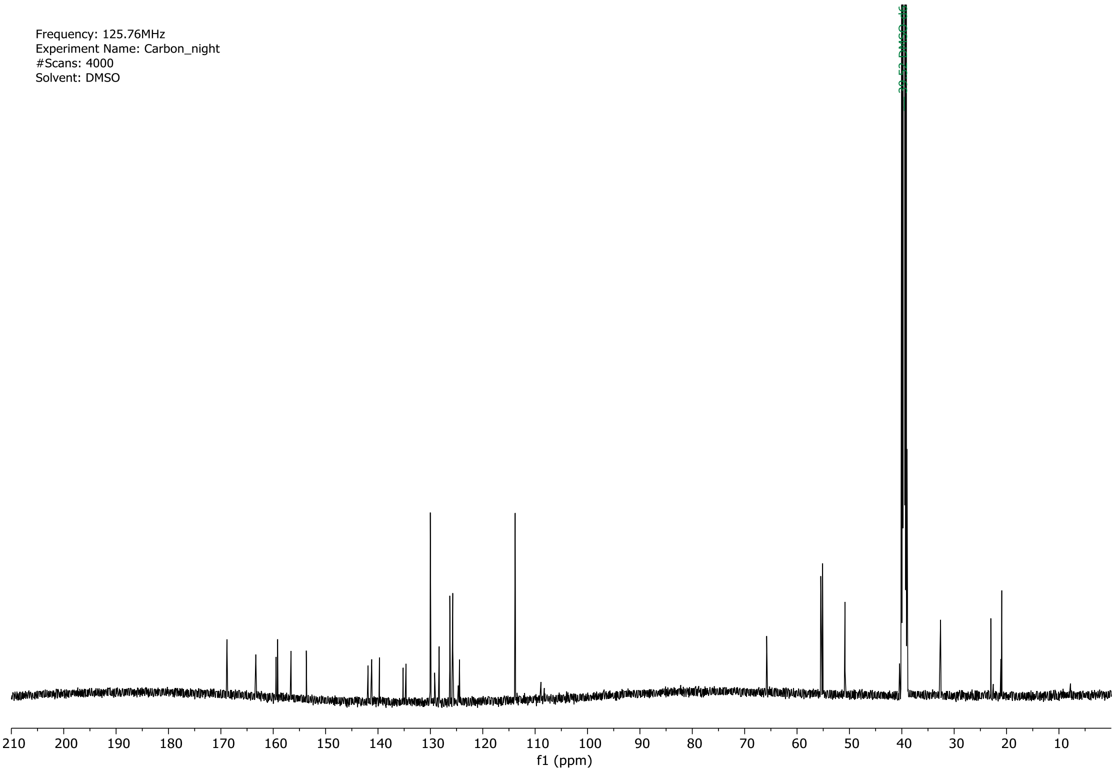

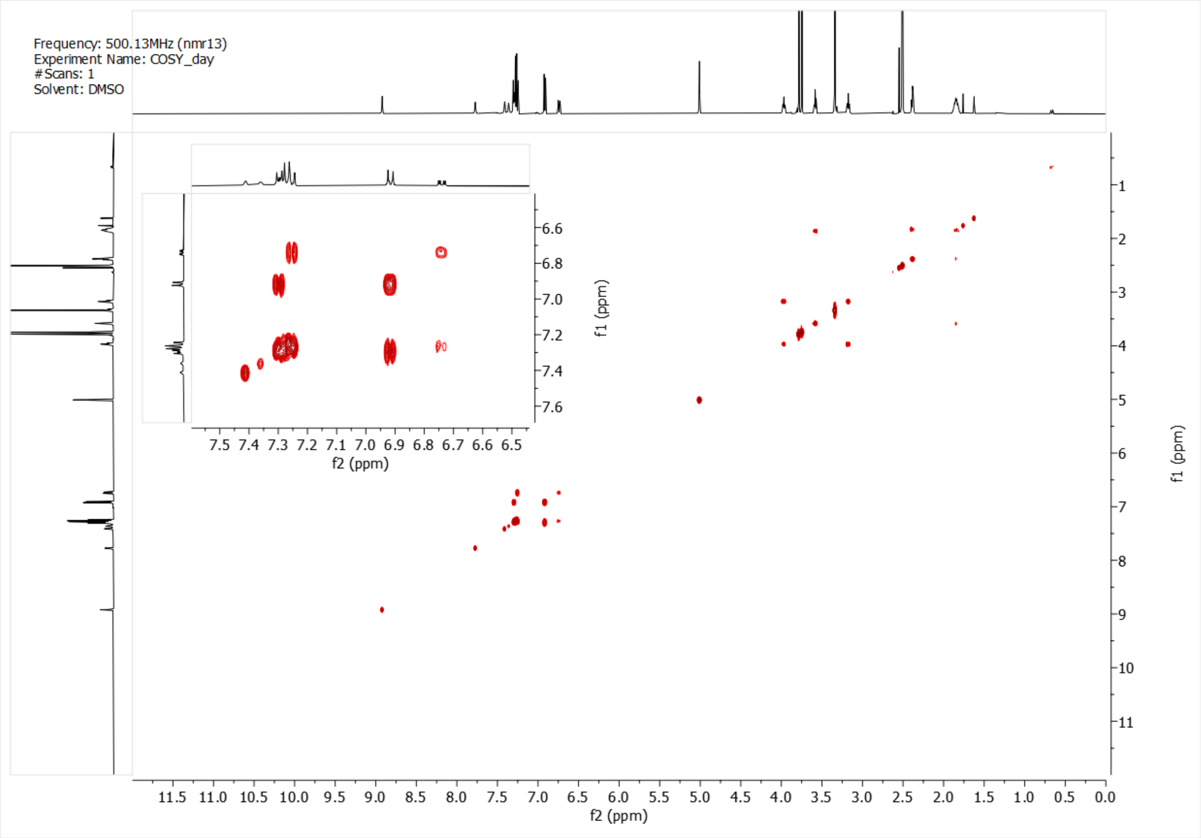
Frequency: 500.13MHz (nmr13)
Experiment Name: COSY_day
#Scans: 1
Solvent: DMSO
f2 (ppm)
f1 (ppm)

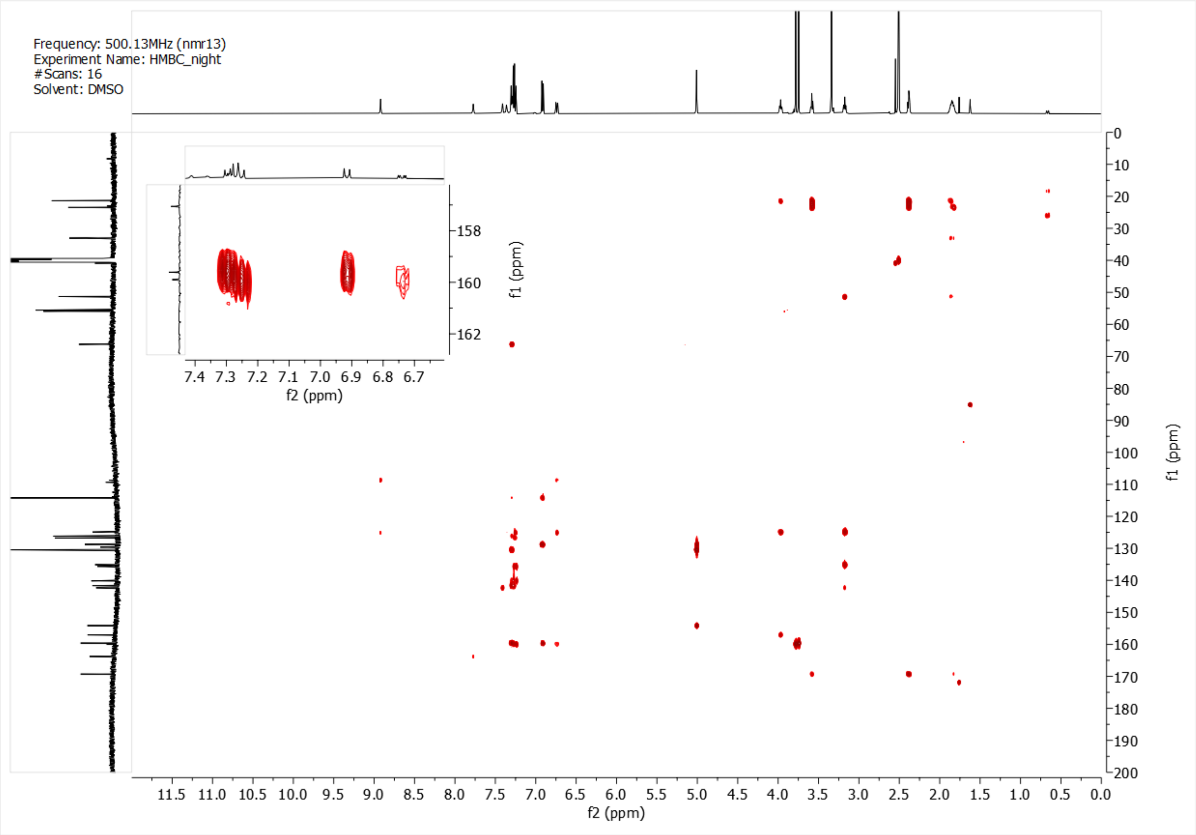
Frequency: 500.13MHz (nmr13)
Experiment Name: HMBC_night
#Scans: 16
Solvent: DMSO
f2 (ppm)
f1 (ppm)

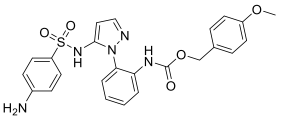

4-methoxybenzyl (2-(5-((4-aminophenyl)sulfonamido)-1H-pyrazol-1-yl)phenyl)carbamate (**3d**)

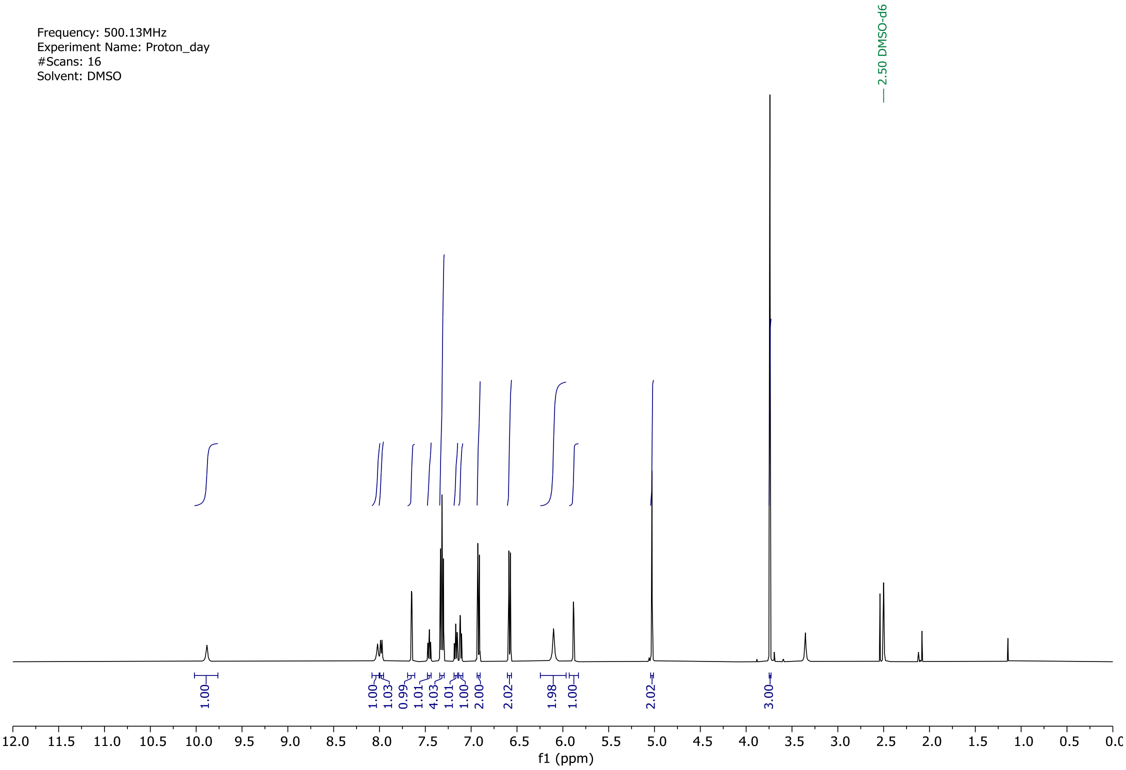

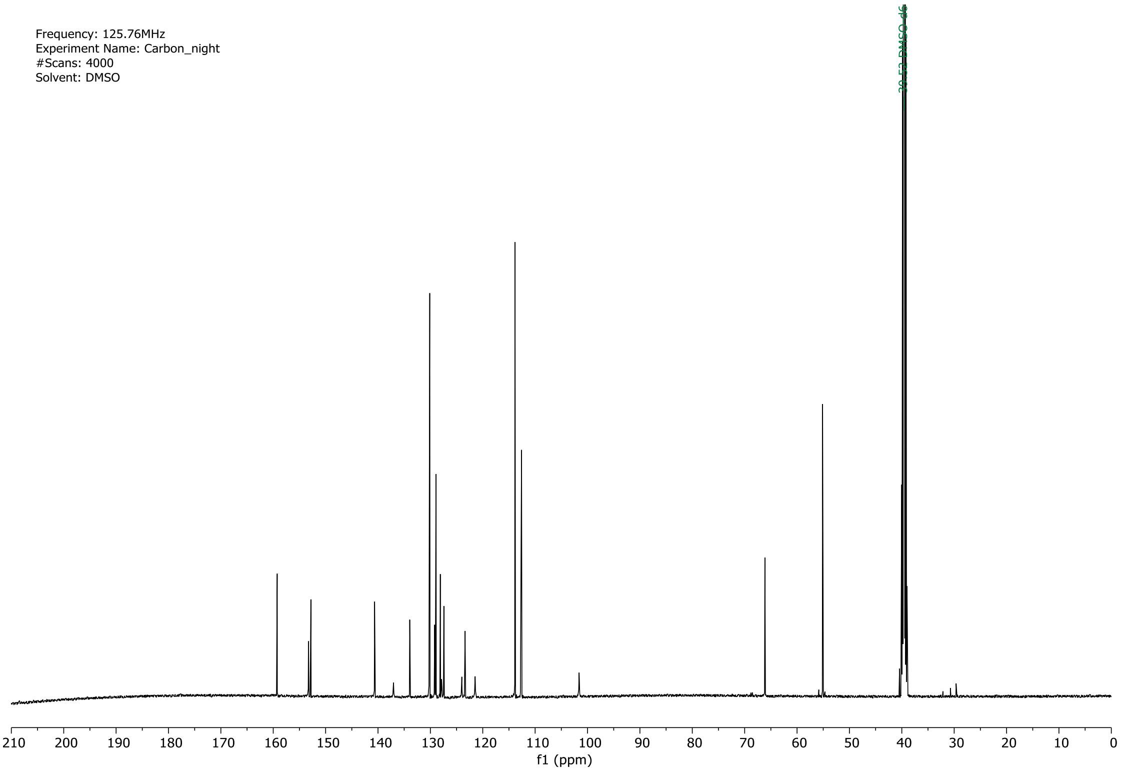

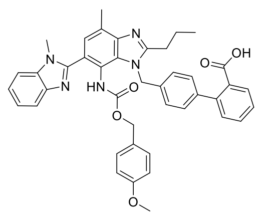

4'-((4'-((((4-methoxybenzyl)oxy)carbonyl)amino)-1,7'-dimethyl-2'-propyl-1H,3'H-[2,5'-bibenzo[d]imidazol]-3'-yl)methyl)-[1,1'-biphenyl]-2-carboxylic acid (**3e**)

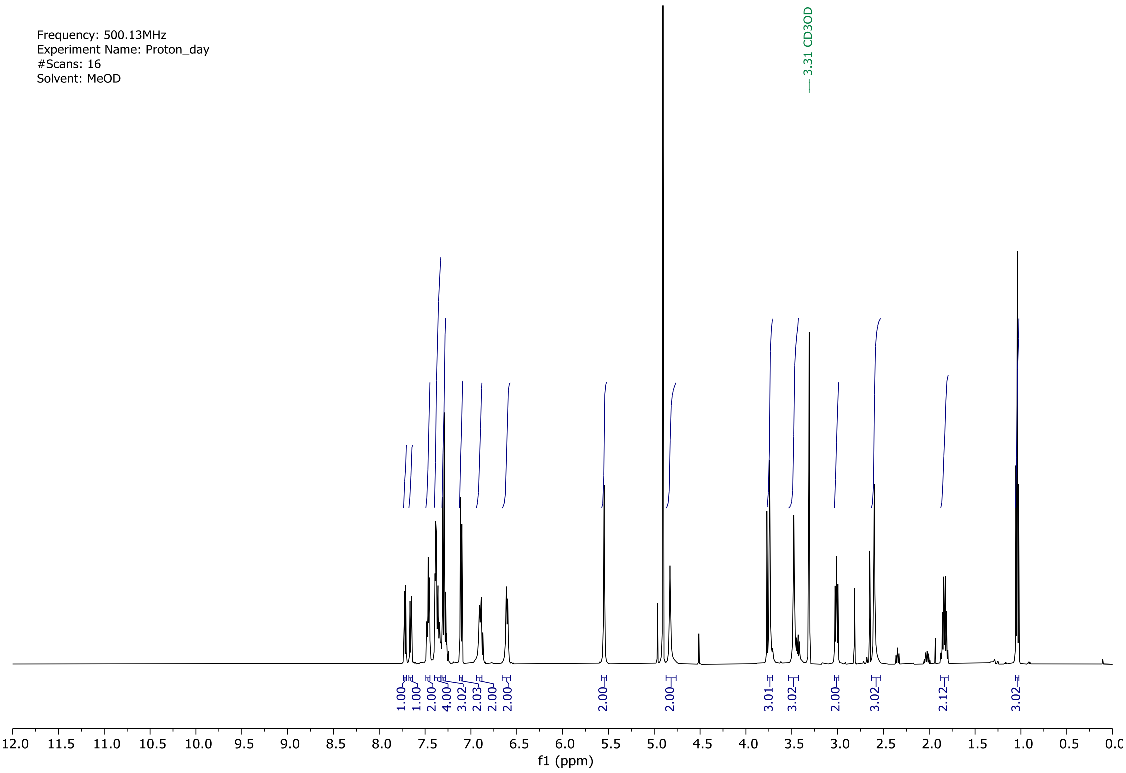

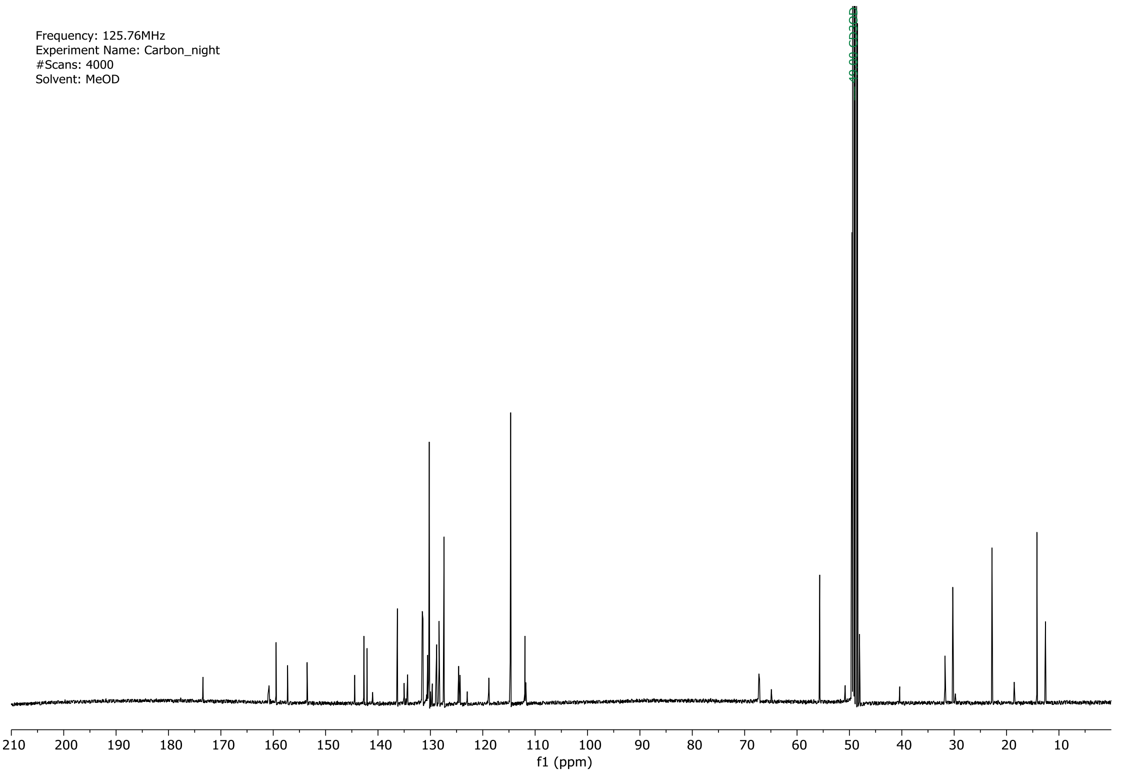

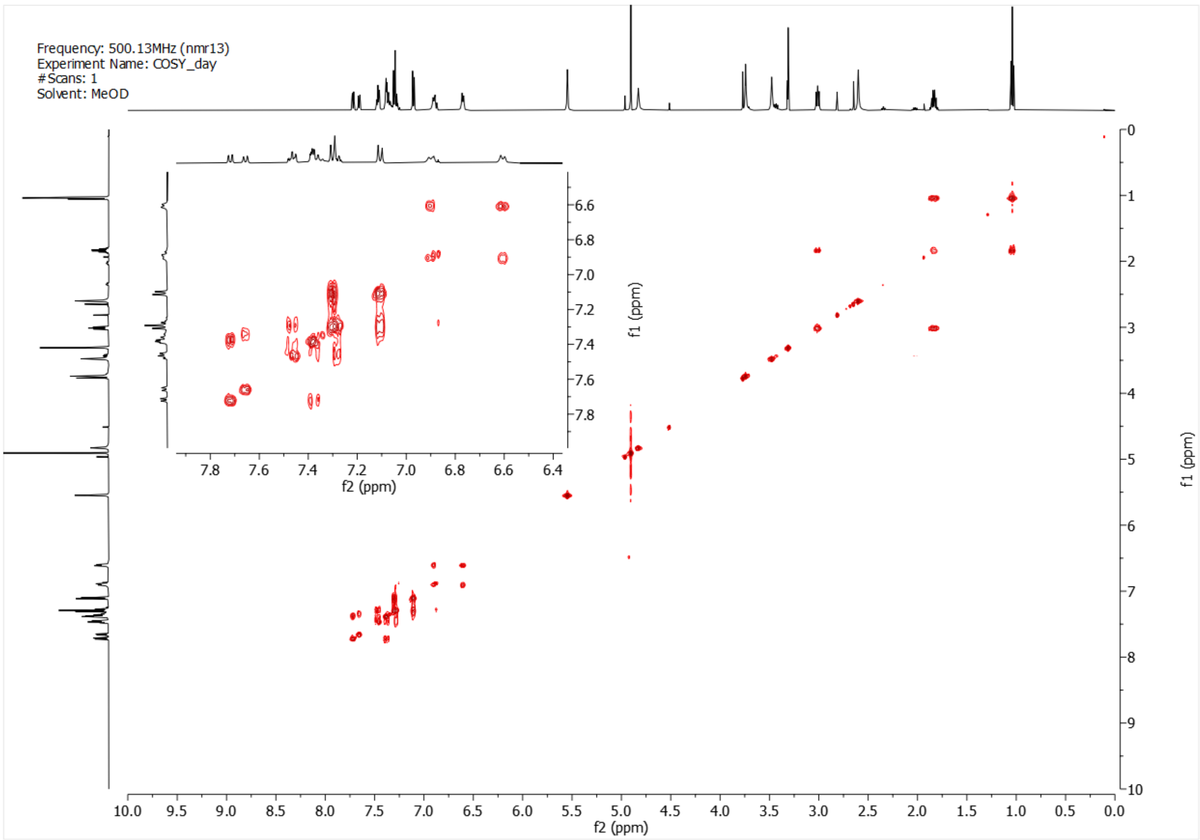
Frequency: 500.13MHz (nmr13)
Experiment Name: COSY_day
#Scans: 1
Solvent: MeOD
f2 (ppm)
f1 (ppm)

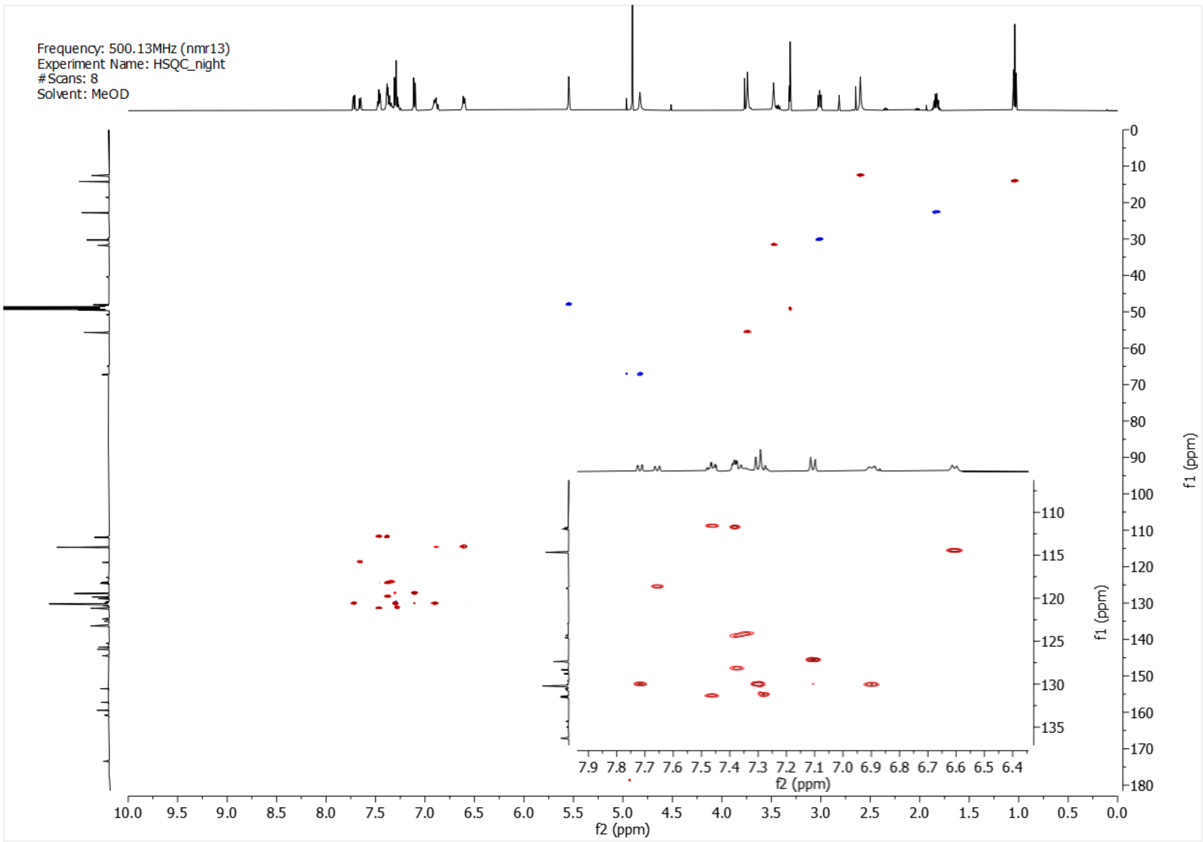
Frequency: 500.13MHz (nmr13)
Experiment Name: HSQC_night
#Scans: 8
Solvent: MeOD
f2 (ppm)
f1 (ppm)

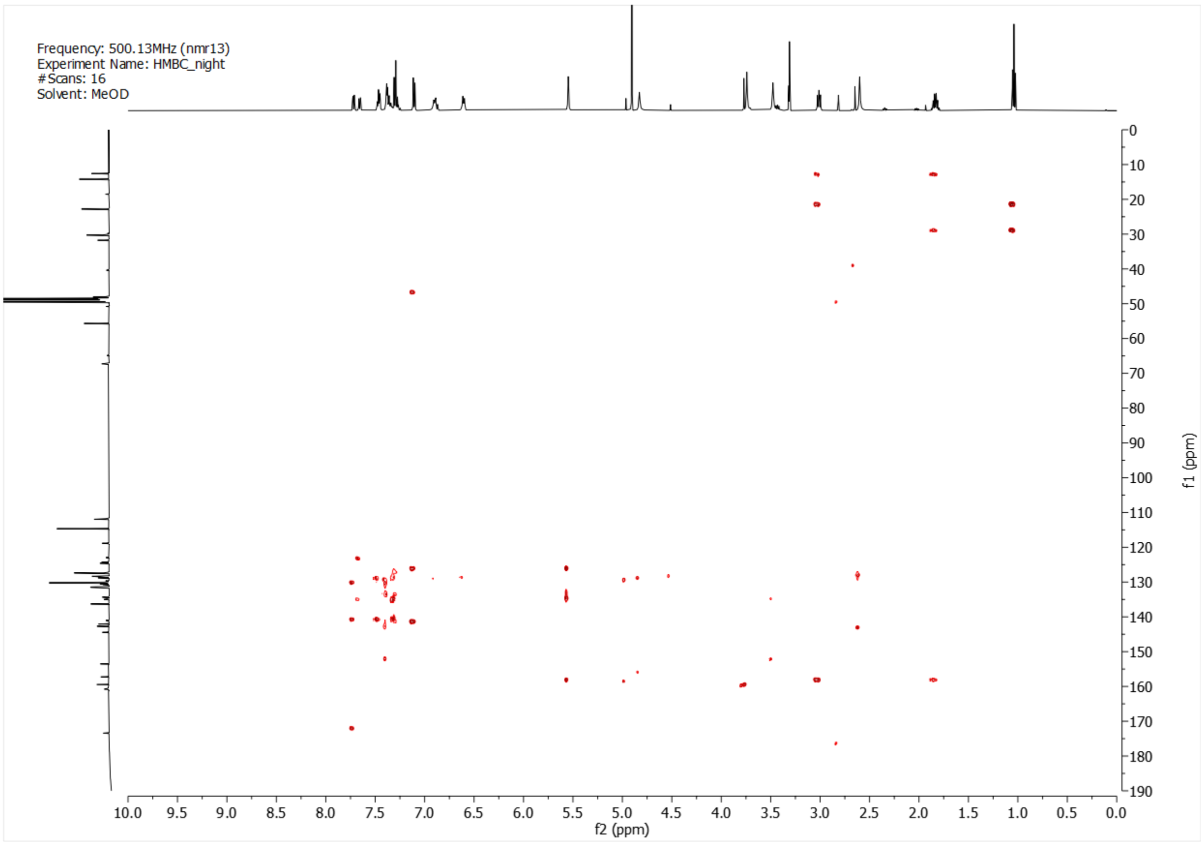

Frequency: 500.13MHz (nmr13)
Experiment Name: HMBC_night
#Scans: 16
Solvent: MeOD
f2 (ppm)
f1 (ppm)

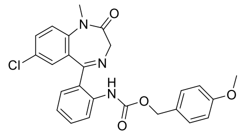

4-methoxybenzyl (2-(7-chloro-1-methyl-2-oxo-2,3-dihydro-1H-benzo[e][1,4]diazepin-5-yl)phenyl)carbamate (**3f**)

Frequency: 500.13MHz
Experiment Name: Proton_day
#Scans: 16
Solvent: DMSO

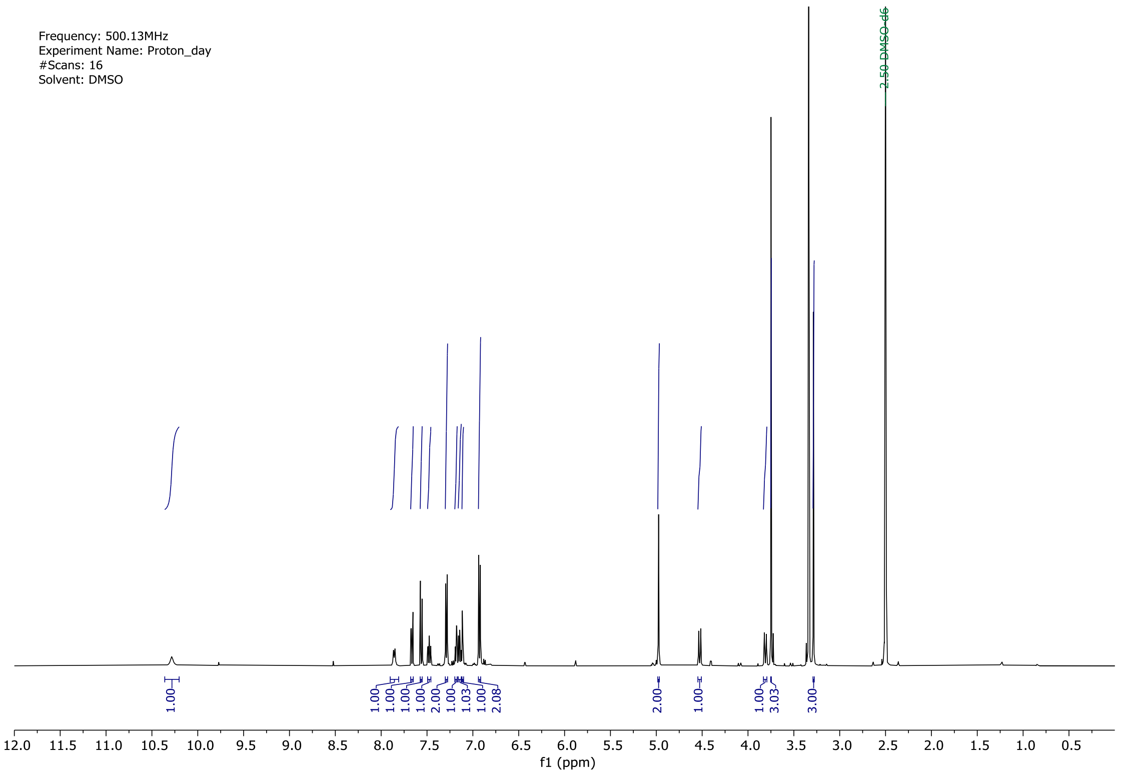

Frequency: 150.94MHz
Experiment Name: Carbon_night
#Scans: 4000
Solvent: DMSO

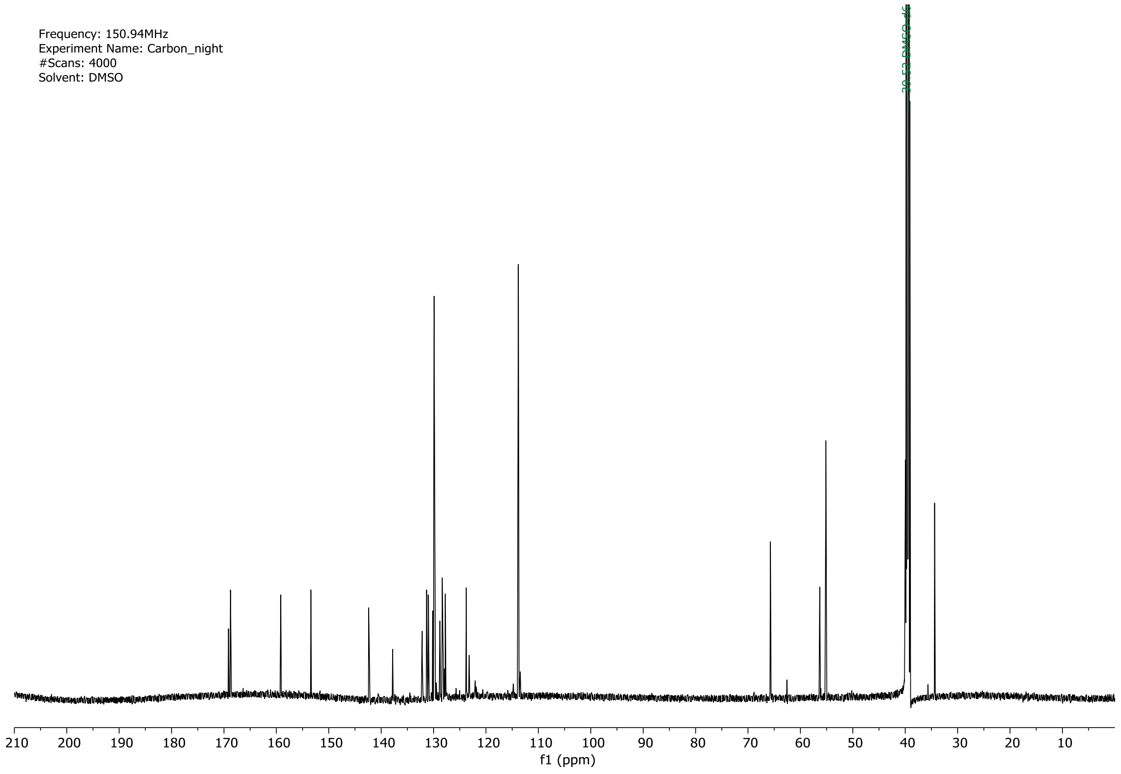

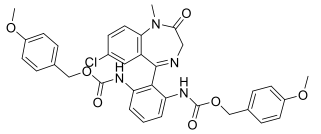

bis(4-methoxybenzyl) (2-(7-chloro-1-methyl-2-oxo-2,3-dihydro-1H-benzo[e][1,4]diazepin-5-yl)-1,3-phenylene)dicarbamate (**3f′**)

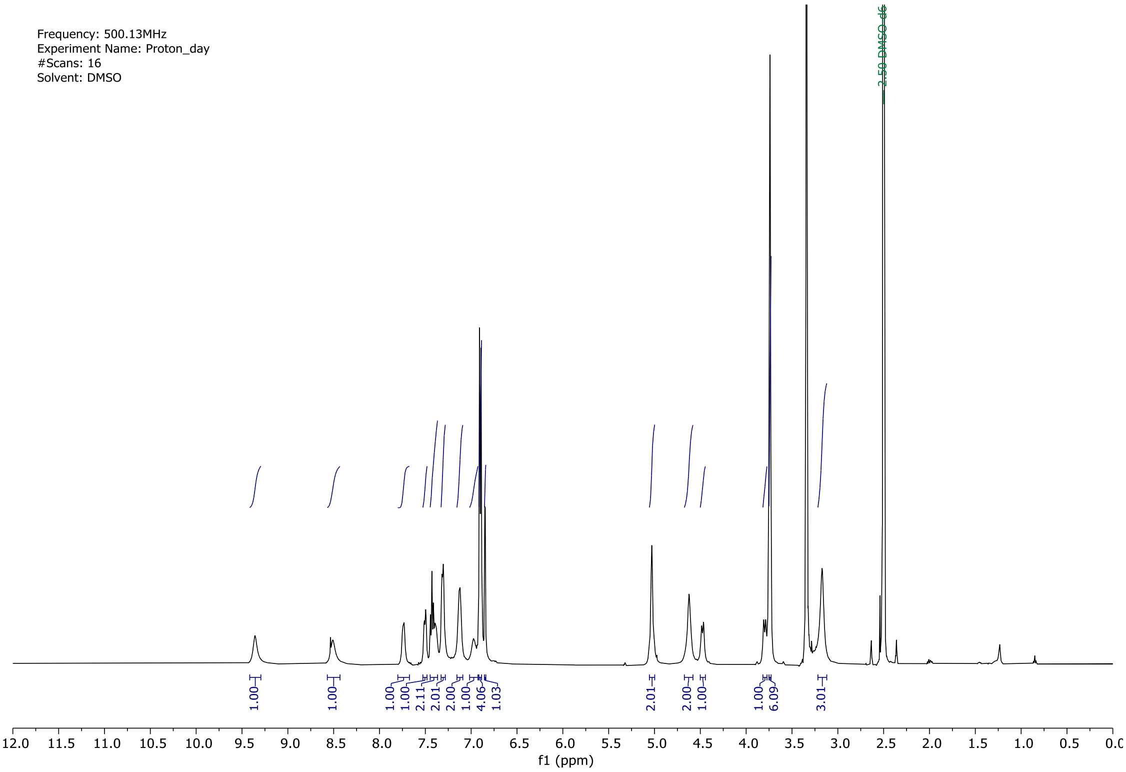

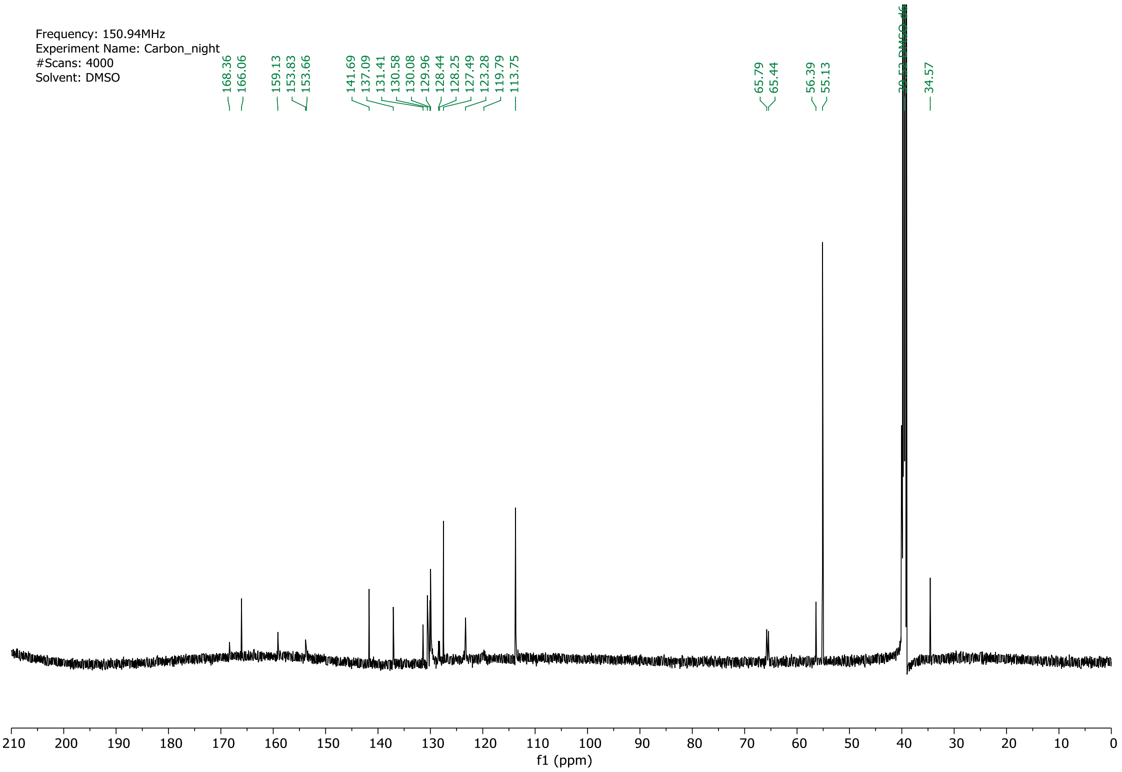

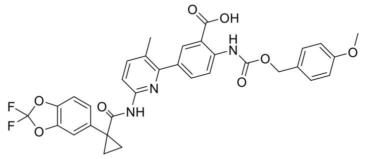

5-(6-(1-(2,2-difluorobenzo[d][1,3]dioxol-5-yl)cyclopropane-1-carboxamido)-3-methylpyridin-2-yl)-2-((((4-methoxybenzyl)oxy)carbonyl)amino)benzoic acid (**3g**)

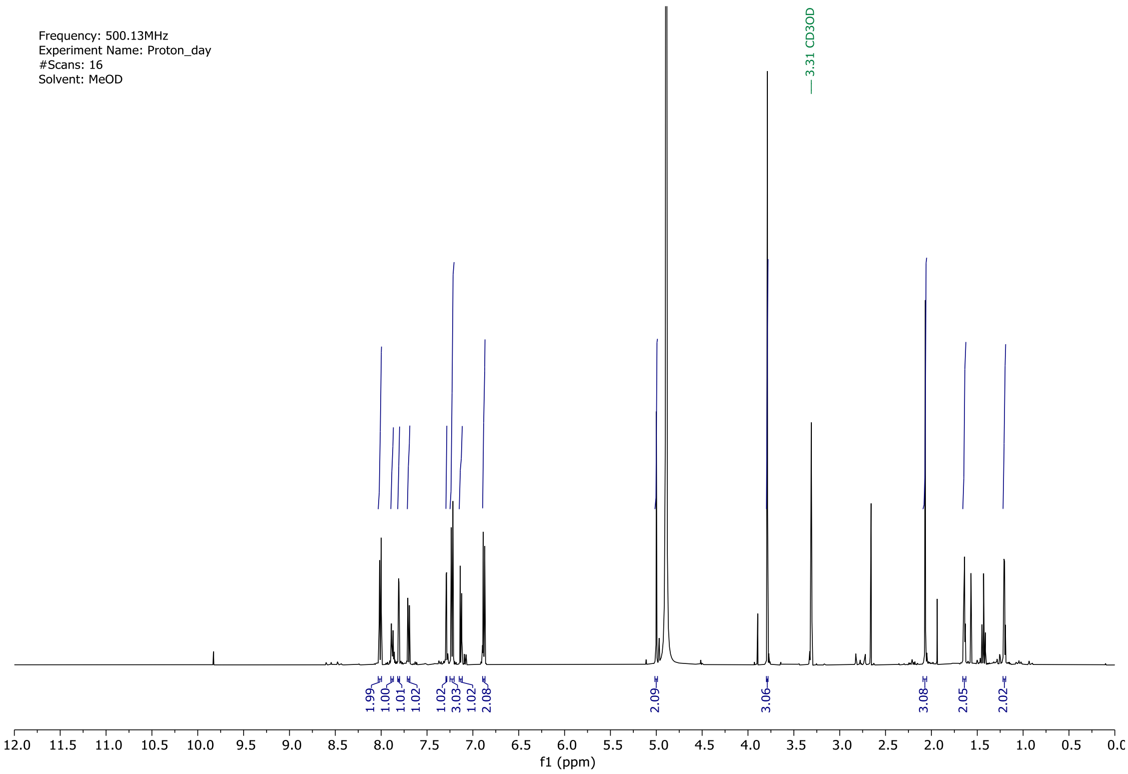

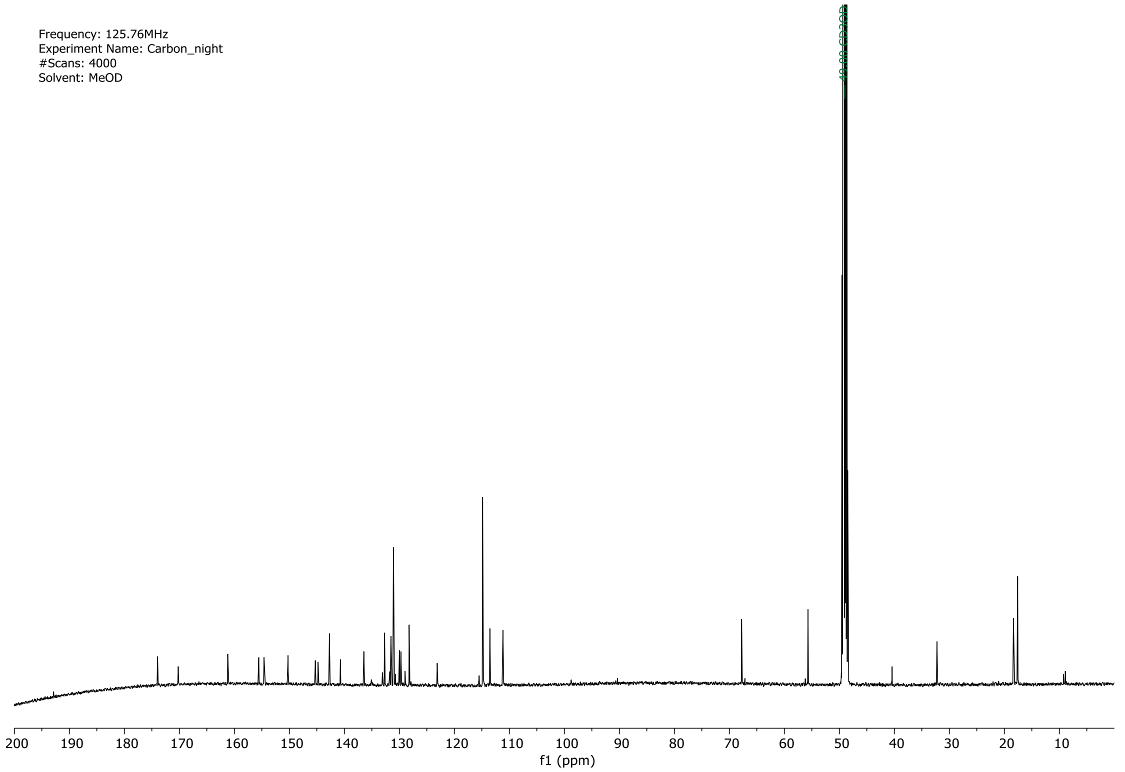

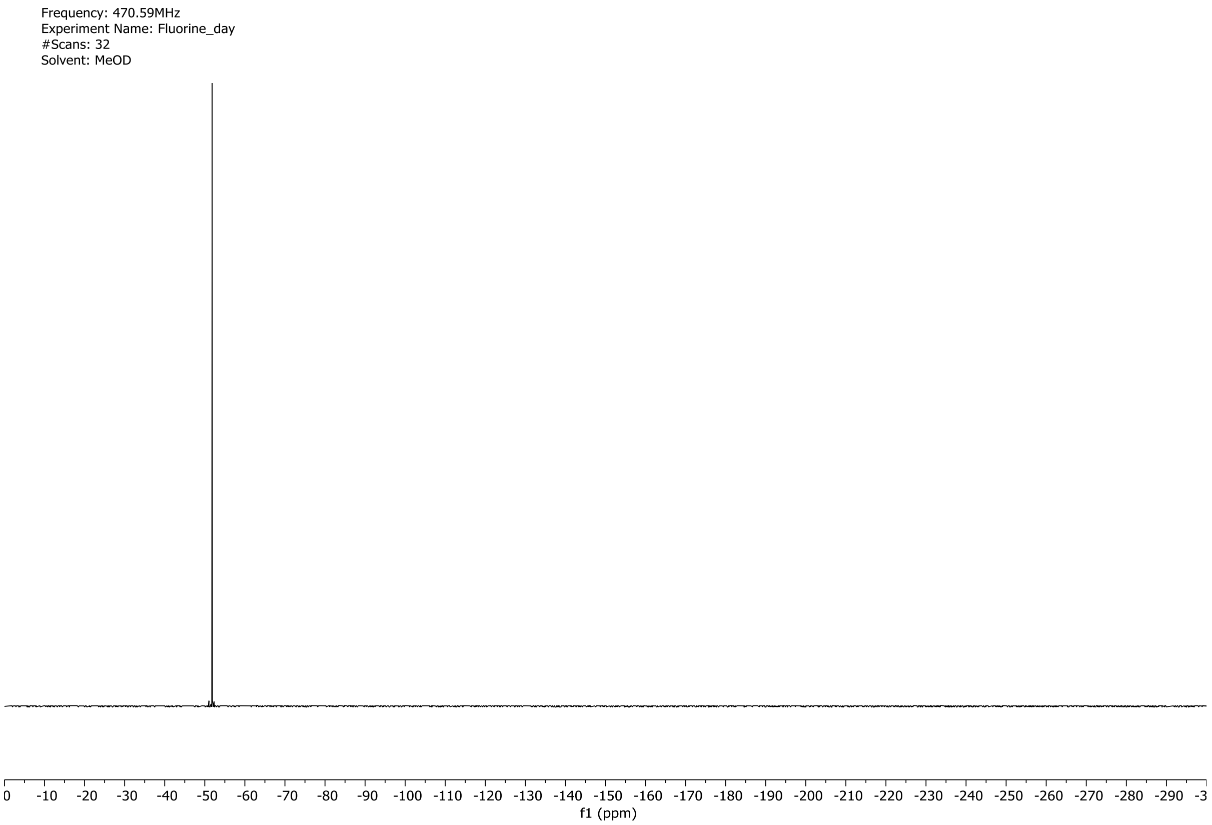
Frequency: 470.59MHz
Experiment Name: Fluorine_day
#Scans: 32
Solvent: MeOD
f1 (ppm)

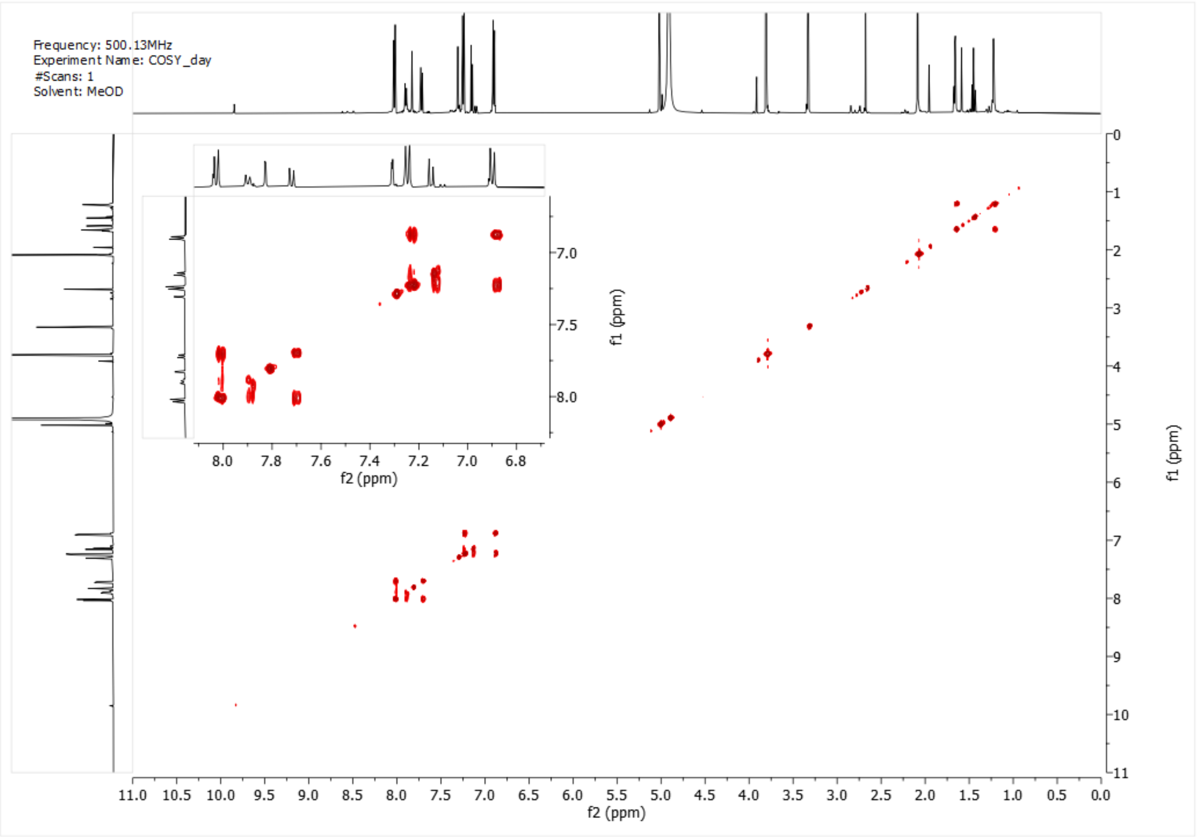
Frequency: 500.13MHz
Experiment Name: COSY_day
#Scans: 1
Solvent: MeOD
f2 (ppm)
f1 (ppm)

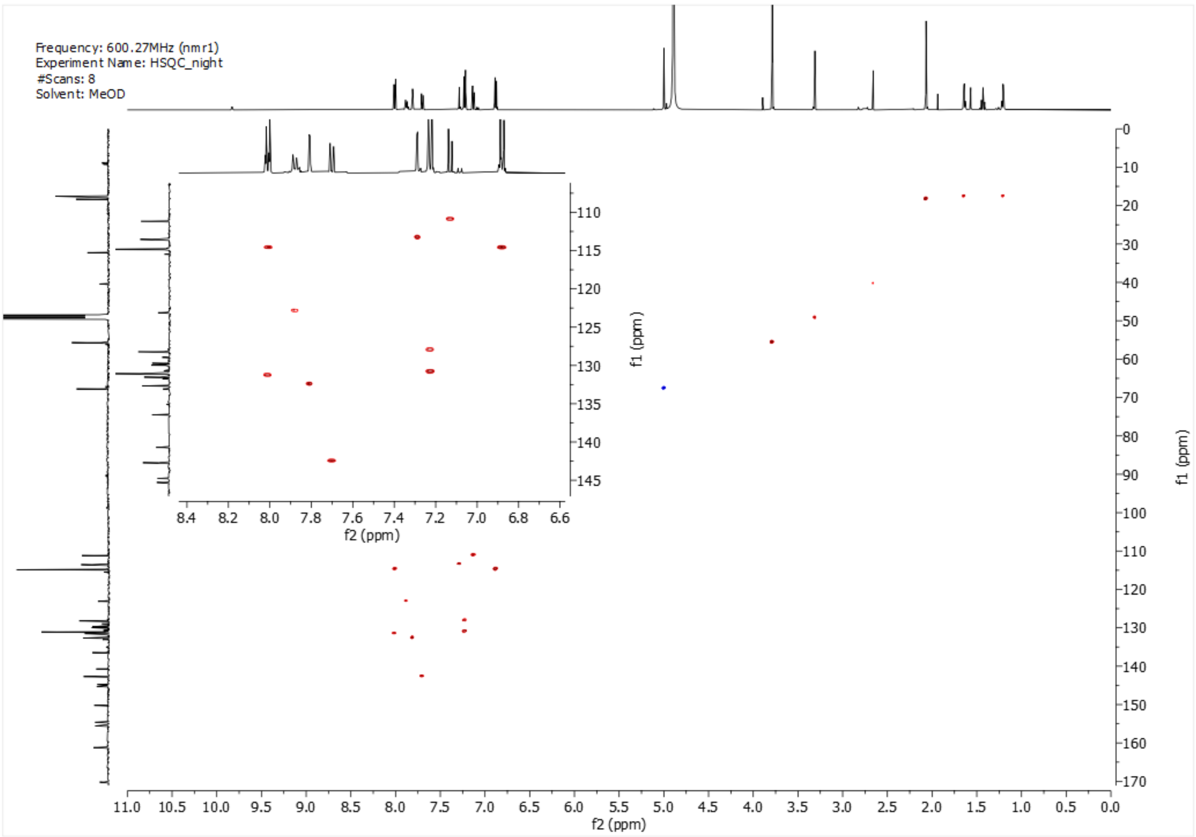
Frequency: 600.27MHz (nmr1)
Experiment Name: HSQC_night
#Scans: 8
Solvent: MeOD
f1 (ppm)
f2 (ppm)

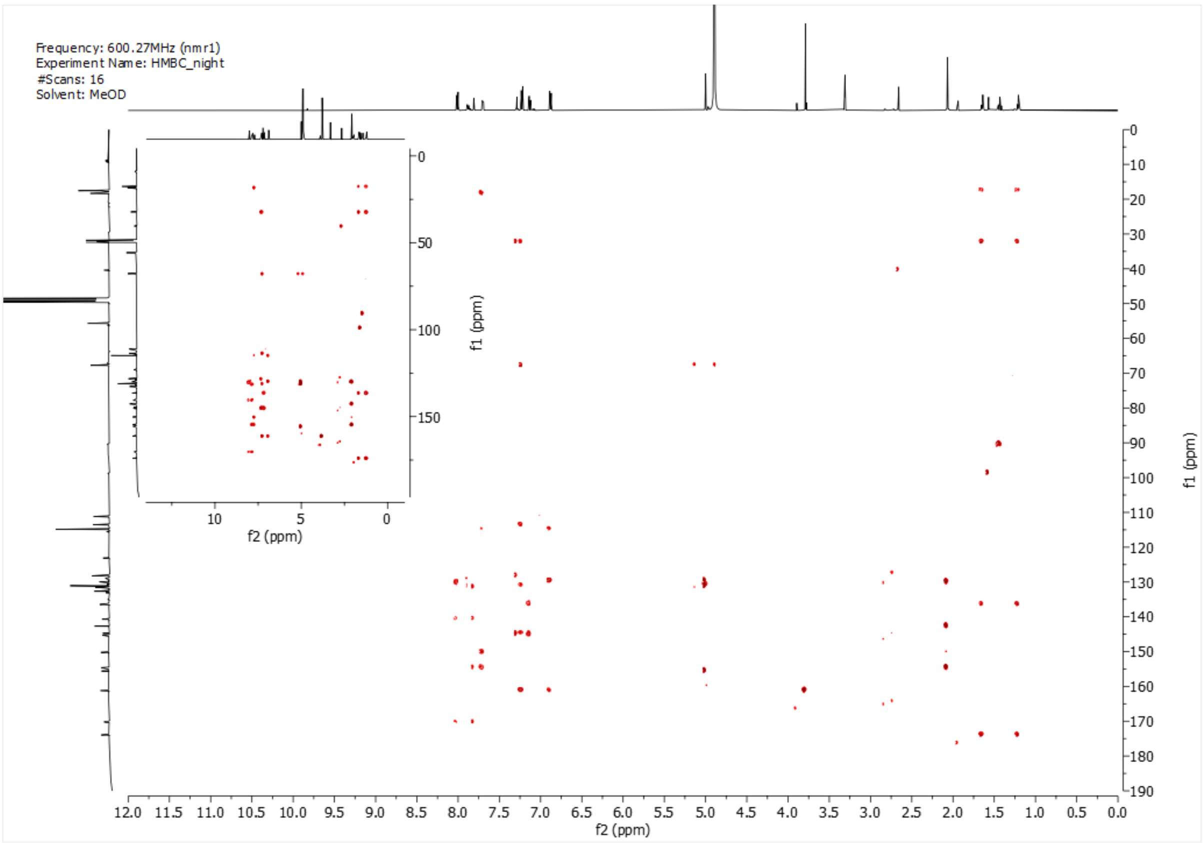
Frequency: 600.27MHz (nmr1)
Experiment Name: HMBC_night
#Scans: 16
Solvent: MeOD
f1 (ppm)
f2 (ppm)

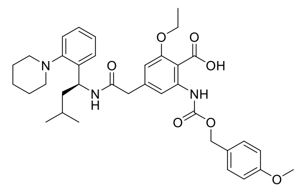

(S)-2-ethoxy-6-((((4-methoxybenzyl)oxy)carbonyl)amino)-4-(2-((3-methyl-1-(2-(piperidin-1-yl)phenyl)butyl)amino)-2-oxoethyl)benzoic acid (**3h**)

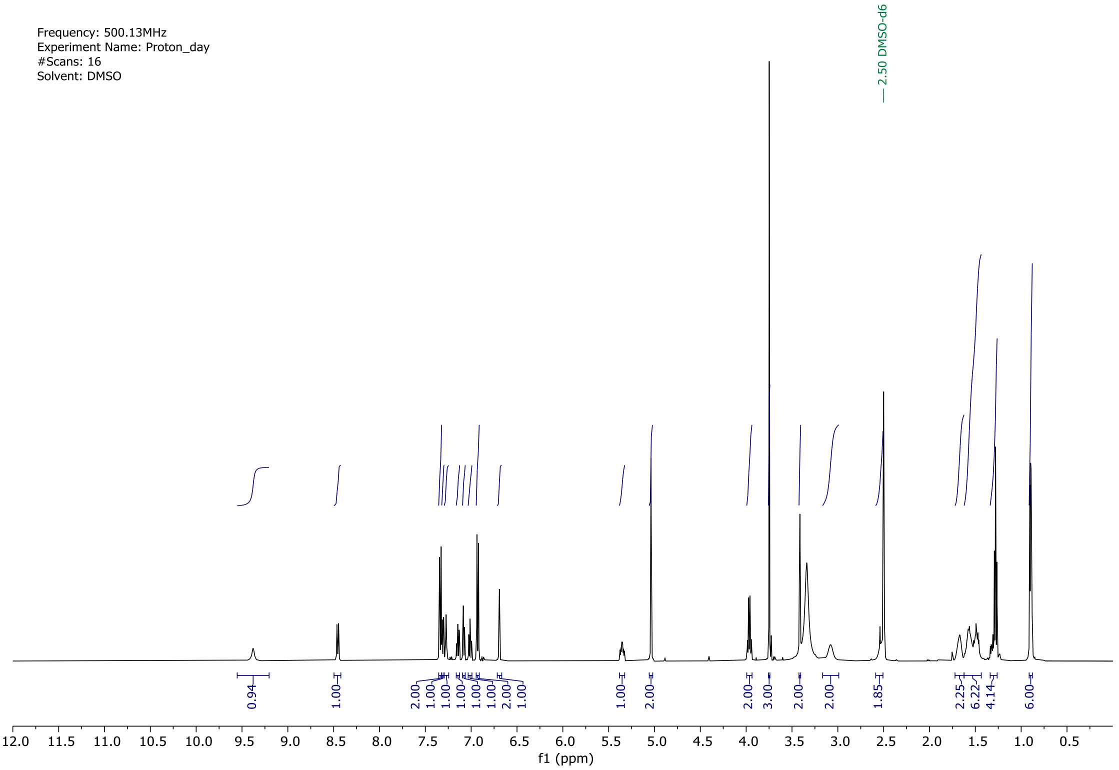

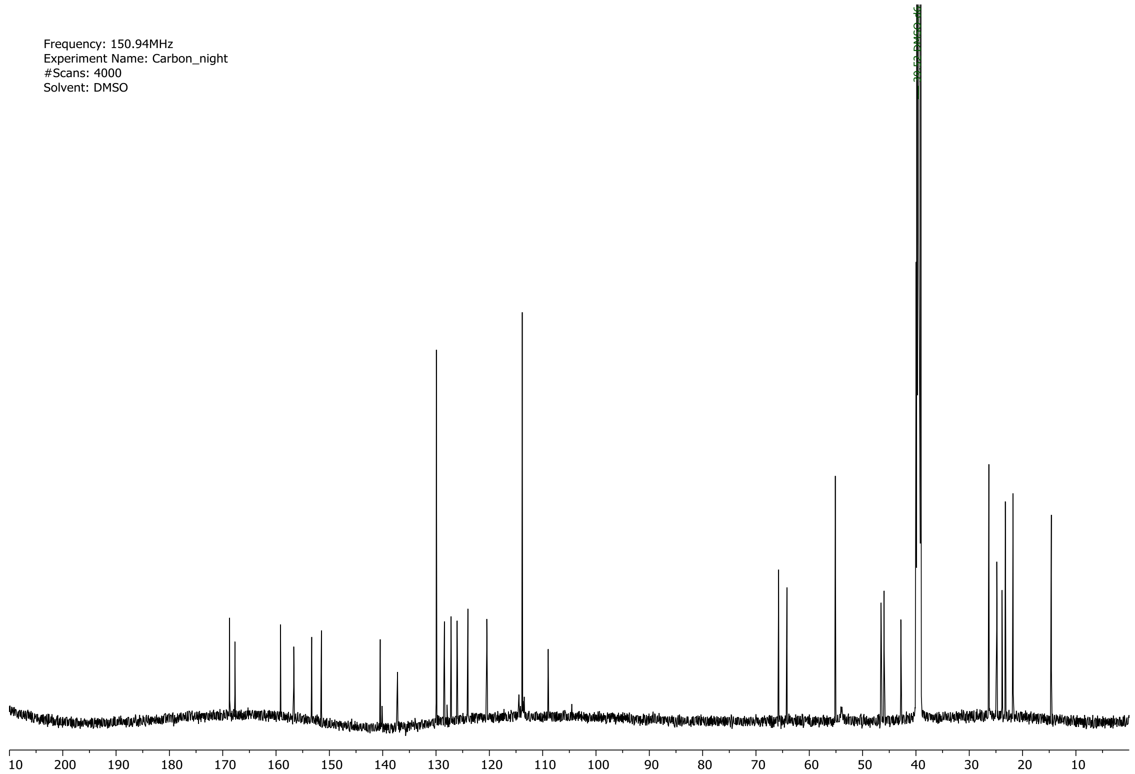

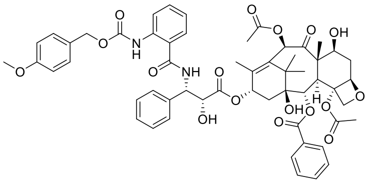

(2aR,4S,4aS,6R,9S,11S,12S,12aR,12bS)-12-(benzoyloxy)-4,11-dihydroxy-9-(((2R,3S)-2-hydroxy-3-(2-((((4-methoxybenzyl)oxy)carbonyl)amino)benzamido)-3-phenylpropanoyl)oxy)-4a,8,13,13-tetramethyl-5-oxo-3,4,4a,5,6,9,10,11,12,12a-decahydro-1H-7,11-methanocyclodeca[3,4]benzo[1,2-b]oxete-6,12b(2aH)-diyl diacetate (**3i**)

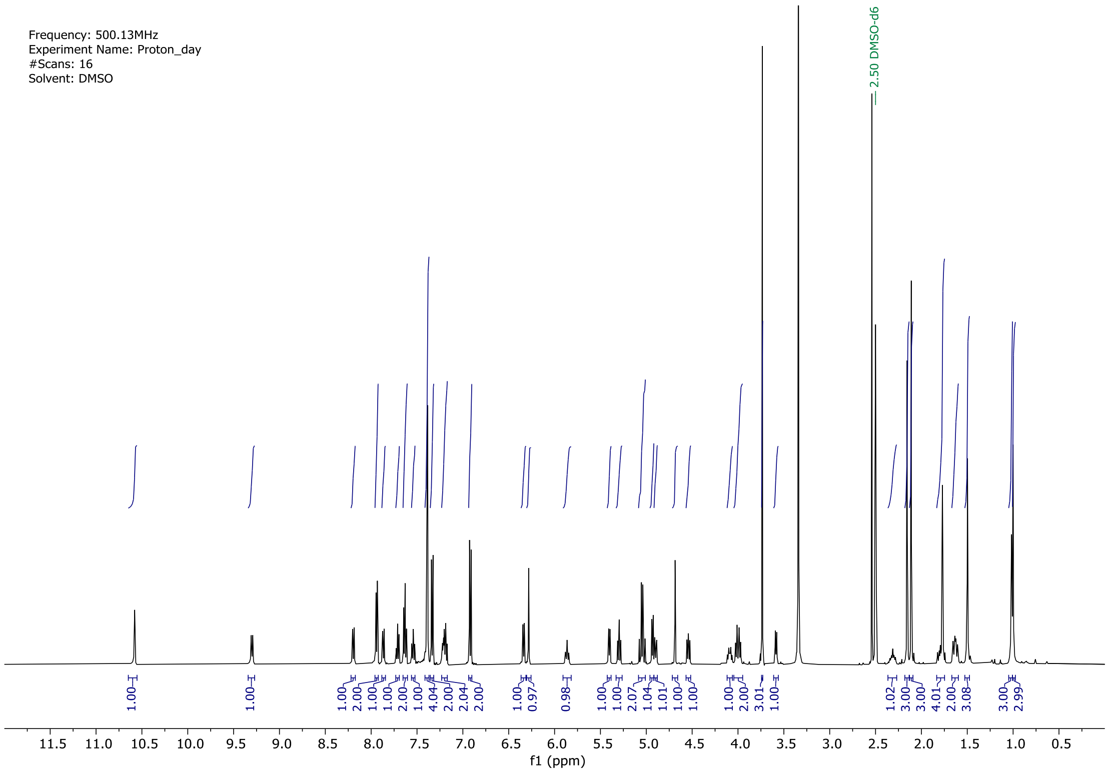

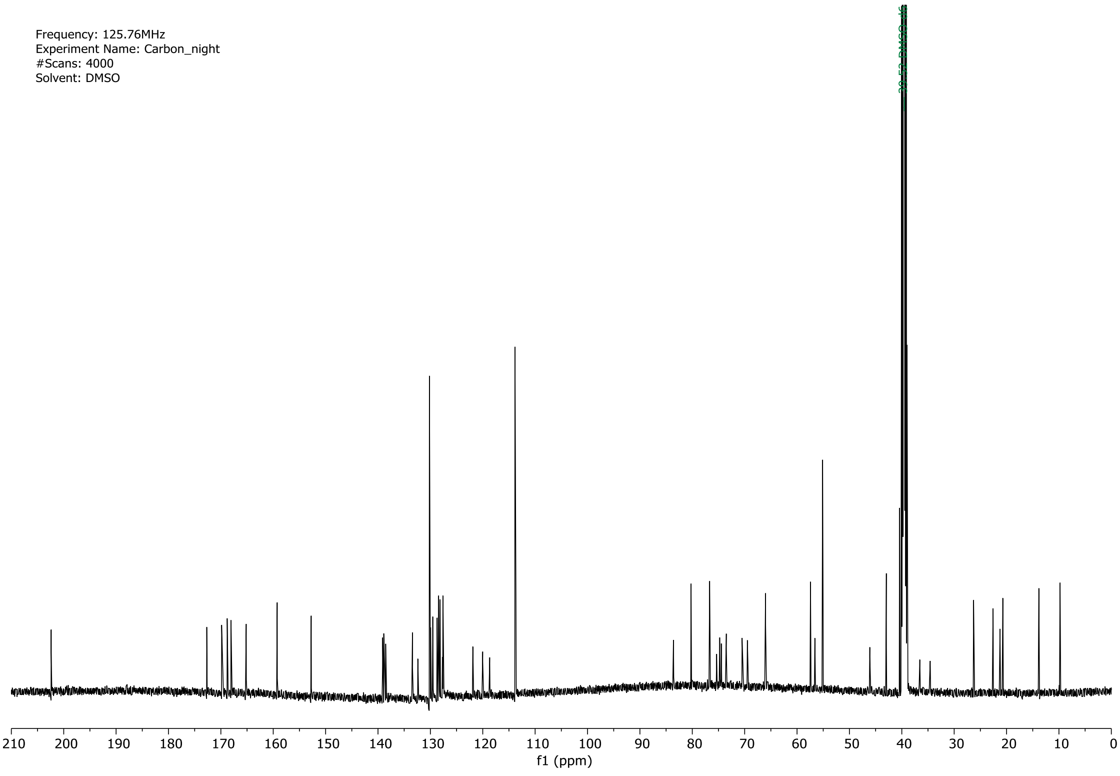

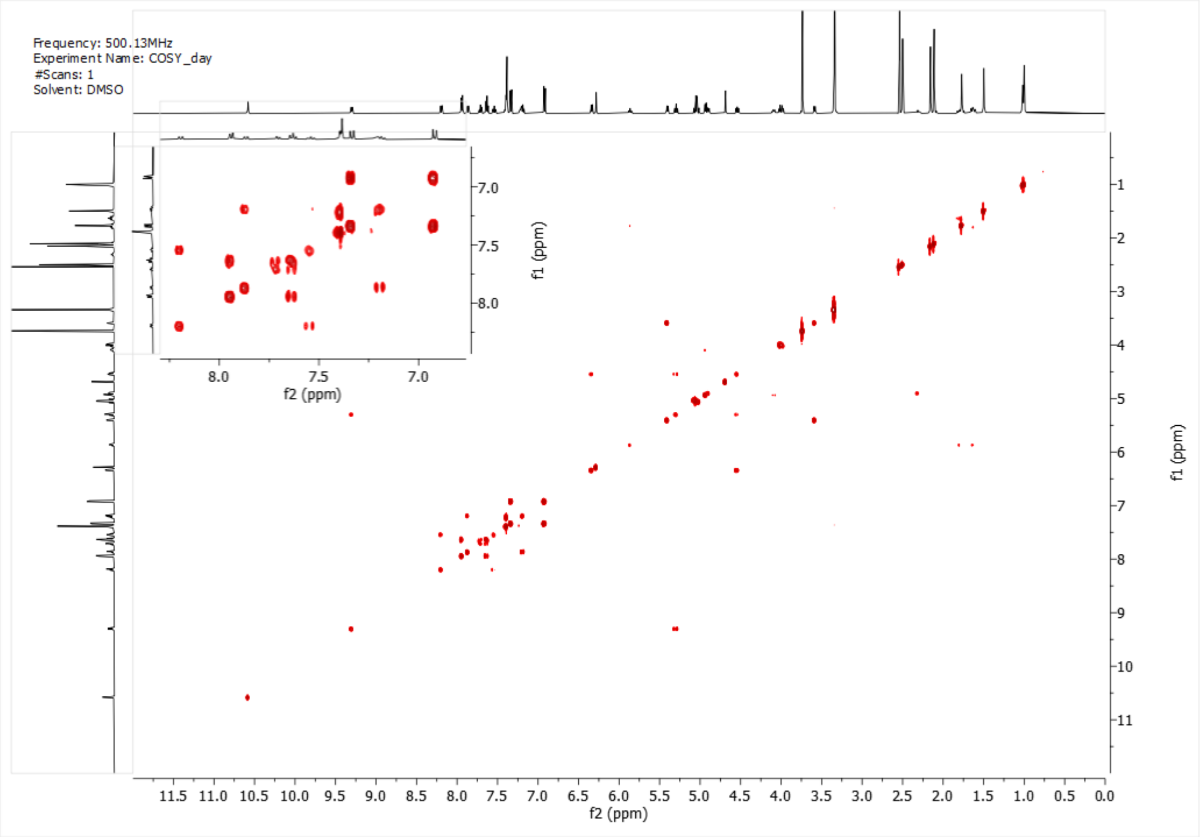

Frequency: 500.13MHz
Experiment Name: COSY_day
#Scans: 1
Solvent: DMSO
f2 (ppm)
f1 (ppm)

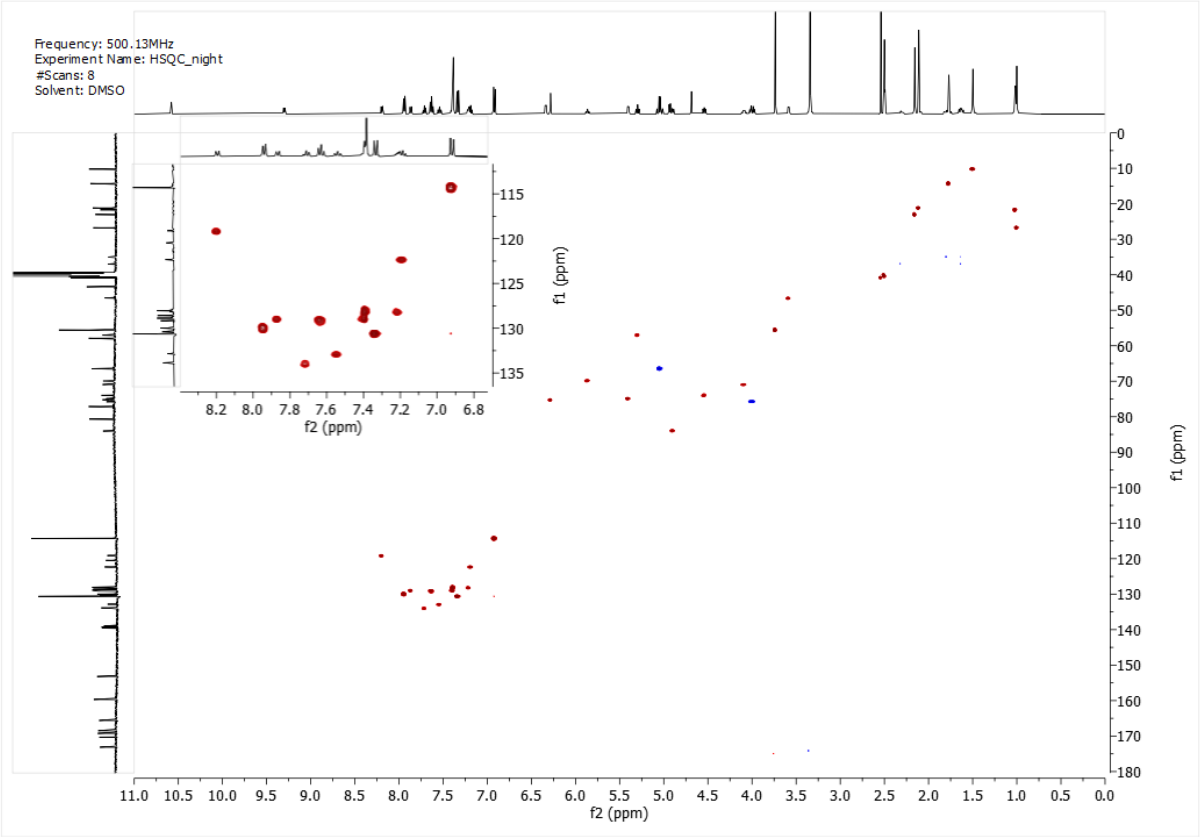

Frequency: 500.13MHz
Experiment Name: HSQC_night
#Scans: 8
Solvent: DMSO
f2 (ppm)
f1 (ppm)

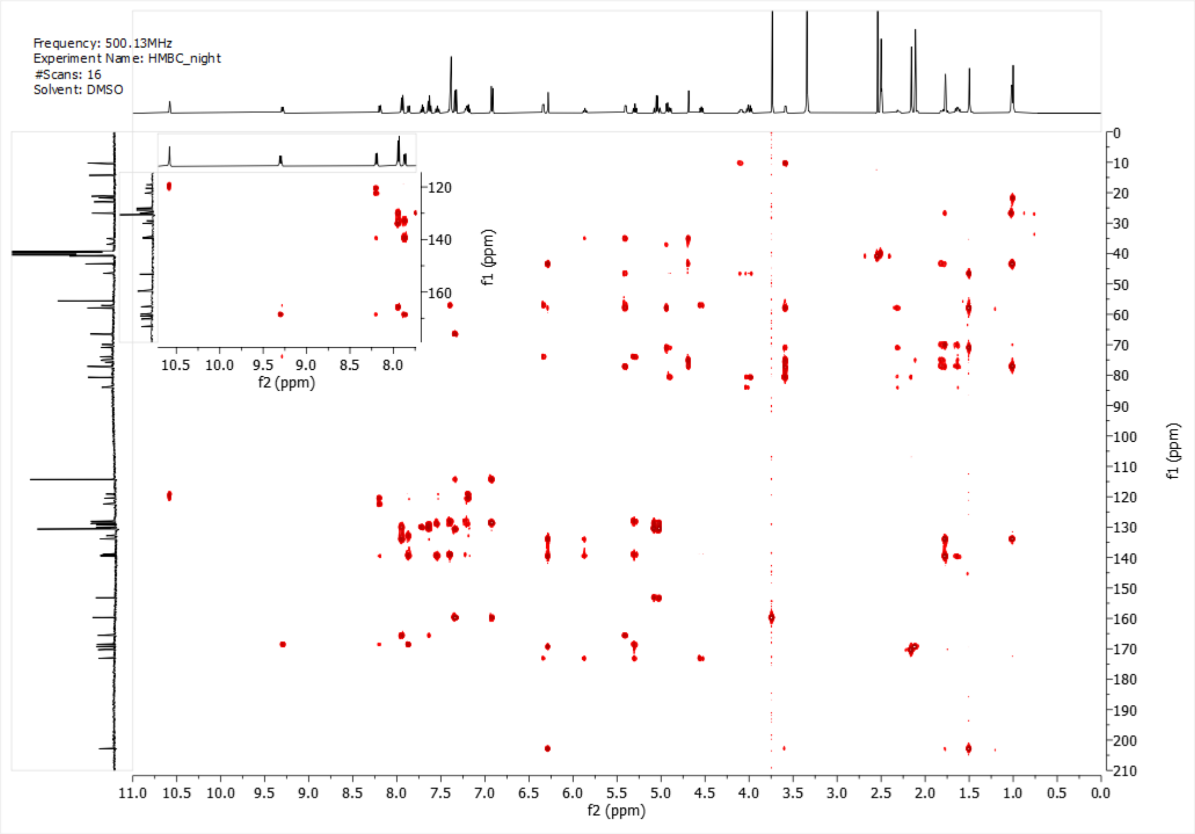

Frequency: 500.13MHz
Experiment Name: HMBC_night
#Scans: 16
Solvent: DMSO
f2 (ppm)
f1 (ppm)

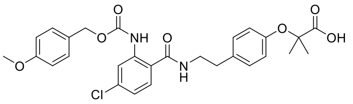

2-(4-(2-(4-chloro-2-((((4-methoxybenzyl)oxy)carbonyl)amino)benzamido)ethyl)phenoxy)-2-methylpropanoic acid (**3j**)

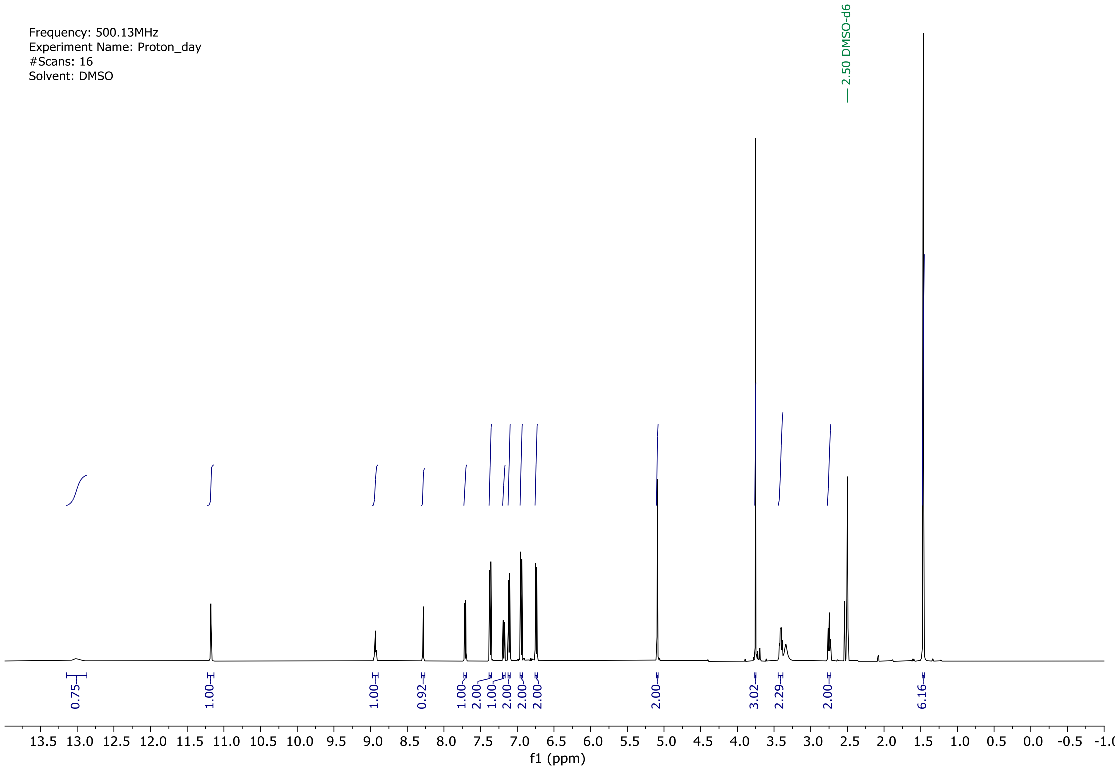

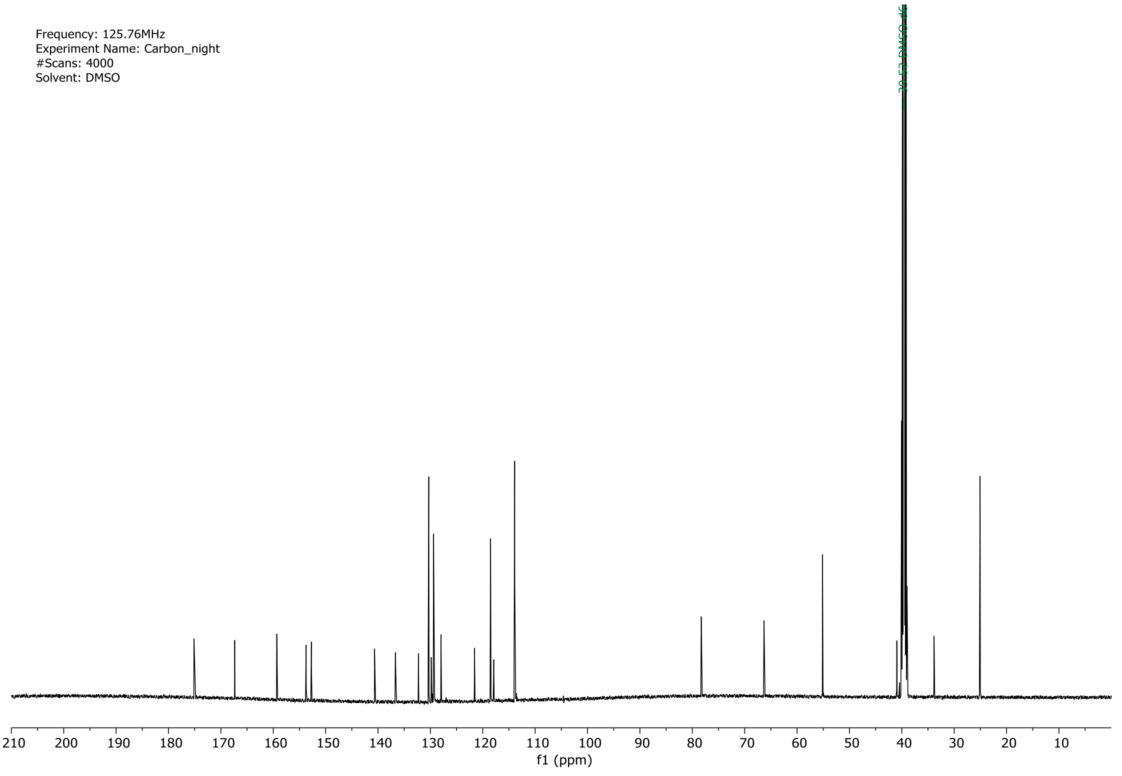

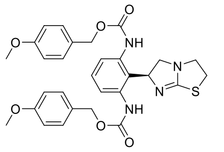

Bis(4-methoxybenzyl) (2-(2,3,5,6-tetrahydroimidazo[2,1-b]thiazol-6-yl)-1,3-phenylene)(S)-dicarbamate (**3k**)

Frequency: 500.13MHz
Experiment Name: Proton_day
#Scans: 16
Solvent: DMSO

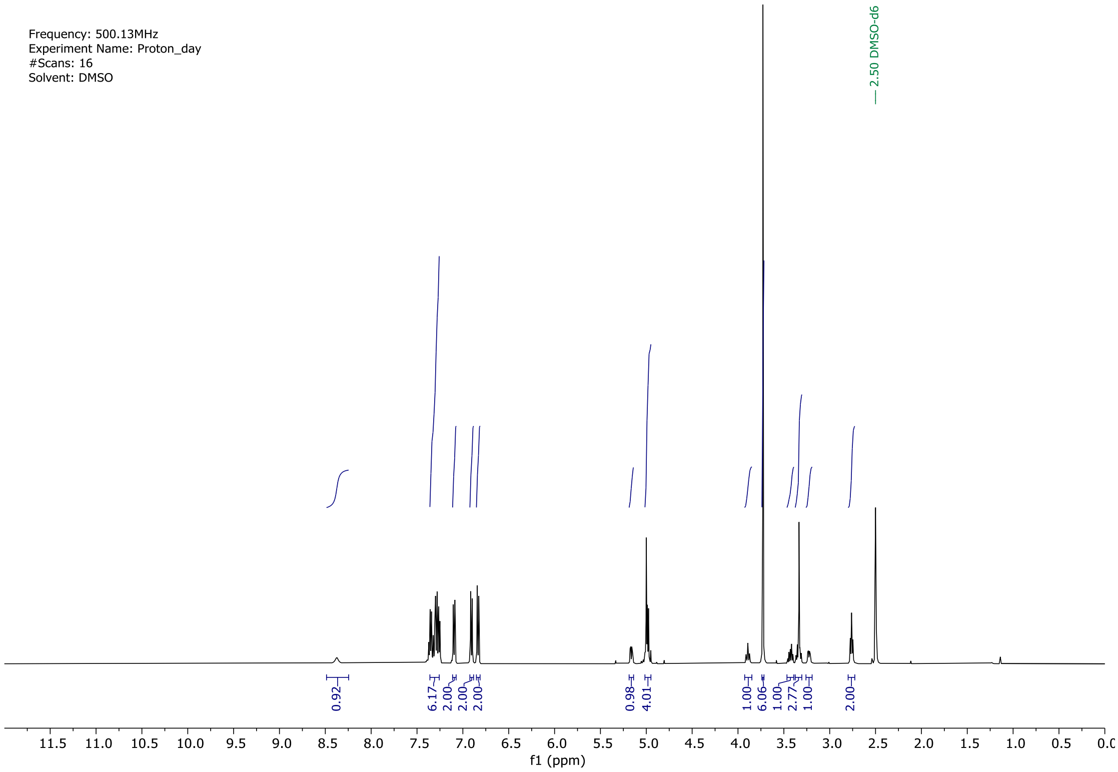

Frequency: 150.94MHz
Experiment Name: Carbon_night
#Scans: 4000
Solvent: DMSO

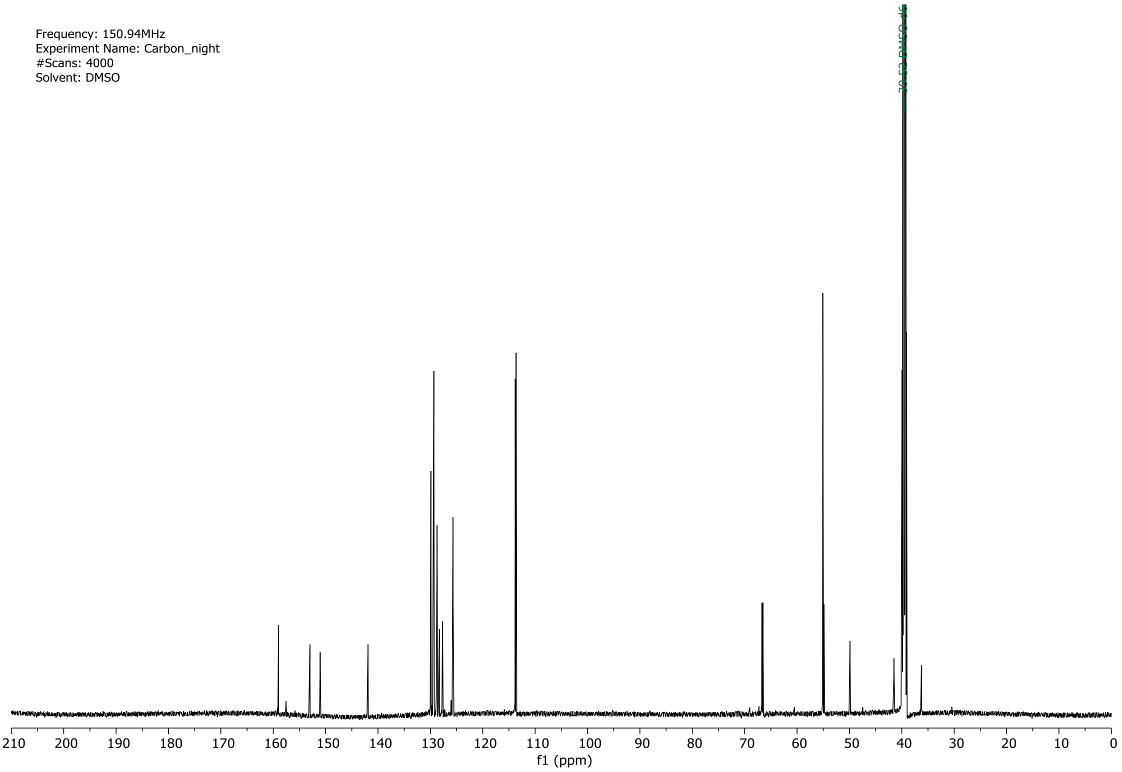

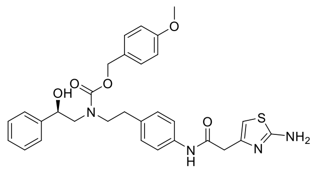

4-methoxybenzyl (R)-(4-(2-(2-aminothiazol-4-yl)acetamido)phenethyl)(2-hydroxy-2-phenylethyl)carbamate (**3l**)

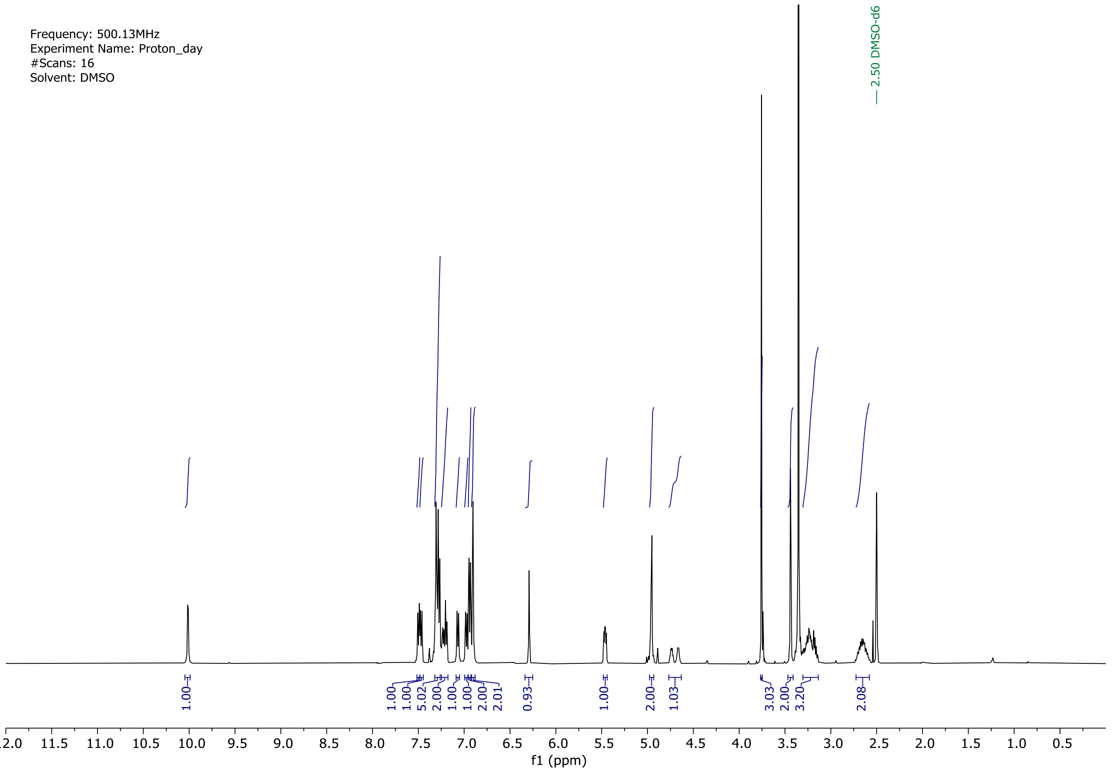

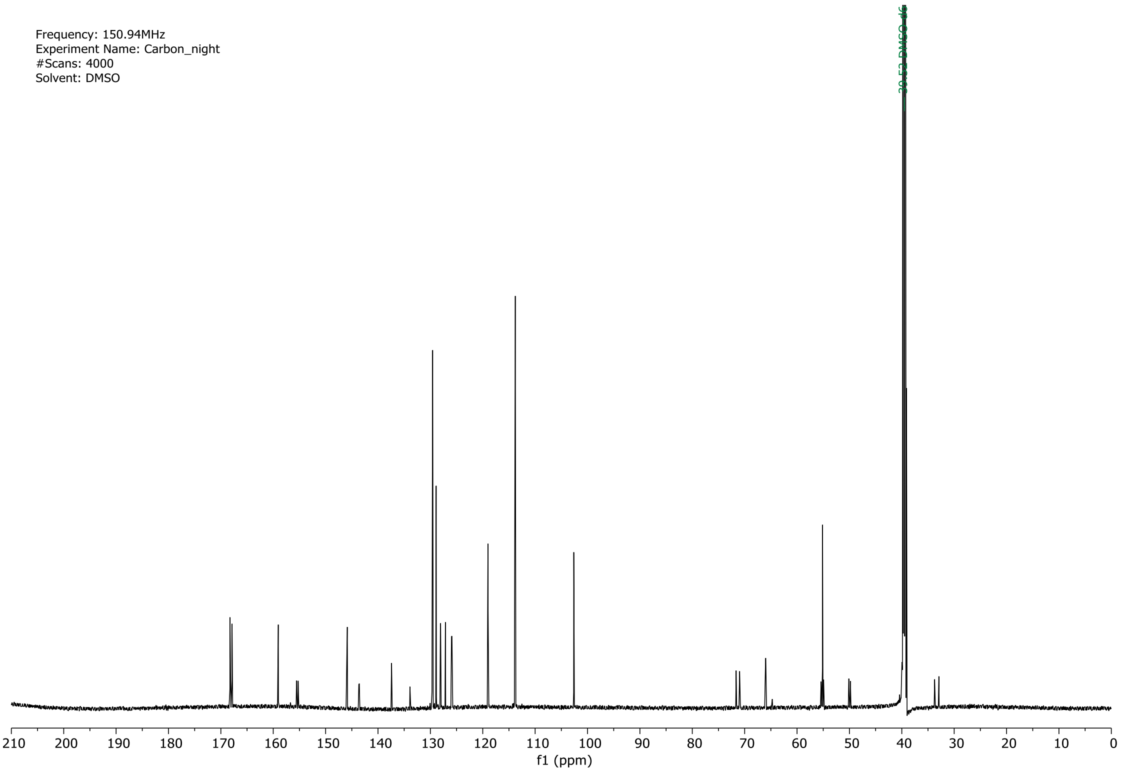

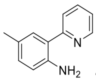

4-methyl-2-(pyridin-2-yl)aniline (**4a**)

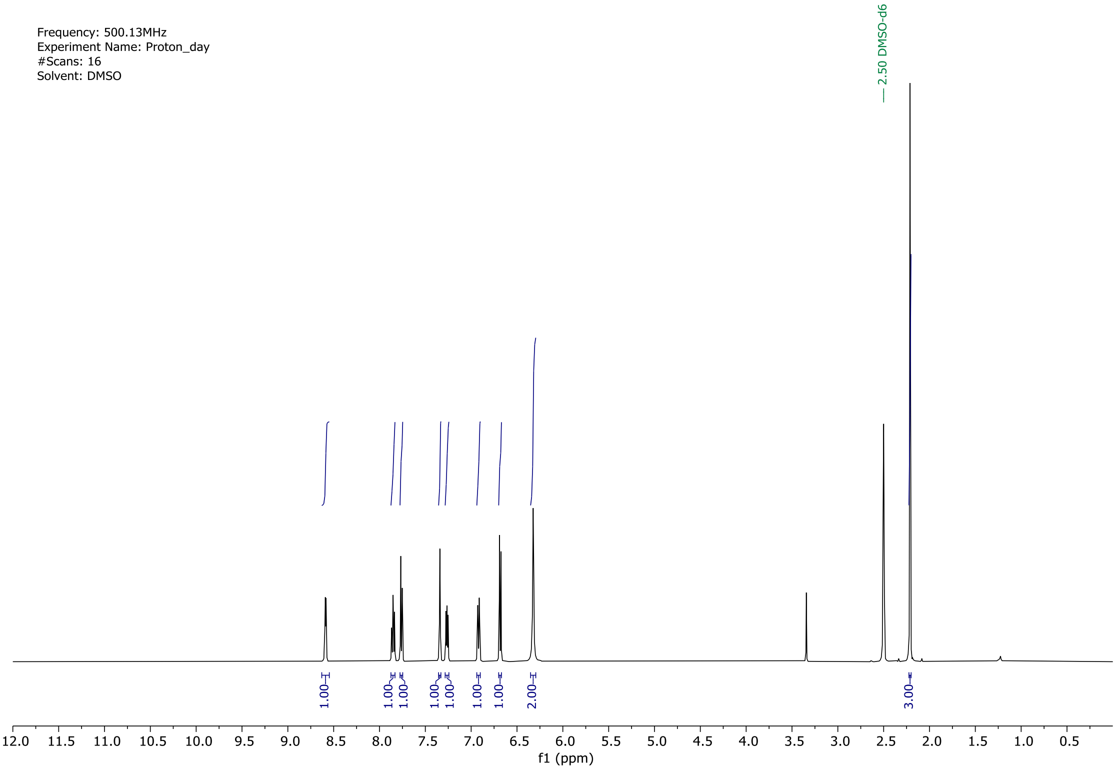

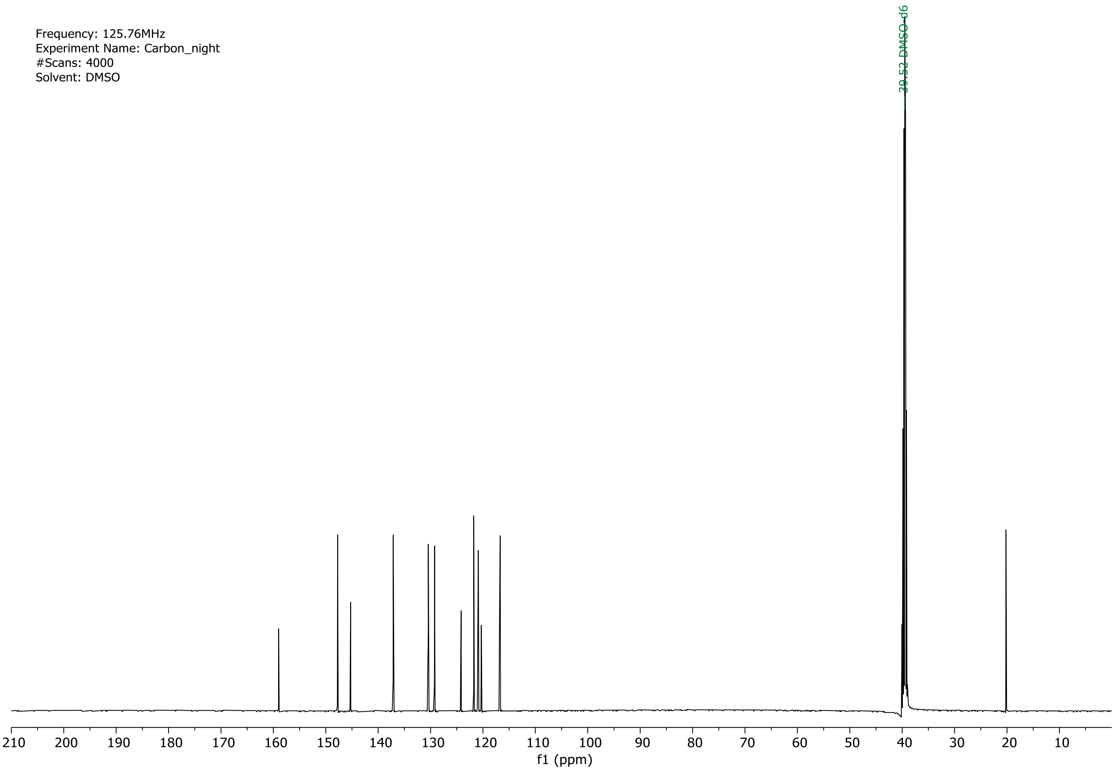

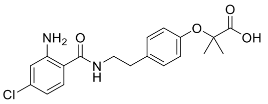

2-(4-(2-(2-amino-4-chlorobenzamido)ethyl)phenoxy)-2-methylpropanoic acid (**4b**)

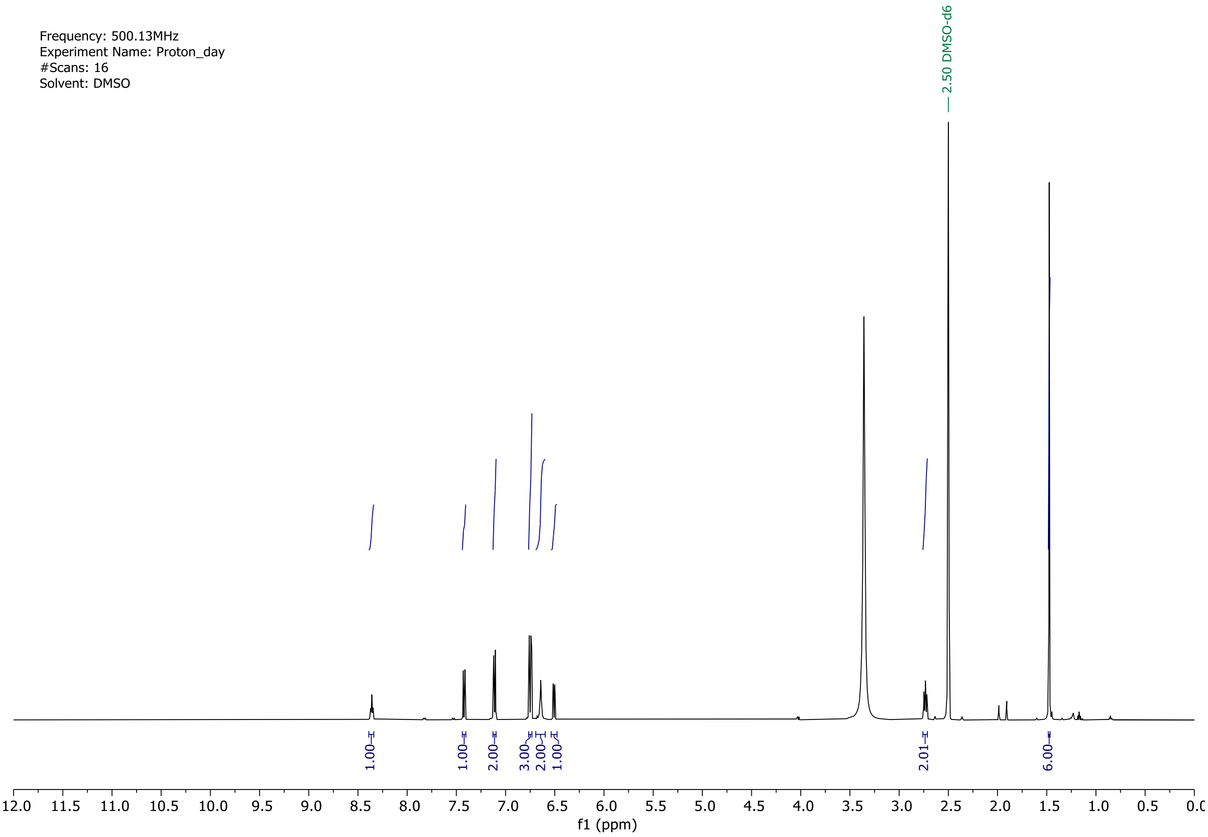

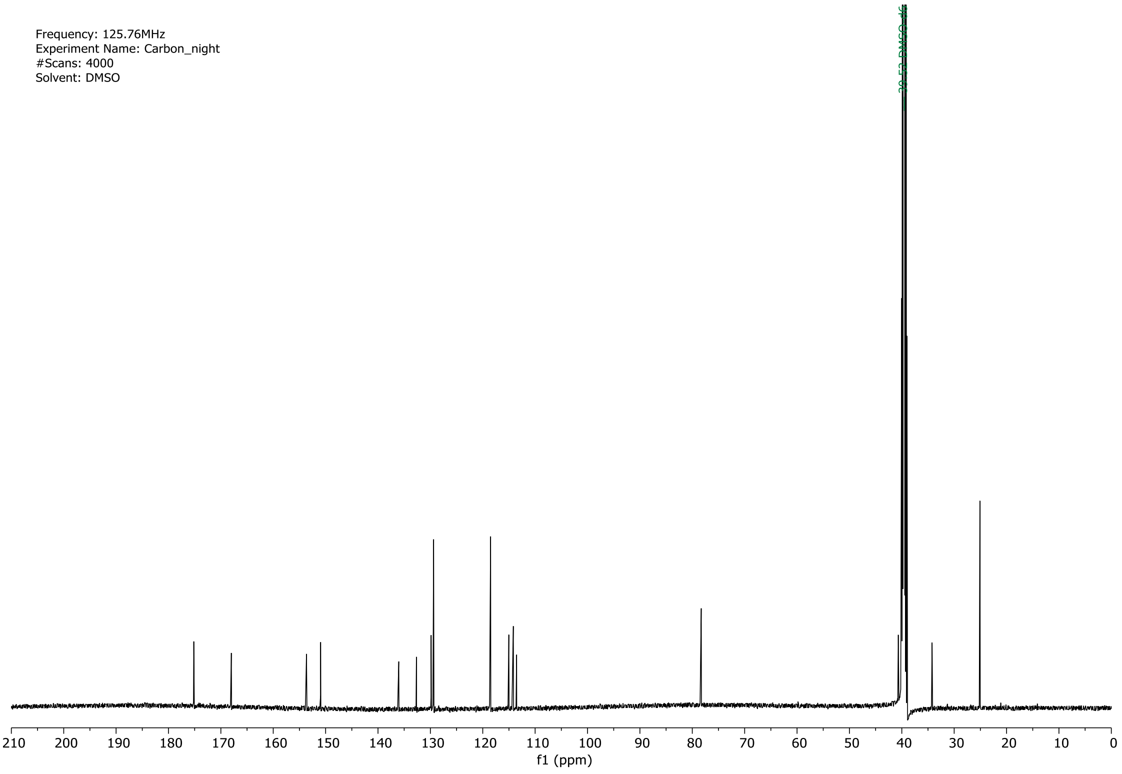

References

(1) Sinclair, I.; Bachman, M.; Addison, D.; Rohman, M.; Murray, D. C.; Davies, G.; Mouchet, E.; Tonge, M. E.; Stearns, R. G.; Ghislain, L.; Datwani, S. S.; Majlof, L.; Hall, E.; Jones, G. R.; Hoyes, E.; Olechno, J.; Ellson, R. N.; Barran, P. E.; Pringle, S. D.; Morris, M. R.; Wingfield, J. Acoustic Mist Ionization Platform for Direct and Contactless Ultrahigh-Throughput Mass Spectrometry Analysis of Liquid Samples. *Anal. Chem.* **2019,** *91* (6), 3790-3794.

(2) Weis, E.; Johansson, M. J.; Martín-Matute, B. Late-stage amination of drug-like benzoic acids: Access to anilines and drug conjugates via directed iridium-catalyzed C−H activation. *Chem. Eur. J.*, **2021**, *27* (72), 18188-18200.

(3) Kim, H.; Park, G.; Park, J.; Chang, S. A Facile Access to Primary Alkylamines and Anilines via Ir(III)-Catalyzed C–H Amidation Using Azidoformates. *ACS Catal.* **2016,** *6* (9), 5922-5929.
